# Efficacy of a 12-Week Simeprevir Plus Peginterferon/Ribavirin (PR) Regimen in Treatment-Naïve Patients with Hepatitis C Virus (HCV) Genotype 4 (GT4) Infection and Mild-To-Moderate Fibrosis Displaying Early On-Treatment Virologic Response

**DOI:** 10.1371/journal.pone.0168713

**Published:** 2017-01-05

**Authors:** Tarik Asselah, Christophe Moreno, Christoph Sarrazin, Michael Gschwantler, Graham R. Foster, Antonio Craxí, Peter Buggisch, Faisal Sanai, Ceyhun Bicer, Oliver Lenz, Gino Van Dooren, Catherine Nalpas, Isabelle Lonjon-Domanec, Michael Schlag, Maria Buti

**Affiliations:** 1 Hepatology Department, Beaujon Hospital, University of Paris, Paris, France; 2 CUB Hôpital Erasme, Université Libre de Bruxelles, Brussels, Belgium; 3 Johann Wolfgang Goethe University Hospital, Medizinische Klinik 1, Frankfurt am Main, Germany; 4 Department of Internal Medicine IV, Wilhelminenspital, Vienna, Austria; 5 Queen Mary Hospital, University of London, Barts Health, London, United Kingdom; 6 Sezione di Gastroenterologia & Epatologia, Di.Bi.M.I.S., University of Palermo, Palermo, Italy; 7 Institute for Interdisciplinary Medicine at the Asklepios Klinik St. Georg, Hamburg, Germany; 8 Division of Gastroenterology, Department of Medicine, King Abdulaziz Medical City, Jeddah, Saudi Arabia; 9 Janssen Infectious Diseases BVBA, Beerse, Belgium; 10 Janssen Pharmaceuticals, Paris, France; 11 Janssen-Cilag, Vienna, Austria; 12 Liver Unit, Hospital Valle Hebron and Ciberehd del Institut Carlos III, Barcelona, Spain; University of New South Wales, AUSTRALIA

## Abstract

**Background:**

HCV GT4 accounts for up to 20% of HCV infections worldwide. Simeprevir, given for 12 weeks as part of a 24- or 48-week combination regimen with PR is approved for the treatment of chronic HCV GT4 infection. Primary study objectives were assessment of efficacy and safety of simeprevir plus PR in treatment-naïve patients with HCV GT4 treated for 12 weeks. Primary efficacy outcome was sustained virologic response 12 weeks post-treatment (SVR12). Additional objectives included investigation of potential associations of rapid virologic response and baseline factors with SVR12.

**Methods:**

This multicentre, open-label, single-arm study (NCT01846832) evaluated efficacy and safety of simeprevir plus PR in 67 patients with HCV GT4 infection. Patients were treatment-naïve, aged 18–70 years with METAVIR F0–F2 fibrosis. Patients with early virologic response (HCV RNA <25 IU/mL [detectable/undetectable in *IL28B* CC patients or undetectable in *IL28B* CT/TT patients] at Week 2 and undetectable at Weeks 4 and 8) were eligible to stop all treatment at the end of Week 12, otherwise PR therapy was continued to Week 24.

**Results:**

Of 67 patients treated, 34 (51%) qualified for 12-week treatment including all but one patient with *IL28B* CC genotype (14/15). All patients in the 12-week group had undetectable HCV RNA at end of treatment, and 97% (33/34) achieved SVR12. No new safety signals with simeprevir plus PR were identified. The proportion of patients experiencing Grade 3–4 adverse events was lower in the 12-week group than in the 24-week group.

**Conclusions:**

Our findings on simeprevir plus PR therapy shortened to 12 weeks in patients with HCV GT4 infection with favourable baseline characteristics and displaying early on-treatment virologic response are encouraging. No new safety signals were associated with simeprevir plus PR in this study.

**Trial Registration:**

NCT01846832

## Introduction

Hepatitis C virus (HCV) is among the leading causes of liver disease, estimated to affect around 180 million people worldwide [1]. Untreated HCV infection puts patients at risk of developing cirrhosis and hepatocellular carcinoma. Fortunately, cure of HCV infection is now possible in most cases [2]. Sustained virologic response (SVR), defined as undetectable HCV RNA 12 weeks after end of treatment (SVR12), is associated with cure of the infection in >99% of patients and is the primary measure of efficacy of HCV treatment [1].

HCV variants are classified into seven genotypes whose coding region sequences differ by at least 15% [3]. HCV treatment efficacy varies according to HCV genotype [4]. HCV genotype 4 (GT4) has not, until recently, been the focus of the same extent of research as GT1–3, although GT4 is responsible for approximately 34 million HCV infections worldwide, and represents up to 20% of all cases [5]. GT4 is particularly widespread in the Middle East and sub-Saharan Africa, where it accounts for over 80% of cases [6, 7]. It is becoming increasingly prevalent in Southern Europe, as a result of migration and spread of the virus through injection drug users [7, 8].

HCV GT4 has long been considered to be one of the more difficult genotypes to treat, since peginterferon-α (IFN; P) and ribavirin (RBV; R))-only regimens are only partially effective in patients with HCV GT4 [9, 10]. Historically, treatment of HCV GT4 with PR necessitated a 48-week treatment regimen and achieved SVR rates of 43–70% [10].

The advent of IFN-free DAA regimens has revolutionised HCV treatment. SVR rates exceeding 90% have been achieved across all genotypes, even in treatment-experienced patients and those with advanced fibrosis, in randomised trials and real-world studies. Regimens demonstrating effectiveness in GT4 with 8 or 12 weeks of treatment include ombitasvir/paritaprevir/ritonavir (OBV/PTV/r) [11–15], simeprevir plus sofosbuvir [16], elbasvir plus grazoprevir [17], and sofosbuvir plus ledipasvir [18]. Furthermore, pan-genotypic DAA therapies such as velpatasvir, which has recently demonstrated an SVR12 rate approaching 100% when administered for 12 weeks in combination with sofosbuvir [19], have the potential to eliminate the need for genotyping and thereby greatly simplify treatment. GS-9857 [20, 21] and ravidasvir [22] also show significant promise in this area; the latter has demonstrated a 97% SVR12 rate in 182 non-cirrhotic patients with HCV GT4 in Egypt when given for 12 weeks in combination with sofosbuvir [23].

However, the cost of DAAs and restricted availability remain significant barriers to access to treatment for many HCV patients even in the most developed countries [24]. These barriers are particularly formidable for regimens containing two or more DAAs. Even in 2016, access to IFN-free regimens continues to be restricted to patients with advanced liver disease (F3–F4 fibrosis) and/or those who cannot tolerate IFN in many European and Asian countries [25, 26]. Single-DAA IFN-based regimens may broaden the range of patients able to access effective treatment earlier in the disease course, forestalling the progress of liver disease, decreasing the risk of hepatocellular carcinoma, and increasing the likelihood of success with short-course treatment. The clinical management and adverse event burdens associated with PR therapy [27] mean that decreasing treatment duration is very appealing to patients and physicians.

Simeprevir is a once-daily DAA approved for use in combination with PR or with sofosbuvir in HCV GT1 and GT4 [28, 29]. Simeprevir plus PR has demonstrated SVR rates of 83% in treatment-naïve patients and 86% in prior relapsers with GT4 following 12 weeks of simeprevir plus 24–48 weeks of PR [30].

Previous findings suggest that the overall duration of IFN-based therapy in combination with a protease inhibitor could be shortened to 12 weeks in some patients without reducing its efficacy [31]. In patients infected with HCV GT1 and F0–F2 fibrosis only, SVR12 rates of >80% were reported in patients with *IL28B* CC genotype (94%) or HCV RNA ≤800,000 IU/mL (82%) who received simeprevir plus PR for 12 weeks after achieving early on-treatment response [32]. Recent reports suggest that timely initiation of therapy in patients with early-stage fibrosis could be associated with improved long-term outcomes and reduced HCV-associated mortality [33]. The analysis described here was conducted to assess the potential for shortening the total duration of simeprevir plus PR treatment to 12 weeks in treatment-naïve patients with HCV GT4 and F0–F2 fibrosis displaying an early on-treatment virologic response.

## Materials and Methods

### Patients and study design

A multicentre, open-label, single-arm, phase III clinical trial evaluating efficacy and safety of simeprevir plus PR in treatment-naïve patients with HCV GT1 (reported separately [32]) and GT4 infection took place between 3 September 2013 (first patient screened) and 31 August 2015 (date of last patient contact). The study was approved by each centre’s institutional review board and conducted in accordance with the Declaration of Helsinki and Good Clinical Practice guidelines. The review boards are listed in S1 Text. Here we report data in the GT4 population.

Patients with HCV GT4 were recruited from centres in Austria, Belgium, France, Italy, Saudi Arabia and Spain. Written informed consent was obtained from all patients at screening prior to the performance of any study-specific assessments or procedures. Each patient received an identification code to permit easy identification of each patient during and after the study. The code list was treated as confidential and filed by the investigator in the study file.

Eligible patients were treatment-naïve adults aged 18 to 70 years with chronic HCV GT4 infection confirmed at least 6 months prior to screening, plasma HCV RNA >10,000 IU/mL, and mild-to-moderate liver fibrosis (METAVIR F0–F2). HCV genotype subtype is based on the NS5B assay or, if data were unavailable, on LIPA HCV II or TRUGENE results. Liver fibrosis score was confirmed by liver biopsy within 2 years of screening or non-invasive assessment of liver disease stage by transient elastography performed within 6 months of screening [34]. For patients with liver biopsy data, lesions were evaluated according to the METAVIR scoring system whereby fibrosis was staged on a 0–4 scale (F0: no fibrosis; F1: portal fibrosis without septa; F2: few septa; F3: numerous septa without cirrhosis; F4: cirrhosis) [34, 35]. For non-invasive fibrosis assessment, the following cut-offs were defined: FibroScan™ F0–F1 <6.9 kPa, F2 <8 kPa [36, 37]; magnetic resonance elastography F0–F1 <2.5 kPa; F2 <3.1 kPa [38]. For other methods, such as shear wave elastography, the advice of the sponsor was taken.

Exclusion criteria included advanced liver disease (METAVIR F3–F4) or liver disease of aetiology other than HCV; previous or current hepatic decompensation; co-infection with hepatitis B virus or HIV; significant abnormalities in laboratory parameters; pregnancy, breast-feeding, or plans to conceive within 6 months of end of treatment.

Patients received simeprevir (150 mg once-daily, orally) in combination with peg-IFN alfa-2a (180 μg, weekly by subcutaneous injection) and RBV (1000 mg/day if body weight <75 kg, or 1200 mg/day if >75 kg, orally) [Simeprevir SmPC]. Total treatment duration was determined on the basis of on-treatment response, with HCV RNA levels determined by Roche Cobas^®^ TaqMan v2.0^®^ assay [lower limit of quantification 25 IU/mL; limit of detection 15 IU/mL]. Treatments were stored and administered by participants in accordance with instructions provided by study site personnel. The investigator or designated study site personnel maintained a log of all study treatments dispensed and returned for compliance assessment purposes.

To be eligible for the 12-week regimen, patients were required to display HCV RNA <25 IU/mL at Week 2 (detectable or undetectable in patients with *IL28B* CC genotype, undetectable in those with CT or TT genotype), and undetectable HCV RNA at Weeks 4 and 8. For all patients, the planned duration of simeprevir treatment was 12 weeks. Patients fulfilling the 12-week eligibility criteria also discontinued PR at Week 12; otherwise, PR treatment was continued for another 12 weeks following discontinuation of simeprevir (S1 Fig). Patients who stopped treatment prior to Week 8 were assigned to the 24-week treatment group for the purpose of the analysis.

Patients discontinued all treatment if they met any of the following virologic stopping rules: HCV RNA concentration ≥25 IU/mL at Week 4; HCV RNA <25 IU/mL detectable or ≥25 IU/mL at Week 12; or viral breakthrough (defined as a confirmed increase of >1 log_10_ IU/mL in HCV RNA concentration from the lowest level reached, or a confirmed HCV RNA level of >100 IU/mL in patients whose HCV RNA level had previously been <25 IU/mL [detectable or undetectable] while on study treatment).

### Objectives

The objectives of the present study were to determine the efficacy, defined in terms of the proportion of patients achieving SVR12, and the safety and tolerability of simeprevir plus PR in patients with HCV GT4 eligible for the shortened (12-week) treatment regimen. SVR12 was defined as HCV RNA <25 IU/mL undetectable 12 weeks after the planned end of all study treatment. SVR12 is considered equivalent to SVR 24 weeks after end of treatment (SVR24).

Efficacy and safety of simeprevir plus PR in patients with HCV GT4 receiving 24 weeks of treatment were also assessed, as were early virologic responses at Weeks 2 and 4, SVR24, and on-treatment HCV RNA levels.

### Evaluations

Blood samples for determination of HCV RNA levels were collected at screening and/or baseline, at each on-treatment study visit (Weeks 2, 4, 8, 12, 16, 20 and 24), and at the follow-up visits (Weeks 1, 2, 4, 8, 12, 16, 20, 24, 28, 36, and 48), as pre-specified in the time and events schedule, or at the time of discontinuation of treatment. Genotyping of the rs12979860 single nucleotide polymorphism of *IL28B*, previously shown to be strongly associated with SVR in patients with HCV GT4 [9], was conducted at screening. Adverse events (AEs), as reported by the patient or appropriate caregiver, were recorded throughout the study. Descriptions of AEs were coded using the Medical Dictionary for Regulatory Activities. All reported AEs occurring during the treatment phase were included in the safety analysis, in which the percentage of patients experiencing each recorded AE was summarised according to treatment group.

Blood samples collected at each study visit were used for biochemical and haematological analyses. Electrocardiograms, vital sign assessments and physical examinations were also performed throughout the study period.

### Statistical methods

The efficacy analysis population was the intent-to-treat (ITT) population, consisting of all study participants with HCV GT4 who received at least one dose of study medication. Summary statistics for the effects of treatment and of viral clearance are reported for each treatment group. Baseline factors associated with treatment cessation and eligibility for 12 weeks were also examined. Statistical analysis was conducted using SAS statistical analysis software (SAS Institute Inc.). P-values for assessment of differences in demographic characteristics and adverse event incidences between 12- and 24-week groups were calculated using the Chi-squared test (categorical parameters) or Wilcoxon-Mann-Whitney test (continuous parameters).

## Results

### Patient characteristics and disposition

The ITT population comprised 67 patients with HCV GT4 who received study treatment. The majority of patients were male (69%) and white (80%), with 75% located in Europe, and the remaining 25% being based in Saudi Arabia (Table 1). Most patients were infected with either HCV GT4a (40%) or GT4d (37%) (S1 Table). Patients mainly had mild fibrosis (F0–F1, 81%), and 22% had *IL28B* CC genotype.

**Table 1:** Patient Demographics and Disease Characteristics at Baseline for Patients with HCV GT4 by Treatment Duration and Overall (n = 67).

	duration		
	12 weeks (n = 34)	24 weeks (n = 33)	All patients (N = 67)
**Male, n (%)**	23 (68)	23 (70)	46 (69)
**Median age (range), years**	47.5 (19–63)	48 (21–66)	48 (19–66)
Race, n/N (%)*			
**White**	23/30 (77)	24/29 (83)	47/59 (80)
**Black or African American**	3/30 (10)	4/29 (14)	7/59 (12)
**Asian**	2/30 (7)	1/29 (3)	3/59 (5)
**Multiple**	2/30 (7)	0	2/59 (3)
**Geographical region, n/N (%)**			
**Europe**	24 (71)	26 (79)	50 (75)
**Saudi Arabia**	10 (29)	7 (21)	17 (25)
**Body mass index, kg/m^2^, median (interquartile range)**	24.6 (22.1, 27.5)	27.5 (24.8, 30.4)	26.3 (23.0, 29.6)
**HCV genotype subtype^†^, n (%)**			
**4**	1 (3)	1 (3)	2 (3)
**4a**	14 (41)	13 (39)	27 (40)
**4a/4c/4d**	1 (3)	1 (3)	2 (3)
**4c**	1 (3)	1 (3)	2 (3)
**4d**	13 (38)	12 (36)	25 (37)
**Other 4 subtypes^‡^**	4 (12)	5 (15)	9 (13)
**Baseline HCV RNA, log_10_ IU/mL, median (range)**	5.8 (3.1–7.5)	6.4 (4.8–7.2)	6.1 (3.1–7.5)
**Baseline HCV RNA, IU/mL, n (%)**			
**<400,000**	11 (32)	3 (9)	14 (21)
**≥**400,000 –**≤**800,000	8 (24)	4 (12)	12 (18)
**>800,000**	15 (44)	26 (79)	41 (61)
**METAVIR score, n (%)**			
**F0–F1**	29 (85)	25 (76)	54 (81)
**F2**	5 (15)	7 (21)	12 (18)
**F3**	–	1 (3)	1 (1)
***IL28B* genotype subtype, n (%)**			
**CC**	14 (41)	1 (3)	15 (22)
**CT**	15 (44)	27 (82)	42 (63)
**TT**	5 (15)	5 (15)	10 (15)

GT4, genotype 4; HCV, hepatitis C virus.

*Race data unavailable for 8 patients: not allowed to ask per local regulations.

^†^HCV genotype subtype is based on the NS5B assay, and if not available on LIPA HCV II or Trugene results.

^‡^Other subtypes were 4e, 4f, 4k, 4n, 4q and 4r.

**Fig 1:**
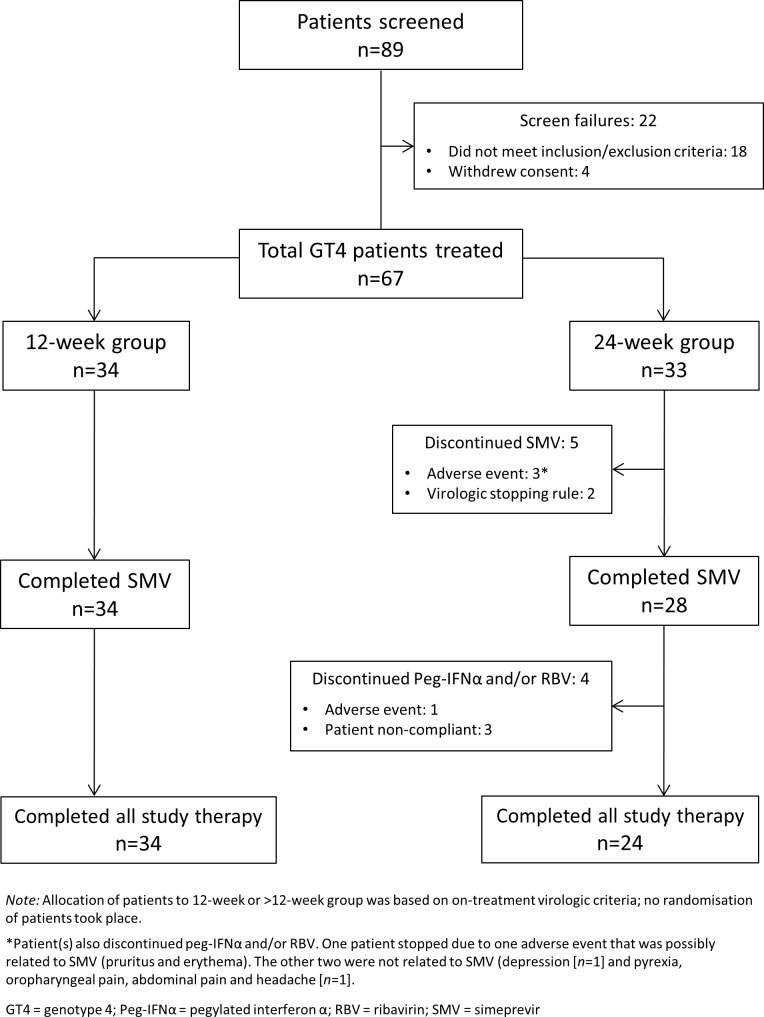
Patient Disposition Flow Chart for Patients with HCV GT4.

Of the 67 GT4 patients enrolled in the study, 34 (51%) met the criteria for 12-week treatment with all 34 completing 12 weeks of simeprevir plus PR (Fig 1).

Median body mass index was significantly greater (Wilcoxon-Mann-Whitney p = 0.01) in the 24-week group than in the 12-week group. Patients in the 24-week group also had significantly higher median HCV RNA levels (Wilcoxon-Mann-Whitney p = 0.011) and were more likely to have non-CC *IL28B* genotype (Chi-Squared p = 0.00065) than those in the 12-week group. All other patient characteristics assessed (Table 1) were not significantly different between the two groups.

Of the 33 patients who were not eligible for the 12-week regimen and were therefore assigned to receive 24 weeks of treatment, 28 (85%) completed simeprevir and 24 (73%) completed all study therapy. Five patients in the 24-week group discontinued simeprevir: two met a virologic stopping rule, and three discontinued treatment because of an AE. Two of these discontinuations were considered not related to simeprevir; the other patient had AEs considered to be probably related to simeprevir (erythema [Grade 1] and pruritus [Grade 2]). Nine patients in the 24-week group discontinued PR: five as described above, who also discontinued simeprevir, one patient who discontinued PR only [due to erythema nodosum]) and three because of non-compliance (Fig 1). All patients who discontinued PR were in the PR-only treatment phase; no patients received simeprevir monotherapy.

### Eligibility of patients for 12 weeks total treatment duration

**Fig 2:**
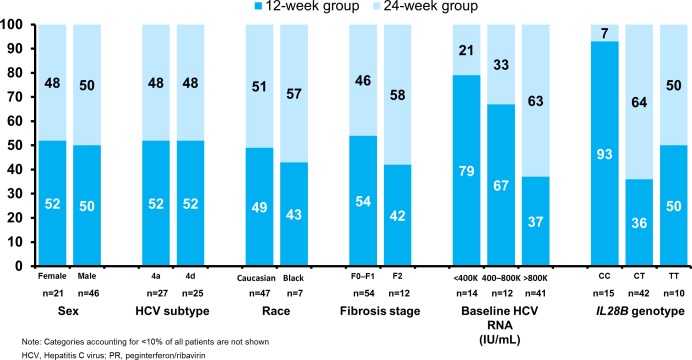
Eligibility for 12-week simeprevir plus PR regimen according to patient baseline characteristics.

Patients with *IL28B* CC genotype appeared more likely to stop all treatment at Week 12 (93% of *IL28B* CC patients compared with 36% of CT and 50% of TT patients); the difference in response guided therapy criteria for *IL28B* CC versus non-CC patients did not explain this observation, since 12/14 of *IL28B* CC patients in the 12-week group had undetectable HCV RNA at Week 2. A greater proportion of patients with low RNA viral load at baseline were eligible for the 12-week group–respectively, 79% of patients with HCV RNA < 400,000 IU/mL, 67% of patients with HCV RNA 400,000–800,000 IU/mL, and 37% of patients with HCV RNA > 800,000 IU/ml qualified to stop treatment at Week 12. There were small or no differences in 12-week eligibility rates according to gender, HCV 4 subtype, race, geographical region, or fibrosis stage on eligibility for 12 weeks versus 24 weeks of treatment (Fig 2; Table 1). The minimal dataset of the present study is available in S1 Dataset.

### Efficacy

Among all patients with GT4 receiving 12 or 24 weeks of simeprevir plus PR, 90% (60/67) achieved SVR12.

**Fig 3:**
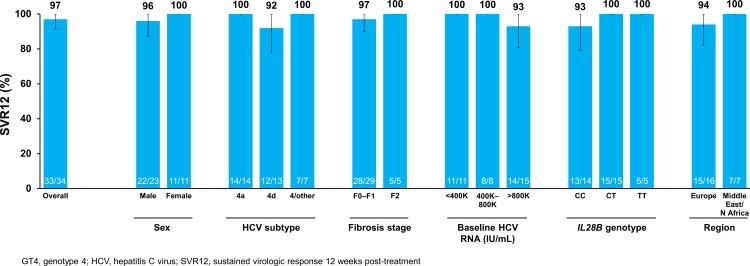
SVR12 among patients with HCV GT4 in the 12-week treatment group.

All patients with GT4 receiving the 12-week treatment regimen had undetectable HCV RNA at end of treatment; 97% (33/34) achieved SVR12 (Fig 3) and SVR24 (Data Not Shown). The one patient in the 12-week group who did not achieve SVR12 relapsed 58 days after the end of treatment. This patient was male, infected with HCV GT4d, had *IL28B* CC genotype, F0–F1 fibrosis and had a baseline HCV RNA of 2,080,000 IU/mL.

Among those GT4 patients assigned to the 24-week treatment group, 82% (27/33) achieved SVR12 and SVR24. Three patients experienced on-treatment failure: two met the Week 4 stopping rule (HCV RNA ≥25 IU/mL at Week 4) and one discontinued all study drugs during the first week of therapy due to pyrexia, oropharyngeal pain, headache and abdominal pain, and had detectable HCV RNA at time of discontinuation.

Three patients in the 24-week group experienced relapse post-treatment (two completed PR and one stopped all treatment at Week 8 due to depression) (S2 Table). The two patients who had completed the 24 weeks of treatment relapsed between follow-up Weeks 4 and 12. The patient who discontinued prematurely due to depression relapsed during the first 4 weeks of follow up. All had *IL28B* non-CC genotype.

### Safety

Among all GT4 patients included in the analysis (n = 67), 60 (90%) experienced at least one treatment-emergent AE. The most commonly reported treatment-emergent AEs (reported in ≥20% of patients in either treatment group) were pruritus, neutropenia, asthenia, fatigue, decreased appetite, influenza-like illness and headache (Table 2). Two patients (3%) experienced a serious AE (acute sinusitis [n = 1] and phlebitis [n = 1]); both occurred in patients in the 24-week group and were considered not to be related to simeprevir (Table 2).

**Table 2:** On-treatment AEs Reported During the Study Period.

	Treatment duration		All patients
	12 weeks (n = 34)	24 weeks (n = 33)	All patients (N = 67)
**Any AE**	31 (91)	29 (88)	60 (90)
**Any serious AE**	0	2 (6)	2 (3)
**Any AE at least possibly related to simeprevir**	10 (29)	15 (45)	25 (37)
**Any AE at least possibly related to peg-IFN**	20 (59)	26 (79)	46 (69)
**Any AE at least possibly related to RBV**	17 (50)	19 (58)	36 (54)
**Any AE leading to permanent stop of all study drugs**	0	3 (9)	3 (4)
Worst Grade 3 or 4 AE*	6 (18)	14 (42)	20 (30)
**Most commonly reported AE**^†^			
**Pruritus**	9 (26)	7 (21)	16 (24)
**Neutropenia**	9 (26)	7 (21)	16 (24)
**Asthenia**	5 (15)	9 (27)	14 (21)
**Fatigue**	6 (18)	8 (24)	14 (21)
**Decreased appetite**	6 (18)	8 (24)	14 (21)
**Headache**	5 (15)	8 (24)	13 (19)
**Influenza-like illness**	3 (9)	9 (27)	12 (18)

AE, adverse event; peg-IFN, pegylated interferon; RBV, ribavirin.

*Three Grade 3 AEs were considered at least possibly related to simeprevir: asthenia (n = 2) and blood bilirubin increase (n = 1), all in the 24-week group. No Grade 4 AEs were considered at least possibly related to simeprevir.

^†^Occurring in ≥20% of patients in either treatment group.

Four patients discontinued at least one study treatment (PR ± simeprevir) because of AEs. All four were in the 24-week group. Only one (erythema and pruritus [n = 1]), was considered probably related to simeprevir (the patient discontinued all treatment during Weeks 10–11). The other three (depression [n = 1, discontinued all treatment at Week 8], pyrexia/oropharyngeal pain/headache/abdominal pain [n = 1, discontinued all treatment during Week 1], and erythema nodosum [n = 1, discontinued PR during Weeks 22–23]) were considered not to be related to simeprevir.

Similar proportions of patients in the 12- and 24-week groups (91% and 88% respectively; Chi-Squared p = NS) reported treatment-emergent AEs (all grades) and AEs of special interest. Overall, a higher proportion of patients experienced Grade 3/4 AEs in the 24-week group (42% [14/33]) than the 12-week group (18% [6/34]; Chi-Squared p = 0.027). However, of the Grade 3/4 AEs experienced by patients in the 24-week group, 11 had their onset in the first 12 weeks of treatment and four began during the PR-only phase. Of those patients who received at least one dose of PR in the PR-only phase, 18/27 (67%) experienced ≥1 new AE during Weeks 12–24 and 4/27 (15%) experienced a worst Grade 3/4 AE during this phase.

Most on-treatment Grade 3/4 AEs, across both groups, were not considered related to simeprevir (85% [17/20]). The three Grade 3/4 AEs considered at least possibly related to simeprevir were Grade 3 instances of asthenia [n = 2] and increased blood bilirubin [n = 1], all in the 24-week group. Grade 4 AEs were reported in three patients in the 24-week group (neutrophil count decrease [n = 3]) and none in the 12-week group; all Grade 4 AEs were considered not to be related to simeprevir.

Most on-treatment Grade 3/4 AEs were considered related to peg-IFN (90% [18/20]) and some were also considered related to RBV (30% [6/20]); however, all Grade 4 AEs were considered not to be related to RBV (S3 Table).

Regardless of AE severity or causality, the proportions of patients in whom asthenia (27% versus 15%), fatigue (24% versus 18%) and influenza-like illness (27% versus 9%) were observed were markedly higher in the 24-week arm than the 12-week arm.

Findings of the laboratory analysis of haemoglobin, neutrophils and precursors, platelets, and bilirubin are summarised in S2 Fig.

## Discussion

This study comprised 67 treatment-naïve patients with HCV GT4 mild-to-moderate (F0–F2) fibrosis, infected with various GT4 subtypes (GT4a, d or others). The overall SVR12 rate for patients who met the eligibility criteria for the 12-week group and discontinued both simeprevir and PR after 12 weeks of treatment was 97% (33/34). All patients who achieved SVR12 also achieved SVR24. The 34 patients who discontinued all treatment at Week 12 represented 51% of the total GT4 patient population.

The simeprevir plus PR regimen has previously displayed an acceptable safety and tolerability profile in patients with HCV GT1 [39] and GT4 [30]. No new safety signals were identified in this study. The same proportion of patients in the 12- and 24-week groups reported any AE. However, 67% of patients in the 24-week group who received PR experienced at least one new AE during the PR-only phase, and four patients experienced a new Grade 3/4 AE during this phase. Ending treatment after 12 weeks appeared to be associated with a reduction in overall treatment-associated morbidity versus the 24-week or longer regimen. Notably, the majority of Grade 3, and all Grade 4 AEs were considered unrelated to simeprevir, whereas 90% of Grade 3/4 AEs were considered related to peg-IFN.

Excellent results have been reported in broad HCV patient populations, including treatment-naïve and -experienced patients with HCV GT4, with a variety of IFN-free combination DAA regimens [11–23]. SVR rates exceeding 95% are now routinely reported in randomised clinical trials and in real-world studies after 12 weeks of treatment with such combinations. However, access to treatment continues to be limited by availability and cost, and the proportion of patients who can receive such regimens, if any, varies widely across countries and socioeconomic groups [25, 26, 40]. Voluntary licensing agreements have dramatically improved access to IFN-free regimens such as sofosbuvir plus ledipasvir in some countries, such as Egypt, but prices remain unaffordably high in many others [41] and are likely to remain so for some time in the absence of agreements [42]. Efforts are ongoing to improve access more widely (e.g. [43]); however as of 2016 there remains a sizable patient population who would clearly benefit from DAA therapy but are untreated or receiving clearly inferior regimens. For instance, in Greece–which has one of the highest rates of HCV prevalence in Europe with 14% of patients having GT4 –PR-only regimens remain widely used with estimated SVR of just 65% following 48 weeks of treatment [44].

In accordance with clinical practice recommendations, patients with advanced fibrosis or cirrhosis are typically prioritised for treatment [1, 26]. In many middle- and even high-income countries, patients with F0–F2 fibrosis continue to be ineligible for treatment [25, 26]. In Italy, the country with the highest HCV prevalence in Europe, simeprevir is the only DAA eligible for reimbursement for treatment of patients with F0–F2 fibrosis; other European countries do not reimburse DAA therapy at all for this patient population [45]. The cost-effectiveness of early treatment is well established [46–48], particularly when the risks of transmission and of complications such as hepatocellular carcinoma are taken into consideration [49–52]. Indeed, broadening access to treatment and eliminating the requirement for fibrosis staging to determine eligibility may be essential if future HCV incidence is to be significantly reduced [53].

Our findings are similar to those previously reported with an IFN-based single-DAA regimen in a similar, though smaller, treatment-naïve GT4 patient population [54]: an SVR rate of 96% (27/28) was achieved with 12 weeks of sofosbuvir plus PR. IFN-free single-DAA regimens of sofosbuvir plus RBV have been assessed in two studies of GT4 patients in Egypt and of Egyptian ancestry [55, 56], with overall SVR rates after 12 weeks of treatment of 77% and 68%, respectively (84% and 79% in treatment-naïve patients); SVR rates of ≥90% were achieved in both studies with 24 weeks of sofosbuvir plus PR.

HCV genotype 4 is particularly heterogeneous, with more described subtypes than for any other genotype [3]. In the aforementioned study of patients of Egyptian ancestry, SVR rates approaching 100% were achieved with 12 weeks of sofosbuvir plus PR in treatment-naïve patients with low (<600,000 IU/mL) baseline viral load and F0–F2 fibrosis [56]. In the RESTORE study of simeprevir plus PR [30], 94% (29/31) of treatment-naïve patients meeting response-guided therapy criteria achieved SVR12 with 12 weeks of simeprevir plus PR followed by 12 weeks of PR.

The criteria we used to determine eligibility to shorten total treatment duration of simeprevir plus PR treatment to 12 weeks were informed by previous observations of increased likelihood of SVR in patients displaying an early on-treatment virologic response [30, 54, 57, 58]. Based on previous findings of markedly greater treatment efficacy in HCV GT1-infected patients with *IL28B* CC genotype, more stringent eligibility criteria for 12-week treatment were applied to non-CC patients [32]. Virologic response was the only on-treatment factor used to determine eligibility for 12-week treatment. Among *IL28B* CC patients, 93% were eligible to stop therapy at Week 12 compared with 38% of *IL28B* non-CC, and all but one *IL28B* CC patient achieved SVR12. A low baseline viral load appeared to increase the probability of eligibility to stop all therapy at Week 12. No apparent effects of GT4 subtype, race, sex, geographical region or fibrosis stage on likelihood of eligibility for shortened treatment were observed and very high SVR rates were noted regardless of baseline characteristics. This is in accordance with previous findings with IFN-containing regimens, showing that early on-treatment response is associated with high SVR rates. Early on-treatment response continues to be more informative about eventual outcomes than baseline characteristics even as overall treatment duration is reduced to 12 weeks.

Examining negative baseline predictors of SVR in the 12-week group was not possible as treatment was successful in all but one patient in this group. However, it is noteworthy that patients infected with non-4a HCV genotypes treated for 12 weeks achieved 95% (19/20) SVR12 in this study. Genotype subtype has previously appeared to play a role in treatment outcomes for HCV GT4-infected patients treated with PR alone, with lower SVR rates observed in patients infected with a non-4a HCV genotype [59]. Although the numbers are small in this study, GT4 subtype did not appear to influence SVR rates in patients receiving 12 weeks of therapy, as only one such patient failed. This is particularly encouraging as the distribution of GT4 subtypes is subject to substantial geographical variation, with many regions of Africa, such as the Democratic Republic of the Congo and Gabon having a high prevalence of GT4 non-4a subtypes [8].

The substantial benefits of early treatment, in the context of ongoing limitation of access to DAAs in many countries in which HCV GT4 is prevalent, mean that an option of a short-duration, single-DAA, IFN-based regimen remains of considerable potential interest. PR treatment has previously been determined to be cost-effective even when non-drug costs associated with treatment, such as genotyping and clinical management, are taken into account [60]. However, the on-treatment impact on quality of life of protracted PR-only regimens, combined with their relatively low success rates even in patients with mild fibrosis, represent a considerable disincentive for patients to embark upon and adhere to HCV treatment [27].

The patients with HCV GT4 who participated in this study were recruited at sites in Europe and Saudi Arabia. The sample was well-balanced in terms of GT4 subtype and *IL28B* genotype and is considered to be representative of GT4 patients in Europe and the Middle East; subtype 4a and the *IL28B* CC genotype are relatively uncommon in sub-Saharan Africa [32], and the study population may, therefore, be less representative of sub-Saharan African patients. The sample size of 67 was similar to those of previously-reported DAA studies in HCV GT4, but we urge caution when interpreting the subgroup data because of the small numbers. Additionally, the eligibility criteria were defined such that patients who were not eligible for shortened treatment (e.g. because they discontinued treatment because of an AE or experienced virologic failure) were included by default in the 24-week group, potentially impacting the interpretation of the SVR data.

In conclusion, simeprevir plus PR for a total treatment duration of 12 weeks in treatment-naïve patients with early virologic response and mild to moderate liver fibrosis (METAVIR F0–F2) demonstrated an encouragingly high SVR rate.

## Supporting Information

S1 Checklist TREND Checklist. (DOC)

S1 Dataset Minimal Data Set. (ZIP)

S1 Fig Study Design Schematic. (DOCX)

S2 Fig Mean (±SE) On-treatment and EOT Laboratory Measures in Patients Receiving 12 and >12 Weeks’ Treatment: [A] Haemoglobin (g/L); [B] Neutrophils and Precursors (x10^9^/L); [C] Platelets (x10^9^/L); [D] Total Bilirubin (μmol/L).
(DOCX)

S1 Protocol Study Protocol. (PDF)

S1 Table HCV GT4 Subtype of All Patients According to a) Duration of Treatment; b) Country of Study Site.
(DOCX)

S2 Table Demographics and Disease Characteristics of Patients in the 24-Week Group who Experienced Viral Relapse (n = 3). (DOCX)

S3 Table Grade 3/4 AEs Considered At Least Possibly Related to a) Peg-IFN; b) RBV. (DOCX)

S1 Text List of Institutional Review Boards. (DOCX)

## References

[1] European Association for the Study of the Liver. EASL recommendations on treatment of hepatitis C. September 2016. Available at: http://www.easl.eu/medias/cpg/HCV2016/English-report.pdf [last accessed 22 November 2016]. 10.1016/j.jhep.2022.10.006 36464532

[2] Shiffman ML, Long AG, James A, Alexander P. My treatment approach to chronic hepatitis C virus. Mayo Clin Proc. 2014; 89:934–42. 10.1016/j.mayocp.2014.04.013 24867397

[3] Smith DB, Bukh J, Kuiken C, Muerhoff AS, Rice CM, Stapleton JT, et al Expanded classification of hepatitis C virus into 7 genotypes and 67 subtypes: updated criteria and genotype assignment web resource. Hepatology 2014; 59:318–27. 10.1002/hep.26744 24115039 PMC4063340

[4] Tapper EB, Afdhal NH. Is 3 the new 1: perspectives on virology, natural history and treatment for hepatitis C genotype 3. J Vir Hepat. 2013; 20:669–77. 10.1111/jvh.12168 24010641

[5] Khattab MA, Ferenci P, Hadziyannis SJ, Colombo M, Manns MP, Almasio PL, et al Management of hepatitis C virus genotype 4: recommendations of an international expert panel. J Hepatol. 2011; 54:1250–62. 10.1016/j.jhep.2010.11.016 21316497

[6] Gower E, Estes C, Blach S, Razavi-Shearer K, Razavi H. Global epidemiology and genotype distribution of the hepatitis C virus infection. J Hepatol. 2014; 61:S45–57. 10.1016/j.jhep.2014.07.027 25086286

[7] Messina JP, Humphreys I, Flaxman A, Brown A, Cooke GS, Pybus OG, et al Global distribution and prevalence of hepatitis C virus genotypes. Hepatology 2015; 61:77–87. 10.1002/hep.27259 25069599 PMC4303918

[8] Kamal SM. Hepatitis C virus genotype 4 therapy: progress and challenges. Liver Int. 2011; 31:S45–52. 10.1111/j.1478-3231.2010.02385.x 21205137

[9] Asselah T, De Muynck S, Broët P, Masliah-Planchon J, Blanluet M, Bièche I, et al IL28B polymorphism is associated with treatment response in chronic hepatitis C. J Hepatol. 2012; 56:527–32. 21951981 10.1016/j.jhep.2011.09.008

[10] Antaki N, Craxi A, Kamal S, Moucari R, Van der Merwe S, Haffar S, et al The neglected hepatitis C genotypes 4, 5 and 6: an international consensus report. Liver Int. 2010; 30:342–55. 10.1111/j.1478-3231.2009.02188.x 20015149

[11] Asselah T, Hézode C, Qaqish RB, ElKhashab M, Hassanein T, Papatheodoridis G, et al Ombitasvir, paritaprevir, and ritonavir plus ribavirin in adults with hepatitis C virus genotype 4 infection and cirrhosis (AGATE-I): a multicentre, phase 3, randomised open-label trial. Lancet Gastroenterol Hepatol 2016; 1:25–35. 28404108 10.1016/S2468-1253(16)30001-2

[12] Waked I, Shiha G, Qaqish RB, Esmat G, Yosry A, Hassany M, et al Ombitasvir, paritaprevir, and ritonavir plus ribavirin for chronic hepatitis C virus genotype 4 infection in Egyptian patients with or without compensated cirrhosis (AGATE-II): a multicentre, phase 3, partly randomised open-label trial. Lancet Gastroenterol Hepatol 2016; 1:36–44. 28404110 10.1016/S2468-1253(16)30002-4

[13] Hinrichsen H, Wedemeyer H, Christensen S, Sarrazin CM, Baumgarten A, Mauss S, et al Real-World Safety and Effectiveness of Ombitasvir/ Paritaprevir/R with Dasabuvir and/or Ribavirin in the German Hepatitis C Registry. J Hepatol 2016 64(Suppl 1):S159.

[14] Flisiak R, Janczewska E, Wawrzynowicz-Syczewska M, Jaroszewicz J, Zarębska-Michaluk D, Nazzal K, et al Real-world effectiveness and safety of ombitasvir/paritaprevir/ritonavir ± dasabuvir ± ribavirin in hepatitis C: AMBER study. Aliment Pharmacol Ther 2016; 44:946–56. 27611776 10.1111/apt.13790

[15] Hézode C, Asselah T, Reddy KR, Hassanein T, Berenguer M, Fleischer-Stepniewska K, et al Ombitasvir plus paritaprevir plus ritonavir with or without ribavirin in treatment-naïve and treatment-experienced patients with genotype 4 chronic hepatitis C virus infection (PEARL-I): a randomised, open-label trial. Lancet 2015; 385:2502–9. 10.1016/S0140-6736(15)60159-3 25837829

[16] El-Khayat HR, Fouad YM, Maher M, El-Amin H, Muhammed H. Efficacy and safety of sofosbuvir plus simeprevir in Egyptian patients with chronic hepatitis C: a real-world experience. Gut 2016; ePub ahead of print. 10.1136/gutjnl-2016-312012 27511197

[17] Kwo P, Gane E, Peng C-Y, Pearlman B, Vierling JM, Serfaty L, et al Effectiveness of Elbasvir and Grazoprevir Combination, With or Without Ribavirin, for Treatment-Experienced Patients with Chronic Hepatitis C Infection. Gastroenterology 2016; ePub ahead of print. 10.1053/j.gastro.2016.09.045 27720838

[18] Abergel A, Metivier S, Samuel D, Jiang D, Kersey K, Pang PS, et al Ledipasvir plus sofosbuvir for 12 weeks in patients with Hepatitis C Genotype 4 Infection. Hepatology 2016; 64:1049–56. 10.1002/hep.28706 27351341

[19] Feld JJ, Jacobson IM, Hézode C, Asselah T, Ruane PJ, Gruener N, et al Sofosbuvir and Velpatasvir for HCV Genotype 1, 2, 4, 5, and 6 Infection. N Engl J Med 2016; 373:2599–607. 10.1056/NEJMoa1512610 26571066

[20] Gane EJ, Kowdley KV, Pound D, Stedman CAM, Davis M, Etzkom K, et al Efficacy of Sofosbuvir, Velpatasvir, and GS-9857 in Patients With Hepatitis C Virus Genotype 2, 3, 4, or 6 Infections in an Open-label, Phase 2 Trial. Gastroenterology 2016; ePub ahead of print. 10.1053/j.gastro.2016.07.038 27486033

[21] Lawitz E, Reau N, Hinestrosa F, Rabinovitz M, Schiff E, Sheikh A, et al Efficacy of Sofosbuvir, Velpatasvir, and GS-9857 in Patients With Genotype 1 Hepatitis C Virus Infection in an Open-Label, Phase 2 Trial. Gastroenterology 2016; ePub ahead of print. 10.1053/j.gastro.2016.07.039 27486034

[22] Zhong M, Peng E, Huang N, Huang Q, Huq A, Lau M, et al Discovery of ravidasvir (PPI-668) as a potent pan-genotypic HCV NS5A inhibitor. Bioorg Med Chem Lett 2016; 26:4508–12. 10.1016/j.bmcl.2016.07.066 27506559

[23] Esmat G, El Raziky M, Elbaz T, Abouelkhair MM, Gamal El Deen H, Ashour MK, et al High virologic response rate in Egyptian HCV Genotype 4 patients treated with Ravidasvir (PPI-668) and sofosbuvir: results of a large multicenter Phase 3 registrational trial. AASLD 2015. Abstract LB-4.

[24] Hoofnagle JH, Sherker AH. Therapy for hepatitis C–the costs of success. N Engl J Med. 2014; 370:1552–3. 10.1056/NEJMe1401508 24725236

[25] MapCrowd. Dying at these prices: Generic HCV cure denied. July 2016. Available at: http://www.mapcrowd.org/public/pdf/EN_mapCrowd_Report2.pdf [Last accessed 22 November 2016].

[26] Omata M, Kanda T, Wei L, Yu M-L, Chuang W-L, Ibrahim A, et al APASL consensus statements and recommendation on treatment of hepatitis C. Hepatol Int 2016; 10:702–26. 10.1007/s12072-016-9717-6 27130427 PMC5003907

[27] Bernstein D, Kleinman L, Barker CM, Revicki DA, Green J, et al Relationship of health-related quality of life to treatment adherence and sustained response in chronic hepatitis C patients. Hepatology 2002; 35:704–8. 10.1053/jhep.2002.31311 11870387

[28] Jansen-Cilag. OLYSIO (simeprevir) tablets. Summary of product characteristics. 2014. http://www.ema.europa.eu/docs/en_GB/document_library/EPAR_-_Product_Information/human/002777/WC500167867.pdf [last accessed 22 November 2016].

[29] Janssen Therapeutics. OLYSIO (simeprevir) tablets. Prescribing information. 2014. http://www.accessdata.fda.gov/drugsatfda_docs/label/2013/205123s001lbl.pdf [last accessed 22 November 2016].

[30] Moreno C, Hezode C, Marcellin P, Bourgeois S, Francque S, Samuel D, Zoulim F, et al Efficacy and safety of simeprevir with peg-IFN/ribavirin in naïve or experienced patients infected with chronic HCV genotype 4. J Hepatol. 2015; 62:1047–55. 10.1016/j.jhep.2014.12.031 25596313

[31] Nelson DR, Poordad F, Feld JJ, Fried MW, Jacobson IM, Pockros PJ, et al High SVR rates (SVR4) for 12-week total telaprevir combination therapy in IL28B CC treatment-naïves and prior relapsers with G1 chronic hepatitis C: concise interim analysis. J Hepatol. 2013; 58:S362.

[32] Asselah T, Moreno C, Sarrazin C, Gschwantler M, Foster GR, Craxí A, et al An open label trial of 12-week simeprevir plus pegylated interferon/ribavirin in treatment-naïve patients with HCV genotype 1. PLoS ONE 2016 [in press]. 10.1371/journal.pone.0158526 PMC4948848 27428331

[33] American Association for the Study of Liver Diseases. HCV guidance: recommendations for testing, managing and treating hepatitis C. 2015. Available at: http://www.hcvguidelines.org/full-report-view [last accessed 22 November 2016].

[34] Bedossa P, Poynard T. An algorithm for the grading of activity in chronic hepatitis C. The METAVIR co-operative study group. Hepatology 1996; 24:289–93. 10.1002/hep.510240201 8690394

[35] Poynard T, Bedossa P, Opolon P. Natural history of liver fibrosis progression in patients with chronic hepatitis C. The OBSVIRC, METAVIR, CLINIVIR and DOSVIRC groups. Lancet 1997; 349:825–32. 9121257 10.1016/s0140-6736(96)07642-8

[36] Castera L. Non-invasive assessment of liver fibrosis in chronic hepatitis C. Hepatol Int. 2011; 5:625–34. 10.1007/s12072-010-9240-0 21484142 PMC3090550

[37] Ferraioli G, Tinelli C, Dal Bello B, Zicchetti M, Lissandrin R, Filice G, et al Performance of liver stiffness measurements by transient elastography in chronic hepatitis. World J Gastroenterol. 2013; 7:49–56. 10.3748/wjg.v19.i1.49 PMC3542745 23326162

[38] Huwart L, Sempoux C, Salameh N, Jamart J, Annet L, Sinkus R, et al. Liver fibrosis: noninvasive assessment with MR elastography versus aspartate aminotransferase-to-platelet ratio index. Radiology 2007; 245:458–66. 10.1148/radiol.2452061673 17940304

[39] Manns M, Fried MW, Zeuzem S, Jacobson IM, Forns X, Poordad F, et al Simeprevir with peginterferon/ribavirin for treatment of chronic hepatitis C virus genotype 1 infection: pooled safety analysis from phase IIb and IIII studies. J Viral Hep. 2015; 22:366–75. 10.1111/jvh.12346 25363449

[40] Edlin BR. Access to treatment for hepatitis C virus infection: time to put patients first. Lancet Infect Dis 2016; e196–201. 10.1016/S1473-3099(16)30005-6 27421993

[41] Andrieux-Meyer I, Cohn J, Affonso de Araújo ES, Hamid SS. Disparity in market prices for hepatitis C virus direct-acting drugs. Lancet Glob Health 2015; 3:e676–7. 10.1016/S2214-109X(15)00156-4 26475012

[42] Iyengar S, Tay-Teo K, Vogler S, Beyer P, Wiktor S, de Joncheere K, et al Prices, costs and affordability of new medicines for hepatitis C in 30 countries: an economic analysis. PLoS Medicine 2016; 13:e1002032 10.1371/journal.pmed.1002032 27243629 PMC4886962

[43] Drugs for Neglected Diseases Initiative (DNDI). An alternative research and development strategy to deliver affordable treatments for Hepatitis C patients. April 2016. Available at: http://www.dndi.org/wp-content/uploads/2016/04/AlternativeRDStrategyHepC.pdf [Last accessed 22 November 2016].

[44] Gountas I, Sypsa V, Papatheodoridis G, Souliotis K, Razavi H, Hatzakis A. Is elimination of HCV possible in a country with low diagnostic rate and moderate HCV prevalence? The case of Greece. J Gastroenterol Hepatol 2016; ePub ahead of print. 10.1111/jgh.13485 27403912

[45] Gentile I, Maraolo AE, Niola M, Graziano V, Borgia G, Paternoster M. Limiting the access to direct-acting antivirals against HCV: an ethical dilemma. Expert Rev Gastroenterol Hepatol 2016; ePub ahead of print. 10.1080/17474124.2016.1234375 27607920

[46] McCombs JS, Yuan Y, Shin J, Saab S. Economic burden associated with patients diagnosed with hepatitis C. Clin Ther. 2011; 33:1268–80. 10.1016/j.clinthera.2011.07.008 21840056

[47] Deuffic-Burban S, Obach D, Canva V, Pol S, Roudot-Thoraval F, Dhumeaux D, et al Cost-effectiveness and budget impact of interferon-free direct-acting antiviral-based regimens for hepatitis C treatment: the French case. J Vir Hepat 2016;23:767–79. 10.1111/jvh.12546 27144512

[48] Chahal HS, Marseille EA, Tice JA, Pearson SD, Ollendorf DA, Fox RK, et al Cost-effectiveness of early treatment of Hepatitis C Virus Genotype 1 by stage of liver fibrosis in a US treatment-naïve population. JAMA Intern Med 2016; 176:65–73. 10.1001/jamainternmed.2015.6011 26595724 PMC5144154

[49] Kim DD, Hutton DW, Raouf AA, Salama M, Hablas A, Seifeldin IA, et al Cost-effectiveness model for hepatitis C screening and treatment: implications for Egypt and other countries with high prevalence. Glob Public Health. 2015; 10:296–317. 10.1080/17441692.2014.984742 25469976 PMC4320005

[50] Yoshida H, Shiratori Y, Moriyama M, Arakawa Y, Ide T, Sata M, et al Interferon therapy reduces the risk for hepatocellular carcinoma: national surveillance program of cirrhotic and noncirrhotic patients with hepatitis C in Japan. Ann Intern Med 1999; 131:174–81. 10428733 10.7326/0003-4819-131-3-199908030-00003

[51] Leidner AJ, Chesson HW, Xu F, Ward JW, Spradling PR, Holmberg SD. Cost-effectiveness of hepatitis C treatment for patients in early stages of liver disease. Hepatology 2015; 61:1860–9. 10.1002/hep.27736 25677072 PMC5802336

[52] Dillon JF, Lazarus JV, Razavi HA. Urgent action to fight hepatitis C in people who inject drugs in Europe. Hepat Med. 2016 3 4;8:21–6. 30288305 10.1186/s41124-016-0011-y PMC5918492

[53] Woode ME, Abu-Zaineh M, Perriëns J, Renaud F, Wiktor S, Moatti J-P. Potential market size and impact of hepatitis C treatment in low- and middle-income countries. J Vir Hepat 2016; 23:522–34. 10.1111/jvh.12516 26924428

[54] Lawitz E, Mangia A, Wyles D, Rodriguez-Torres M, Hassanein T, Gordon SC, et al Sofosbuvir for previously untreated chronic hepatitis C infection. N Engl J Med 2013; 368:1878–87. 10.1056/NEJMoa1214853 23607594

[55] Doss W, Shiha G, Hassany M, Soliman R, Fouad R, Khairy M, et al Sofosbuvir plus ribavirin for treating Egyptian patients with hepatitis C genotype 4. J Hepatol. 2015; 63:581–5. 10.1016/j.jhep.2015.04.023 25937436

[56] Ruane PJ, Ain D, Stryker R, Meshrekey R, Soliman M, Wolfe PR, et al Sofosbuvir plus ribavirin in the treatment of chronic HCV genotype 4 infection in patients of Egyptian ancestry. J Hepatol 2015; 62:1040–6. 10.1016/j.jhep.2014.10.044 25450208

[57] Jacobson IM, Dore GJ, Foster GR, Fried MW, Radu M, Rafalsky VV, et al Simeprevir with pegylated interferon alfa 2a plus ribavirin in treatment-naïve patients with chronic hepatitis C virus genotype 1 infection (QUEST-1): a phase 3, randomised, double-blind, placebo-controlled trial. Lancet 2014; 384:403–13. 10.1016/S0140-6736(14)60494-3 24907225

[58] Manns M, Marcellin P, Poordad F, de Araujo ES, Buti M, Horsmans Y, et al Simeprevir with pegylated interferon alfa 2a or 2b plus ribavirin in treatment-naïve patients with chronic hepatitis C virus genotype 1 infection (QUEST-2): a randomised, double-blind, placebo-controlled phase 3 trial. Lancet 2014; 384:414–26. 10.1016/S0140-6736(14)60538-9 24907224

[59] Al-Ashgar HI, Khan MQ, Helmy A, Al-Thawadi S, Al-Ahdal MN, Khalaf N, et al Relationship of interferon-γ-inducible protein-10 kDa with viral response in patients with various heterogeneities of hepatitis C virus genotype-4. Eur J Gastroenterol Hepatol. 2013; 25:404–10. 10.1097/MEG.0b013e32835bc2cf 23470264

[60] Buti M, Medina M, Casado MA, Wong JB, Fosbrook L, Esteban R. A cost-effectiveness analysis of peginterferon alfa-2b plus ribavirin for the treatment of naive patients with chronic hepatitis C. Aliment Pharmacol Ther 2003; 17:687–94. 12641518 10.1046/j.1365-2036.2003.01453.x

