# Supplementary material for: Efficacy of a 12-Week Simeprevir Plus Peginterferon/Ribavirin (PR) Regimen in Treatment-Naïve Patients with Hepatitis C Virus (HCV) Genotype 4 (GT4) Infection and Mild-To-Moderate Fibrosis Displaying Early On-Treatment Virologic Response
Source: PLoS One. 2017 Jan 5;12(1):e0168713. doi: 10.1371/journal.pone.0168713 (PMC5215882; doi:10.1371/journal.pone.0168713)
Supplement: S1 Dataset — (ZIP) [file pone.0168713.s002.zip › TSFAE12TDG4.rtf]

TSFAE12TDG4:	Number and percentage of GT4 Subjects with Adverse Events at Least Possibly Related to Pegylated Interferona-2a by Worst WHO Toxicity Grade - Intent-to-treat (Study TMC435HPC3014)	
	Simeprevir
12 Wks
150 mg
PR 12/24 	
	SMV + PR 	Ent Trt 	PR Only 	Follow-Up 	Overall 	
Analysis set: Intent-to-treat	67	67	30	66	67	
Any Grade 1 AE	17 (25.4%)	17 (25.4%)	6 (20.0%)	0	17 (25.4%)	
General disorders and administration site conditions	23 (34.3%)	24 (35.8%)	5 (16.7%)	0	24 (35.8%)	
Fatigue	9 (13.4%)	9 (13.4%)	0	0	9 (13.4%)	
Influenza like illness	9 (13.4%)	9 (13.4%)	1 (3.3%)	0	9 (13.4%)	
Asthenia	8 (11.9%)	8 (11.9%)	0	0	8 (11.9%)	
Pyrexia	5 (7.5%)	6 (9.0%)	1 (3.3%)	0	6 (9.0%)	
Injection site erythema	1 (1.5%)	3 (4.5%)	2 (6.7%)	0	3 (4.5%)	
Irritability	1 (1.5%)	2 (3.0%)	1 (3.3%)	0	2 (3.0%)	
Application site alopecia	0	1 (1.5%)	1 (3.3%)	0	1 (1.5%)	
Discomfort	1 (1.5%)	1 (1.5%)	0	0	1 (1.5%)	
Injection site rash	1 (1.5%)	1 (1.5%)	0	0	1 (1.5%)	
Mucosal dryness	1 (1.5%)	1 (1.5%)	0	0	1 (1.5%)	
Metabolism and nutrition disorders	14 (20.9%)	14 (20.9%)	0	0	14 (20.9%)	
Decreased appetite	13 (19.4%)	13 (19.4%)	0	0	13 (19.4%)	
Hyperinsulinaemia	1 (1.5%)	1 (1.5%)	0	0	1 (1.5%)	
Hypokalaemia	1 (1.5%)	1 (1.5%)	0	0	1 (1.5%)	
Psychiatric disorders	12 (17.9%)	13 (19.4%)	1 (3.3%)	0	13 (19.4%)	
Depression	4 (6.0%)	5 (7.5%)	1 (3.3%)	0	5 (7.5%)	
Insomnia	5 (7.5%)	5 (7.5%)	0	0	5 (7.5%)	
Anxiety	1 (1.5%)	1 (1.5%)	0	0	1 (1.5%)	
Anxiety disorder	1 (1.5%)	1 (1.5%)	0	0	1 (1.5%)	
Depressed mood	1 (1.5%)	1 (1.5%)	0	0	1 (1.5%)	
Libido decreased	1 (1.5%)	1 (1.5%)	0	0	1 (1.5%)	
Nervousness	1 (1.5%)	1 (1.5%)	0	0	1 (1.5%)	
Sleep disorder	1 (1.5%)	1 (1.5%)	0	0	1 (1.5%)	
Gastrointestinal disorders	11 (16.4%)	11 (16.4%)	1 (3.3%)	0	11 (16.4%)	
Diarrhoea	5 (7.5%)	5 (7.5%)	1 (3.3%)	0	5 (7.5%)	
Vomiting	4 (6.0%)	4 (6.0%)	0	0	4 (6.0%)	
Dry mouth	3 (4.5%)	3 (4.5%)	0	0	3 (4.5%)	
Nausea	3 (4.5%)	3 (4.5%)	0	0	3 (4.5%)	
Dyspepsia	2 (3.0%)	2 (3.0%)	0	0	2 (3.0%)	
Abdominal distension	1 (1.5%)	1 (1.5%)	0	0	1 (1.5%)	
Abdominal pain upper	1 (1.5%)	1 (1.5%)	0	0	1 (1.5%)	
Constipation	1 (1.5%)	1 (1.5%)	0	0	1 (1.5%)	
Haemorrhoids	1 (1.5%)	1 (1.5%)	0	0	1 (1.5%)	
Skin and subcutaneous tissue disorders	7 (10.4%)	11 (16.4%)	5 (16.7%)	0	11 (16.4%)	
Pruritus	4 (6.0%)	5 (7.5%)	2 (6.7%)	0	5 (7.5%)	
Rash	3 (4.5%)	5 (7.5%)	3 (10.0%)	0	5 (7.5%)	
Erythema	2 (3.0%)	3 (4.5%)	1 (3.3%)	0	3 (4.5%)	
Erythema nodosum	0	1 (1.5%)	1 (3.3%)	0	1 (1.5%)	
Hyperhidrosis	0	1 (1.5%)	1 (3.3%)	0	1 (1.5%)	
Nervous system disorders	7 (10.4%)	8 (11.9%)	1 (3.3%)	0	8 (11.9%)	
Headache	5 (7.5%)	6 (9.0%)	1 (3.3%)	0	6 (9.0%)	
Dizziness	2 (3.0%)	2 (3.0%)	0	0	2 (3.0%)	
Memory impairment	2 (3.0%)	2 (3.0%)	0	0	2 (3.0%)	
Disturbance in attention	1 (1.5%)	1 (1.5%)	0	0	1 (1.5%)	
Paraesthesia	1 (1.5%)	1 (1.5%)	0	0	1 (1.5%)	
Blood and lymphatic system disorders	4 (6.0%)	6 (9.0%)	1 (3.3%)	0	6 (9.0%)	
Neutropenia	4 (6.0%)	5 (7.5%)	1 (3.3%)	0	5 (7.5%)	
Leukopenia	2 (3.0%)	2 (3.0%)	0	0	2 (3.0%)	
Thrombocytopenia	2 (3.0%)	2 (3.0%)	0	0	2 (3.0%)	
Anaemia	0	1 (1.5%)	0	0	1 (1.5%)	
Musculoskeletal and connective tissue disorders	5 (7.5%)	5 (7.5%)	0	0	5 (7.5%)	
Myalgia	2 (3.0%)	2 (3.0%)	0	0	2 (3.0%)	
Arthralgia	1 (1.5%)	1 (1.5%)	0	0	1 (1.5%)	
Back pain	1 (1.5%)	1 (1.5%)	0	0	1 (1.5%)	
Musculoskeletal stiffness	1 (1.5%)	1 (1.5%)	0	0	1 (1.5%)	
Ear and labyrinth disorders	4 (6.0%)	4 (6.0%)	0	0	4 (6.0%)	
Tinnitus	2 (3.0%)	2 (3.0%)	0	0	2 (3.0%)	
Vertigo	2 (3.0%)	2 (3.0%)	0	0	2 (3.0%)	
Respiratory, thoracic and mediastinal disorders	2 (3.0%)	3 (4.5%)	1 (3.3%)	0	3 (4.5%)	
Dyspnoea	2 (3.0%)	3 (4.5%)	1 (3.3%)	0	3 (4.5%)	
Cardiac disorders	2 (3.0%)	2 (3.0%)	0	0	2 (3.0%)	
Palpitations	2 (3.0%)	2 (3.0%)	0	0	2 (3.0%)	
Eye disorders	1 (1.5%)	2 (3.0%)	1 (3.3%)	0	2 (3.0%)	
Eye disorder	1 (1.5%)	1 (1.5%)	0	0	1 (1.5%)	
Eye pain	1 (1.5%)	1 (1.5%)	0	0	1 (1.5%)	
Vision blurred	0	1 (1.5%)	1 (3.3%)	0	1 (1.5%)	
Investigations	2 (3.0%)	2 (3.0%)	0	0	2 (3.0%)	
Blood glucose increased	1 (1.5%)	1 (1.5%)	0	0	1 (1.5%)	
Blood lactate dehydrogenase increased	0	1 (1.5%)	1 (3.3%)	0	1 (1.5%)	
Haemoglobin decreased	1 (1.5%)	1 (1.5%)	0	0	1 (1.5%)	
Neutrophil count decreased	1 (1.5%)	1 (1.5%)	0	0	1 (1.5%)	
Any Grade 2 AE	13 (19.4%)	11 (16.4%)	1 (3.3%)	0	11 (16.4%)	
General disorders and administration site conditions	7 (10.4%)	7 (10.4%)	1 (3.3%)	0	7 (10.4%)	
Fatigue	3 (4.5%)	3 (4.5%)	0	0	3 (4.5%)	
Influenza like illness	2 (3.0%)	3 (4.5%)	1 (3.3%)	0	3 (4.5%)	
Asthenia	2 (3.0%)	2 (3.0%)	0	0	2 (3.0%)	
Chest pain	1 (1.5%)	1 (1.5%)	0	0	1 (1.5%)	
Pyrexia	1 (1.5%)	1 (1.5%)	0	0	1 (1.5%)	
Blood and lymphatic system disorders	6 (9.0%)	6 (9.0%)	1 (3.3%)	0	6 (9.0%)	
Anaemia	3 (4.5%)	3 (4.5%)	0	0	3 (4.5%)	
Neutropenia	2 (3.0%)	3 (4.5%)	1 (3.3%)	0	3 (4.5%)	
Thrombocytopenia	1 (1.5%)	1 (1.5%)	0	0	1 (1.5%)	
Psychiatric disorders	3 (4.5%)	4 (6.0%)	1 (3.3%)	0	4 (6.0%)	
Depressed mood	2 (3.0%)	2 (3.0%)	0	0	2 (3.0%)	
Sleep disorder	1 (1.5%)	2 (3.0%)	1 (3.3%)	0	2 (3.0%)	
Depression	1 (1.5%)	1 (1.5%)	0	0	1 (1.5%)	
Skin and subcutaneous tissue disorders	3 (4.5%)	4 (6.0%)	1 (3.3%)	0	4 (6.0%)	
Pruritus	2 (3.0%)	2 (3.0%)	0	0	2 (3.0%)	
Dry skin	1 (1.5%)	1 (1.5%)	0	0	1 (1.5%)	
Onychoclasis	0	1 (1.5%)	1 (3.3%)	0	1 (1.5%)	
Investigations	3 (4.5%)	2 (3.0%)	0	0	2 (3.0%)	
Aspartate aminotransferase increased	0	1 (1.5%)	1 (3.3%)	0	1 (1.5%)	
Blood bilirubin increased	1 (1.5%)	1 (1.5%)	0	0	1 (1.5%)	
Haemoglobin decreased	1 (1.5%)	1 (1.5%)	0	0	1 (1.5%)	
Platelet count decreased	1 (1.5%)	1 (1.5%)	0	0	1 (1.5%)	
Weight decreased	1 (1.5%)	1 (1.5%)	0	0	1 (1.5%)	
Nervous system disorders	2 (3.0%)	2 (3.0%)	0	0	2 (3.0%)	
Dizziness	1 (1.5%)	1 (1.5%)	0	0	1 (1.5%)	
Headache	1 (1.5%)	1 (1.5%)	0	0	1 (1.5%)	
Gastrointestinal disorders	1 (1.5%)	1 (1.5%)	0	0	1 (1.5%)	
Mouth ulceration	1 (1.5%)	1 (1.5%)	0	0	1 (1.5%)	
Infections and infestations	1 (1.5%)	1 (1.5%)	0	0	1 (1.5%)	
Gingival infection	1 (1.5%)	1 (1.5%)	0	0	1 (1.5%)	
Metabolism and nutrition disorders	0	1 (1.5%)	1 (3.3%)	0	1 (1.5%)	
Decreased appetite	0	1 (1.5%)	1 (3.3%)	0	1 (1.5%)	
Respiratory, thoracic and mediastinal disorders	1 (1.5%)	1 (1.5%)	0	0	1 (1.5%)	
Dyspnoea	1 (1.5%)	1 (1.5%)	0	0	1 (1.5%)	
Vascular disorders	0	1 (1.5%)	1 (3.3%)	0	1 (1.5%)	
Pallor	0	1 (1.5%)	1 (3.3%)	0	1 (1.5%)	
Any Grade 3 AE	13 (19.4%)	15 (22.4%)	3 (10.0%)	0	15 (22.4%)	
Blood and lymphatic system disorders	7 (10.4%)	8 (11.9%)	1 (3.3%)	0	8 (11.9%)	
Neutropenia	7 (10.4%)	8 (11.9%)	1 (3.3%)	0	8 (11.9%)	
Investigations	3 (4.5%)	4 (6.0%)	2 (6.7%)	0	4 (6.0%)	
Neutrophil count decreased	3 (4.5%)	3 (4.5%)	1 (3.3%)	0	3 (4.5%)	
Alanine aminotransferase increased	0	1 (1.5%)	1 (3.3%)	0	1 (1.5%)	
General disorders and administration site conditions	2 (3.0%)	2 (3.0%)	0	0	2 (3.0%)	
Asthenia	2 (3.0%)	2 (3.0%)	0	0	2 (3.0%)	
Nervous system disorders	1 (1.5%)	1 (1.5%)	0	0	1 (1.5%)	
Headache	1 (1.5%)	1 (1.5%)	0	0	1 (1.5%)	
Psychiatric disorders	1 (1.5%)	1 (1.5%)	0	0	1 (1.5%)	
Depression	1 (1.5%)	1 (1.5%)	0	0	1 (1.5%)	
Any Grade 4 AE	2 (3.0%)	3 (4.5%)	1 (3.3%)	0	3 (4.5%)	
Investigations	2 (3.0%)	3 (4.5%)	1 (3.3%)	0	3 (4.5%)	
Neutrophil count decreased	2 (3.0%)	3 (4.5%)	1 (3.3%)	0	3 (4.5%)	
Any Grade 3-4 AE	15 (22.4%)	18 (26.9%)	4 (13.3%)	0	18 (26.9%)	
Blood and lymphatic system disorders	7 (10.4%)	8 (11.9%)	1 (3.3%)	0	8 (11.9%)	
Neutropenia	7 (10.4%)	8 (11.9%)	1 (3.3%)	0	8 (11.9%)	
Investigations	5 (7.5%)	7 (10.4%)	3 (10.0%)	0	7 (10.4%)	
Neutrophil count decreased	5 (7.5%)	6 (9.0%)	2 (6.7%)	0	6 (9.0%)	
Alanine aminotransferase increased	0	1 (1.5%)	1 (3.3%)	0	1 (1.5%)	
General disorders and administration site conditions	2 (3.0%)	2 (3.0%)	0	0	2 (3.0%)	
Asthenia	2 (3.0%)	2 (3.0%)	0	0	2 (3.0%)	
Nervous system disorders	1 (1.5%)	1 (1.5%)	0	0	1 (1.5%)	
Headache	1 (1.5%)	1 (1.5%)	0	0	1 (1.5%)	
Psychiatric disorders	1 (1.5%)	1 (1.5%)	0	0	1 (1.5%)	
Depression	1 (1.5%)	1 (1.5%)	0	0	1 (1.5%)	
	
[TSFAE12TDG4.RTF] [TMC435\HPC3014\DBR_FINAL_ANALYSIS\RE_FINAL_ANALYSIS\PDEV\TEMPFILE.SAS] 21OCT2016, 16:35	
