# Supplementary material for: Efficacy of a 12-Week Simeprevir Plus Peginterferon/Ribavirin (PR) Regimen in Treatment-Naïve Patients with Hepatitis C Virus (HCV) Genotype 4 (GT4) Infection and Mild-To-Moderate Fibrosis Displaying Early On-Treatment Virologic Response
Source: PLoS One. 2017 Jan 5;12(1):e0168713. doi: 10.1371/journal.pone.0168713 (PMC5215882; doi:10.1371/journal.pone.0168713)
Supplement: S1 Dataset — (ZIP) [file pone.0168713.s002.zip › tsfae15tdg4all.rtf]

TSFAE15TDG4ALL:	Number (pcnt) of Genotype 4 Subjects with Adverse Events of Special/Clinical Interest by Preferred Term, Intent-to-treat, Study TMC435HPC3014, All Subjects	
	Simeprevir
12 Wks
150 mg
PR 12/24 	
	SMV + PR 	Ent Trt 	PR Only 	Follow-Up 	Overall 	
Analysis set: intent-to-treat	67	67	30	66	67	
Any AE	59 (88.1%)	60 (89.6%)	20 (66.7%)	12 (18.2%)	61 (91.0%)	
NEUTRO	19 (28.4%)	23 (34.3%)	5 (16.7%)	0	23 (34.3%)	
Neutropenia	13 (19.4%)	16 (23.9%)	3 (10.0%)	0	16 (23.9%)	
Neutrophil count decreased	6 (9.0%)	7 (10.4%)	2 (6.7%)	0	7 (10.4%)	
RASH (ANY TYPE)	13 (19.4%)	17 (25.4%)	4 (13.3%)	0	17 (25.4%)	
Rash	8 (11.9%)	11 (16.4%)	3 (10.0%)	0	11 (16.4%)	
Erythema	5 (7.5%)	6 (9.0%)	1 (3.3%)	0	6 (9.0%)	
PRURITUS (ANY TYPE)	15 (22.4%)	16 (23.9%)	3 (10.0%)	0	16 (23.9%)	
Pruritus	15 (22.4%)	16 (23.9%)	3 (10.0%)	0	16 (23.9%)	
UPPER GI	11 (16.4%)	12 (17.9%)	2 (6.7%)	1 (1.5%)	12 (17.9%)	
Vomiting	6 (9.0%)	7 (10.4%)	1 (3.3%)	1 (1.5%)	7 (10.4%)	
Abdominal pain upper	3 (4.5%)	4 (6.0%)	1 (3.3%)	0	4 (6.0%)	
Dyspepsia	3 (4.5%)	3 (4.5%)	0	0	3 (4.5%)	
Nausea	3 (4.5%)	3 (4.5%)	0	0	3 (4.5%)	
ANEMIA	8 (11.9%)	9 (13.4%)	0	0	9 (13.4%)	
Anaemia	6 (9.0%)	7 (10.4%)	0	0	7 (10.4%)	
Haemoglobin decreased	2 (3.0%)	2 (3.0%)	0	0	2 (3.0%)	
DYSPNEA	6 (9.0%)	7 (10.4%)	1 (3.3%)	0	7 (10.4%)	
Dyspnoea	5 (7.5%)	6 (9.0%)	1 (3.3%)	0	6 (9.0%)	
Dyspnoea exertional	1 (1.5%)	1 (1.5%)	0	0	1 (1.5%)	
INCREASED BILIRUBIN	4 (6.0%)	4 (6.0%)	1 (3.3%)	0	4 (6.0%)	
Blood bilirubin increased	3 (4.5%)	3 (4.5%)	1 (3.3%)	0	3 (4.5%)	
Hyperbilirubinaemia	1 (1.5%)	1 (1.5%)	0	0	1 (1.5%)	
Rash FDA						
Y	13 (19.4%)	19 (28.4%)	6 (20.0%)	0	19 (28.4%)	
Rash	8 (11.9%)	11 (16.4%)	3 (10.0%)	0	11 (16.4%)	
Erythema	5 (7.5%)	6 (9.0%)	1 (3.3%)	0	6 (9.0%)	
Eczema	0	2 (3.0%)	2 (6.7%)	0	2 (3.0%)	
	
[TSFAE15TDG4ALL.RTF] [TMC435\HPC3014\DBR_FINAL_ANALYSIS\RE_FINAL_ANALYSIS\PROD\TSFAE15TDG4ALL.SAS] 02NOV2015, 11:20	
