# Supplementary material for: Efficacy of a 12-Week Simeprevir Plus Peginterferon/Ribavirin (PR) Regimen in Treatment-Naïve Patients with Hepatitis C Virus (HCV) Genotype 4 (GT4) Infection and Mild-To-Moderate Fibrosis Displaying Early On-Treatment Virologic Response
Source: PLoS One. 2017 Jan 5;12(1):e0168713. doi: 10.1371/journal.pone.0168713 (PMC5215882; doi:10.1371/journal.pone.0168713)
Supplement: S1 Dataset — (ZIP) [file pone.0168713.s002.zip › tsfae15tdg4gt12.rtf]

TSFAE15TDG4GT12:	Number (pcnt) of Genotype 4 Subjects with Adverse Events of Special/Clinical Interest by Preferred Term, Intent-to-treat, Study TMC435HPC3014, Trt Dur gt 12 Wks 	
	Simeprevir
12 Wks
150 mg
PR 12/24 	
	SMV + PR 	Ent Trt 	PR Only 	Follow-Up 	Overall 	
Analysis set: intent-to-treat	33	33	27	32	33	
Any AE	29 (87.9%)	29 (87.9%)	18 (66.7%)	7 (21.9%)	30 (90.9%)	
NEUTRO	8 (24.2%)	12 (36.4%)	5 (18.5%)	0	12 (36.4%)	
Neutropenia	4 (12.1%)	7 (21.2%)	3 (11.1%)	0	7 (21.2%)	
Neutrophil count decreased	4 (12.1%)	5 (15.2%)	2 (7.4%)	0	5 (15.2%)	
RASH (ANY TYPE)	6 (18.2%)	9 (27.3%)	4 (14.8%)	0	9 (27.3%)	
Rash	4 (12.1%)	6 (18.2%)	3 (11.1%)	0	6 (18.2%)	
Erythema	2 (6.1%)	3 (9.1%)	1 (3.7%)	0	3 (9.1%)	
DYSPNEA	6 (18.2%)	7 (21.2%)	1 (3.7%)	0	7 (21.2%)	
Dyspnoea	5 (15.2%)	6 (18.2%)	1 (3.7%)	0	6 (18.2%)	
Dyspnoea exertional	1 (3.0%)	1 (3.0%)	0	0	1 (3.0%)	
PRURITUS (ANY TYPE)	6 (18.2%)	7 (21.2%)	3 (11.1%)	0	7 (21.2%)	
Pruritus	6 (18.2%)	7 (21.2%)	3 (11.1%)	0	7 (21.2%)	
UPPER GI	5 (15.2%)	6 (18.2%)	2 (7.4%)	1 (3.1%)	6 (18.2%)	
Vomiting	4 (12.1%)	5 (15.2%)	1 (3.7%)	1 (3.1%)	5 (15.2%)	
Abdominal pain upper	1 (3.0%)	2 (6.1%)	1 (3.7%)	0	2 (6.1%)	
Dyspepsia	2 (6.1%)	2 (6.1%)	0	0	2 (6.1%)	
Nausea	2 (6.1%)	2 (6.1%)	0	0	2 (6.1%)	
ANEMIA	5 (15.2%)	5 (15.2%)	0	0	5 (15.2%)	
Anaemia	4 (12.1%)	4 (12.1%)	0	0	4 (12.1%)	
Haemoglobin decreased	1 (3.0%)	1 (3.0%)	0	0	1 (3.0%)	
INCREASED BILIRUBIN	3 (9.1%)	3 (9.1%)	1 (3.7%)	0	3 (9.1%)	
Blood bilirubin increased	3 (9.1%)	3 (9.1%)	1 (3.7%)	0	3 (9.1%)	
Rash FDA						
Y	6 (18.2%)	10 (30.3%)	5 (18.5%)	0	10 (30.3%)	
Rash	4 (12.1%)	6 (18.2%)	3 (11.1%)	0	6 (18.2%)	
Erythema	2 (6.1%)	3 (9.1%)	1 (3.7%)	0	3 (9.1%)	
Eczema	0	1 (3.0%)	1 (3.7%)	0	1 (3.0%)	
	
[TSFAE15TDG4GT12.RTF] [TMC435\HPC3014\DBR_FINAL_ANALYSIS\RE_FINAL_ANALYSIS\PROD\TSFAE15TDG4GT12.SAS] 02NOV2015, 11:20	
