# Supplementary material for: Efficacy of a 12-Week Simeprevir Plus Peginterferon/Ribavirin (PR) Regimen in Treatment-Naïve Patients with Hepatitis C Virus (HCV) Genotype 4 (GT4) Infection and Mild-To-Moderate Fibrosis Displaying Early On-Treatment Virologic Response
Source: PLoS One. 2017 Jan 5;12(1):e0168713. doi: 10.1371/journal.pone.0168713 (PMC5215882; doi:10.1371/journal.pone.0168713)
Supplement: S1 Dataset — (ZIP) [file pone.0168713.s002.zip › tsfae15tdg412.rtf]

TSFAE15TDG412:	Number (pcnt) of Genotype 4 Subjects with Adverse Events of Special/Clinical Interest by Preferred Term, Intent-to-treat, Study TMC435HPC3014, Trt Dur 12 Wks 	
	Simeprevir
12 Wks
150 mg
PR 12/24 	
	SMV + PR 	Ent Trt 	PR Only 	Follow-Up 	Overall 	
Analysis set: intent-to-treat	34	34	3	34	34	
Any AE	30 (88.2%)	31 (91.2%)	2 (66.7%)	5 (14.7%)	31 (91.2%)	
NEUTRO	11 (32.4%)	11 (32.4%)	0	0	11 (32.4%)	
Neutropenia	9 (26.5%)	9 (26.5%)	0	0	9 (26.5%)	
Neutrophil count decreased	2 (5.9%)	2 (5.9%)	0	0	2 (5.9%)	
PRURITUS (ANY TYPE)	9 (26.5%)	9 (26.5%)	0	0	9 (26.5%)	
Pruritus	9 (26.5%)	9 (26.5%)	0	0	9 (26.5%)	
RASH (ANY TYPE)	7 (20.6%)	8 (23.5%)	0	0	8 (23.5%)	
Rash	4 (11.8%)	5 (14.7%)	0	0	5 (14.7%)	
Erythema	3 (8.8%)	3 (8.8%)	0	0	3 (8.8%)	
UPPER GI	6 (17.6%)	6 (17.6%)	0	0	6 (17.6%)	
Abdominal pain upper	2 (5.9%)	2 (5.9%)	0	0	2 (5.9%)	
Vomiting	2 (5.9%)	2 (5.9%)	0	0	2 (5.9%)	
Dyspepsia	1 (2.9%)	1 (2.9%)	0	0	1 (2.9%)	
Nausea	1 (2.9%)	1 (2.9%)	0	0	1 (2.9%)	
ANEMIA	3 (8.8%)	4 (11.8%)	0	0	4 (11.8%)	
Anaemia	2 (5.9%)	3 (8.8%)	0	0	3 (8.8%)	
Haemoglobin decreased	1 (2.9%)	1 (2.9%)	0	0	1 (2.9%)	
INCREASED BILIRUBIN	1 (2.9%)	1 (2.9%)	0	0	1 (2.9%)	
Hyperbilirubinaemia	1 (2.9%)	1 (2.9%)	0	0	1 (2.9%)	
Rash FDA						
Y	7 (20.6%)	9 (26.5%)	1 (33.3%)	0	9 (26.5%)	
Rash	4 (11.8%)	5 (14.7%)	0	0	5 (14.7%)	
Erythema	3 (8.8%)	3 (8.8%)	0	0	3 (8.8%)	
Eczema	0	1 (2.9%)	1 (33.3%)	0	1 (2.9%)	
	
[TSFAE15TDG412.RTF] [TMC435\HPC3014\DBR_FINAL_ANALYSIS\RE_FINAL_ANALYSIS\PROD\TSFAE15TDG412.SAS] 02NOV2015, 11:10	
