# Supplementary material for: Efficacy of a 12-Week Simeprevir Plus Peginterferon/Ribavirin (PR) Regimen in Treatment-Naïve Patients with Hepatitis C Virus (HCV) Genotype 4 (GT4) Infection and Mild-To-Moderate Fibrosis Displaying Early On-Treatment Virologic Response
Source: PLoS One. 2017 Jan 5;12(1):e0168713. doi: 10.1371/journal.pone.0168713 (PMC5215882; doi:10.1371/journal.pone.0168713)
Supplement: S1 Dataset — (ZIP) [file pone.0168713.s002.zip › TSFLAB01-GT.rtf]

TSFLAB01-GT:	Actual Laboratory Parameters values by Analysis Timepoint (Entire Treatment Phase); Intent-to-treat (Study TMC435HPC3014)	
	Simeprevir
12 Wks
150 mg
PR 12/24 	
	 Genotype 4 		
	 12 Wks
(N=34) 	 >12 Wks
(N=33) 	 All subjects
(N=67) 				
Hemoglobin (g/L)							
Baseline							
N	34	33	67				
Mean	150.47	151.48	150.97				
Std. Err.	2.372	2.691	1.778				
Std. Dev.	13.833	15.458	14.553				
95% C.I.	(145.644; 155.297)	(146.004; 156.966)	(147.420; 154.520)				
Minimum	120.0	121.0	120.0				
First quartile	141.00	137.00	140.00				
Median	150.00	156.00	153.00				
Third quartile	160.00	162.00	162.00				
Maximum	174.0	180.0	180.0				
Week 01							
N	34	31	65				
Mean	148.88	150.00	149.42				
Std. Err.	2.231	2.306	1.592				
Std. Dev.	13.008	12.837	12.838				
95% C.I.	(144.344; 153.421)	(145.291; 154.709)	(146.234; 152.596)				
Minimum	120.0	122.0	120.0				
First quartile	139.00	141.00	140.00				
Median	148.00	154.00	150.00				
Third quartile	157.00	159.00	158.00				
Maximum	173.0	174.0	174.0				
Week 02							
N	31	30	61				
Mean	138.94	140.97	139.93				
Std. Err.	2.232	3.067	1.876				
Std. Dev.	12.428	16.800	14.652				
95% C.I.	(134.377; 143.494)	(134.693; 147.240)	(136.182; 143.687)				
Minimum	107.0	112.0	107.0				
First quartile	130.00	127.00	130.00				
Median	141.00	139.50	140.00				
Third quartile	147.00	154.00	152.00				
Maximum	160.0	174.0	174.0				
Week 04							
N	34	32	66				
Mean	130.50	129.16	129.85				
Std. Err.	2.201	3.079	1.862				
Std. Dev.	12.835	17.419	15.126				
95% C.I.	(126.022; 134.978)	(122.876; 135.437)	(126.130; 133.567)				
Minimum	105.0	87.0	87.0				
First quartile	123.00	120.50	121.00				
Median	131.00	129.00	130.50				
Third quartile	137.00	144.00	138.00				
Maximum	160.0	157.0	160.0				
Week 08							
N	33	30	63				
Mean	123.58	125.47	124.48				
Std. Err.	2.051	3.012	1.781				
Std. Dev.	11.785	16.498	14.139				
95% C.I.	(119.397; 127.754)	(119.306; 131.627)	(120.915; 128.037)				
Minimum	96.0	93.0	93.0				
First quartile	116.00	114.00	115.00				
Median	125.00	126.50	125.00				
Third quartile	132.00	135.00	133.00				
Maximum	148.0	156.0	156.0				
Week 12							
N	32	27	59				
Mean	121.66	123.81	122.64				
Std. Err.	1.966	3.076	1.756				
Std. Dev.	11.123	15.986	13.485				
95% C.I.	(117.646; 125.666)	(117.491; 130.139)	(119.130; 126.158)				
Minimum	91.0	88.0	88.0				
First quartile	115.50	112.00	113.00				
Median	123.50	125.00	124.00				
Third quartile	128.00	137.00	129.00				
Maximum	144.0	155.0	155.0				
Week 16							
N	8	28	36				
Mean	130.38	124.18	125.56				
Std. Err.	5.217	3.137	2.703				
Std. Dev.	14.755	16.600	16.215				
95% C.I.	(118.040; 142.710)	(117.742; 130.615)	(120.069; 131.042)				
Minimum	107.0	90.0	90.0				
First quartile	120.50	113.00	115.50				
Median	130.50	123.50	124.50				
Third quartile	141.50	135.50	135.50				
Maximum	151.0	159.0	159.0				
Week 20							
N	0	24	24				
Mean	-	122.83	122.83				
Std. Err.	-	2.887	2.887				
Std. Dev.	-	14.141	14.141				
95% C.I.	-	(116.862; 128.805)	(116.862; 128.805)				
Minimum	-	99.0	99.0				
First quartile	-	110.00	110.00				
Median	-	122.50	122.50				
Third quartile	-	130.50	130.50				
Maximum	-	146.0	146.0				
Week 24							
N	0	24	24				
Mean	-	123.38	123.38				
Std. Err.	-	3.088	3.088				
Std. Dev.	-	15.128	15.128				
95% C.I.	-	(116.987; 129.763)	(116.987; 129.763)				
Minimum	-	101.0	101.0				
First quartile	-	109.50	109.50				
Median	-	124.50	124.50				
Third quartile	-	136.00	136.00				
Maximum	-	146.0	146.0				
Week 28							
N	0	3	3				
Mean	-	131.33	131.33				
Std. Err.	-	14.948	14.948				
Std. Dev.	-	25.891	25.891				
95% C.I.	-	(67.017; 195.650)	(67.017; 195.650)				
Minimum	-	102.0	102.0				
First quartile	-	102.00	102.00				
Median	-	141.00	141.00				
Third quartile	-	151.00	151.00				
Maximum	-	151.0	151.0				
Week 36							
N	0	0	0				
Mean	-	-	-				
95% C.I.	-	-	-				
Minimum	-	-	-				
First quartile	-	-	-				
Median	-	-	-				
Third quartile	-	-	-				
Maximum	-	-	-				
Week 48							
N	0	0	0				
Mean	-	-	-				
95% C.I.	-	-	-				
Minimum	-	-	-				
First quartile	-	-	-				
Median	-	-	-				
Third quartile	-	-	-				
Maximum	-	-	-				
EOT							
N	34	32	66				
Mean	122.18	124.56	123.33				
Std. Err.	1.995	2.653	1.640				
Std. Dev.	11.632	15.007	13.325				
95% C.I.	(118.118; 126.235)	(119.152; 129.973)	(120.058; 126.609)				
Minimum	91.0	101.0	91.0				
First quartile	114.00	111.00	113.00				
Median	124.00	125.50	124.50				
Third quartile	128.00	135.50	131.00				
Maximum	144.0	148.0	148.0				
Neutrophils and Precursors (x10E9/L)							
Baseline							
N	34	33	67				
Mean	3.38	3.37	3.38				
Std. Err.	0.422	0.218	0.238				
Std. Dev.	2.463	1.253	1.948				
95% C.I.	(2.523; 4.242)	(2.930; 3.819)	(2.903; 3.854)				
Minimum	1.3	1.0	1.0				
First quartile	2.34	2.64	2.34				
Median	2.71	3.33	3.16				
Third quartile	3.54	3.96	3.89				
Maximum	15.4	6.3	15.4				
Week 01							
N	33	31	64				
Mean	1.85	1.83	1.84				
Std. Err.	0.141	0.135	0.097				
Std. Dev.	0.809	0.751	0.775				
95% C.I.	(1.568; 2.142)	(1.554; 2.105)	(1.649; 2.036)				
Minimum	0.5	0.8	0.5				
First quartile	1.36	1.30	1.32				
Median	1.85	1.79	1.83				
Third quartile	2.16	2.22	2.18				
Maximum	3.9	3.9	3.9				
Week 02							
N	31	30	61				
Mean	1.78	1.77	1.78				
Std. Err.	0.147	0.195	0.121				
Std. Dev.	0.820	1.069	0.942				
95% C.I.	(1.480; 2.082)	(1.372; 2.170)	(1.535; 2.017)				
Minimum	0.5	0.7	0.5				
First quartile	1.09	1.22	1.14				
Median	1.71	1.55	1.64				
Third quartile	2.12	1.87	2.10				
Maximum	3.7	6.4	6.4				
Week 04							
N	34	32	66				
Mean	1.49	1.91	1.69				
Std. Err.	0.080	0.232	0.122				
Std. Dev.	0.468	1.312	0.988				
95% C.I.	(1.327; 1.654)	(1.433; 2.379)	(1.449; 1.935)				
Minimum	0.6	0.7	0.6				
First quartile	1.23	1.21	1.23				
Median	1.46	1.61	1.52				
Third quartile	1.91	2.22	1.96				
Maximum	2.3	8.0	8.0				
Week 08							
N	33	30	63				
Mean	1.46	1.88	1.66				
Std. Err.	0.108	0.303	0.156				
Std. Dev.	0.623	1.659	1.238				
95% C.I.	(1.239; 1.681)	(1.257; 2.496)	(1.347; 1.970)				
Minimum	0.7	0.8	0.7				
First quartile	1.04	1.16	1.04				
Median	1.38	1.45	1.40				
Third quartile	1.76	1.95	1.87				
Maximum	3.2	9.6	9.6				
Week 12							
N	32	27	59				
Mean	1.49	1.40	1.45				
Std. Err.	0.179	0.103	0.107				
Std. Dev.	1.015	0.537	0.825				
95% C.I.	(1.124; 1.856)	(1.191; 1.616)	(1.235; 1.665)				
Minimum	0.6	0.7	0.6				
First quartile	0.91	1.07	0.97				
Median	1.20	1.28	1.24				
Third quartile	1.62	1.67	1.67				
Maximum	5.2	3.2	5.2				
Week 16							
N	8	28	36				
Mean	1.87	1.54	1.61				
Std. Err.	0.201	0.160	0.133				
Std. Dev.	0.569	0.849	0.800				
95% C.I.	(1.397; 2.348)	(1.211; 1.869)	(1.344; 1.885)				
Minimum	1.2	0.5	0.5				
First quartile	1.31	1.11	1.21				
Median	1.92	1.43	1.44				
Third quartile	2.43	1.73	2.04				
Maximum	2.5	4.0	4.0				
Week 20							
N	0	24	24				
Mean	-	1.42	1.42				
Std. Err.	-	0.133	0.133				
Std. Dev.	-	0.651	0.651				
95% C.I.	-	(1.144; 1.694)	(1.144; 1.694)				
Minimum	-	0.6	0.6				
First quartile	-	0.89	0.89				
Median	-	1.33	1.33				
Third quartile	-	1.76	1.76				
Maximum	-	2.8	2.8				
Week 24							
N	0	24	24				
Mean	-	2.40	2.40				
Std. Err.	-	0.579	0.579				
Std. Dev.	-	2.835	2.835				
95% C.I.	-	(1.206; 3.600)	(1.206; 3.600)				
Minimum	-	0.6	0.6				
First quartile	-	1.18	1.18				
Median	-	1.39	1.39				
Third quartile	-	2.38	2.38				
Maximum	-	14.0	14.0				
Week 28							
N	0	3	3				
Mean	-	2.70	2.70				
Std. Err.	-	0.725	0.725				
Std. Dev.	-	1.256	1.256				
95% C.I.	-	(-0.417; 5.824)	(-0.417; 5.824)				
Minimum	-	1.5	1.5				
First quartile	-	1.48	1.48				
Median	-	2.64	2.64				
Third quartile	-	3.99	3.99				
Maximum	-	4.0	4.0				
Week 36							
N	0	0	0				
Mean	-	-	-				
95% C.I.	-	-	-				
Minimum	-	-	-				
First quartile	-	-	-				
Median	-	-	-				
Third quartile	-	-	-				
Maximum	-	-	-				
Week 48							
N	0	0	0				
Mean	-	-	-				
95% C.I.	-	-	-				
Minimum	-	-	-				
First quartile	-	-	-				
Median	-	-	-				
Third quartile	-	-	-				
Maximum	-	-	-				
EOT							
N	34	32	66				
Mean	1.44	2.02	1.72				
Std. Err.	0.172	0.441	0.232				
Std. Dev.	1.001	2.493	1.886				
95% C.I.	(1.090; 1.789)	(1.118; 2.916)	(1.256; 2.183)				
Minimum	0.5	0.6	0.5				
First quartile	0.90	1.10	0.92				
Median	1.16	1.29	1.20				
Third quartile	1.52	1.95	1.72				
Maximum	5.2	14.0	14.0				
Platelets (x10E9/L)							
Baseline							
N	34	33	67				
Mean	226.24	224.64	225.45				
Std. Err.	8.360	8.525	5.924				
Std. Dev.	48.749	48.971	48.494				
95% C.I.	(209.226; 243.245)	(207.272; 242.001)	(213.619; 237.276)				
Minimum	116.0	118.0	116.0				
First quartile	178.00	203.00	190.00				
Median	236.50	234.00	235.00				
Third quartile	252.00	259.00	255.00				
Maximum	349.0	307.0	349.0				
Week 01							
N	34	30	64				
Mean	179.09	179.83	179.44				
Std. Err.	8.122	8.768	5.912				
Std. Dev.	47.361	48.024	47.294				
95% C.I.	(162.563; 195.613)	(161.901; 197.766)	(167.624; 191.251)				
Minimum	104.0	71.0	71.0				
First quartile	140.00	154.00	150.50				
Median	183.00	162.50	169.00				
Third quartile	202.00	223.00	214.00				
Maximum	302.0	294.0	302.0				
Week 02							
N	31	29	60				
Mean	176.45	179.97	178.15				
Std. Err.	9.934	11.453	7.488				
Std. Dev.	55.308	61.677	57.998				
95% C.I.	(156.165; 196.739)	(156.505; 203.426)	(163.167; 193.133)				
Minimum	110.0	65.0	65.0				
First quartile	134.00	142.00	137.00				
Median	170.00	176.00	172.00				
Third quartile	204.00	206.00	205.00				
Maximum	363.0	351.0	363.0				
Week 04							
N	34	32	66				
Mean	188.97	183.41	186.27				
Std. Err.	10.814	11.233	7.739				
Std. Dev.	63.058	63.546	62.868				
95% C.I.	(166.969; 210.972)	(160.496; 206.317)	(170.818; 201.728)				
Minimum	102.0	53.0	53.0				
First quartile	142.00	150.00	149.00				
Median	175.50	188.50	183.50				
Third quartile	222.00	207.00	215.00				
Maximum	332.0	328.0	332.0				
Week 08							
N	33	30	63				
Mean	157.91	169.03	163.21				
Std. Err.	7.624	8.902	5.819				
Std. Dev.	43.795	48.759	46.188				
95% C.I.	(142.380; 173.438)	(150.826; 187.240)	(151.574; 174.839)				
Minimum	92.0	59.0	59.0				
First quartile	131.00	139.00	132.00				
Median	147.00	163.50	155.00				
Third quartile	174.00	198.00	193.00				
Maximum	282.0	283.0	283.0				
Week 12							
N	31	27	58				
Mean	160.94	167.59	164.03				
Std. Err.	8.818	9.341	6.371				
Std. Dev.	49.094	48.537	48.522				
95% C.I.	(142.928; 178.943)	(148.392; 186.793)	(151.276; 176.793)				
Minimum	97.0	58.0	58.0				
First quartile	127.00	135.00	130.00				
Median	158.00	169.00	160.50				
Third quartile	182.00	187.00	187.00				
Maximum	284.0	308.0	308.0				
Week 16							
N	8	28	36				
Mean	189.75	156.96	164.25				
Std. Err.	19.341	8.607	8.147				
Std. Dev.	54.704	45.542	48.883				
95% C.I.	(144.017; 235.483)	(139.305; 174.623)	(147.710; 180.790)				
Minimum	141.0	61.0	61.0				
First quartile	144.50	132.00	138.00				
Median	178.00	153.50	158.50				
Third quartile	214.50	177.00	180.00				
Maximum	303.0	258.0	303.0				
Week 20							
N	0	24	24				
Mean	-	161.42	161.42				
Std. Err.	-	10.469	10.469				
Std. Dev.	-	51.289	51.289				
95% C.I.	-	(139.759; 183.074)	(139.759; 183.074)				
Minimum	-	65.0	65.0				
First quartile	-	132.00	132.00				
Median	-	155.50	155.50				
Third quartile	-	181.00	181.00				
Maximum	-	275.0	275.0				
Week 24							
N	0	24	24				
Mean	-	175.33	175.33				
Std. Err.	-	10.498	10.498				
Std. Dev.	-	51.429	51.429				
95% C.I.	-	(153.617; 197.050)	(153.617; 197.050)				
Minimum	-	99.0	99.0				
First quartile	-	136.00	136.00				
Median	-	173.00	173.00				
Third quartile	-	200.00	200.00				
Maximum	-	334.0	334.0				
Week 28							
N	0	3	3				
Mean	-	219.33	219.33				
Std. Err.	-	4.667	4.667				
Std. Dev.	-	8.083	8.083				
95% C.I.	-	(199.254; 239.412)	(199.254; 239.412)				
Minimum	-	210.0	210.0				
First quartile	-	210.00	210.00				
Median	-	224.00	224.00				
Third quartile	-	224.00	224.00				
Maximum	-	224.0	224.0				
Week 36							
N	0	0	0				
Mean	-	-	-				
95% C.I.	-	-	-				
Minimum	-	-	-				
First quartile	-	-	-				
Median	-	-	-				
Third quartile	-	-	-				
Maximum	-	-	-				
Week 48							
N	0	0	0				
Mean	-	-	-				
95% C.I.	-	-	-				
Minimum	-	-	-				
First quartile	-	-	-				
Median	-	-	-				
Third quartile	-	-	-				
Maximum	-	-	-				
EOT							
N	34	32	66				
Mean	158.03	160.97	159.45				
Std. Err.	8.112	9.973	6.344				
Std. Dev.	47.303	56.415	51.537				
95% C.I.	(141.524; 174.534)	(140.629; 181.308)	(146.785; 172.124)				
Minimum	97.0	62.0	62.0				
First quartile	125.00	132.00	128.00				
Median	156.50	153.00	156.50				
Third quartile	178.00	192.00	188.00				
Maximum	284.0	334.0	334.0				
Direct Bilirubin (umol/L)							
Baseline							
N	34	33	67				
Mean	2.53	2.91	2.72				
Std. Err.	0.175	0.196	0.132				
Std. Dev.	1.022	1.128	1.084				
95% C.I.	(2.173; 2.886)	(2.509; 3.309)	(2.452; 2.981)				
Minimum	1.0	1.0	1.0				
First quartile	2.00	2.00	2.00				
Median	2.00	3.00	3.00				
Third quartile	3.00	3.00	3.00				
Maximum	5.0	6.0	6.0				
Week 01							
N	33	29	62				
Mean	4.15	4.76	4.44				
Std. Err.	0.335	0.320	0.234				
Std. Dev.	1.922	1.725	1.843				
95% C.I.	(3.470; 4.833)	(4.102; 5.415)	(3.967; 4.904)				
Minimum	1.0	2.0	1.0				
First quartile	3.00	4.00	3.00				
Median	4.00	4.00	4.00				
Third quartile	5.00	6.00	5.00				
Maximum	10.0	9.0	10.0				
Week 02							
N	31	29	60				
Mean	4.58	5.93	5.23				
Std. Err.	0.261	0.371	0.239				
Std. Dev.	1.455	1.999	1.854				
95% C.I.	(4.047; 5.115)	(5.171; 6.691)	(4.754; 5.712)				
Minimum	2.0	3.0	2.0				
First quartile	4.00	5.00	4.00				
Median	4.00	6.00	5.00				
Third quartile	5.00	6.00	6.00				
Maximum	8.0	11.0	11.0				
Week 04							
N	32	31	63				
Mean	4.13	5.52	4.81				
Std. Err.	0.272	0.444	0.271				
Std. Dev.	1.540	2.475	2.154				
95% C.I.	(3.570; 4.680)	(4.608; 6.424)	(4.267; 5.352)				
Minimum	2.0	3.0	2.0				
First quartile	3.00	4.00	3.00				
Median	4.00	5.00	4.00				
Third quartile	5.00	7.00	6.00				
Maximum	10.0	13.0	13.0				
Week 08							
N	33	29	62				
Mean	4.45	6.07	5.21				
Std. Err.	0.351	0.620	0.357				
Std. Dev.	2.017	3.337	2.812				
95% C.I.	(3.739; 5.170)	(4.800; 7.338)	(4.496; 5.924)				
Minimum	1.0	2.0	1.0				
First quartile	3.00	4.00	3.00				
Median	4.00	4.00	4.00				
Third quartile	6.00	8.00	6.00				
Maximum	12.0	16.0	16.0				
Week 12							
N	33	28	61				
Mean	4.42	5.43	4.89				
Std. Err.	0.302	0.550	0.305				
Std. Dev.	1.733	2.911	2.381				
95% C.I.	(3.810; 5.039)	(4.300; 6.557)	(4.275; 5.495)				
Minimum	2.0	3.0	2.0				
First quartile	3.00	3.00	3.00				
Median	4.00	5.00	4.00				
Third quartile	6.00	6.00	6.00				
Maximum	8.0	14.0	14.0				
Week 16							
N	8	27	35				
Mean	1.38	3.00	2.63				
Std. Err.	0.263	0.226	0.217				
Std. Dev.	0.744	1.177	1.285				
95% C.I.	(0.753; 1.997)	(2.535; 3.465)	(2.187; 3.070)				
Minimum	1.0	1.0	1.0				
First quartile	1.00	2.00	2.00				
Median	1.00	3.00	3.00				
Third quartile	1.50	4.00	3.00				
Maximum	3.0	7.0	7.0				
Week 20							
N	0	25	25				
Mean	-	2.80	2.80				
Std. Err.	-	0.191	0.191				
Std. Dev.	-	0.957	0.957				
95% C.I.	-	(2.405; 3.195)	(2.405; 3.195)				
Minimum	-	1.0	1.0				
First quartile	-	2.00	2.00				
Median	-	3.00	3.00				
Third quartile	-	3.00	3.00				
Maximum	-	5.0	5.0				
Week 24							
N	0	25	25				
Mean	-	2.76	2.76				
Std. Err.	-	0.194	0.194				
Std. Dev.	-	0.970	0.970				
95% C.I.	-	(2.360; 3.160)	(2.360; 3.160)				
Minimum	-	1.0	1.0				
First quartile	-	2.00	2.00				
Median	-	3.00	3.00				
Third quartile	-	3.00	3.00				
Maximum	-	4.0	4.0				
Week 28							
N	0	3	3				
Mean	-	1.67	1.67				
Std. Err.	-	0.333	0.333				
Std. Dev.	-	0.577	0.577				
95% C.I.	-	(0.232; 3.101)	(0.232; 3.101)				
Minimum	-	1.0	1.0				
First quartile	-	1.00	1.00				
Median	-	2.00	2.00				
Third quartile	-	2.00	2.00				
Maximum	-	2.0	2.0				
Week 36							
N	0	0	0				
Mean	-	-	-				
95% C.I.	-	-	-				
Minimum	-	-	-				
First quartile	-	-	-				
Median	-	-	-				
Third quartile	-	-	-				
Maximum	-	-	-				
Week 48							
N	0	0	0				
Mean	-	-	-				
95% C.I.	-	-	-				
Minimum	-	-	-				
First quartile	-	-	-				
Median	-	-	-				
Third quartile	-	-	-				
Maximum	-	-	-				
EOT							
N	34	32	66				
Mean	4.56	3.16	3.88				
Std. Err.	0.299	0.262	0.216				
Std. Dev.	1.744	1.483	1.759				
95% C.I.	(3.950; 5.167)	(2.621; 3.691)	(3.446; 4.311)				
Minimum	2.0	1.0	1.0				
First quartile	3.00	2.50	3.00				
Median	4.00	3.00	3.00				
Third quartile	6.00	4.00	5.00				
Maximum	8.0	9.0	9.0				
Indirect Bilirubin (umol/L)							
Baseline							
N	34	33	67				
Mean	7.03	7.09	7.06				
Std. Err.	0.623	0.551	0.413				
Std. Dev.	3.631	3.166	3.384				
95% C.I.	(5.763; 8.296)	(5.968; 8.213)	(6.234; 7.885)				
Minimum	3.0	3.0	3.0				
First quartile	5.00	5.00	5.00				
Median	6.00	7.00	6.00				
Third quartile	8.00	8.00	8.00				
Maximum	19.0	21.0	21.0				
Week 01							
N	33	29	62				
Mean	12.85	14.83	13.77				
Std. Err.	1.531	2.297	1.343				
Std. Dev.	8.797	12.372	10.576				
95% C.I.	(9.729; 15.968)	(10.121; 19.534)	(11.088; 16.460)				
Minimum	3.0	5.0	3.0				
First quartile	6.00	8.00	7.00				
Median	9.00	11.00	11.00				
Third quartile	18.00	16.00	17.00				
Maximum	36.0	57.0	57.0				
Week 02							
N	31	29	60				
Mean	11.32	15.52	13.35				
Std. Err.	1.147	1.619	1.011				
Std. Dev.	6.384	8.720	7.828				
95% C.I.	(8.981; 13.664)	(12.200; 18.834)	(11.328; 15.372)				
Minimum	3.0	5.0	3.0				
First quartile	7.00	11.00	8.50				
Median	11.00	14.00	12.00				
Third quartile	13.00	16.00	15.00				
Maximum	33.0	52.0	52.0				
Week 04							
N	32	31	63				
Mean	10.88	13.97	12.40				
Std. Err.	1.146	1.572	0.980				
Std. Dev.	6.484	8.750	7.778				
95% C.I.	(8.537; 13.213)	(10.758; 17.177)	(10.438; 14.356)				
Minimum	5.0	5.0	5.0				
First quartile	7.00	8.00	7.00				
Median	9.50	11.00	10.00				
Third quartile	12.00	16.00	14.00				
Maximum	35.0	48.0	48.0				
Week 08							
N	33	29	62				
Mean	12.24	13.55	12.85				
Std. Err.	1.328	1.591	1.021				
Std. Dev.	7.628	8.567	8.041				
95% C.I.	(9.538; 14.947)	(10.293; 16.811)	(10.813; 14.897)				
Minimum	5.0	5.0	5.0				
First quartile	8.00	7.00	8.00				
Median	10.00	11.00	10.00				
Third quartile	13.00	14.00	13.00				
Maximum	34.0	38.0	38.0				
Week 12							
N	33	28	61				
Mean	11.15	11.75	11.43				
Std. Err.	1.150	1.003	0.769				
Std. Dev.	6.605	5.310	6.004				
95% C.I.	(8.809; 13.494)	(9.691; 13.809)	(9.889; 12.964)				
Minimum	4.0	5.0	4.0				
First quartile	7.00	8.00	8.00				
Median	9.00	10.00	10.00				
Third quartile	13.00	15.50	14.00				
Maximum	38.0	24.0	38.0				
Week 16							
N	8	27	35				
Mean	4.63	7.56	6.89				
Std. Err.	0.565	0.535	0.478				
Std. Dev.	1.598	2.778	2.826				
95% C.I.	(3.289; 5.961)	(6.457; 8.655)	(5.915; 7.856)				
Minimum	3.0	4.0	3.0				
First quartile	3.50	6.00	5.00				
Median	4.50	7.00	7.00				
Third quartile	5.00	9.00	8.00				
Maximum	8.0	14.0	14.0				
Week 20							
N	0	25	25				
Mean	-	7.24	7.24				
Std. Err.	-	0.681	0.681				
Std. Dev.	-	3.407	3.407				
95% C.I.	-	(5.834; 8.646)	(5.834; 8.646)				
Minimum	-	4.0	4.0				
First quartile	-	5.00	5.00				
Median	-	7.00	7.00				
Third quartile	-	7.00	7.00				
Maximum	-	19.0	19.0				
Week 24							
N	0	25	25				
Mean	-	6.88	6.88				
Std. Err.	-	0.459	0.459				
Std. Dev.	-	2.297	2.297				
95% C.I.	-	(5.932; 7.828)	(5.932; 7.828)				
Minimum	-	3.0	3.0				
First quartile	-	6.00	6.00				
Median	-	6.00	6.00				
Third quartile	-	8.00	8.00				
Maximum	-	14.0	14.0				
Week 28							
N	0	3	3				
Mean	-	4.67	4.67				
Std. Err.	-	0.667	0.667				
Std. Dev.	-	1.155	1.155				
95% C.I.	-	(1.798; 7.535)	(1.798; 7.535)				
Minimum	-	4.0	4.0				
First quartile	-	4.00	4.00				
Median	-	4.00	4.00				
Third quartile	-	6.00	6.00				
Maximum	-	6.0	6.0				
Week 36							
N	0	0	0				
Mean	-	-	-				
95% C.I.	-	-	-				
Minimum	-	-	-				
First quartile	-	-	-				
Median	-	-	-				
Third quartile	-	-	-				
Maximum	-	-	-				
Week 48							
N	0	0	0				
Mean	-	-	-				
95% C.I.	-	-	-				
Minimum	-	-	-				
First quartile	-	-	-				
Median	-	-	-				
Third quartile	-	-	-				
Maximum	-	-	-				
EOT							
N	34	32	66				
Mean	11.85	7.91	9.94				
Std. Err.	1.227	0.723	0.758				
Std. Dev.	7.153	4.091	6.157				
95% C.I.	(9.357; 14.349)	(6.431; 9.381)	(8.426; 11.453)				
Minimum	6.0	3.0	3.0				
First quartile	8.00	6.00	6.00				
Median	9.00	7.00	8.00				
Third quartile	14.00	8.00	10.00				
Maximum	38.0	25.0	38.0				
Bilirubin (umol/L)							
Baseline							
N	34	33	67				
Mean	9.56	9.82	9.69				
Std. Err.	0.754	0.672	0.502				
Std. Dev.	4.398	3.860	4.113				
95% C.I.	(8.024; 11.093)	(8.449; 11.187)	(8.683; 10.690)				
Minimum	4.0	4.0	4.0				
First quartile	7.00	8.00	7.00				
Median	8.50	10.00	9.00				
Third quartile	11.00	11.00	11.00				
Maximum	23.0	26.0	26.0				
Week 01							
N	33	30	63				
Mean	17.00	19.47	18.17				
Std. Err.	1.773	2.319	1.439				
Std. Dev.	10.186	12.700	11.425				
95% C.I.	(13.388; 20.612)	(14.724; 24.209)	(15.297; 21.052)				
Minimum	5.0	7.0	5.0				
First quartile	10.00	12.00	10.00				
Median	13.00	16.50	15.00				
Third quartile	23.00	21.00	23.00				
Maximum	40.0	62.0	62.0				
Week 02							
N	31	30	61				
Mean	15.90	21.53	18.67				
Std. Err.	1.355	1.681	1.127				
Std. Dev.	7.547	9.209	8.805				
95% C.I.	(13.135; 18.671)	(18.095; 24.972)	(16.417; 20.927)				
Minimum	5.0	9.0	5.0				
First quartile	11.00	18.00	14.00				
Median	15.00	20.50	18.00				
Third quartile	18.00	24.00	21.00				
Maximum	41.0	57.0	57.0				
Week 04							
N	33	32	65				
Mean	15.42	19.31	17.34				
Std. Err.	1.389	1.736	1.126				
Std. Dev.	7.977	9.819	9.075				
95% C.I.	(12.596; 18.253)	(15.772; 22.853)	(15.090; 19.587)				
Minimum	7.0	9.0	7.0				
First quartile	10.00	13.00	12.00				
Median	13.00	16.50	14.00				
Third quartile	18.00	21.50	20.00				
Maximum	41.0	53.0	53.0				
Week 08							
N	33	30	63				
Mean	16.70	19.70	18.13				
Std. Err.	1.618	1.913	1.249				
Std. Dev.	9.295	10.475	9.910				
95% C.I.	(13.401; 19.993)	(15.788; 23.612)	(15.631; 20.623)				
Minimum	7.0	8.0	7.0				
First quartile	11.00	13.00	11.00				
Median	15.00	16.50	15.00				
Third quartile	18.00	22.00	19.00				
Maximum	46.0	45.0	46.0				
Week 12							
N	33	28	61				
Mean	15.58	17.18	16.31				
Std. Err.	1.361	1.342	0.958				
Std. Dev.	7.818	7.103	7.480				
95% C.I.	(12.803; 18.348)	(14.424; 19.933)	(14.396; 18.227)				
Minimum	7.0	8.0	7.0				
First quartile	10.00	13.00	11.00				
Median	14.00	15.00	14.00				
Third quartile	18.00	20.50	19.00				
Maximum	45.0	36.0	45.0				
Week 16							
N	8	28	36				
Mean	6.00	10.57	9.56				
Std. Err.	0.779	0.653	0.622				
Std. Dev.	2.204	3.458	3.730				
95% C.I.	(4.157; 7.843)	(9.231; 11.912)	(8.294; 10.818)				
Minimum	4.0	5.0	4.0				
First quartile	4.50	8.00	6.50				
Median	6.00	10.00	9.50				
Third quartile	6.00	12.00	11.00				
Maximum	11.0	20.0	20.0				
Week 20							
N	0	26	26				
Mean	-	10.15	10.15				
Std. Err.	-	0.809	0.809				
Std. Dev.	-	4.125	4.125				
95% C.I.	-	(8.488; 11.820)	(8.488; 11.820)				
Minimum	-	6.0	6.0				
First quartile	-	8.00	8.00				
Median	-	9.00	9.00				
Third quartile	-	10.00	10.00				
Maximum	-	24.0	24.0				
Week 24							
N	0	25	25				
Mean	-	9.64	9.64				
Std. Err.	-	0.586	0.586				
Std. Dev.	-	2.928	2.928				
95% C.I.	-	(8.431; 10.849)	(8.431; 10.849)				
Minimum	-	5.0	5.0				
First quartile	-	8.00	8.00				
Median	-	9.00	9.00				
Third quartile	-	11.00	11.00				
Maximum	-	18.0	18.0				
Week 28							
N	0	3	3				
Mean	-	6.33	6.33				
Std. Err.	-	0.882	0.882				
Std. Dev.	-	1.528	1.528				
95% C.I.	-	(2.539; 10.128)	(2.539; 10.128)				
Minimum	-	5.0	5.0				
First quartile	-	5.00	5.00				
Median	-	6.00	6.00				
Third quartile	-	8.00	8.00				
Maximum	-	8.0	8.0				
Week 36							
N	0	0	0				
Mean	-	-	-				
95% C.I.	-	-	-				
Minimum	-	-	-				
First quartile	-	-	-				
Median	-	-	-				
Third quartile	-	-	-				
Maximum	-	-	-				
Week 48							
N	0	0	0				
Mean	-	-	-				
95% C.I.	-	-	-				
Minimum	-	-	-				
First quartile	-	-	-				
Median	-	-	-				
Third quartile	-	-	-				
Maximum	-	-	-				
EOT							
N	34	32	66				
Mean	16.41	11.06	13.82				
Std. Err.	1.438	0.957	0.929				
Std. Dev.	8.385	5.412	7.545				
95% C.I.	(13.486; 19.337)	(9.111; 13.014)	(11.963; 15.673)				
Minimum	8.0	6.0	6.0				
First quartile	11.00	8.00	9.00				
Median	14.00	10.00	11.50				
Third quartile	19.00	12.00	15.00				
Maximum	45.0	34.0	45.0				
	
	
