# Supplementary material for: Efficacy of a 12-Week Simeprevir Plus Peginterferon/Ribavirin (PR) Regimen in Treatment-Naïve Patients with Hepatitis C Virus (HCV) Genotype 4 (GT4) Infection and Mild-To-Moderate Fibrosis Displaying Early On-Treatment Virologic Response
Source: PLoS One. 2017 Jan 5;12(1):e0168713. doi: 10.1371/journal.pone.0168713 (PMC5215882; doi:10.1371/journal.pone.0168713)
Supplement: S1 Dataset — (ZIP) [file pone.0168713.s002.zip › TSFLAB02-GT.rtf]

TSFLAB02-GT:	Change from Baseline in Laboratory Parameters by Analysis Timepoint (Entire Treatment Phase); Intent-to-treat (Study TMC435HPC3014)	
	Simeprevir
12 Wks
150 mg
PR 12/24 	
	 Genotype 4 	 	
	 12 Wks
(N=34) 	 >12 Wks
(N=33) 	 All subjects
(N=67) 				
Hemoglobin (g/L)							
Week 01							
N	34	31	65				
Mean	-1.59	-1.23	-1.42				
Std. Err.	0.871	1.146	0.706				
Std. Dev.	5.076	6.381	5.692				
95% C.I.	(-3.359; 0.183)	(-3.566; 1.115)	(-2.826; -0.005)				
Minimum	-12.0	-14.0	-14.0				
First quartile	-5.00	-5.00	-5.00				
Median	-1.00	-1.00	-1.00				
Third quartile	1.00	2.00	2.00				
Maximum	10.0	12.0	12.0				
Week 02							
N	31	30	61				
Mean	-12.16	-9.97	-11.08				
Std. Err.	1.744	1.901	1.285				
Std. Dev.	9.709	10.414	10.039				
95% C.I.	(-15.723; -8.600)	(-13.855; -6.078)	(-13.653; -8.511)				
Minimum	-37.0	-28.0	-37.0				
First quartile	-16.00	-18.00	-17.00				
Median	-13.00	-11.50	-13.00				
Third quartile	-7.00	-4.00	-7.00				
Maximum	12.0	17.0	17.0				
Week 04							
N	34	32	66				
Mean	-19.97	-22.19	-21.05				
Std. Err.	1.805	2.391	1.481				
Std. Dev.	10.527	13.523	12.030				
95% C.I.	(-23.644; -16.298)	(-27.063; -17.312)	(-24.003; -18.088)				
Minimum	-46.0	-46.0	-46.0				
First quartile	-25.00	-31.00	-29.00				
Median	-18.00	-21.50	-20.00				
Third quartile	-14.00	-15.00	-15.00				
Maximum	1.0	3.0	3.0				
Week 08							
N	33	30	63				
Mean	-27.00	-25.13	-26.11				
Std. Err.	1.917	2.593	1.583				
Std. Dev.	11.011	14.205	12.564				
95% C.I.	(-30.904; -23.096)	(-30.437; -19.829)	(-29.275; -22.947)				
Minimum	-51.0	-65.0	-65.0				
First quartile	-33.00	-33.00	-33.00				
Median	-26.00	-25.00	-25.00				
Third quartile	-22.00	-16.00	-18.00				
Maximum	0.0	8.0	8.0				
Week 12							
N	32	27	59				
Mean	-27.53	-26.67	-27.14				
Std. Err.	1.861	3.002	1.690				
Std. Dev.	10.525	15.598	12.979				
95% C.I.	(-31.326; -23.737)	(-32.837; -20.496)	(-30.518; -23.753)				
Minimum	-51.0	-70.0	-70.0				
First quartile	-35.50	-31.00	-34.00				
Median	-25.50	-27.00	-27.00				
Third quartile	-18.50	-17.00	-18.00				
Maximum	-13.0	4.0	4.0				
Week 16							
N	8	28	36				
Mean	-19.25	-26.14	-24.61				
Std. Err.	3.614	2.300	1.997				
Std. Dev.	10.223	12.171	11.984				
95% C.I.	(-27.796; -10.704)	(-30.862; -21.424)	(-28.666; -20.556)				
Minimum	-39.0	-43.0	-43.0				
First quartile	-23.00	-37.00	-35.50				
Median	-19.50	-25.50	-23.00				
Third quartile	-12.00	-20.00	-19.00				
Maximum	-6.0	0.0	0.0				
Week 20							
N	0	24	24				
Mean	-	-25.21	-25.21				
Std. Err.	-	2.481	2.481				
Std. Dev.	-	12.155	12.155				
95% C.I.	-	(-30.341; -20.076)	(-30.341; -20.076)				
Minimum	-	-46.0	-46.0				
First quartile	-	-34.00	-34.00				
Median	-	-27.00	-27.00				
Third quartile	-	-20.00	-20.00				
Maximum	-	0.0	0.0				
Week 24							
N	0	25	25				
Mean	-	-25.64	-25.64				
Std. Err.	-	2.772	2.772				
Std. Dev.	-	13.862	13.862				
95% C.I.	-	(-31.362; -19.918)	(-31.362; -19.918)				
Minimum	-	-56.0	-56.0				
First quartile	-	-34.00	-34.00				
Median	-	-28.00	-28.00				
Third quartile	-	-18.00	-18.00				
Maximum	-	5.0	5.0				
Week 28							
N	0	6	6				
Mean	-	-17.00	-17.00				
Std. Err.	-	4.235	4.235				
Std. Dev.	-	10.373	10.373				
95% C.I.	-	(-27.886; -6.114)	(-27.886; -6.114)				
Minimum	-	-32.0	-32.0				
First quartile	-	-21.00	-21.00				
Median	-	-17.50	-17.50				
Third quartile	-	-13.00	-13.00				
Maximum	-	-1.0	-1.0				
Week 36							
N	0	0	0				
Mean	-	-	-				
95% C.I.	-	-	-				
Minimum	-	-	-				
First quartile	-	-	-				
Median	-	-	-				
Third quartile	-	-	-				
Maximum	-	-	-				
Week 48							
N	0	0	0				
Mean	-	-	-				
95% C.I.	-	-	-				
Minimum	-	-	-				
First quartile	-	-	-				
Median	-	-	-				
Third quartile	-	-	-				
Maximum	-	-	-				
EOT							
N	34	32	66				
Mean	-28.29	-26.78	-27.56				
Std. Err.	1.861	2.476	1.527				
Std. Dev.	10.850	14.004	12.405				
95% C.I.	(-32.080; -24.508)	(-31.830; -21.732)	(-30.610; -24.511)				
Minimum	-51.0	-56.0	-56.0				
First quartile	-37.00	-35.50	-36.00				
Median	-28.00	-28.50	-28.50				
Third quartile	-19.00	-20.00	-19.00				
Maximum	-13.0	3.0	3.0				
Neutrophils and Precursors (x10E9/L)							
Week 01							
N	33	31	64				
Mean	-1.53	-1.55	-1.54				
Std. Err.	0.434	0.181	0.239				
Std. Dev.	2.494	1.006	1.908				
95% C.I.	(-2.417; -0.649)	(-1.924; -1.186)	(-2.020; -1.067)				
Minimum	-14.2	-3.7	-14.2				
First quartile	-1.50	-2.19	-1.95				
Median	-1.01	-1.42	-1.18				
Third quartile	-0.44	-0.90	-0.58				
Maximum	0.7	0.2	0.7				
Week 02							
N	31	30	61				
Mean	-1.65	-1.60	-1.63				
Std. Err.	0.450	0.263	0.261				
Std. Dev.	2.506	1.441	2.035				
95% C.I.	(-2.573; -0.735)	(-2.136; -1.060)	(-2.148; -1.105)				
Minimum	-13.6	-4.5	-13.6				
First quartile	-1.58	-2.37	-2.03				
Median	-1.01	-1.66	-1.39				
Third quartile	-0.65	-0.89	-0.66				
Maximum	0.8	3.0	3.0				
Week 04							
N	34	32	66				
Mean	-1.89	-1.48	-1.69				
Std. Err.	0.427	0.332	0.272				
Std. Dev.	2.491	1.878	2.208				
95% C.I.	(-2.761; -1.023)	(-2.158; -0.803)	(-2.235; -1.150)				
Minimum	-14.2	-4.1	-14.2				
First quartile	-1.84	-2.52	-2.20				
Median	-1.53	-1.80	-1.63				
Third quartile	-0.73	-0.84	-0.77				
Maximum	0.3	6.3	6.3				
Week 08							
N	33	30	63				
Mean	-1.94	-1.52	-1.74				
Std. Err.	0.390	0.395	0.277				
Std. Dev.	2.240	2.163	2.196				
95% C.I.	(-2.735; -1.147)	(-2.326; -0.711)	(-2.293; -1.187)				
Minimum	-12.2	-4.4	-12.2				
First quartile	-2.24	-2.65	-2.47				
Median	-1.44	-2.12	-1.66				
Third quartile	-0.93	-0.87	-0.87				
Maximum	0.3	7.8	7.8				
Week 12							
N	32	27	59				
Mean	-1.78	-2.08	-1.92				
Std. Err.	0.465	0.230	0.272				
Std. Dev.	2.630	1.198	2.089				
95% C.I.	(-2.732; -0.835)	(-2.557; -1.610)	(-2.465; -1.376)				
Minimum	-14.3	-4.5	-14.3				
First quartile	-2.06	-3.10	-2.33				
Median	-1.40	-2.14	-1.74				
Third quartile	-0.79	-0.96	-0.88				
Maximum	3.0	0.1	3.0				
Week 16							
N	8	28	36				
Mean	-2.46	-1.76	-1.92				
Std. Err.	1.488	0.199	0.353				
Std. Dev.	4.210	1.053	2.118				
95% C.I.	(-5.981; 1.058)	(-2.167; -1.351)	(-2.632; -1.199)				
Minimum	-12.8	-3.6	-12.8				
First quartile	-1.48	-2.59	-2.48				
Median	-1.04	-1.76	-1.50				
Third quartile	-0.67	-1.02	-0.86				
Maximum	-0.5	0.7	0.7				
Week 20							
N	0	24	24				
Mean	-	-1.83	-1.83				
Std. Err.	-	0.238	0.238				
Std. Dev.	-	1.166	1.166				
95% C.I.	-	(-2.319; -1.335)	(-2.319; -1.335)				
Minimum	-	-3.5	-3.5				
First quartile	-	-2.65	-2.65				
Median	-	-2.19	-2.19				
Third quartile	-	-1.10	-1.10				
Maximum	-	0.8	0.8				
Week 24							
N	0	25	25				
Mean	-	-0.88	-0.88				
Std. Err.	-	0.640	0.640				
Std. Dev.	-	3.198	3.198				
95% C.I.	-	(-2.202; 0.439)	(-2.202; 0.439)				
Minimum	-	-3.7	-3.7				
First quartile	-	-2.72	-2.72				
Median	-	-1.51	-1.51				
Third quartile	-	-0.64	-0.64				
Maximum	-	12.3	12.3				
Week 28							
N	0	6	6				
Mean	-	-0.89	-0.89				
Std. Err.	-	0.690	0.690				
Std. Dev.	-	1.689	1.689				
95% C.I.	-	(-2.664; 0.881)	(-2.664; 0.881)				
Minimum	-	-3.7	-3.7				
First quartile	-	-1.26	-1.26				
Median	-	-0.86	-0.86				
Third quartile	-	-0.31	-0.31				
Maximum	-	1.6	1.6				
Week 36							
N	0	0	0				
Mean	-	-	-				
95% C.I.	-	-	-				
Minimum	-	-	-				
First quartile	-	-	-				
Median	-	-	-				
Third quartile	-	-	-				
Maximum	-	-	-				
Week 48							
N	0	0	0				
Mean	-	-	-				
95% C.I.	-	-	-				
Minimum	-	-	-				
First quartile	-	-	-				
Median	-	-	-				
Third quartile	-	-	-				
Maximum	-	-	-				
EOT							
N	34	32	66				
Mean	-1.94	-1.37	-1.67				
Std. Err.	0.449	0.508	0.337				
Std. Dev.	2.620	2.874	2.740				
95% C.I.	(-2.857; -1.029)	(-2.406; -0.334)	(-2.339; -0.992)				
Minimum	-14.3	-3.7	-14.3				
First quartile	-2.19	-2.78	-2.43				
Median	-1.50	-2.10	-1.78				
Third quartile	-0.88	-0.95	-0.88				
Maximum	3.0	12.3	12.3				
Platelets (x10E9/L)							
Week 01							
N	34	30	64				
Mean	-47.15	-50.20	-48.58				
Std. Err.	6.330	7.210	4.733				
Std. Dev.	36.908	39.492	37.866				
95% C.I.	(-60.025; -34.269)	(-64.947; -35.453)	(-58.037; -39.120)				
Minimum	-129.0	-134.0	-134.0				
First quartile	-65.00	-74.00	-67.00				
Median	-43.50	-52.50	-50.50				
Third quartile	-27.00	-35.00	-29.50				
Maximum	63.0	79.0	79.0				
Week 02							
N	31	29	60				
Mean	-52.48	-47.07	-49.87				
Std. Err.	7.462	9.362	5.904				
Std. Dev.	41.545	50.415	45.730				
95% C.I.	(-67.723; -37.245)	(-66.246; -27.892)	(-61.680; -38.053)				
Minimum	-160.0	-112.0	-160.0				
First quartile	-69.00	-74.00	-70.00				
Median	-54.00	-58.00	-55.00				
Third quartile	-39.00	-33.00	-34.00				
Maximum	47.0	136.0	136.0				
Week 04							
N	34	32	66				
Mean	-37.26	-43.06	-40.08				
Std. Err.	8.978	8.473	6.149				
Std. Dev.	52.348	47.930	49.954				
95% C.I.	(-55.530; -19.000)	(-60.343; -25.782)	(-52.356; -27.796)				
Minimum	-137.0	-109.0	-137.0				
First quartile	-68.00	-77.50	-71.00				
Median	-38.50	-51.50	-49.50				
Third quartile	-14.00	-21.00	-16.00				
Maximum	94.0	113.0	113.0				
Week 08							
N	33	30	63				
Mean	-65.76	-61.10	-63.54				
Std. Err.	7.468	8.014	5.429				
Std. Dev.	42.903	43.895	43.090				
95% C.I.	(-80.970; -50.545)	(-77.491; -44.709)	(-74.392; -52.688)				
Minimum	-158.0	-125.0	-158.0				
First quartile	-91.00	-89.00	-91.00				
Median	-60.00	-71.50	-67.00				
Third quartile	-45.00	-40.00	-43.00				
Maximum	26.0	54.0	54.0				
Week 12							
N	31	27	58				
Mean	-62.32	-63.63	-62.93				
Std. Err.	8.741	9.841	6.486				
Std. Dev.	48.669	51.136	49.395				
95% C.I.	(-80.175; -44.471)	(-83.858; -43.401)	(-75.919; -49.943)				
Minimum	-174.0	-157.0	-174.0				
First quartile	-83.00	-86.00	-84.00				
Median	-65.00	-70.00	-66.50				
Third quartile	-20.00	-28.00	-28.00				
Maximum	26.0	93.0	93.0				
Week 16							
N	8	28	36				
Mean	-19.88	-70.25	-59.06				
Std. Err.	13.264	9.263	8.475				
Std. Dev.	37.517	49.015	50.853				
95% C.I.	(-51.240; 11.490)	(-89.256; -51.244)	(-76.262; -41.849)				
Minimum	-81.0	-150.0	-150.0				
First quartile	-36.00	-97.50	-93.00				
Median	-23.50	-74.50	-58.50				
Third quartile	-5.00	-46.50	-27.50				
Maximum	51.0	38.0	51.0				
Week 20							
N	0	24	24				
Mean	-	-68.54	-68.54				
Std. Err.	-	10.121	10.121				
Std. Dev.	-	49.583	49.583				
95% C.I.	-	(-89.479; -47.605)	(-89.479; -47.605)				
Minimum	-	-158.0	-158.0				
First quartile	-	-106.50	-106.50				
Median	-	-71.50	-71.50				
Third quartile	-	-50.50	-50.50				
Maximum	-	49.0	49.0				
Week 24							
N	0	25	25				
Mean	-	-56.56	-56.56				
Std. Err.	-	12.014	12.014				
Std. Dev.	-	60.072	60.072				
95% C.I.	-	
(-81.357; -31.763)	(-81.357; -31.763)				
Minimum	-	-165.0	-165.0				
First quartile	-	-109.00	-109.00				
Median	-	-60.00	-60.00				
Third quartile	-	-22.00	-22.00				
Maximum	-	73.0	73.0				
Week 28							
N	0	6	6				
Mean	-	-41.67	-41.67				
Std. Err.	-	19.339	19.339				
Std. Dev.	-	47.369	47.369				
95% C.I.	-	
(-91.378; 8.045)	(-91.378; 8.045)				
Minimum	-	-94.0	-94.0				
First quartile	-	-90.00	-90.00				
Median	-	-45.00	-45.00				
Third quartile	-	3.00	3.00				
Maximum	-	21.0	21.0				
Week 36							
N	0	0	0				
Mean	-	-	-				
95% C.I.	-	-	-				
Minimum	-	-	-				
First quartile	-	-	-				
Median	-	-	-				
Third quartile	-	-	-				
Maximum	-	-	-				
Week 48							
N	0	0	0				
Mean	-	-	-				
95% C.I.	-	-	-				
Minimum	-	-	-				
First quartile	-	-	-				
Median	-	-	-				
Third quartile	-	-	-				
Maximum	-	-	-				
EOT							
N	34	32	66				
Mean	-68.21	-65.50	-66.89				
Std. Err.	8.321	9.122	6.114				
Std. Dev.	48.519	51.600	49.667				
95% C.I.	(-85.135; -51.277)	(-84.104; -46.896)	(-79.104; -54.684)				
Minimum	-174.0	-165.0	-174.0				
First quartile	-86.00	-108.50	-97.00				
Median	-65.50	-67.50	-66.00				
Third quartile	-50.00	-40.00	-46.00				
Maximum	26.0	73.0	73.0				
Direct Bilirubin (umol/L)							
Week 01							
N	33	29	62				
Mean	1.64	1.76	1.69				
Std. Err.	0.301	0.220	0.189				
Std. Dev.	1.729	1.185	1.489				
95% C.I.	(1.023; 2.249)	(1.308; 2.209)	(1.316; 2.072)				
Minimum	-2.0	0.0	-2.0				
First quartile	1.00	1.00	1.00				
Median	1.00	2.00	1.50				
Third quartile	2.00	3.00	3.00				
Maximum	6.0	4.0	6.0				
Week 02							
N	31	29	60				
Mean	2.10	3.00	2.53				
Std. Err.	0.243	0.354	0.218				
Std. Dev.	1.350	1.909	1.692				
95% C.I.	(1.601; 2.592)	(2.274; 3.726)	(2.096; 2.970)				
Minimum	-2.0	0.0	-2.0				
First quartile	1.00	2.00	1.50				
Median	2.00	3.00	2.00				
Third quartile	3.00	4.00	3.00				
Maximum	4.0	8.0	8.0				
Week 04							
N	32	31	63				
Mean	1.69	2.58	2.13				
Std. Err.	0.226	0.401	0.234				
Std. Dev.	1.281	2.233	1.853				
95% C.I.	(1.226; 2.149)	(1.762; 3.400)	(1.660; 2.594)				
Minimum	-2.0	0.0	-2.0				
First quartile	1.00	1.00	1.00				
Median	2.00	2.00	2.00				
Third quartile	2.00	4.00	3.00				
Maximum	6.0	10.0	10.0				
Week 08							
N	33	29	62				
Mean	1.91	3.10	2.47				
Std. Err.	0.309	0.567	0.318				
Std. Dev.	1.774	3.051	2.507				
95% C.I.	(1.280; 2.538)	(1.943; 4.264)	(1.831; 3.104)				
Minimum	-2.0	0.0	-2.0				
First quartile	1.00	1.00	1.00				
Median	2.00	2.00	2.00				
Third quartile	3.00	4.00	3.00				
Maximum	8.0	13.0	13.0				
Week 12							
N	33	28	61				
Mean	1.91	2.54	2.20				
Std. Err.	0.300	0.543	0.297				
Std. Dev.	1.721	2.874	2.322				
95% C.I.	(1.299; 2.519)	(1.421; 3.650)	(1.602; 2.792)				
Minimum	-2.0	-2.0	-2.0				
First quartile	1.00	1.00	1.00				
Median	2.00	2.00	2.00				
Third quartile	3.00	3.00	3.00				
Maximum	6.0	11.0	11.0				
Week 16							
N	8	27	35				
Mean	-0.88	0.07	-0.14				
Std. Err.	0.398	0.302	0.256				
Std. Dev.	1.126	1.567	1.517				
95% C.I.	(-1.816; 0.066)	(-0.546; 0.694)	(-0.664; 0.378)				
Minimum	-3.0	-3.0	-3.0				
First quartile	-1.50	-1.00	-1.00				
Median	-0.50	0.00	0.00				
Third quartile	0.00	1.00	0.00				
Maximum	0.0	4.0	4.0				
Week 20							
N	0	25	25				
Mean	-	-0.12	-0.12				
Std. Err.	-	0.273	0.273				
Std. Dev.	-	1.364	1.364				
95% C.I.	-	(-0.683; 0.443)	(-0.683; 0.443)				
Minimum	-	-3.0	-3.0				
First quartile	-	-1.00	-1.00				
Median	-	0.00	0.00				
Third quartile	-	1.00	1.00				
Maximum	-	3.0	3.0				
Week 24							
N	0	26	26				
Mean	-	-0.15	-0.15				
Std. Err.	-	0.246	0.246				
Std. Dev.	-	1.255	1.255				
95% C.I.	-	(-0.661; 0.353)	(-0.661; 0.353)				
Minimum	-	-3.0	-3.0				
First quartile	-	-1.00	-1.00				
Median	-	0.00	0.00				
Third quartile	-	1.00	1.00				
Maximum	-	2.0	2.0				
Week 28							
N	0	6	6				
Mean	-	-1.17	-1.17				
Std. Err.	-	0.477	0.477				
Std. Dev.	-	1.169	1.169				
95% C.I.	-	(-2.394; 0.060)	(-2.394; 0.060)				
Minimum	-	-3.0	-3.0				
First quartile	-	-2.00	-2.00				
Median	-	-1.00	-1.00				
Third quartile	-	0.00	0.00				
Maximum	-	0.0	0.0				
Week 36							
N	0	0	0				
Mean	-	-	-				
95% C.I.	-	-	-				
Minimum	-	-	-				
First quartile	-	-	-				
Median	-	-	-				
Third quartile	-	-	-				
Maximum	-	-	-				
Week 48							
N	0	0	0				
Mean	-	-	-				
95% C.I.	-	-	-				
Minimum	-	-	-				
First quartile	-	-	-				
Median	-	-	-				
Third quartile	-	-	-				
Maximum	-	-	-				
EOT							
N	34	32	66				
Mean	2.03	0.25	1.17				
Std. Err.	0.285	0.266	0.223				
Std. Dev.	1.660	1.503	1.811				
95% C.I.	(1.450; 2.609)	(-0.292; 0.792)	(0.721; 1.612)				
Minimum	-2.0	-3.0	-3.0				
First quartile	1.00	-1.00	0.00				
Median	2.00	0.00	1.00				
Third quartile	3.00	1.00	2.00				
Maximum	6.0	4.0	6.0				
Indirect Bilirubin (umol/L)							
Week 01							
N	33	29	62				
Mean	5.82	7.59	6.65				
Std. Err.	1.207	2.203	1.209				
Std. Dev.	6.935	11.864	9.520				
95% C.I.	(3.359; 8.277)	(3.073; 12.099)	(4.228; 9.063)				
Minimum	-3.0	-2.0	-3.0				
First quartile	1.00	2.00	1.00				
Median	3.00	4.00	3.00				
Third quartile	11.00	9.00	10.00				
Maximum	20.0	49.0	49.0				
Week 02							
N	31	29	60				
Mean	4.52	8.38	6.38				
Std. Err.	0.820	1.572	0.898				
Std. Dev.	4.567	8.466	6.958				
95% C.I.	(2.841; 6.191)	(5.159; 11.600)	(4.586; 8.181)				
Minimum	-6.0	-1.0	-6.0				
First quartile	2.00	4.00	2.50				
Median	5.00	7.00	5.00				
Third quartile	7.00	11.00	9.00				
Maximum	16.0	44.0	44.0				
Week 04							
N	32	31	63				
Mean	4.38	6.74	5.54				
Std. Err.	0.916	1.413	0.843				
Std. Dev.	5.179	7.870	6.693				
95% C.I.	(2.508; 6.242)	(3.855; 9.629)	(3.854; 7.225)				
Minimum	-3.0	-1.0	-3.0				
First quartile	1.00	2.00	1.00				
Median	4.00	5.00	4.00				
Third quartile	6.50	9.00	8.00				
Maximum	26.0	40.0	40.0				
Week 08							
N	33	29	62				
Mean	5.12	6.17	5.61				
Std. Err.	0.945	1.313	0.790				
Std. Dev.	5.430	7.071	6.221				
95% C.I.	(3.196; 7.047)	(3.483; 8.862)	(4.033; 7.193)				
Minimum	-5.0	-1.0	-5.0				
First quartile	2.00	2.00	2.00				
Median	4.00	5.00	4.50				
Third quartile	7.00	7.00	7.00				
Maximum	21.0	30.0	30.0				
Week 12							
N	33	28	61				
Mean	4.18	4.89	4.51				
Std. Err.	0.939	0.940	0.663				
Std. Dev.	5.394	4.977	5.176				
95% C.I.	(2.269; 6.094)	(2.963; 6.823)	(3.183; 5.834)				
Minimum	-4.0	-6.0	-6.0				
First quartile	2.00	2.00	2.00				
Median	3.00	3.00	3.00				
Third quartile	5.00	7.00	7.00				
Maximum	22.0	16.0	22.0				
Week 16							
N	8	27	35				
Mean	-2.38	0.22	-0.37				
Std. Err.	1.295	0.650	0.603				
Std. Dev.	3.662	3.378	3.565				
95% C.I.	(-5.437; 0.687)	(-1.114; 1.558)	(-1.596; 0.853)				
Minimum	-11.0	-12.0	-12.0				
First quartile	-2.50	-1.00	-1.00				
Median	-1.50	0.00	0.00				
Third quartile	0.00	2.00	2.00				
Maximum	0.0	6.0	6.0				
Week 20							
N	0	25	25				
Mean	-	-0.24	-0.24				
Std. Err.	-	0.839	0.839				
Std. Dev.	-	4.196	4.196				
95% C.I.	-	(-1.972; 1.492)	(-1.972; 1.492)				
Minimum	-	-14.0	-14.0				
First quartile	-	-2.00	-2.00				
Median	-	-1.00	-1.00				
Third quartile	-	1.00	1.00				
Maximum	-	11.0	11.0				
Week 24							
N	0	26	26				
Mean	-	-0.54	-0.54				
Std. Err.	-	0.555	0.555				
Std. Dev.	-	2.832	2.832				
95% C.I.	-	(-1.682; 0.605)	(-1.682; 0.605)				
Minimum	-	-9.0	-9.0				
First quartile	-	-2.00	-2.00				
Median	-	-0.50	-0.50				
Third quartile	-	0.00	0.00				
Maximum	-	6.0	6.0				
Week 28							
N	0	6	6				
Mean	-	-4.00	-4.00				
Std. Err.	-	2.206	2.206				
Std. Dev.	-	5.404	5.404				
95% C.I.	-	(-9.671; 1.671)	(-9.671; 1.671)				
Minimum	-	-14.0	-14.0				
First quartile	-	-6.00	-6.00				
Median	-	-2.00	-2.00				
Third quartile	-	-1.00	-1.00				
Maximum	-	1.0	1.0				
Week 36							
N	0	0	0				
Mean	-	-	-				
95% C.I.	-	-	-				
Minimum	-	-	-				
First quartile	-	-	-				
Median	-	-	-				
Third quartile	-	-	-				
Maximum	-	-	-				
Week 48							
N	0	0	0				
Mean	-	-	-				
95% C.I.	-	-	-				
Minimum	-	-	-				
First quartile	-	-	-				
Median	-	-	-				
Third quartile	-	-	-				
Maximum	-	-	-				
EOT							
N	34	32	66				
Mean	4.82	0.75	2.85				
Std. Err.	1.009	0.746	0.677				
Std. Dev.	5.885	4.220	5.503				
95% C.I.	(2.770; 6.877)	(-0.771; 2.271)	(1.496; 4.201)				
Minimum	-4.0	-9.0	-9.0				
First quartile	2.00	-1.50	-1.00				
Median	3.50	0.00	2.00				
Third quartile	6.00	2.50	5.00				
Maximum	22.0	14.0	22.0				
Bilirubin (umol/L)							
Week 01							
N	33	30	63				
Mean	7.45	9.50	8.43				
Std. Err.	1.416	2.233	1.292				
Std. Dev.	8.136	12.233	10.258				
95% C.I.	(4.570; 10.339)	(4.932; 14.068)	(5.845; 11.012)				
Minimum	-5.0	-2.0	-5.0				
First quartile	1.00	2.00	2.00				
Median	4.00	6.00	5.00				
Third quartile	13.00	11.00	13.00				
Maximum	24.0	52.0	52.0				
Week 02							
N	31	30	61				
Mean	6.61	11.70	9.11				
Std. Err.	1.010	1.678	1.018				
Std. Dev.	5.625	9.192	7.952				
95% C.I.	(4.549; 8.676)	(8.268; 15.132)	(7.078; 11.151)				
Minimum	-6.0	-1.0	-6.0				
First quartile	3.00	6.00	4.00				
Median	7.00	10.00	8.00				
Third quartile	10.00	15.00	12.00				
Maximum	20.0	47.0	47.0				
Week 04							
N	33	32	65				
Mean	6.18	9.44	7.78				
Std. Err.	1.042	1.566	0.950				
Std. Dev.	5.987	8.861	7.658				
95% C.I.	(4.059; 8.305)	(6.243; 12.632)	(5.887; 9.682)				
Minimum	-5.0	-1.0	-5.0				
First quartile	2.00	3.50	3.00				
Median	6.00	7.50	6.00				
Third quartile	10.00	12.00	10.00				
Maximum	29.0	43.0	43.0				
Week 08							
N	33	30	63				
Mean	7.03	9.67	8.29				
Std. Err.	1.183	1.632	1.000				
Std. Dev.	6.794	8.938	7.934				
95% C.I.	(4.621; 9.439)	(6.329; 13.004)	(6.288; 10.284)				
Minimum	-6.0	0.0	-6.0				
First quartile	2.00	4.00	4.00				
Median	7.00	7.00	7.00				
Third quartile	9.00	14.00	10.00				
Maximum	25.0	35.0	35.0				
Week 12							
N	33	28	61				
Mean	6.09	7.64	6.80				
Std. Err.	1.146	1.287	0.855				
Std. Dev.	6.583	6.811	6.678				
95% C.I.	(3.757; 8.425)	(5.002; 10.284)	(5.093; 8.514)				
Minimum	-5.0	-4.0	-5.0				
First quartile	2.00	3.00	3.00				
Median	5.00	6.00	6.00				
Third quartile	9.00	10.00	10.00				
Maximum	25.0	24.0	25.0				
Week 16							
N	8	28	36				
Mean	-3.25	0.64	-0.22				
Std. Err.	1.623	0.814	0.768				
Std. Dev.	4.590	4.305	4.605				
95% C.I.	(-7.088; 0.588)	(-1.027; 2.312)	(-1.780; 1.336)				
Minimum	-14.0	-13.0	-14.0				
First quartile	-3.50	-2.00	-2.00				
Median	-2.00	0.00	0.00				
Third quartile	-0.50	3.00	1.50				
Maximum	0.0	8.0	8.0				
Week 20							
N	0	26	26				
Mean	-	-0.04	-0.04				
Std. Err.	-	1.041	1.041				
Std. Dev.	-	5.310	5.310				
95% C.I.	-	(-2.183; 2.106)	(-2.183; 2.106)				
Minimum	-	-16.0	-16.0				
First quartile	-	-3.00	-3.00				
Median	-	-1.00	-1.00				
Third quartile	-	3.00	3.00				
Maximum	-	14.0	14.0				
Week 24							
N	0	26	26				
Mean	-	-0.62	-0.62				
Std. Err.	-	0.732	0.732				
Std. Dev.	-	3.732	3.732				
95% C.I.	-	(-2.123; 0.892)	(-2.123; 0.892)				
Minimum	-	-10.0	-10.0				
First quartile	-	-3.00	-3.00				
Median	-	-1.00	-1.00				
Third quartile	-	1.00	1.00				
Maximum	-	7.0	7.0				
Week 28							
N	0	6	6				
Mean	-	-5.17	-5.17				
Std. Err.	-	2.774	2.774				
Std. Dev.	-	6.795	6.795				
95% C.I.	-	(-12.297; 1.964)	(-12.297; 1.964)				
Minimum	-	-17.0	-17.0				
First quartile	-	-8.00	-8.00				
Median	-	-3.50	-3.50				
Third quartile	-	-2.00	-2.00				
Maximum	-	3.0	3.0				
Week 36							
N	0	0	0				
Mean	-	-	-				
95% C.I.	-	-	-				
Minimum	-	-	-				
First quartile	-	-	-				
Median	-	-	-				
Third quartile	-	-	-				
Maximum	-	-	-				
Week 48							
N	0	0	0				
Mean	-	-	-				
95% C.I.	-	-	-				
Minimum	-	-	-				
First quartile	-	-	-				
Median	-	-	-				
Third quartile	-	-	-				
Maximum	-	-	-				
EOT							
N	34	32	66				
Mean	6.85	1.19	4.11				
Std. Err.	1.201	1.042	0.867				
Std. Dev.	7.003	5.894	7.043				
95% C.I.	(4.410; 9.296)	(-0.937; 3.312)	(2.375; 5.837)				
Minimum	-3.0	-10.0	-10.0				
First quartile	2.00	-3.00	-1.00				
Median	5.50	0.00	3.00				
Third quartile	10.00	4.00	7.00				
Maximum	25.0	22.0	25.0				
	
[TSFLAB02-GT.RTF] [TMC435\HPC3014\DBR_FINAL_ANALYSIS\RE_FINAL_ANALYSIS\PROD\TSFLAB02-GT.SAS] 02NOV2015, 12:10	
