# Supplementary material for: Efficacy of a 12-Week Simeprevir Plus Peginterferon/Ribavirin (PR) Regimen in Treatment-Naïve Patients with Hepatitis C Virus (HCV) Genotype 4 (GT4) Infection and Mild-To-Moderate Fibrosis Displaying Early On-Treatment Virologic Response
Source: PLoS One. 2017 Jan 5;12(1):e0168713. doi: 10.1371/journal.pone.0168713 (PMC5215882; doi:10.1371/journal.pone.0168713)
Supplement: S1 Dataset — (ZIP) [file pone.0168713.s002.zip › TSIDEM01.rtf]

TSIDEM01:	Demographics parameters	
	Simeprevir
12 Wks
150 mg
PR 12/24 	
	 Genotype 4 	
	 12 Wks 	 >12 Wks 	
Analysis Set: Intent-to-treat	34	33	
Gender			
N	34	33	
F	11 (32.4%)	10 (30.3%)	
M	23 (67.6%)	23 (69.7%)	
Overall p-value (Chi-squre)		0.856	
Age (years)			
N	34	33	
Mean	43.6	46.4	
Std. Err.	2.38	1.95	
Std. Dev.	13.87	11.19	
95% C.I.	(38.75; 48.43)	(42.42; 50.36)	
Minimum	19	21	
First quartile	31.0	42.0	
Median	47.5	48.0	
Third quartile	54.0	55.0	
Maximum	63	66	
P-value (Wilcoxon-Mann-Whitney)		0.585	
Age (years)			
N	34	33	
45	16 (47.1%)	14 (42.4%)	
>45 - 65	18 (52.9%)	18 (54.5%)	
>65		1 (3.0%)	
Overall p-value (Chi-squre)		0.572	
Race			
Not allowed to ask per local regulations	4	4	
N	30	29	
Asian	2 (6.7%)	1 (3.4%)	
Black or African American	3 (10.0%)	4 (13.8%)	
Native Hawaiian or Other Pacific Islander			
White	23 (76.7%)	24 (82.8%)	
American Indian or Alaska Native			
Multiple	2 (6.7%)		
Overall p-value (Chi-squre)		0.479	
Ethnicity			
Not allowed to ask per local regulations	4	4	
N	30	29	
Hispanic or Latino		1 (3.4%)	
Not Hispanic or Latino	30 (100.0%)	28 (96.6%)	
Overall p-value (Chi-squre)		0.305	
Region			
N	34	33	
EUROPE	24 (70.6%)	26 (78.8%)	
MIDDLE-EAST/NORTH-AFRICA	10 (29.4%)	7 (21.2%)	
Overall p-value (Chi-squre)		0.441	
Country			
N	34	33	
Austria	3 (8.8%)	5 (15.2%)	
Belgium	3 (8.8%)	5 (15.2%)	
France	6 (17.6%)	7 (21.2%)	
Germany			
Italy	4 (11.8%)	4 (12.1%)	
Saudi Arabia	10 (29.4%)	7 (21.2%)	
Spain	8 (23.5%)	5 (15.2%)	
United Kingdom			
Overall p-value (Chi-squre)		0.809	
Origin			
N	34	33	
Europe	16 (47.1%)	16 (48.5%)	
Middle-East/North-Africa	7 (20.6%)	8 (24.2%)	
Other regions	1 (2.9%)		
Overall p-value (Chi-squre)		0.587	
Body weight (kg)			
N	34	33	
Mean	72.76	79.04	
Std. Err.	2.371	2.225	
Std. Dev.	13.824	12.781	
95% C.I.	(67.938; 77.585)	(74.511; 83.574)	
Minimum	52.0	55.0	
First quartile	59.60	69.00	
Median	73.75	78.40	
Third quartile	80.00	88.00	
Maximum	109.0	107.0	
Body Mass Index (kg/m2)			
N	34	33	
Mean	25.00	27.68	
Std. Err.	0.696	0.675	
Std. Dev.	4.056	3.878	
95% C.I.	(23.585; 26.415)	(26.301; 29.051)	
Minimum	18.4	19.0	
First quartile	22.10	24.80	
Median	24.60	27.50	
Third quartile	27.50	30.40	
Maximum	32.9	35.8	
P-value (Wilcoxon-Mann-Whitney)		0.01	
Body Mass Index (kg/m2)			
N	34	33	
<25	18 (52.9%)	9 (27.3%)	
25 - <30	12 (35.3%)	12 (36.4%)	
30	4 (11.8%)	12 (36.4%)	
Overall p-value (Chi-squre)		0.03	

P-value s alculated o est istribution f aseline arameters etween reatment urations
Chi-Square est s sed or ategorical arameters nd ilcoxon-Mann-Whitney est s sed or ontinuous arameters	
[TSIDEM01.RTF] [TMC435\HPC3014\DBR_FINAL_ANALYSIS\RE_FINAL_ANALYSIS\PDEV\TEMPFILE.SAS] 12OCT2016, 09:47	
