# Supplementary material for: Efficacy of a 12-Week Simeprevir Plus Peginterferon/Ribavirin (PR) Regimen in Treatment-Naïve Patients with Hepatitis C Virus (HCV) Genotype 4 (GT4) Infection and Mild-To-Moderate Fibrosis Displaying Early On-Treatment Virologic Response
Source: PLoS One. 2017 Jan 5;12(1):e0168713. doi: 10.1371/journal.pone.0168713 (PMC5215882; doi:10.1371/journal.pone.0168713)
Supplement: S1 Dataset — (ZIP) [file pone.0168713.s002.zip › TSIDEM01A.rtf]

TSIDEM01A:	Demographic Characteristics; Intent-to-treat (Study TMC435HPC3014)
Treatment Group = Simeprevir 12Wks 150 mg PR12/24	
	Genotype 4			
	12 Weeks 
Treatment	>12 Weeks 
Treatment	All Subjects					
Analysis set: intent-to-treat	34	33	67					
	
Gender								
N	34	33	67					
Female	11 (32.4%)	10 (30.3%)	21 (31.3%)					
Male	23 (67.6%)	23 (69.7%)	46 (68.7%)					
	
Age (years)								
N	34	33	67					
Mean (SD)	43.6 (13.87)	46.4 (11.19)	45.0 (12.60)					
Median	47.5	48.0	48.0					
Range	(19; 63)	(21; 66)	(19; 66)					
	
Age (years)								
N	34	33	67					
≤45 years	16 (47.1%)	14 (42.4%)	30 (44.8%)					
>45 - ≤65 years	18 (52.9%)	18 (54.5%)	36 (53.7%)					
>65 years	0	1 (3.0%)	1 (1.5%)					
	
Race								
Not allowed to ask per local regulations	4	4	8					
N	30	29	59					
Asian	2 (6.7%)	1 (3.4%)	3 (5.1%)					
Black or African American	3 (10.0%)	4 (13.8%)	7 (11.9%)					
Multiple	2 (6.7%)	0	2 (3.4%)					
Native Hawaiian or Other Pacific Islander	0	0	0					
White	23 (76.7%)	24 (82.8%)	47 (79.7%)					
	
Ethnicity								
Not allowed to ask per local regulations	4	4	8					
N	30	29	59					
Hispanic or Latino	0	1 (3.4%)	1 (1.7%)					
Not Hispanic or Latino	30 (100.0%)	28 (96.6%)	58 (98.3%)					
	
Region								
N	34	33	67					
Europe	
24 (70.6%)	26 (78.8%)	50 (74.6%)					
Middle-east/North-africa	10 (29.4%)	7 (21.2%)	17 (25.4%)					
	
Country								
N	34	33	67					
Austria	3 (8.8%)	5 (15.2%)	8 (11.9%)					
Belgium	3 (8.8%)	5 (15.2%)	8 (11.9%)					
France	6 (17.6%)	7 (21.2%)	13 (19.4%)					
Germany	0	0	0					
Italy	4 (11.8%)	4 (12.1%)	8 (11.9%)					
Saudi Arabia	10 (29.4%)	7 (21.2%)	17 (25.4%)					
Spain	8 (23.5%)	5 (15.2%)	13 (19.4%)					
United Kingdom	0	0	0					
	
Origin								
N	24	24	48					
Europe	16 (66.7%)	16 (66.7%)	32 (66.7%)					
Middle-East/North-Africa	7 (29.2%)	8 (33.3%)	15 (31.3%)					
Other regions	1 (4.2%)	0	1 (2.1%)					
	
Body weight (kg)								
N	34	33	67					
Mean (SD)	72.76 (13.824)	79.04 (12.781)	75.86 (13.593)					
Median	73.75	78.40	76.20					
Range	(52.0; 109.0)	(55.0; 107.0)	(52.0; 109.0)					
	
Body mass index (kg/m²)								
N	34	33	67					
Mean (SD)	25.00 (4.056)	27.68 (3.878)	26.32 (4.163)					
Median	24.60	27.50	26.30					
Range	(18.4; 32.9)	(19.0; 35.8)	(18.4; 35.8)					
	
Body mass index (kg/m²)								
N	34	33	67					
<25 kg/m²	18 (52.9%)	9 (27.3%)	27 (40.3%)					
≥25 - <30 kg/m²	12 (35.3%)	12 (36.4%)	24 (35.8%)					
≥30 kg/m²	4 (11.8%)	12 (36.4%)	16 (23.9%)					
	

N = number of subjects with data	
[TSIDEM01A.rtf] [\STAT\Analyses\Programs\FinalAnalysis\Final1\2.TLF\1.General\GEN_FA.sas] 23OCT2015, 16:53	
