# Supplementary material for: Efficacy of a 12-Week Simeprevir Plus Peginterferon/Ribavirin (PR) Regimen in Treatment-Naïve Patients with Hepatitis C Virus (HCV) Genotype 4 (GT4) Infection and Mild-To-Moderate Fibrosis Displaying Early On-Treatment Virologic Response
Source: PLoS One. 2017 Jan 5;12(1):e0168713. doi: 10.1371/journal.pone.0168713 (PMC5215882; doi:10.1371/journal.pone.0168713)
Supplement: S1 Dataset — (ZIP) [file pone.0168713.s002.zip › TSIDEM01B.rtf]

TSIDEM01B:	Demographic Characteristics by Subgroups of Interest; Intent-to-treat (Study TMC435HPC3014)
Treatment Group = Simeprevir 12Wks 150 mg PR12/24 
HCV Geno/Subtype = 4a	
	Genotype 4		
	12 Weeks 
Treatment	>12 Weeks 
Treatment	All Subjects		
Analysis set: intent-to-treat	14	13	27		
	
Gender					
N	14	13	27		
Female	4 (28.6%)	2 (15.4%)	6 (22.2%)		
Male	10 (71.4%)	11 (84.6%)	21 (77.8%)		
	
Age (years)					
N	14	13	27		
Mean	45.6	44.9	45.3		
SE	3.89	3.04	2.45		
SD	14.57	10.96	12.72		
95% C.I. *	(37.23; 54.05)	(38.30; 51.54)	(40.27; 50.33)		
Min	19	28	19		
Q1	36.0	42.0	36.0		
Median	50.0	45.0	48.0		
Q3	58.0	53.0	56.0		
Max	63	59	63		
	
Age (years)					
N	14	13	27		
≤45 years	5 (35.7%)	7 (53.8%)	12 (44.4%)		
>45 - ≤65 years	9 (64.3%)	6 (46.2%)	15 (55.6%)		
	
Race					
Not allowed to ask per local regulations	4	2	6		
N	10	11	21		
Asian	1 (10.0%)	0	1 (4.8%)		
Multiple	2 (20.0%)	0	2 (9.5%)		
White	7 (70.0%)	11 (100.0%)	18 (85.7%)		
	
Ethnicity					
Not allowed to ask per local regulations	4	2	6		
N	10	11	21		
Hispanic or Latino	0	1 (9.1%)	1 (4.8%)		
Not Hispanic or Latino	10 (100.0%)	10 (90.9%)	20 (95.2%)		
	
Region					
N	14	13	27		
Europe	8 (57.1%)	10 (76.9%)	18 (66.7%)		
Middle-east/North-africa	6 (42.9%)	3 (23.1%)	9 (33.3%)		
	
Country					
N	14	13	27		
Austria	2 (14.3%)	4 (30.8%)	6 (22.2%)		
Belgium	1 (7.1%)	1 (7.7%)	2 (7.4%)		
France	4 (28.6%)	3 (23.1%)	7 (25.9%)		
Italy	0	1 (7.7%)	1 (3.7%)		
Saudi Arabia	6 (42.9%)	3 (23.1%)	9 (33.3%)		
Spain	1 (7.1%)	1 (7.7%)	2 (7.4%)		
	
Origin					
N	8	11	19		
Europe	3 (37.5%)	6 (54.5%)	9 (47.4%)		
Middle-East/North-Africa	5 (62.5%)	5 (45.5%)	10 (52.6%)		
	
Body weight (kg)					
N	14	13	27		
Mean	73.05	76.29	74.61		
SE	3.291	3.622	2.413		
SD	12.315	13.059	12.540		
95% C.I. *	(65.940; 80.160)	(68.401; 84.184)	(69.650; 79.572)		
Min	52.0	55.0	52.0		
Q1	60.00	68.00	65.20		
Median	77.00	80.90	79.00		
Q3	81.00	84.00	84.00		
Max	89.0	97.0	97.0		
	
Body mass index (kg/m²)					
N	14	13	27		
Mean	25.34	26.46	25.88		
SE	1.158	1.346	0.873		
SD	4.332	4.853	4.536		
95% C.I. *	(22.841; 27.844)	(23.529; 29.394)	(24.087; 27.676)		
Min	18.4	19.0	18.4		
Q1	23.00	23.40	23.00		
Median	25.95	26.50	26.10		
Q3	27.50	30.20	29.40		
Max	32.9	35.8	35.8		
	
Body mass index (kg/m²)					
N	14	13	27		
<25 kg/m²	6 (42.9%)	5 (38.5%)	11 (40.7%)		
≥25 - <30 kg/m²	6 (42.9%)	4 (30.8%)	10 (37.0%)		
≥30 kg/m²	2 (14.3%)	4 (30.8%)	6 (22.2%)		
	

* Confidence interval for mean
N = number of subjects with data	
[TSIDEM01B.rtf] [\STAT\Analyses\Programs\FinalAnalysis\Final1\2.TLF\1.General\GEN_FA.sas] 23OCT2015, 16:53	

TSIDEM01B:	Demographic Characteristics by Subgroups of Interest; Intent-to-treat (Study TMC435HPC3014)
Treatment Group = Simeprevir 12Wks 150 mg PR12/24 
HCV Geno/Subtype = 4d	
	Genotype 4		
	12 Weeks 
Treatment	>12 Weeks 
Treatment	All Subjects		
Analysis set: intent-to-treat	13	12	25		
	
Gender					
N	13	12	25		
Female	5 (38.5%)	3 (25.0%)	8 (32.0%)		
Male	8 (61.5%)	9 (75.0%)	17 (68.0%)		
	
Age (years)					
N	13	12	25		
Mean	39.5	48.4	43.8		
SE	3.55	2.27	2.29		
SD	12.81	7.86	11.44		
95% C.I. *	(31.80; 47.28)	(43.43; 53.41)	(39.08; 48.52)		
Min	20	27	20		
Q1	31.0	47.5	32.0		
Median	45.0	49.5	48.0		
Q3	48.0	53.0	51.0		
Max	61	57	61		
	
Age (years)					
N	13	12	25		
≤45 years	8 (61.5%)	2 (16.7%)	10 (40.0%)		
>45 - ≤65 years	5 (38.5%)	10 (83.3%)	15 (60.0%)		
	
Race					
Not allowed to ask per local regulations	0	1	1		
N	13	11	24		
White	13 (100.0%)	11 (100.0%)	24 (100.0%)		
	
Ethnicity					
Not allowed to ask per local regulations	0	1	1		
N	13	11	24		
Not Hispanic or Latino	13 (100.0%)	11 (100.0%)	24 (100.0%)		
	
Region					
N	13	12	25		
Europe	10 (76.9%)	10 (83.3%)	20 (80.0%)		
Middle-east/North-africa	3 (23.1%)	2 (16.7%)	5 (20.0%)		
	
Country					
N	13	12	25		
Austria	0	1 (8.3%)	1 (4.0%)		
France	0	3 (25.0%)	3 (12.0%)		
Italy	4 (30.8%)	2 (16.7%)	6 (24.0%)		
Saudi Arabia	3 (23.1%)	2 (16.7%)	5 (20.0%)		
Spain	6 (46.2%)	4 (33.3%)	10 (40.0%)		
	
Origin					
N	13	11	24		
Europe	10 (76.9%)	9 (81.8%)	19 (79.2%)		
Middle-East/North-Africa	2 (15.4%)	2 (18.2%)	4 (16.7%)		
Other regions	1 (7.7%)	0	1 (4.2%)		
	
Body weight (kg)					
N	13	12	25		
Mean	65.59	81.90	73.42		
SE	2.150	3.862	2.692		
SD	7.753	13.378	13.462		
95% C.I. *	(60.907; 70.278)	(73.400; 90.400)	(67.863; 78.977)		
Min	54.0	65.3	54.0		
Q1	59.00	70.30	65.30		
Median	66.70	79.00	71.20		
Q3	71.20	91.05	78.00		
Max	77.0	107.0	107.0		
	
Body mass index (kg/m²)					
N	13	12	25		
Mean	22.65	28.01	25.22		
SE	0.573	0.839	0.734		
SD	2.065	2.907	3.669		
95% C.I. *	(21.406; 23.902)	(26.161; 29.855)	(23.710; 26.738)		
Min	19.7	23.8	19.7		
Q1	21.70	24.95	22.20		
Median	22.20	28.90	24.70		
Q3	23.60	30.35	28.70		
Max	26.4	31.9	31.9		
	
Body mass index (kg/m²)					
N	13	12	25		
<25 kg/m²	11 (84.6%)	3 (25.0%)	14 (56.0%)		
≥25 - <30 kg/m²	2 (15.4%)	5 (41.7%)	7 (28.0%)		
≥30 kg/m²	0	4 (33.3%)	4 (16.0%)		
	

* Confidence interval for mean
N = number of subjects with data	
[TSIDEM01B.rtf] [\STAT\Analyses\Programs\FinalAnalysis\Final1\2.TLF\1.General\GEN_FA.sas] 23OCT2015, 16:53	

TSIDEM01B:	Demographic Characteristics by Subgroups of Interest; Intent-to-treat (Study TMC435HPC3014)
Treatment Group = Simeprevir 12Wks 150 mg PR12/24 
HCV Geno/Subtype = 4other	
	Genotype 4		
	12 Weeks 
Treatment	>12 Weeks 
Treatment	All Subjects		
Analysis set: intent-to-treat	7	8	15		
	
Gender					
N	7	8	15		
Female	2 (28.6%)	5 (62.5%)	7 (46.7%)		
Male	5 (71.4%)	3 (37.5%)	8 (53.3%)		
	
Age (years)					
N	7	8	15		
Mean	47.0	45.8	46.3		
SE	5.48	5.68	3.83		
SD	14.50	16.08	14.83		
95% C.I. *	(33.59; 60.41)	(32.31; 59.19)	(38.12; 54.54)		
Min	27	21	21		
Q1	30.0	35.0	30.0		
Median	53.0	44.0	45.0		
Q3	61.0	60.5	61.0		
Max	63	66	66		
	
Age (years)					
N	7	8	15		
≤45 years	3 (42.9%)	5 (62.5%)	8 (53.3%)		
>45 - ≤65 years	4 (57.1%)	2 (25.0%)	6 (40.0%)		
>65 years	0	1 (12.5%)	1 (6.7%)		
	
Race					
Not allowed to ask per local regulations	0	1	1		
N	7	7	14		
Asian	1 (14.3%)	1 (14.3%)	2 (14.3%)		
Black or African American	3 (42.9%)	4 (57.1%)	7 (50.0%)		
White	3 (42.9%)	2 (28.6%)	5 (35.7%)		
	
Ethnicity					
Not allowed to ask per local regulations	0	1	1		
N	7	7	14		
Not Hispanic or Latino	7 (100.0%)	7 (100.0%)	14 (100.0%)		
	
Region					
N	7	8	15		
Europe	6 (85.7%)	6 (75.0%)	12 (80.0%)		
Middle-east/North-africa	1 (14.3%)	2 (25.0%)	3 (20.0%)		
	
Country					
N	7	8	15		
Austria	1 (14.3%)	0	1 (6.7%)		
Belgium	2 (28.6%)	4 (50.0%)	6 (40.0%)		
France	2 (28.6%)	1 (12.5%)	3 (20.0%)		
Italy	0	1 (12.5%)	1 (6.7%)		
Saudi Arabia	1 (14.3%)	2 (25.0%)	3 (20.0%)		
Spain	1 (14.3%)	0	1 (6.7%)		
	
Origin					
N	3	2	5		
Europe	3 (100.0%)	1 (50.0%)	4 (80.0%)		
Middle-East/North-Africa	0	1 (50.0%)	1 (20.0%)		
	
Body weight (kg)					
N	7	8	15		
Mean	85.50	79.23	82.15		
SE	6.562	4.258	3.761		
SD	17.361	12.043	14.567		
95% C.I. *	(69.444; 101.556)	(69.157; 89.293)	(74.087; 90.220)		
Min	58.0	64.0	58.0		
Q1	76.00	70.55	72.10		
Median	80.00	78.10	78.70		
Q3	98.80	85.75	98.00		
Max	109.0	101.0	109.0		
	
Body mass index (kg/m²)					
N	7	8	15		
Mean	28.67	29.15	28.93		
SE	1.381	1.100	0.841		
SD	3.654	3.110	3.259		
95% C.I. *	(25.292; 32.051)	(26.550; 31.750)	(27.122; 30.731)		
Min	22.1	24.4	22.1		
Q1	27.20	26.55	27.20		
Median	28.30	29.45	28.70		
Q3	32.60	32.10	32.40		
Max	32.9	32.6	32.9		
	
Body mass index (kg/m²)					
N	7	8	15		
<25 kg/m²	1 (14.3%)	1 (12.5%)	2 (13.3%)		
≥25 - <30 kg/m²	4 (57.1%)	3 (37.5%)	7 (46.7%)		
≥30 kg/m²	2 (28.6%)	4 (50.0%)	6 (40.0%)		
	

* Confidence interval for mean
N = number of subjects with data	
[TSIDEM01B.rtf] [\STAT\Analyses\Programs\FinalAnalysis\Final1\2.TLF\1.General\GEN_FA.sas] 23OCT2015, 16:53	

TSIDEM01B:	Demographic Characteristics by Subgroups of Interest; Intent-to-treat (Study TMC435HPC3014)
Treatment Group = Simeprevir 12Wks 150 mg PR12/24 
IL28b = CC	
	Genotype 4			
	12 Weeks 
Treatment	>12 Weeks 
Treatment	All Subjects					
Analysis set: intent-to-treat	14	1	15					
	
Gender								
N	14	1	15					
Female	6 (42.9%)	0	6 (40.0%)					
Male	8 (57.1%)	1 (100.0%)	9 (60.0%)					
	
Age (years)								
N	14	1	15					
Mean	41.8	28.0	40.9					
SE	3.62		3.49					
SD	13.55		13.53					
95% C.I. *	(33.96; 49.61)		(33.37; 48.36)					
Min	24		24					
Q1	27.0		27.0					
Median	43.0		41.0					
Q3	51.0		51.0					
Max	61		61					
	
Age (years)								
N	14	1	15					
≤45 years	8 (57.1%)	1 (100.0%)	9 (60.0%)					
>45 - ≤65 years	6 (42.9%)	0	6 (40.0%)					
	
Race								
Not allowed to ask per local regulations	1	0	1					
N	13	1	14					
Asian	0	0	0					
Black or African American	1 (7.7%)	0	1 (7.1%)					
Multiple	2 (15.4%)	0	2 (14.3%)					
White	10 (76.9%)	1 (100.0%)	11 (78.6%)					
	
Ethnicity								
Not allowed to ask per local regulations	1	0	1					
N	13	1	14					
Not Hispanic or Latino	13 (100.0%)	1 (100.0%)	14 (100.0%)					
	
Region								
N	14	1	15					
Europe	8 (57.1%)	0	8 (53.3%)					
Middle-east/North-africa	6 (42.9%)	1 (100.0%)	7 (46.7%)					
	
Country								
N	14	1	15					
Austria	0	0	0					
Belgium	0	0	0					
France	2 (14.3%)	0	2 (13.3%)					
Germany	0	0	0					
Italy	1 (7.1%)	0	1 (6.7%)					
Saudi Arabia	6 (42.9%)	1 (100.0%)	7 (46.7%)					
Spain	5 (35.7%)	0	5 (33.3%)					
United Kingdom	0	0	0					
	
Origin								
N	11	1	12					
Europe	6 (54.5%)	0	6 (50.0%)					
Middle-East/North-Africa	4 (36.4%)	1 (100.0%)	5 (41.7%)					
Other regions	1 (9.1%)	0	1 (8.3%)					
	
Body weight (kg)								
N	14	1	15					
Mean	71.37	71.80	71.40					
SE	2.781		2.589					
SD	10.404		10.026					
95% C.I. *	(65.364; 77.379)		(65.848; 76.952)					
Min	55.0		55.0					
Q1	59.60		59.60					
Median	75.00		73.00					
Q3	79.50		79.50					
Max	85.2		85.2					
	
Body mass index (kg/m²)								
N	14	1	15					
Mean	25.87	24.60	25.79					
SE	1.085		1.013					
SD	4.059		3.925					
95% C.I. *	(23.528; 28.215)		(23.613; 27.960)					
Min	21.2		21.2					
Q1	22.20		22.20					
Median	25.35		24.70					
Q3	27.50		27.50					
Max	32.9		32.9					
	
Body mass index (kg/m²)								
N	14	1	15					
<25 kg/m²	7 (50.0%)	1 (100.0%)	8 (53.3%)					
≥25 - <30 kg/m²	4 (28.6%)	0	4 (26.7%)					
≥30 kg/m²	3 (21.4%)	0	3 (20.0%)					
	

* Confidence interval for mean
N = number of subjects with data	
[TSIDEM01B.rtf] [\STAT\Analyses\Programs\FinalAnalysis\Final1\2.TLF\1.General\GEN_FA.sas] 23OCT2015, 16:53	

TSIDEM01B:	Demographic Characteristics by Subgroups of Interest; Intent-to-treat (Study TMC435HPC3014)
Treatment Group = Simeprevir 12Wks 150 mg PR12/24 
IL28b = CT	
	Genotype 4			
	12 Weeks 
Treatment	>12 Weeks 
Treatment	All Subjects	12 Weeks 
Treatment	>12 Weeks 
Treatment	All Subjects		
Analysis set: intent-to-treat	15	27	42					
	
Gender								
N	15	27	42					
Female	3 (20.0%)	9 (33.3%)	12 (28.6%)					
Male	12 (80.0%)	18 (66.7%)	30 (71.4%)					
	
Age (years)								
N	15	27	42					
Mean	44.7	47.1	46.2					
SE	3.58	2.13	1.85					
SD	13.87	11.05	12.02					
95% C.I. *	(37.05; 52.42)	(42.70; 51.44)	(42.49; 49.98)					
Min	20	21	20					
Q1	31.0	42.0	42.0					
Median	50.0	49.0	49.0					
Q3	54.0	55.0	55.0					
Max	63	66	66					
	
Age (years)								
N	15	27	42					
≤45 years	5 (33.3%)	11 (40.7%)	16 (38.1%)					
>45 - ≤65 years	10 (66.7%)	15 (55.6%)	25 (59.5%)					
>65 years	0	1 (3.7%)	1 (2.4%)					
	
Race								
Not allowed to ask per local regulations	2	4	6					
N	13	23	36					
Asian	2 (15.4%)	1 (4.3%)	3 (8.3%)					
Black or African American	1 (7.7%)	3 (13.0%)	4 (11.1%)					
Native Hawaiian or Other Pacific Islander	0	0	0					
White	10 (76.9%)	19 (82.6%)	29 (80.6%)					
	
Ethnicity								
Not allowed to ask per local regulations	2	4	6					
N	13	23	36					
Hispanic or Latino	0	1 (4.3%)	1 (2.8%)					
Not Hispanic or Latino	13 (100.0%)	22 (95.7%)	35 (97.2%)					
	
Region								
N	15	27	42					
Europe	12 (80.0%)	21 (77.8%)	33 (78.6%)					
Middle-east/North-africa	3 (20.0%)	6 (22.2%)	9 (21.4%)					
	
Country								
N	15	27	42					
Austria	2 (13.3%)	4 (14.8%)	6 (14.3%)					
Belgium	3 (20.0%)	4 (14.8%)	7 (16.7%)					
France	2 (13.3%)	5 (18.5%)	7 (16.7%)					
Germany	0	0	0					
Italy	3 (20.0%)	4 (14.8%)	7 (16.7%)					
Saudi Arabia	3 (20.0%)	6 (22.2%)	9 (21.4%)					
Spain	2 (13.3%)	4 (14.8%)	6 (14.3%)					
United Kingdom	0	0	0					
	
Origin								
N	10	19	29					
Europe	8 (80.0%)	12 (63.2%)	20 (69.0%)					
Middle-East/North-Africa	2 (20.0%)	7 (36.8%)	9 (31.0%)					
	
Body weight (kg)								
N	15	27	42					
Mean	75.88	79.02	77.90					
SE	4.503	2.157	2.103					
SD	17.441	11.207	13.632					
95% C.I. *	(66.222; 85.538)	(74.589; 83.455)	(73.652; 82.148)					
Min	52.0	55.0	52.0					
Q1	59.00	69.00	68.00					
Median	75.00	78.40	77.40					
Q3	89.00	88.00	88.00					
Max	109.0	101.0	109.0					
	
Body mass index (kg/m²)								
N	15	27	42					
Mean	24.71	27.73	26.65					
SE	1.151	0.711	0.647					
SD	4.458	3.694	4.193					
95% C.I. *	(22.244; 27.182)	(26.268; 29.191)	(25.346; 27.959)					
Min	18.4	21.0	18.4					
Q1	19.80	24.80	23.80					
Median	24.50	27.50	27.20					
Q3	28.30	30.40	29.60					
Max	32.6	35.8	35.8					
	
Body mass index (kg/m²)								
N	15	27	42					
<25 kg/m²	8 (53.3%)	7 (25.9%)	15 (35.7%)					
≥25 - <30 kg/m²	6 (40.0%)	11 (40.7%)	17 (40.5%)					
≥30 kg/m²	1 (6.7%)	9 (33.3%)	10 (23.8%)					
	

* Confidence interval for mean
N = number of subjects with data	
[TSIDEM01B.rtf] [\STAT\Analyses\Programs\FinalAnalysis\Final1\2.TLF\1.General\GEN_FA.sas] 23OCT2015, 16:53	

TSIDEM01B:	Demographic Characteristics by Subgroups of Interest; Intent-to-treat (Study TMC435HPC3014)
Treatment Group = Simeprevir 12Wks 150 mg PR12/24 
IL28b = TT	
	Genotype 4			
	12 Weeks 
Treatment	>12 Weeks 
Treatment	All Subjects					
Analysis set: intent-to-treat	5	5	10					
	
Gender								
N	5	5	10					
Female	2 (40.0%)	1 (20.0%)	3 (30.0%)					
Male	3 (60.0%)	4 (80.0%)	7 (70.0%)					
	
Age (years)								
N	5	5	10					
Mean	45.2	46.4	45.8					
SE	7.70	4.94	4.31					
SD	17.21	11.04	13.64					
95% C.I. *	(23.83; 66.57)	(32.70; 60.10)	(36.04; 55.56)					
Min	19	29	19					
Q1	41.0	45.0	41.0					
Median	45.0	48.0	46.5					
Q3	58.0	51.0	58.0					
Max	63	59	63					
	
Age (years)								
N	5	5	10					
≤45 years	3 (60.0%)	2 (40.0%)	5 (50.0%)					
>45 - ≤65 years	2 (40.0%)	3 (60.0%)	5 (50.0%)					
	
Race								
Not allowed to ask per local regulations	1	0	1					
N	4	5	9					
Black or African American	1 (25.0%)	1 (20.0%)	2 (22.2%)					
White	3 (75.0%)	4 (80.0%)	7 (77.8%)					
	
Ethnicity								
Not allowed to ask per local regulations	1	0	1					
N	4	5	9					
Not Hispanic or Latino	4 (100.0%)	5 (100.0%)	9 (100.0%)					
	
Region								
N	5	5	10					
Europe	4 (80.0%)	5 (100.0%)	9 (90.0%)					
Middle-east/North-africa	1 (20.0%)	0	1 (10.0%)					
	
Country								
N	5	5	10					
Austria	1 (20.0%)	1 (20.0%)	2 (20.0%)					
Belgium	0	1 (20.0%)	1 (10.0%)					
France	2 (40.0%)	2 (40.0%)	4 (40.0%)					
Germany	0	0	0					
Italy	0	0	0					
Saudi Arabia	1 (20.0%)	0	1 (10.0%)					
Spain	1 (20.0%)	1 (20.0%)	2 (20.0%)					
United Kingdom	0	0	0					
	
Origin								
N	3	4	7					
Europe	2 (66.7%)	4 (100.0%)	6 (85.7%)					
Middle-East/North-Africa	1 (33.3%)	0	1 (14.3%)					
	
Body weight (kg)								
N	5	5	10					
Mean	67.30	80.60	73.95					
SE	3.992	9.740	5.435					
SD	8.927	21.778	17.186					
95% C.I. *	(56.215; 78.385)	(53.559; 107.641)	(61.656; 86.244)					
Min	58.0	55.0	55.0					
Q1	60.00	64.00	60.00					
Median	67.00	80.00	69.25					
Q3	71.50	97.00	80.00					
Max	80.0	107.0	107.0					
	
Body mass index (kg/m²)								
N	5	5	10					
Mean	23.42	28.00	25.71					
SE	1.143	2.411	1.471					
SD	2.555	5.392	4.653					
95% C.I. *	(20.248; 26.592)	(21.305; 34.695)	(22.381; 29.039)					
Min	20.3	19.0	19.0					
Q1	22.10	27.20	22.10					
Median	22.60	30.10	26.05					
Q3	25.80	31.30	30.10					
Max	26.3	32.4	32.4					
	
Body mass index (kg/m²)								
N	5	5	10					
<25 kg/m²	3 (60.0%)	1 (20.0%)	4 (40.0%)					
≥25 - <30 kg/m²	2 (40.0%)	1 (20.0%)	3 (30.0%)					
≥30 kg/m²	0	3 (60.0%)	3 (30.0%)					
	

* Confidence interval for mean
N = number of subjects with data	
[TSIDEM01B.rtf] [\STAT\Analyses\Programs\FinalAnalysis\Final1\2.TLF\1.General\GEN_FA.sas] 23OCT2015, 16:53	
