# Supplementary material for: Efficacy of a 12-Week Simeprevir Plus Peginterferon/Ribavirin (PR) Regimen in Treatment-Naïve Patients with Hepatitis C Virus (HCV) Genotype 4 (GT4) Infection and Mild-To-Moderate Fibrosis Displaying Early On-Treatment Virologic Response
Source: PLoS One. 2017 Jan 5;12(1):e0168713. doi: 10.1371/journal.pone.0168713 (PMC5215882; doi:10.1371/journal.pone.0168713)
Supplement: S1 Dataset — (ZIP) [file pone.0168713.s002.zip › TSIDEM02.rtf]

TSIDEM02:	Baseline Disease Characteristics; Intent-to-treat (Study TMC435HPC3014)
Treatment Group = Simeprevir 12Wks 150 mg PR12/24	
	Genotype 4		
	12 Weeks 
Treatment	>12 Weeks 
Treatment	All Subjects				
Analysis set: intent-to-treat	34	33	67				
	
Baseline HCV RNA level (IU/mL)							
N	34	33	67				
Mean	2524844.1	3271651.5	2892674.6				
SE	1032105.10	594539.72	597335.46				
SD	6018155.17	3415370.69	4889401.49				
95% C.I. *	(425010.51; 4624677.73)	(2060613.73; 4482689.30)	(1700055.89; 4085293.36)				
Min	1300	68500	1300				
Q1	297000.0	1110000.0	513000.0				
Median	647000.0	2500000.0	1240000.0				
Q3	2740000.0	3990000.0	3640000.0				
Max	34900000	15600000	34900000				
	
Baseline log10 HCV RNA level							
N	34	33	67				
Mean	5.82	6.27	6.05				
SE	0.141	0.093	0.089				
SD	0.825	0.532	0.727				
95% C.I. *	(5.537; 6.112)	(6.084; 6.461)	(5.868; 6.223)				
Min	3.1	4.8	3.1				
Q1	5.47	6.05	5.71				
Median	5.81	6.40	6.09				
Q3	6.44	6.60	6.56				
Max	7.5	7.2	7.5				
	
Baseline HCV RNA level (IU/mL)							
N	34	33	67				
<400000 IU/mL	11 (32.4%)	3 (9.1%)	14 (20.9%)				
≥400000 - ≤800000 IU/mL	8 (23.5%)	4 (12.1%)	12 (17.9%)				
>800000 IU/mL	15 (44.1%)	26 (78.8%)	41 (61.2%)				
	
Metavir fibrosis score a							
N	34	33	67				
Score F0-F1	29 (85.3%)	25 (75.8%)	54 (80.6%)				
Score F2	5 (14.7%)	7 (21.2%)	12 (17.9%)				
Score F3	0	1 (3.0%)	1 (1.5%)				
	
Baseline ALT level (U/L)							
N	34	33	67				
Mean	64.4	60.1	62.3				
SE	10.34	12.06	7.87				
SD	60.27	69.27	64.40				
95% C.I. *	(43.38; 85.44)	(35.50; 84.62)	(46.56; 77.98)				
Min	15	18	15				
Q1	24.0	31.0	29.0				
Median	38.5	43.0	42.0				
Q3	78.0	60.0	72.0				
Max	270	419	419				
	
Baseline ALT toxicity grade							
N	34	33	67				
Grade 0	22 (64.7%)	21 (63.6%)	43 (64.2%)				
Grade 1	6 (17.6%)	9 (27.3%)	15 (22.4%)				
Grade 2	5 (14.7%)	2 (6.1%)	7 (10.4%)				
Grade 3	1 (2.9%)	1 (3.0%)	2 (3.0%)				
	
HCV geno/subtype (coalesce) b							
N	34	33	67				
1a	0	0	0				
1b	0	0	0				
4	1 (2.9%)	1 (3.0%)	2 (3.0%)				
4a	14 (41.2%)	13 (39.4%)	27 (40.3%)				
4a/4c/4d	1 (2.9%)	1 (3.0%)	2 (3.0%)				
4c	1 (2.9%)	1 (3.0%)	2 (3.0%)				
4d	13 (38.2%)	12 (36.4%)	25 (37.3%)				
4e	0	1 (3.0%)	1 (1.5%)				
4f	1 (2.9%)	0	1 (1.5%)				
4k	1 (2.9%)	1 (3.0%)	2 (3.0%)				
4n	1 (2.9%)	1 (3.0%)	2 (3.0%)				
4q	1 (2.9%)	1 (3.0%)	2 (3.0%)				
4r	0	1 (3.0%)	1 (1.5%)				
	
HCV geno/subtype (ns5b)							
N	30	30	60				
4a	13 (43.3%)	12 (40.0%)	25 (41.7%)				
4c	1 (3.3%)	1 (3.3%)	2 (3.3%)				
4d	13 (43.3%)	12 (40.0%)	25 (41.7%)				
4e	0	1 (3.3%)	1 (1.7%)				
4k	1 (3.3%)	1 (3.3%)	2 (3.3%)				
4n	1 (3.3%)	1 (3.3%)	2 (3.3%)				
4q	1 (3.3%)	1 (3.3%)	2 (3.3%)				
4r	0	1 (3.3%)	1 (1.7%)				
	
HCV geno/subtype (Trugene assay)							
N	2	1	3				
1a	0	0	0				
1b	0	0	0				
4	1 (50.0%)	0	1 (33.3%)				
4a	1 (50.0%)	1 (100.0%)	2 (66.7%)				
	
HCV geno/subtype (LIPA 2.0 assay)							
N	32	32	64				
1a	0	0	0				
1b	0	0	0				
4	8 (25.0%)	13 (40.6%)	21 (32.8%)				
4a/4c/4d	21 (65.6%)	17 (53.1%)	38 (59.4%)				
4e	0	1 (3.1%)	1 (1.6%)				
4f	1 (3.1%)	0	1 (1.6%)				
4h	2 (6.3%)	1 (3.1%)	3 (4.7%)				
	
HCV geno/subtype as stratified							
N	34	33	67				
1a	0	0	0				
1b	0	0	0				
4	34 (100.0%)	33 (100.0%)	67 (100.0%)				
	
Il28b genotype							
N	34	33	67				
CC	14 (41.2%)	1 (3.0%)	15 (22.4%)				
CT	15 (44.1%)	27 (81.8%)	42 (62.7%)				
TT	5 (14.7%)	5 (15.2%)	10 (14.9%)				
	
Duration of HCV infection (years)							
N	13	13	26				
Mean	12.49	14.40	13.45				
SE	2.895	3.271	2.148				
SD	10.437	11.794	10.954				
95% C.I. *	(6.185; 18.799)	(7.273; 21.527)	(9.022; 17.871)				
Min	1.4	0.6	0.6				
Q1	4.20	2.40	2.70				
Median	9.40	11.20	10.50				
Q3	20.70	26.30	25.20				
Max	30.3	30.3	30.3				
	
Time since diagnosis (years)							
N	33	33	66				
Mean	7.44	9.41	8.42				
SE	1.179	1.482	0.948				
SD	6.771	8.516	7.698				
95% C.I. *	(5.035; 9.837)	(6.386; 12.426)	(6.529; 10.314)				
Min	0.5	0.6	0.5				
Q1	1.50	1.90	1.70				
Median	4.20	7.70	6.10				
Q3	13.20	16.70	14.30				
Max	24.4	26.3	26.3				
	
Il28b genotype as stratified							
N	34	33	67				
CC	14 (41.2%)	1 (3.0%)	15 (22.4%)				
CT	15 (44.1%)	27 (81.8%)	42 (62.7%)				
TT	5 (14.7%)	5 (15.2%)	10 (14.9%)				
	
Mode of hepatitis C infection							
N	34	33	67				
Blood transfusion	4 (11.8%)	6 (18.2%)	10 (14.9%)				
Hemophilia-associated injections	0	0	0				
Heterosexual contact	0	0	0				
Intravenously injectable drug use	5 (14.7%)	4 (12.1%)	9 (13.4%)				
Mother to child transmission	3 (8.8%)	1 (3.0%)	4 (6.0%)				
Multiple	0	1 (3.0%)	1 (1.5%)				
Other	22 (64.7%)	21 (63.6%)	43 (64.2%)				
	

* Confidence interval for mean
N = number of subjects with data
a	Results from the Metavir scoring system and the non-invasive methods.
b	HCV Geno/Subtype (Coalesce) is based on the NS5B assay, and if not available on LIPA HCV II or Trugene results.	
[TSIDEM02.rtf] [\STAT\Analyses\Programs\FinalAnalysis\Final1\2.TLF\1.General\GEN_FA.sas] 23OCT2015, 16:53	
