# Supplementary material for: Efficacy of a 12-Week Simeprevir Plus Peginterferon/Ribavirin (PR) Regimen in Treatment-Naïve Patients with Hepatitis C Virus (HCV) Genotype 4 (GT4) Infection and Mild-To-Moderate Fibrosis Displaying Early On-Treatment Virologic Response
Source: PLoS One. 2017 Jan 5;12(1):e0168713. doi: 10.1371/journal.pone.0168713 (PMC5215882; doi:10.1371/journal.pone.0168713)
Supplement: S1 Dataset — (ZIP) [file pone.0168713.s002.zip › TSIDEM02B.rtf]

TSIDEM02B:	Baseline Disease Characteristics by Subgroups of Interest; Intent-to-treat (Study TMC435HPC3014)
Treatment Group = Simeprevir 12Wks 150 mg PR12/24 
HCV Geno/Subtype = 4a	
	Genotype 4		
	12 Weeks 
Treatment	>12 Weeks 
Treatment	All Subjects		
Analysis set: intent-to-treat	8	10	18		
	
Baseline HCV RNA level (IU/mL)					
N	8	10	18		
Mean	642625.0	3514800.0	2238277.8		
SE	245417.93	1439415.50	860364.26		
SD	694146.74	4551831.49	3650216.43		
95% C.I. *	(62303.80; 1222946.20)	(258615.91; 6770984.09)	(423067.85; 4053487.70)		
Min	108000	486000	108000		
Q1	160000.0	1130000.0	486000.0		
Median	419500.0	1805000.0	1085000.0		
Q3	852000.0	4740000.0	2170000.0		
Max	2170000	15600000	15600000		
	
Baseline log10 HCV RNA level					
N	8	10	18		
Mean	5.60	6.31	5.99		
SE	0.161	0.146	0.135		
SD	0.456	0.461	0.573		
95% C.I. *	(5.221; 5.983)	(5.978; 6.638)	(5.709; 6.279)		
Min	5.0	5.7	5.0		
Q1	5.20	6.05	5.69		
Median	5.60	6.25	6.04		
Q3	5.92	6.68	6.34		
Max	6.3	7.2	7.2		
	
Baseline HCV RNA level (IU/mL)					
N	8	10	18		
<400000 IU/mL	4 (50.0%)	0	4 (22.2%)		
≥400000 - ≤800000 IU/mL	2 (25.0%)	2 (20.0%)	4 (22.2%)		
>800000 IU/mL	2 (25.0%)	8 (80.0%)	10 (55.6%)		
	
Metavir fibrosis score a					
N	8	10	18		
Score F0-F1	6 (75.0%)	8 (80.0%)	14 (77.8%)		
Score F2	2 (25.0%)	2 (20.0%)	4 (22.2%)		
	
Baseline ALT level (U/L)					
N	8	10	18		
Mean	61.1	48.4	54.1		
SE	15.79	5.68	7.58		
SD	44.67	17.97	32.17		
95% C.I. *	(23.78; 98.47)	(35.54; 61.26)	(38.06; 70.05)		
Min	24	24	24		
Q1	33.0	36.0	36.0		
Median	40.5	48.0	45.5		
Q3	82.5	52.0	63.0		
Max	153	87	153		
	
Baseline ALT toxicity grade					
N	8	10	18		
Grade 0	5 (62.5%)	7 (70.0%)	12 (66.7%)		
Grade 1	2 (25.0%)	3 (30.0%)	5 (27.8%)		
Grade 2	1 (12.5%)	0	1 (5.6%)		
	
HCV geno/subtype (coalesce) b					
N	8	10	18		
4a	8 (100.0%)	10 (100.0%)	18 (100.0%)		
	
HCV geno/subtype (ns5b)					
N	7	9	16		
4a	7 (100.0%)	9 (100.0%)	16 (100.0%)		
	
HCV geno/subtype (Trugene assay)					
N	1	1	2		
4a	1 (100.0%)	1 (100.0%)	2 (100.0%)		
	
HCV geno/subtype (LIPA 2.0 assay)					
N	7	9	16		
4	2 (28.6%)	6 (66.7%)	8 (50.0%)		
4a/4c/4d	5 (71.4%)	3 (33.3%)	8 (50.0%)		
	
HCV geno/subtype as stratified					
N	8	10	18		
4	8 (100.0%)	10 (100.0%)	18 (100.0%)		
	
Il28b genotype					
N	8	10	18		
CC	1 (12.5%)	0	1 (5.6%)		
CT	5 (62.5%)	8 (80.0%)	13 (72.2%)		
TT	2 (25.0%)	2 (20.0%)	4 (22.2%)		
	
Duration of HCV infection (years)					
N	4	5	9		
Mean	19.58	16.38	17.80		
SE	4.902	6.386	3.956		
SD	9.804	14.280	11.867		
95% C.I. *	(3.975; 35.175)	(-1.351; 34.111)	(8.678; 26.922)		
Min	9.4	0.6	0.6		
Q1	11.40	2.40	9.40		
Median	19.30	19.20	19.20		
Q3	27.75	29.40	29.40		
Max	30.3	30.3	30.3		
	
Time since diagnosis (years)					
N	8	10	18		
Mean	7.38	9.21	8.39		
SE	2.359	2.763	1.820		
SD	6.673	8.738	7.723		
95% C.I. *	(1.796; 12.954)	(2.959; 15.461)	(4.554; 12.235)		
Min	0.9	0.6	0.6		
Q1	1.85	1.90	1.90		
Median	4.70	5.65	4.70		
Q3	13.30	16.70	14.30		
Max	18.4	24.4	24.4		
	
Il28b genotype as stratified					
N	8	10	18		
CC	1 (12.5%)	0	1 (5.6%)		
CT	5 (62.5%)	8 (80.0%)	13 (72.2%)		
TT	2 (25.0%)	2 (20.0%)	4 (22.2%)		
	
Mode of hepatitis C infection					
N	8	10	18		
Blood transfusion	1 (12.5%)	1 (10.0%)	2 (11.1%)		
Intravenously injectable drug use	1 (12.5%)	2 (20.0%)	3 (16.7%)		
Other	6 (75.0%)	7 (70.0%)	13 (72.2%)		
	

* Confidence interval for mean
N = number of subjects with data
a	Results from the Metavir scoring system and the non-invasive methods.
b	HCV Geno/Subtype (Coalesce) is based on the NS5B assay, and if not available on LIPA HCV II or Trugene results.	
[TSIDEM02B.rtf] [\STAT\Analyses\Programs\Interim4\Final4\2.TLF\1.General_IA4\GEN_IA4.sas] 18NOV2014, 15:26	

TSIDEM02B:	Baseline Disease Characteristics by Subgroups of Interest; Intent-to-treat (Study TMC435HPC3014)
Treatment Group = Simeprevir 12Wks 150 mg PR12/24 
HCV Geno/Subtype = 4d	
	Genotype 4		
	12 Weeks 
Treatment	>12 Weeks 
Treatment	All Subjects		
Analysis set: intent-to-treat	10	10	20		
	
Baseline HCV RNA level (IU/mL)					
N	10	10	20		
Mean	1420170.0	3370050.0	2395110.0		
SE	418555.04	988460.68	568266.10		
SD	1323587.24	3125787.12	2541363.24		
95% C.I. *	(473332.73; 2367007.27)	(1133996.60; 5606103.40)	(1205715.39; 3584504.61)		
Min	41700	68500	41700		
Q1	208000.0	1110000.0	391000.0		
Median	948500.0	3020000.0	1670000.0		
Q3	2740000.0	3990000.0	3565000.0		
Max	3730000	9300000	9300000		
	
Baseline log10 HCV RNA level					
N	10	10	20		
Mean	5.85	6.23	6.04		
SE	0.205	0.215	0.151		
SD	0.648	0.679	0.675		
95% C.I. *	(5.390; 6.317)	(5.746; 6.718)	(5.727; 6.359)		
Min	4.6	4.8	4.6		
Q1	5.32	6.05	5.55		
Median	5.96	6.48	6.21		
Q3	6.44	6.60	6.55		
Max	6.6	7.0	7.0		
	
Baseline HCV RNA level (IU/mL)					
N	10	10	20		
<400000 IU/mL	3 (30.0%)	2 (20.0%)	5 (25.0%)		
≥400000 - ≤800000 IU/mL	2 (20.0%)	0	2 (10.0%)		
>800000 IU/mL	5 (50.0%)	8 (80.0%)	13 (65.0%)		
	
Metavir fibrosis score a					
N	10	10	20		
Score F0-F1	8 (80.0%)	7 (70.0%)	15 (75.0%)		
Score F2	2 (20.0%)	3 (30.0%)	5 (25.0%)		
	
Baseline ALT level (U/L)					
N	10	10	20		
Mean	81.7	60.5	71.1		
SE	24.62	11.10	13.37		
SD	77.86	35.10	59.78		
95% C.I. *	(26.00; 137.40)	(35.39; 85.61)	(43.12; 99.08)		
Min	22	29	22		
Q1	24.0	34.0	30.0		
Median	56.0	51.5	51.5		
Q3	115.0	74.0	78.5		
Max	270	146	270		
	
Baseline ALT toxicity grade					
N	10	10	20		
Grade 0	5 (50.0%)	5 (50.0%)	10 (50.0%)		
Grade 1	2 (20.0%)	4 (40.0%)	6 (30.0%)		
Grade 2	2 (20.0%)	1 (10.0%)	3 (15.0%)		
Grade 3	1 (10.0%)	0	1 (5.0%)		
	
HCV geno/subtype (coalesce) b					
N	10	10	20		
4d	10 (100.0%)	10 (100.0%)	20 (100.0%)		
	
HCV geno/subtype (ns5b)					
N	10	10	20		
4d	10 (100.0%)	10 (100.0%)	20 (100.0%)		
	
HCV geno/subtype (LIPA 2.0 assay)					
N	10	10	20		
4	1 (10.0%)	1 (10.0%)	2 (10.0%)		
4a/4c/4d	9 (90.0%)	9 (90.0%)	18 (90.0%)		
	
HCV geno/subtype as stratified					
N	10	10	20		
4	10 (100.0%)	10 (100.0%)	20 (100.0%)		
	
Il28b genotype					
N	10	10	20		
CC	5 (50.0%)	0	5 (25.0%)		
CT	4 (40.0%)	8 (80.0%)	12 (60.0%)		
TT	1 (10.0%)	2 (20.0%)	3 (15.0%)		
	
Duration of HCV infection (years)					
N	1	1	2		
Mean	1.40	2.40	1.90		
SE			0.500		
SD			0.707		
95% C.I. *			(-4.453; 8.253)		
Min			1.4		
Q1			1.40		
Median			1.90		
Q3			2.40		
Max			2.4		
	
Time since diagnosis (years)					
N	9	10	19		
Mean	11.46	10.11	10.75		
SE	2.800	3.193	2.088		
SD	8.401	10.096	9.100		
95% C.I. *	(4.998; 17.913)	(2.888; 17.332)	(6.361; 15.133)		
Min	0.6	1.0	0.6		
Q1	3.90	1.30	1.30		
Median	11.40	4.65	10.40		
Q3	17.10	21.30	18.30		
Max	24.4	24.4	24.4		
	
Il28b genotype as stratified					
N	10	10	20		
CC	5 (50.0%)	0	5 (25.0%)		
CT	4 (40.0%)	8 (80.0%)	12 (60.0%)		
TT	1 (10.0%)	2 (20.0%)	3 (15.0%)		
	
Mode of hepatitis C infection					
N	10	10	20		
Intravenously injectable drug use	4 (40.0%)	2 (20.0%)	6 (30.0%)		
Mother to child transmission	1 (10.0%)	0	1 (5.0%)		
Multiple	0	1 (10.0%)	1 (5.0%)		
Other	5 (50.0%)	7 (70.0%)	12 (60.0%)		
	

* Confidence interval for mean
N = number of subjects with data
a	Results from the Metavir scoring system and the non-invasive methods.
b	HCV Geno/Subtype (Coalesce) is based on the NS5B assay, and if not available on LIPA HCV II or Trugene results.	
[TSIDEM02B.rtf] [\STAT\Analyses\Programs\Interim4\Final4\2.TLF\1.General_IA4\GEN_IA4.sas] 18NOV2014, 15:26	

TSIDEM02B:	Baseline Disease Characteristics by Subgroups of Interest; Intent-to-treat (Study TMC435HPC3014)
Treatment Group = Simeprevir 12Wks 150 mg PR12/24 
HCV Geno/Subtype = 4other	
	Genotype 4		
	12 Weeks 
Treatment	>12 Weeks 
Treatment	All Subjects		
Analysis set: intent-to-treat	6	6	12		
	
Baseline HCV RNA level (IU/mL)					
N	6	6	12		
Mean	6275216.7	2767166.7	4521191.7		
SE	5727016.04	827401.57	2808831.35		
SD	14028267.05	2026711.66	9730077.21		
95% C.I. *	(-8446546.74; 20996980.08)	(640263.22; 4894070.12)	(-1661004.45; 10703387.78)		
Min	1300	513000	1300		
Q1	359000.0	1060000.0	565000.0		
Median	620500.0	2365000.0	1105000.0		
Q3	1150000.0	4730000.0	3800000.0		
Max	34900000	5570000	34900000		
	
Baseline log10 HCV RNA level					
N	6	6	12		
Mean	5.64	6.31	5.98		
SE	0.584	0.162	0.306		
SD	1.431	0.397	1.060		
95% C.I. *	(4.142; 7.144)	(5.897; 6.730)	(5.305; 6.652)		
Min	3.1	5.7	3.1		
Q1	5.56	6.03	5.75		
Median	5.79	6.36	6.04		
Q3	6.06	6.67	6.57		
Max	7.5	6.7	7.5		
	
Baseline HCV RNA level (IU/mL)					
N	6	6	12		
<400000 IU/mL	2 (33.3%)	0	2 (16.7%)		
≥400000 - ≤800000 IU/mL	2 (33.3%)	1 (16.7%)	3 (25.0%)		
>800000 IU/mL	2 (33.3%)	5 (83.3%)	7 (58.3%)		
	
Metavir fibrosis score a					
N	6	6	12		
Score F0-F1	5 (83.3%)	5 (83.3%)	10 (83.3%)		
Score F2	1 (16.7%)	1 (16.7%)	2 (16.7%)		
	
Baseline ALT level (U/L)					
N	6	6	12		
Mean	92.3	47.8	70.1		
SE	32.06	9.83	17.34		
SD	78.52	24.09	60.05		
95% C.I. *	(9.93; 174.74)	(22.56; 73.11)	(31.93; 108.24)		
Min	24	28	24		
Q1	33.0	29.0	31.0		
Median	60.0	42.0	43.5		
Q3	162.0	53.0	85.5		
Max	215	93	215		
	
Baseline ALT toxicity grade					
N	6	6	12		
Grade 0	3 (50.0%)	3 (50.0%)	6 (50.0%)		
Grade 1	1 (16.7%)	2 (33.3%)	3 (25.0%)		
Grade 2	2 (33.3%)	1 (16.7%)	3 (25.0%)		
	
HCV geno/subtype (coalesce) b					
N	6	6	12		
4	1 (16.7%)	0	1 (8.3%)		
4a/4c/4d	1 (16.7%)	1 (16.7%)	2 (16.7%)		
4c	1 (16.7%)	1 (16.7%)	2 (16.7%)		
4e	0	1 (16.7%)	1 (8.3%)		
4f	1 (16.7%)	0	1 (8.3%)		
4k	1 (16.7%)	1 (16.7%)	2 (16.7%)		
4n	0	1 (16.7%)	1 (8.3%)		
4q	1 (16.7%)	1 (16.7%)	2 (16.7%)		
	
HCV geno/subtype (ns5b)					
N	3	5	8		
4c	1 (33.3%)	1 (20.0%)	2 (25.0%)		
4e	0	1 (20.0%)	1 (12.5%)		
4k	1 (33.3%)	1 (20.0%)	2 (25.0%)		
4n	0	1 (20.0%)	1 (12.5%)		
4q	1 (33.3%)	1 (20.0%)	2 (25.0%)		
	
HCV geno/subtype (Trugene assay)					
N	1	0	1		
4	1 (100.0%)	0	1 (100.0%)		
	
HCV geno/subtype (LIPA 2.0 assay)					
N	5	6	11		
4	1 (20.0%)	1 (16.7%)	2 (18.2%)		
4a/4c/4d	2 (40.0%)	3 (50.0%)	5 (45.5%)		
4e	0	1 (16.7%)	1 (9.1%)		
4f	1 (20.0%)	0	1 (9.1%)		
4h	1 (20.0%)	1 (16.7%)	2 (18.2%)		
	
HCV geno/subtype as stratified					
N	6	6	12		
4	6 (100.0%)	6 (100.0%)	12 (100.0%)		
	
Il28b genotype					
N	6	6	12		
CC	2 (33.3%)	0	2 (16.7%)		
CT	3 (50.0%)	5 (83.3%)	8 (66.7%)		
TT	1 (16.7%)	1 (16.7%)	2 (16.7%)		
	
Duration of HCV infection (years)					
N	1	3	4		
Mean	29.40	21.33	23.35		
SE		5.526	4.397		
SD		9.571	8.794		
95% C.I. *		(-2.442; 45.109)	(9.357; 37.343)		
Min		10.3	10.3		
Q1		10.30	18.30		
Median		26.30	26.85		
Q3		27.40	28.40		
Max		27.4	29.4		
	
Time since diagnosis (years)					
N	6	6	12		
Mean	7.37	12.18	9.78		
SE	2.683	3.475	2.215		
SD	6.572	8.512	7.674		
95% C.I. *	(0.470; 14.264)	(3.251; 21.116)	(4.899; 14.651)		
Min	1.2	1.1	1.1		
Q1	1.30	8.30	1.50		
Median	7.00	10.60	10.60		
Q3	13.30	16.20	13.85		
Max	14.4	26.3	26.3		
	
Il28b genotype as stratified					
N	6	6	12		
CC	2 (33.3%)	0	2 (16.7%)		
CT	3 (50.0%)	5 (83.3%)	8 (66.7%)		
TT	1 (16.7%)	1 (16.7%)	2 (16.7%)		
	
Mode of hepatitis C infection					
N	6	6	12		
Blood transfusion	1 (16.7%)	2 (33.3%)	3 (25.0%)		
MSM	1 (16.7%)	0	1 (8.3%)		
Mother to child transmission	0	1 (16.7%)	1 (8.3%)		
Other	4 (66.7%)	3 (50.0%)	7 (58.3%)		
	

* Confidence interval for mean
N = number of subjects with data
a	Results from the Metavir scoring system and the non-invasive methods.
b	HCV Geno/Subtype (Coalesce) is based on the NS5B assay, and if not available on LIPA HCV II or Trugene results.	
[TSIDEM02B.rtf] [\STAT\Analyses\Programs\Interim4\Final4\2.TLF\1.General_IA4\GEN_IA4.sas] 18NOV2014, 15:26	

TSIDEM02B:	Baseline Disease Characteristics by Subgroups of Interest; Intent-to-treat (Study TMC435HPC3014)
Treatment Group = Simeprevir 12Wks 150 mg PR12/24 
IL28b = CC	
	Genotype 4		
	12 Weeks 
Treatment	All Subjects		
Analysis set: intent-to-treat	8	8		
Baseline HCV RNA level (IU/mL)				
N	8	8		
Mean	4908837.5	4908837.5		
SE	4291394.29	4291394.29		
SD	12137896.02	12137896.02		
95% C.I. *	(-5238697.52; 15056372.52)	(-5238697.52; 15056372.52)		
Min	41700	41700		
Q1	165500.0	165500.0		
Median	384000.0	384000.0		
Q3	1615000.0	1615000.0		
Max	34900000	34900000		
	
Baseline log10 HCV RNA level							
N	8	8		
Mean	5.75	5.75		
SE	0.318	0.318		
SD	0.900	0.900		
95% C.I. *	(5.002; 6.508)	(5.002; 6.508)		
Min	4.6	4.6		
Q1	5.22	5.22		
Median	5.53	5.53		
Q3	6.19	6.19		
Max	7.5	7.5		
	
Baseline HCV RNA level (IU/mL)							
N	8	8		
<400000 IU/mL	4 (50.0%)	4 (50.0%)		
≥400000 - ≤800000 IU/mL	1 (12.5%)	1 (12.5%)		
>800000 IU/mL	3 (37.5%)	3 (37.5%)		
	
Metavir fibrosis score a							
N	8	8		
Score F0-F1	7 (87.5%)	7 (87.5%)		
Score F2	1 (12.5%)	1 (12.5%)		
	
Baseline ALT level (U/L)							
N	8	8		
Mean	100.9	100.9		
SE	20.23	20.23		
SD	57.22	57.22		
95% C.I. *	(53.04; 148.71)	(53.04; 148.71)		
Min	42	42		
Q1	56.0	56.0		
Median	92.0	92.0		
Q3	127.0	127.0		
Max	215	215		
	
Baseline ALT toxicity grade							
N	8	8		
Grade 0	2 (25.0%)	2 (25.0%)		
Grade 1	3 (37.5%)	3 (37.5%)		
Grade 2	3 (37.5%)	3 (37.5%)		
Grade 3	0	0		
	
HCV geno/subtype (coalesce) b							
N	8	8		
1a	0	0		
1b	0	0		
4a	1 (12.5%)	1 (12.5%)		
4a/4c/4d	1 (12.5%)	1 (12.5%)		
4d	5 (62.5%)	5 (62.5%)		
4f	1 (12.5%)	1 (12.5%)		
	
HCV geno/subtype (ns5b)							
N	6	6		
4a	1 (16.7%)	1 (16.7%)		
4d	5 (83.3%)	5 (83.3%)		
	
HCV geno/subtype (LIPA 2.0 assay)							
N	8	8		
1a	0	0		
1b	0	0		
4	2 (25.0%)	2 (25.0%)		
4a/4c/4d	5 (62.5%)	5 (62.5%)		
4f	1 (12.5%)	1 (12.5%)		
	
HCV geno/subtype as stratified				
N	8	8		
1a	0	0		
1b	0	0		
4	8 (100.0%)	8 (100.0%)		
	
Il28b genotype							
N	8	8		
CC	8 (100.0%)	8 (100.0%)		
	
Duration of HCV infection (years)							
N	1	1		
Mean	1.40	1.40		
SE				
SD				
95% C.I. *				
Min				
Q1				
Median				
Q3				
Max				
	
Time since diagnosis (years)							
N	7	7		
Mean	3.54	3.54		
SE	1.502	1.502		
SD	3.975	3.975		
95% C.I. *	(-0.133; 7.219)	(-0.133; 7.219)		
Min	0.6	0.6		
Q1	0.60	0.60		
Median	1.30	1.30		
Q3	5.80	5.80		
Max	11.4	11.4		
	
Il28b genotype as stratified							
N	8	8		
CC	8 (100.0%)	8 (100.0%)		
	
Mode of hepatitis C infection							
N	8	8		
Blood transfusion	0	0		
Heterosexual contact	0	0		
Intravenously injectable drug use	1 (12.5%)	1 (12.5%)		
MSM	1 (12.5%)	1 (12.5%)		
Mother to child transmission	0	0		
Multiple	0	0		
Other	6 (75.0%)	6 (75.0%)		
	

* Confidence interval for mean
N = number of subjects with data
a	Results from the Metavir scoring system and the non-invasive methods.
b	HCV Geno/Subtype (Coalesce) is based on the NS5B assay, and if not available on LIPA HCV II or Trugene results.	
[TSIDEM02B.rtf] [\STAT\Analyses\Programs\Interim4\Final4\2.TLF\1.General_IA4\GEN_IA4.sas] 18NOV2014, 15:26	

TSIDEM02B:	Baseline Disease Characteristics by Subgroups of Interest; Intent-to-treat (Study TMC435HPC3014)
Treatment Group = Simeprevir 12Wks 150 mg PR12/24 
IL28b = CT	
	Genotype 4	
	12 Weeks 
Treatment	>12 Weeks 
Treatment	All Subjects	
Analysis set: intent-to-treat	12	21	33	
	
Baseline HCV RNA level (IU/mL)							
N	12	21	33	
Mean	1327941.7	3321500.0	2596569.7	
SE	354585.49	768549.12	528564.68	
SD	1228320.17	3521934.54	3036372.94	
95% C.I. *	(547504.26; 2108379.07)	(1718334.62; 4924665.38)	(1519918.67; 3673220.73)	
Min	1300	68500	1300	
Q1	419500.0	1110000.0	582000.0	
Median	832000.0	2500000.0	1860000.0	
Q3	2455000.0	4730000.0	3720000.0	
Max	3730000	15600000	15600000	
	
Baseline log10 HCV RNA level							
N	12	21	33	
Mean	5.74	6.27	6.08	
SE	0.269	0.122	0.130	
SD	0.933	0.560	0.749	
95% C.I. *	(5.152; 6.338)	(6.012; 6.522)	(5.812; 6.343)	
Min	3.1	4.8	3.1	
Q1	5.60	6.05	5.76	
Median	5.91	6.40	6.27	
Q3	6.39	6.67	6.57	
Max	6.6	7.2	7.2	
	
Baseline HCV RNA level (IU/mL)							
N	12	21	33	
<400000 IU/mL	3 (25.0%)	2 (9.5%)	5 (15.2%)	
≥400000 - ≤800000 IU/mL	3 (25.0%)	3 (14.3%)	6 (18.2%)	
>800000 IU/mL	6 (50.0%)	16 (76.2%)	22 (66.7%)	
	
Metavir fibrosis score a							
N	12	21	33	
Score F0-F1	8 (66.7%)	16 (76.2%)	24 (72.7%)	
Score F2	4 (33.3%)	5 (23.8%)	9 (27.3%)	
	
Baseline ALT level (U/L)							
N	12	21	33	
Mean	57.0	56.5	56.7	
SE	14.47	6.16	6.42	
SD	50.14	28.21	36.90	
95% C.I. *	(25.14; 88.86)	(43.64; 69.32)	(43.58; 69.75)	
Min	22	28	22	
Q1	24.0	36.0	29.0	
Median	33.0	49.0	45.0	
Q3	70.5	72.0	72.0	
Max	162	146	162	
	
Baseline ALT toxicity grade							
N	12	21	33	
Grade 0	8 (66.7%)	11 (52.4%)	19 (57.6%)	
Grade 1	2 (16.7%)	8 (38.1%)	10 (30.3%)	
Grade 2	2 (16.7%)	2 (9.5%)	4 (12.1%)	
	
HCV geno/subtype (coalesce) b							
N	12	21	33	
1a	0	0	0	
1b	0	0	0	
4	1 (8.3%)	0	1 (3.0%)	
4a	5 (41.7%)	8 (38.1%)	13 (39.4%)	
4a/4c/4d	0	1 (4.8%)	1 (3.0%)	
4c	0	1 (4.8%)	1 (3.0%)	
4d	4 (33.3%)	8 (38.1%)	12 (36.4%)	
4e	0	1 (4.8%)	1 (3.0%)	
4k	1 (8.3%)	1 (4.8%)	2 (6.1%)	
4n	0	1 (4.8%)	1 (3.0%)	
4q	1 (8.3%)	0	1 (3.0%)	
	
HCV geno/subtype (ns5b)							
N	10	19	29	
4a	4 (40.0%)	7 (36.8%)	11 (37.9%)	
4c	0	1 (5.3%)	1 (3.4%)	
4d	4 (40.0%)	8 (42.1%)	12 (41.4%)	
4e	0	1 (5.3%)	1 (3.4%)	
4k	1 (10.0%)	1 (5.3%)	2 (6.9%)	
4n	0	1 (5.3%)	1 (3.4%)	
4q	1 (10.0%)	0	1 (3.4%)	
	
HCV geno/subtype (Trugene assay)							
N	2	1	3	
1a	0	0	0	
1b	0	0	0	
4	1 (50.0%)	0	1 (33.3%)	
4a	1 (50.0%)	1 (100.0%)	2 (66.7%)	
	
HCV geno/subtype (LIPA 2.0 assay)							
N	10	20	30	
1a	0	0	0	
1b	0	0	0	
4	1 (10.0%)	5 (25.0%)	6 (20.0%)	
4a/4c/4d	8 (80.0%)	13 (65.0%)	21 (70.0%)	
4e	0	1 (5.0%)	1 (3.3%)	
4h	1 (10.0%)	1 (5.0%)	2 (6.7%)	
	
HCV geno/subtype as stratified							
N	12	21	33	
1a	0	0	0	
1b	0	0	0	
4	12 (100.0%)	21 (100.0%)	33 (100.0%)	
	
Il28b genotype							
N	12	21	33	
CT	12 (100.0%)	21 (100.0%)	33 (100.0%)	
	
Duration of HCV infection (years)							
N	5	8	13	
Mean	21.54	15.25	17.67	
SE	4.275	4.550	3.249	
SD	9.560	12.869	11.714	
95% C.I. *	(9.670; 33.410)	(4.491; 26.009)	(10.591; 24.748)	
Min	9.4	0.6	0.6	
Q1	13.40	2.40	9.40	
Median	25.20	14.75	19.20	
Q3	29.40	28.40	29.40	
Max	30.3	30.3	30.3	
	
Time since diagnosis (years)							
N	12	21	33	
Mean	12.19	10.07	10.84	
SE	2.143	1.895	1.427	
SD	7.424	8.685	8.195	
95% C.I. *	(7.475; 16.909)	(6.113; 14.020)	(7.933; 13.745)	
Min	0.9	0.6	0.6	
Q1	6.05	1.90	1.90	
Median	13.35	8.30	12.00	
Q3	17.35	16.70	16.70	
Max	24.4	24.4	24.4	
	
Il28b genotype as stratified							
N	12	21	33	
CT	12 (100.0%)	21 (100.0%)	33 (100.0%)	
	
Mode of hepatitis C infection							
N	12	21	33	
Blood transfusion	2 (16.7%)	2 (9.5%)	4 (12.1%)	
Hemophilia-associated injections	0	0	0	
Heterosexual contact	0	0	0	
Intravenously injectable drug use	3 (25.0%)	3 (14.3%)	6 (18.2%)	
Mother to child transmission	1 (8.3%)	1 (4.8%)	2 (6.1%)	
Multiple	0	0	0	
Other	6 (50.0%)	15 (71.4%)	21 (63.6%)	
	

* Confidence interval for mean
N = number of subjects with data
a	Results from the Metavir scoring system and the non-invasive methods.
b	HCV Geno/Subtype (Coalesce) is based on the NS5B assay, and if not available on LIPA HCV II or Trugene results.	
[TSIDEM02B.rtf] [\STAT\Analyses\Programs\Interim4\Final4\2.TLF\1.General_IA4\GEN_IA4.sas] 18NOV2014, 15:26	

TSIDEM02B:	Baseline Disease Characteristics by Subgroups of Interest; Intent-to-treat (Study TMC435HPC3014)
Treatment Group = Simeprevir 12Wks 150 mg PR12/24 
IL28b = TT	
	Genotype 4	
	12 Weeks 
Treatment	>12 Weeks 
Treatment	All Subjects	
Analysis set: intent-to-treat	4	5	9	
	
Baseline HCV RNA level (IU/mL)							
N	4	5	9	
Mean	447000.0	3140000.0	1943111.1	
SE	133495.94	1564304.96	952124.55	
SD	266991.89	3497892.22	2856373.64	
95% C.I. *	(22156.33; 871843.67)	(-1203206.84; 7483206.84)	(-252492.03; 4138714.25)	
Min	108000	1060000	108000	
Q1	233500.0	1220000.0	657000.0	
Median	508000.0	1490000.0	1060000.0	
Q3	660500.0	2630000.0	1490000.0	
Max	664000	9300000	9300000	
	
Baseline log10 HCV RNA level							
N	4	5	9	
Mean	5.56	6.33	5.99	
SE	0.185	0.172	0.181	
SD	0.371	0.385	0.542	
95% C.I. *	(4.967; 6.147)	(5.857; 6.813)	(5.573; 6.406)	
Min	5.0	6.0	5.0	
Q1	5.29	6.09	5.82	
Median	5.69	6.17	6.03	
Q3	5.82	6.42	6.17	
Max	5.8	7.0	7.0	
	
Baseline HCV RNA level (IU/mL)							
N	4	5	9	
<400000 IU/mL	2 (50.0%)	0	2 (22.2%)	
≥400000 - ≤800000 IU/mL	2 (50.0%)	0	2 (22.2%)	
>800000 IU/mL	0	5 (100.0%)	5 (55.6%)	
	
Metavir fibrosis score a							
N	4	5	9	
Score F0-F1	4 (100.0%)	4 (80.0%)	8 (88.9%)	
Score F2	0	1 (20.0%)	1 (11.1%)	
	
Baseline ALT level (U/L)							
N	4	5	9	
Mean	92.3	38.0	62.1	
SE	59.26	5.18	26.15	
SD	118.53	11.58	78.44	
95% C.I. *	(-96.35; 280.85)	(23.63; 52.37)	(1.82; 122.40)	
Min	30	24	24	
Q1	31.5	29.0	30.0	
Median	34.5	41.0	36.0	
Q3	153.0	43.0	43.0	
Max	270	53	270	
	
Baseline ALT toxicity grade							
N	4	5	9	
Grade 0	3 (75.0%)	4 (80.0%)	7 (77.8%)	
Grade 1	0	1 (20.0%)	1 (11.1%)	
Grade 2	0	0	0	
Grade 3	1 (25.0%)	0	1 (11.1%)	
	
HCV geno/subtype (coalesce) b							
N	4	5	9	
1a	0	0	0	
1b	0	0	0	
4a	2 (50.0%)	2 (40.0%)	4 (44.4%)	
4c	1 (25.0%)	0	1 (11.1%)	
4d	1 (25.0%)	2 (40.0%)	3 (33.3%)	
4q	0	1 (20.0%)	1 (11.1%)	
	
HCV geno/subtype (ns5b)							
N	4	5	9	
4a	2 (50.0%)	2 (40.0%)	4 (44.4%)	
4c	1 (25.0%)	0	1 (11.1%)	
4d	1 (25.0%)	2 (40.0%)	3 (33.3%)	
4q	0	1 (20.0%)	1 (11.1%)	
	
HCV geno/subtype (Trugene assay)							
N	0	0	0	
1b	0	0	0	
	
HCV geno/subtype (LIPA 2.0 assay)							
N	4	5	9	
1a	0	0	0	
1b	0	0	0	
4	1 (25.0%)	3 (60.0%)	4 (44.4%)	
4a/4c/4d	3 (75.0%)	2 (40.0%)	5 (55.6%)	
	
HCV geno/subtype as stratified							
N	4	5	9	
1a	0	0	0	
1b	0	0	0	
4	4 (100.0%)	5 (100.0%)	9 (100.0%)	
	
Il28b genotype							
N	4	5	9	
TT	4 (100.0%)	5 (100.0%)	9 (100.0%)	
	
Duration of HCV infection (years)							
N	0	1	1	
Mean		26.30	26.30	
SE				
SD				
95% C.I. *				
Min				
Q1				
Median				
Q3				
Max				
	
Time since diagnosis (years)							
N	4	5	9	
Mean	8.80	10.98	10.01	
SE	3.556	4.942	3.006	
SD	7.112	11.050	9.019	
95% C.I. *	(-2.517; 20.117)	(-2.741; 24.701)	(3.079; 16.944)	
Min	2.2	1.0	1.0	
Q1	2.90	1.30	2.20	
Median	7.95	8.10	8.10	
Q3	14.70	18.20	17.10	
Max	17.1	26.3	26.3	
	
Il28b genotype as stratified							
N	4	5	9	
TT	4 (100.0%)	5 (100.0%)	9 (100.0%)	
	
Mode of hepatitis C infection							
N	4	5	9	
Blood transfusion	0	1 (20.0%)	1 (11.1%)	
Intravenously injectable drug use	1 (25.0%)	1 (20.0%)	2 (22.2%)	
Multiple	0	1 (20.0%)	1 (11.1%)	
Other	3 (75.0%)	2 (40.0%)	5 (55.6%)	
	

* Confidence interval for mean
N = number of subjects with data
a	Results from the Metavir scoring system and the non-invasive methods.
b	HCV Geno/Subtype (Coalesce) is based on the NS5B assay, and if not available on LIPA HCV II or Trugene results.	
[TSIDEM02B.rtf] [\STAT\Analyses\Programs\Interim4\Final4\2.TLF\1.General_IA4\GEN_IA4.sas] 18NOV2014, 15:26	
