# Supplementary material for: Efficacy of a 12-Week Simeprevir Plus Peginterferon/Ribavirin (PR) Regimen in Treatment-Naïve Patients with Hepatitis C Virus (HCV) Genotype 4 (GT4) Infection and Mild-To-Moderate Fibrosis Displaying Early On-Treatment Virologic Response
Source: PLoS One. 2017 Jan 5;12(1):e0168713. doi: 10.1371/journal.pone.0168713 (PMC5215882; doi:10.1371/journal.pone.0168713)
Supplement: S1 Dataset — (ZIP) [file pone.0168713.s002.zip › TSIDS01.rtf]

TSIDS01:	Subjects Screened and Treated; All Subjects (Study TMC435HPC3014)	
	Simeprevir
12Wks
150 mg
PR12/24		
	Unknown	Genotype 4	All Subjects		
Screened	5	89	277		
Screen Failure	5 (100.0%)	22 (24.7%)	45 (16.2%)		
Enrolled/Not treated			2 (0.7%)		
Treated		67 (75.3%)	230 (83.0%)		
	
[TSIDS01.rtf] [\STAT\Analyses\Programs\FinalAnalysis\Final1\2.TLF\1.General\GEN_FA.sas] 23OCT2015, 16:53	
