# Supplementary material for: Efficacy of a 12-Week Simeprevir Plus Peginterferon/Ribavirin (PR) Regimen in Treatment-Naïve Patients with Hepatitis C Virus (HCV) Genotype 4 (GT4) Infection and Mild-To-Moderate Fibrosis Displaying Early On-Treatment Virologic Response
Source: PLoS One. 2017 Jan 5;12(1):e0168713. doi: 10.1371/journal.pone.0168713 (PMC5215882; doi:10.1371/journal.pone.0168713)
Supplement: S1 Dataset — (ZIP) [file pone.0168713.s002.zip › TSIDS05A.rtf]

TSIDS05A:	Completions and Discontinuations of Study Medication and Reasons for Discontinuation; Intent-to-treat (Study TMC435HPC3014)
Treatment Group = Simeprevir 12Wks 150 mg PR12/24	
	Genotype 4			
	12 Weeks 
Treatment	>12 Weeks 
Treatment	All Subjects					
Analysis set: intent-to-treat	34	33	67					
	
Simeprevir								
N	34	33	67					
Completed	34 
(100.0%)	28 
(84.8%)	62 
(92.5%)					
Discontinued		5 
(15.2%)	5 
(7.5%)					
Adverse event a		3 
(9.1%)	3 
(4.5%)					
Subject non-compliant								
Subject reached a virologic endpoint b		2 
(6.1%)	2 
(3.0%)					
Ribavirin								
N	34	33	67					
Completed	34 
(100.0%)	24 
(72.7%)	58 
(86.6%)					
Discontinued		9 
(27.3%)	9 
(13.4%)					
Adverse event a		4 
(12.1%)	4 
(6.0%)					
Subject non-compliant		3 
(9.1%)	3 
(4.5%)					
Subject reached a virologic endpoint b		2 
(6.1%)	2 
(3.0%)					
PegIFN								
N	34	33	67					
Completed	34 
(100.0%)	24 
(72.7%)	58 
(86.6%)					
Discontinued		9 
(27.3%)	9 
(13.4%)					
Adverse event a		4 
(12.1%)	4 
(6.0%)					
Subject non-compliant		3 
(9.1%)	3 
(4.5%)					
Subject reached a virologic endpoint b		2 
(6.1%)	2 
(3.0%)					
	

a	Adverse event: This category may include subjects who stopped Simeprevir because they had to stop RBV and/or PegIFN
	due to an adverse event.
b	Subject reached a virologic endpoint: subject met a virologic stopping rule.
Information presented in the table is based upon 'Treatment Termination' CRF page (investigator's evaluation).	
[TSIDS05A.rtf] [\STAT\Analyses\Programs\FinalAnalysis\Final1\2.TLF\1.General\GEN_FA.sas] 23OCT2015, 16:53	
