# Supplementary material for: Efficacy of a 12-Week Simeprevir Plus Peginterferon/Ribavirin (PR) Regimen in Treatment-Naïve Patients with Hepatitis C Virus (HCV) Genotype 4 (GT4) Infection and Mild-To-Moderate Fibrosis Displaying Early On-Treatment Virologic Response
Source: PLoS One. 2017 Jan 5;12(1):e0168713. doi: 10.1371/journal.pone.0168713 (PMC5215882; doi:10.1371/journal.pone.0168713)
Supplement: S1 Dataset — (ZIP) [file pone.0168713.s002.zip › LEFVF01.rtf]

LEFVF01:	Subjects with Failure; Intent-to-treat (Study TMC435HPC3014)
Treatment Group: Simeprevir 12Wks 150 mg PR12/24	
HCV Geno/subtype	IL28B Genotype	Subject ID	Category 1	Category 2	Category 3	
HCV Genotype: Genotype 4 - Treatment Duration: 12 Weeks Treatment	
4d	CC	30140239	Post-treatment failure	Viral relapse	Completed PegIFN and/or RBV	
	
	
HCV Genotype: Genotype 4 - Treatment Duration: 24 Weeks Treatment	
4a	CT	30140228	On-treatment failure	Did not complete treatment at Week 12, Week 24 or Week 48	Other	
	
	TT	30140201	Post-treatment failure	Viral relapse	Discontinued PegIFN and RBV	
	
	
4d	CT	30140209	On-treatment failure	Did not complete treatment at Week 12, Week 24 or Week 48	Met a stopping rule at Week 4	
		30140220	Post-treatment failure	Viral relapse	Completed PegIFN and/or RBV	
		30140255	On-treatment failure	Did not complete treatment at Week 12, Week 24 or Week 48	Met a stopping rule at Week 4	
	
	
4other	CT	30140214	Post-treatment failure	Viral relapse	Completed PegIFN and/or RBV	
	
	

Subject with CRF ID TMC435HPC3014-0043 achieved HCV RNA <25 iu/ml detected at last study related visit (week 36) after previously having experienced a viral relapse. 
This subject will be further described in the CSR.	
[LEFVF01.rtf] [\STAT\Analyses\Programs\FinalAnalysis\Final1\2.TLF\2.Efficacy\EFF_FA.sas] 23OCT2015, 18:04	
