# Supplementary material for: Efficacy of a 12-Week Simeprevir Plus Peginterferon/Ribavirin (PR) Regimen in Treatment-Naïve Patients with Hepatitis C Virus (HCV) Genotype 4 (GT4) Infection and Mild-To-Moderate Fibrosis Displaying Early On-Treatment Virologic Response
Source: PLoS One. 2017 Jan 5;12(1):e0168713. doi: 10.1371/journal.pone.0168713 (PMC5215882; doi:10.1371/journal.pone.0168713)
Supplement: S1 Dataset — (ZIP) [file pone.0168713.s002.zip › LSIDS01.rtf]

LSIDS01:	Subjects Who Were Not Treated; All Subjects (Study TMC435HPC3014)	
Subject ID	Country	Main Investigator	Reason	
HCV Genotype: Genotype 4	
30140205	Austria	Ferenci, Peter	Subject did not fulfill all inclusion/exclusion criteria	
30140207	France	Asselah, Tarik	Subject withdrew consent	
30140216	France	Asselah, Tarik	Subject did not fulfill all inclusion/exclusion criteria	
30140221	Spain	Diago, Moises	Subject did not fulfill all inclusion/exclusion criteria	
30140224	Belgium	Francque, Sven	Subject withdrew consent	
30140234	Italy	Orlandini, Alessandra	Subject did not fulfill all inclusion/exclusion criteria	
30140235	Italy	Orlandini, Alessandra	Subject withdrew consent	
30140240	Italy	Orlandini, Alessandra	Subject did not fulfill all inclusion/exclusion criteria	
30140242	Italy	Brunetto, Maurizia	Subject withdrew consent	
30140248	Saudi Arabia	Abdo, Ayman	Subject did not fulfill all inclusion/exclusion criteria	
30140249	Saudi Arabia	Al Traif, Ibrahim	Subject did not fulfill all inclusion/exclusion criteria	
30140251	Saudi Arabia	Abdo, Ayman	Subject did not fulfill all inclusion/exclusion criteria	
30140253	Saudi Arabia	Al Traif, Ibrahim	Subject did not fulfill all inclusion/exclusion criteria	
30140260	Saudi Arabia	Al Traif, Ibrahim	Subject did not fulfill all inclusion/exclusion criteria	
30140262	Saudi Arabia	Alashger, Hamad	Subject did not fulfill all inclusion/exclusion criteria	
30140264	Saudi Arabia	Alashger, Hamad	Subject did not fulfill all inclusion/exclusion criteria	
30140269	Saudi Arabia	Alashger, Hamad	Subject did not fulfill all inclusion/exclusion criteria	
30140270	Saudi Arabia	Alashger, Hamad	Subject did not fulfill all inclusion/exclusion criteria	
30140272	Saudi Arabia	Alashger, Hamad	Subject did not fulfill all inclusion/exclusion criteria	
30140274	Saudi Arabia	Abdo, Ayman	Subject did not fulfill all inclusion/exclusion criteria	
30140276	Saudi Arabia	Al Traif, Ibrahim	Subject did not fulfill all inclusion/exclusion criteria	
30140277	Saudi Arabia	Alashger, Hamad	Subject did not fulfill all inclusion/exclusion criteria	
	
[LSIDS01.rtf] [\STAT\Analyses\Programs\FinalAnalysis\Final1\2.TLF\1.General\GEN_FA.sas] 23OCT2015, 16:53	
