# Supplementary material for: Efficacy of a 12-Week Simeprevir Plus Peginterferon/Ribavirin (PR) Regimen in Treatment-Naïve Patients with Hepatitis C Virus (HCV) Genotype 4 (GT4) Infection and Mild-To-Moderate Fibrosis Displaying Early On-Treatment Virologic Response
Source: PLoS One. 2017 Jan 5;12(1):e0168713. doi: 10.1371/journal.pone.0168713 (PMC5215882; doi:10.1371/journal.pone.0168713)
Supplement: S1 Dataset — (ZIP) [file pone.0168713.s002.zip › TEFSUB01.rtf]

TEFSUB01:	Sustained Virologic Response 12 Weeks After the Planned End of Treatment - Subgroup Analyses; Intent-to-treat (Study TMC435HPC3014)
1) HCV Geno/subtype 
Treatment Group = Simeprevir 12Wks 150 mg PR12/24	
	Genotype 4		
	12 Weeks 
Treatment	>12 Weeks 
Treatment	All Subjects		
Analysis set: intent-to-treata								
	34	33	67					
	
Sustained Virologic Response 12 Weeks after EOT								
1a/other								
n/N (%)	-	-	-					
95% CI	-	-	-					
1b								
n/N (%)	-	-	-					
95% CI	-	-	-					
4a								
n/N (%)	14/ 14 
( 100.0%)	11/ 13 
( 84.6%)	25/ 27 
( 92.6%)					
95% CI	(100.00; 100.00)	(65.00; 100.00)	(82.71; 100.00)					
4d								
n/N (%)	12/ 13 
( 92.3%)	9/ 12 
( 75.0%)	21/ 25 
( 84.0%)					
95% CI	(77.82; 100.00)	(50.50; 99.50)	(69.63; 98.37)					
4other								
n/N (%)	7/  7 
( 100.0%)	7/  8 
( 87.5%)	14/ 15 
( 93.3%)					
95% CI	(100.00; 100.00)	(64.58; 100.00)	(80.71; 100.00)					
	


a Number of ITT subjects that reached 12 weeks after planned EOT	
[TEFSUB01.rtf] [\STAT\Analyses\Programs\FinalAnalysis\Final1\2.TLF\2.Efficacy\EFF_FA.sas] 23OCT2015, 18:04	

TEFSUB01:	Sustained Virologic Response 12 Weeks After the Planned End of Treatment - Subgroup Analyses; Intent-to-treat (Study TMC435HPC3014)
2) IL28B Genotype 
Treatment Group = Simeprevir 12Wks 150 mg PR12/24	
	Genotype 4		
	12 Weeks 
Treatment	>12 Weeks 
Treatment	All Subjects		
Analysis set: intent-to-treata								
	34	33	67					
	
Sustained Virologic Response 12 Weeks after EOT								
CC								
n/N (%)	13/ 14 
( 92.9%)	1/  1 
( 100.0%)	14/ 15 
( 93.3%)					
95% CI	(79.37; 100.00)	-	(80.71; 100.00)					
CT								
n/N (%)	15/ 15 
( 100.0%)	22/ 27 
( 81.5%)	37/ 42 
( 88.1%)					
95% CI	(100.00; 100.00)	(66.83; 96.13)	(78.30; 97.89)					
TT								
n/N (%)	5/  5 
( 100.0%)	4/  5 
( 80.0%)	9/ 10 
( 90.0%)					
95% CI	(100.00; 100.00)	(44.94; 100.00)	(71.41; 100.00)					
	


a Number of ITT subjects that reached 12 weeks after planned EOT	
[TEFSUB01.rtf] [\STAT\Analyses\Programs\FinalAnalysis\Final1\2.TLF\2.Efficacy\EFF_FA.sas] 23OCT2015, 18:04	

TEFSUB01:	Sustained Virologic Response 12 Weeks After the Planned End of Treatment - Subgroup Analyses; Intent-to-treat (Study TMC435HPC3014)
3) Sex 
Treatment Group = Simeprevir 12Wks 150 mg PR12/24	
	Genotype 4			
	12 Weeks 
Treatment	>12 Weeks 
Treatment	All Subjects					
Analysis set: intent-to-treata								
	34	33	67					
	
Sustained Virologic Response 12 Weeks after EOT								
Female								
n/N (%)	11/ 11 
( 100.0%)	8/ 10 
( 80.0%)	19/ 21 
( 90.5%)					
95% CI	(100.00; 100.00)	(55.21; 100.00)	(77.92; 100.00)					
Male								
n/N (%)	22/ 23 
( 95.7%)	19/ 23 
( 82.6%)	41/ 46 
( 89.1%)					
95% CI	(87.32; 100.00)	(67.12; 98.10)	(80.14; 98.13)					
	


a Number of ITT subjects that reached 12 weeks after planned EOT	
[TEFSUB01.rtf] [\STAT\Analyses\Programs\FinalAnalysis\Final1\2.TLF\2.Efficacy\EFF_FA.sas] 23OCT2015, 18:04	

TEFSUB01:	Sustained Virologic Response 12 Weeks After the Planned End of Treatment - Subgroup Analyses; Intent-to-treat (Study TMC435HPC3014)
4) Race 
Treatment Group = Simeprevir 12Wks 150 mg PR12/24	
	Genotype 4		
	12 Weeks 
Treatment	>12 Weeks 
Treatment	All Subjects		
Analysis set: intent-to-treata								
	34	33	67					
							
Sustained Virologic Response 12 Weeks after EOT								
Caucasian								
n/N (%)	22/ 23 
( 95.7%)	18/ 24 
( 75.0%)	40/ 47 
( 85.1%)					
95% CI	(87.32; 100.00)	(57.68; 92.32)	(74.93; 95.28)					
Black								
n/N (%)	3/  3 
( 100.0%)	4/  4 
( 100.0%)	7/  7 
( 100.0%)					
95% CI	(100.00; 100.00)	(100.00; 100.00)	(100.00; 100.00)					
Asian								
n/N (%)	2/  2 
( 100.0%)	1/  1 
( 100.0%)	3/  3 
( 100.0%)					
95% CI	(100.00; 100.00)	-	(100.00; 100.00)					
Other								
n/N (%)	2/  2 
( 100.0%)	-	2/  2 
( 100.0%)					
95% CI	(100.00; 100.00)	-	(100.00; 100.00)					
Unknown								
n/N (%)	4/  4 
( 100.0%)	4/  4 
( 100.0%)	8/  8 
( 100.0%)					
95% CI	(100.00; 100.00)	(100.00; 100.00)	(100.00; 100.00)					
	


a Number of ITT subjects that reached 12 weeks after planned EOT	
	

TEFSUB01:	Sustained Virologic Response 12 Weeks After the Planned End of Treatment - Subgroup Analyses; Intent-to-treat (Study TMC435HPC3014)
5) Origin 
Treatment Group = Simeprevir 12Wks 150 mg PR12/24	
	Genotype 4		
	12 Weeks 
Treatment	>12 Weeks 
Treatment	All Subjects		
Analysis set: intent-to-treata					
	34	33	67		
	
Sustained Virologic Response 12 Weeks after EOT					
Europe					
n/N (%)	15/ 16 
( 93.8%)	11/ 16 
( 68.8%)	26/ 32 
( 81.3%)		
95% CI	(81.89; 100.00)	(46.04; 91.46)	(67.73; 94.77)		
Middle-East/North-Africa					
n/N (%)	7/  7 
( 100.0%)	7/  8 
( 87.5%)	14/ 15 
( 93.3%)		
95% CI	(100.00; 100.00)	(64.58; 100.00)	(80.71; 100.00)		
Other regions					
n/N (%)	1/  1 
( 100.0%)	-	1/  1 
( 100.0%)		
Missing					
n/N (%)	10/ 10 
( 100.0%)	9/  9 
( 100.0%)	19/ 19 
( 100.0%)		
95% CI	(100.00; 100.00)	(100.00; 100.00)	(100.00; 100.00)		
	


a Number of ITT subjects that reached 12 weeks after planned EOT	
[TEFSUB01.rtf] [\STAT\Analyses\Programs\FinalAnalysis\Final1\2.TLF\2.Efficacy\EFF_FA.sas] 23OCT2015, 18:04	

TEFSUB01:	Sustained Virologic Response 12 Weeks After the Planned End of Treatment - Subgroup Analyses; Intent-to-treat (Study TMC435HPC3014)
6) Country 
Treatment Group = Simeprevir 12Wks 150 mg PR12/24	
	Genotype 4			
	12 Weeks 
Treatment	>12 Weeks 
Treatment	All Subjects					
Analysis set: intent-to-treata								
	34	33	67					
	
Sustained Virologic Response 12 Weeks after EOT								
Austria								
n/N (%)	3/  3 
( 100.0%)	5/  5 
( 100.0%)	8/  8 
( 100.0%)					
95% CI	(100.00; 100.00)	(100.00; 100.00)	(100.00; 100.00)					
Belgium								
n/N (%)	3/  3 
( 100.0%)	5/  5 
( 100.0%)	8/  8 
( 100.0%)					
95% CI	(100.00; 100.00)	(100.00; 100.00)	(100.00; 100.00)					
France								
n/N (%)	6/  6 
( 100.0%)	6/  7 
( 85.7%)	12/ 13 
( 92.3%)					
95% CI	(100.00; 100.00)	(59.79; 100.00)	(77.82; 100.00)					
Germany								
n/N (%)	-	-	-					
95% CI	-	-	-					
Italy								
n/N (%)	4/  4 
( 100.0%)	3/  4 
( 75.0%)	7/  8 
( 87.5%)					
95% CI	(100.00; 100.00)	(32.57; 100.00)	(64.58; 100.00)					
Saudi Arabia								
n/N (%)	10/ 10 
( 100.0%)	6/  7 
( 85.7%)	16/ 17 
( 94.1%)					
95% CI	(100.00; 100.00)	(59.79; 100.00)	(82.93; 100.00)					
Spain								
n/N (%)	7/  8 
( 87.5%)	2/  5 
( 40.0%)	9/ 13 
( 69.2%)					
95% CI	(64.58; 100.00)	(0.00; 82.94)	(44.14; 94.32)					
United Kingdom								
n/N (%)	-	-	-					
95% CI	-	-	-					
	


a Number of ITT subjects that reached 12 weeks after planned EOT	
[TEFSUB01.rtf] [\STAT\Analyses\Programs\FinalAnalysis\Final1\2.TLF\2.Efficacy\EFF_FA.sas] 23OCT2015, 18:04	

TEFSUB01:	Sustained Virologic Response 12 Weeks After the Planned End of Treatment - Subgroup Analyses; Intent-to-treat (Study TMC435HPC3014)
7) Baseline BMI 
Treatment Group = Simeprevir 12Wks 150 mg PR12/24	
	Genotype 4		
	12 Weeks 
Treatment	>12 Weeks 
Treatment	All Subjects		
Analysis set: intent-to-treata								
	34	33	67					
	
Sustained Virologic Response 12 Weeks after EOT								
<25 kg/m2								
n/N (%)	17/ 18 
( 94.4%)	5/  9 
( 55.6%)	22/ 27 
( 81.5%)					
95% CI	(83.86; 100.00)	(23.09; 88.02)	(66.83; 96.13)					
>=25 - <30 kg/m2								
n/N (%)	12/ 12 
( 100.0%)	11/ 12 
( 91.7%)	23/ 24 
( 95.8%)					
95% CI	(100.00; 100.00)	(76.03; 100.00)	(87.84; 100.00)					
>=30 kg/m2								
n/N (%)	4/  4 
( 100.0%)	11/ 12 
( 91.7%)	15/ 16 
( 93.8%)					
95% CI	(100.00; 100.00)	(76.03; 100.00)	(81.89; 100.00)					
	


a Number of ITT subjects that reached 12 weeks after planned EOT	
[TEFSUB01.rtf] [\STAT\Analyses\Programs\FinalAnalysis\Final1\2.TLF\2.Efficacy\EFF_FA.sas] 23OCT2015, 18:04	

TEFSUB01:	Sustained Virologic Response 12 Weeks After the Planned End of Treatment - Subgroup Analyses; Intent-to-treat (Study TMC435HPC3014)
8) Baseline HCV RNA 
Treatment Group = Simeprevir 12Wks 150 mg PR12/24	
	Genotype 4		
	12 Weeks 
Treatment	>12 Weeks 
Treatment	All Subjects		
Analysis set: intent-to-treata								
	34	33	67					
	
Sustained Virologic Response 12 Weeks after EOT								
<=800000 IU/mL								
n/N (%)	19/ 19 
( 100.0%)	6/  7 
( 85.7%)	25/ 26 
( 96.2%)					
95% CI	(100.00; 100.00)	(59.79; 100.00)	(88.76; 100.00)					
>800000 IU/mL								
n/N (%)	14/ 15 
( 93.3%)	21/ 26 
( 80.8%)	35/ 41 
( 85.4%)					
95% CI	(80.71; 100.00)	(65.62; 95.92)	(74.55; 96.18)					
	


a Number of ITT subjects that reached 12 weeks after planned EOT	
[TEFSUB01.rtf] [\STAT\Analyses\Programs\FinalAnalysis\Final1\2.TLF\2.Efficacy\EFF_FA.sas] 23OCT2015, 18:04	

TEFSUB01:	Sustained Virologic Response 12 Weeks After the Planned End of Treatment - Subgroup Analyses; Intent-to-treat (Study TMC435HPC3014)
9) Metavir Score 
Treatment Group = Simeprevir 12Wks 150 mg PR12/24	
	Genotype 4		
	12 Weeks 
Treatment	>12 Weeks 
Treatment	All Subjects		
Analysis set: intent-to-treata								
	34	33	67					
	
Sustained Virologic Response 12 Weeks after EOT								
Score F0-F1								
n/N (%)	28/ 29 
( 96.6%)	20/ 25 
( 80.0%)	48/ 54 
( 88.9%)					
95% CI	(89.91; 100.00)	(64.32; 95.68)	(80.51; 97.27)					
Score F2								
n/N (%)	5/  5 
( 100.0%)	6/  7 
( 85.7%)	11/ 12 
( 91.7%)					
95% CI	(100.00; 100.00)	(59.79; 100.00)	(76.03; 100.00)					
Score F3								
n/N (%)	-	1/  1 
( 100.0%)	1/  1 
( 100.0%)					
Missing								
n/N (%)	-	-	-					
	


a Number of ITT subjects that reached 12 weeks after planned EOT	
[TEFSUB01.rtf] [\STAT\Analyses\Programs\FinalAnalysis\Final1\2.TLF\2.Efficacy\EFF_FA.sas] 23OCT2015, 18:04	

TEFSUB01:	Sustained Virologic Response 12 Weeks After the Planned End of Treatment - Subgroup Analyses; Intent-to-treat (Study TMC435HPC3014)
10) Baseline Q80K Mutation 
Treatment Group = Simeprevir 12Wks 150 mg PR12/24	
	Genotype 4		
	12 Weeks 
Treatment	>12 Weeks 
Treatment	All Subjects		
Analysis set: intent-to-treata								
	34	33	67					
	
Sustained Virologic Response 12 Weeks after EOT								
Q80K								
n/N (%)	-	-	-					
95% CI	-	-	-					
No Q80K								
n/N (%)	26/ 27 
( 96.3%)	17/ 22 
( 77.3%)	43/ 49 
( 87.8%)					
95% CI	(89.17; 100.00)	(59.76; 94.78)	(78.58; 96.93)					
Missing								
n/N (%)	7/  7 
( 100.0%)	10/ 11 
( 90.9%)	17/ 18 
( 94.4%)					
95% CI	(100.00; 100.00)	(73.92; 100.00)	(83.86; 100.00)					
	


a Number of ITT subjects that reached 12 weeks after planned EOT	
[TEFSUB01.rtf] [\STAT\Analyses\Programs\FinalAnalysis\Final1\2.TLF\2.Efficacy\EFF_FA.sas] 23OCT2015, 18:04	

TEFSUB01:	Sustained Virologic Response 12 Weeks After the Planned End of Treatment - Subgroup Analyses; Intent-to-treat (Study TMC435HPC3014)
11) Mode of HCV Infection 
Treatment Group = Simeprevir 12Wks 150 mg PR12/24	
	Genotype 4		
	12 Weeks 
Treatment	>12 Weeks 
Treatment	All Subjects		
Analysis set: intent-to-treata								
	34	33	67					
	
Sustained Virologic Response 12 Weeks after EOT								
Blood Transfusion								
n/N (%)	4/  4 
( 100.0%)	6/  6 
( 100.0%)	10/ 10 
( 100.0%)					
95% CI	(100.00; 100.00)	(100.00; 100.00)	(100.00; 100.00)					
Hemophilia-Associated Injections								
n/N (%)	-	-	-					
Heterosexual Contact								
n/N (%)	-	-	-					
95% CI	-	-	-					
Intravenously Injectable Drug Use								
n/N (%)	5/  5 
( 100.0%)	4/  4 
( 100.0%)	9/  9 
( 100.0%)					
95% CI	(100.00; 100.00)	(100.00; 100.00)	(100.00; 100.00)					
Mother To Child Transmission								
n/N (%)	3/  3 
( 100.0%)	1/  1 
( 100.0%)	4/  4 
( 100.0%)					
95% CI	(100.00; 100.00)	-	(100.00; 100.00)					
Multiple								
n/N (%)	-	1/  1 
( 100.0%)	1/  1 
( 100.0%)					
95% CI	-	-	-					
Other								
n/N (%)	21/ 22 
( 95.5%)	15/ 21 
( 71.4%)	36/ 43 
( 83.7%)					
95% CI	(86.75; 100.00)	(52.11; 90.75)	(72.69; 94.76)					
	


a Number of ITT subjects that reached 12 weeks after planned EOT	
[TEFSUB01.rtf] [\STAT\Analyses\Programs\FinalAnalysis\Final1\2.TLF\2.Efficacy\EFF_FA.sas] 23OCT2015, 18:04	
