# Supplementary material for: Efficacy of a 12-Week Simeprevir Plus Peginterferon/Ribavirin (PR) Regimen in Treatment-Naïve Patients with Hepatitis C Virus (HCV) Genotype 4 (GT4) Infection and Mild-To-Moderate Fibrosis Displaying Early On-Treatment Virologic Response
Source: PLoS One. 2017 Jan 5;12(1):e0168713. doi: 10.1371/journal.pone.0168713 (PMC5215882; doi:10.1371/journal.pone.0168713)
Supplement: S1 Dataset — (ZIP) [file pone.0168713.s002.zip › TEFSUB02.rtf]

TEFSUB02:	Sustained Virologic Response 12 Weeks After the Planned End of Treatment - Subgroup Analyses for Early Response Parameters; Intent-to-treat (Study TMC435HPC3014)
1) Virologic Response at Week 2 
Treatment Group = Simeprevir 12Wks 150 mg PR12/24	
	Genotype 4			
	12 Weeks 
Treatment	>12 Weeks 
Treatment	All Subjects					
Analysis set: intent-to-treata								
	34	33	67					
	
Sustained Virologic Response 12 Weeks after EOT								
< 25 undetectable								
n/N (%)	31/ 32 
( 96.9%)	-	31/ 32 
( 96.9%)					
95% CI	(90.85; 100.00)	-	(90.85; 100.00)					
< 25 detectable								
n/N (%)	2/  2 
( 100.0%)	20/ 22 
( 90.9%)	22/ 24 
( 91.7%)					
95% CI	(100.00; 100.00)	(78.90; 100.00)	(80.61; 100.00)					
>= 25 IU/mL								
n/N (%)	-	6/  9 
( 66.7%)	6/  9 
( 66.7%)					
95% CI	-	(35.87; 97.46)	(35.87; 97.46)					
Missing								
n/N (%)	-	1/  2 
( 50.0%)	1/  2 
( 50.0%)					
95% CI	-	(0.00; 100.00)	(0.00; 100.00)					
	


a Number of ITT subjects that reached 12 weeks after planned EOT	
[TEFSUB02.rtf] [\STAT\Analyses\Programs\FinalAnalysis\Final1\2.TLF\2.Efficacy\EFF_FA.sas] 23OCT2015, 18:04	

TEFSUB02:	Sustained Virologic Response 12 Weeks After the Planned End of Treatment - Subgroup Analyses for Early Response Parameters; Intent-to-treat (Study TMC435HPC3014)
2) Virologic Response at Week 4 
Treatment Group = Simeprevir 12Wks 150 mg PR12/24	
	Genotype 4			
	12 Weeks 
Treatment	>12 Weeks 
Treatment	All Subjects					
Analysis set: intent-to-treata								
	34	33	67					
	
Sustained Virologic Response 12 Weeks after EOT								
< 25 undetectable								
n/N (%)	33/ 34 
( 97.1%)	21/ 24 
( 87.5%)	54/ 58 
( 93.1%)					
95% CI	(91.38; 100.00)	(74.27; 100.00)	(86.58; 99.62)					
< 25 detectable								
n/N (%)	-	6/  6 
( 100.0%)	6/  6 
( 100.0%)					
95% CI	-	(100.00; 100.00)	(100.00; 100.00)					
>= 25 IU/mL								
n/N (%)	-	0/  2 
(  0.0%)	0/  2 
(  0.0%)					
Missing								
n/N (%)	-	0/  1 
(  0.0%)	0/  1 
(  0.0%)					
	


a Number of ITT subjects that reached 12 weeks after planned EOT	
[TEFSUB02.rtf] [\STAT\Analyses\Programs\FinalAnalysis\Final1\2.TLF\2.Efficacy\EFF_FA.sas] 23OCT2015, 18:04	

TEFSUB02:	Sustained Virologic Response 12 Weeks After the Planned End of Treatment - Subgroup Analyses for Early Response Parameters; Intent-to-treat (Study TMC435HPC3014)
3) Hgb Reduction from Baseline at Week 12 
Treatment Group = Simeprevir 12Wks 150 mg PR12/24	
	Genotype 4			
	12 Weeks 
Treatment	>12 Weeks 
Treatment	All Subjects					
Analysis set: intent-to-treata								
	34	33	67					
	
Sustained Virologic Response 12 Weeks after EOT								
<10 g/L								
n/N (%)	-	2/  4 
( 50.0%)	2/  4 
( 50.0%)					
95% CI	-	(1.00; 99.00)	(1.00; 99.00)					
10-19 g/L								
n/N (%)	9/  9 
( 100.0%)	6/  6 
( 100.0%)	15/ 15 
( 100.0%)					
95% CI	(100.00; 100.00)	(100.00; 100.00)	(100.00; 100.00)					
20-29 g/L								
n/N (%)	9/  9 
( 100.0%)	8/  8 
( 100.0%)	17/ 17 
( 100.0%)					
95% CI	(100.00; 100.00)	(100.00; 100.00)	(100.00; 100.00)					
>=30 g/L								
n/N (%)	13/ 14 
( 92.9%)	10/ 10 
( 100.0%)	23/ 24 
( 95.8%)					
95% CI	(79.37; 100.00)	(100.00; 100.00)	(87.84; 100.00)					
Missing								
n/N (%)	2/  2 
( 100.0%)	1/  5 
( 20.0%)	3/  7 
( 42.9%)					
95% CI	(100.00; 100.00)	(0.00; 55.06)	(6.20; 79.52)					
	


a Number of ITT subjects that reached 12 weeks after planned EOT	
[TEFSUB02.rtf] [\STAT\Analyses\Programs\FinalAnalysis\Final1\2.TLF\2.Efficacy\EFF_FA.sas] 23OCT2015, 18:04	
