# Supplementary material for: Efficacy of a 12-Week Simeprevir Plus Peginterferon/Ribavirin (PR) Regimen in Treatment-Naïve Patients with Hepatitis C Virus (HCV) Genotype 4 (GT4) Infection and Mild-To-Moderate Fibrosis Displaying Early On-Treatment Virologic Response
Source: PLoS One. 2017 Jan 5;12(1):e0168713. doi: 10.1371/journal.pone.0168713 (PMC5215882; doi:10.1371/journal.pone.0168713)
Supplement: S1 Dataset — (ZIP) [file pone.0168713.s002.zip › TEFSUB04C.rtf]

TEFSUB04C:	Efficacy Endpoints - by IL28B; Intent-to-treat (Study TMC435HPC3014)
Treatment Group = Simeprevir 12Wks 150 mg PR12/24	
	Genotype 4		
	12 Weeks 
Treatment	>12 Weeks 
Treatment	All Subjects		
Analysis set: intent-to-treat	34	33	67		
	
01) Sustained Virologic Response 4 Weeks after EOT								
CC	14/ 14 
( 100.0%)	1/  1 
( 100.0%)	15/ 15 
( 100.0%)		
CT	15/ 15 
( 100.0%)	24/ 27 
( 88.9%)	39/ 42 
( 92.9%)		
TT	5/  5 
( 100.0%)	4/  5 
( 80.0%)	9/ 10 
( 90.0%)		
	
02) Sustained Virologic Response 12 Weeks after EOT								
CC	13/ 14 
( 92.9%)	1/  1 
( 100.0%)	14/ 15 
( 93.3%)		
CT	15/ 15 
( 100.0%)	22/ 27 
( 81.5%)	37/ 42 
( 88.1%)		
TT	5/  5 
( 100.0%)	4/  5 
( 80.0%)	9/ 10 
( 90.0%)		
	
03) Sustained Virologic Response 24 Weeks after EOT								
CC	13/ 14 
( 92.9%)	1/  1 
( 100.0%)	14/ 15 
( 93.3%)		
CT	15/ 15 
( 100.0%)	22/ 27 
( 81.5%)	37/ 42 
( 88.1%)		
TT	5/  5 
( 100.0%)	4/  5 
( 80.0%)	9/ 10 
( 90.0%)		
	
04a) Virologic Response at Week 2
< 25 undetectable								
CC	12/ 14 
( 85.7%)	0/  1 
(  0.0%)	12/ 15 
( 80.0%)		
CT	15/ 15 
( 100.0%)	0/ 25 
(  0.0%)	15/ 40 
( 37.5%)		
TT	5/  5 
( 100.0%)	0/  5 
(  0.0%)	5/ 10 
( 50.0%)		
	
04b) Virologic Response at Week 2
< 25 detectable								
CC	2/ 14 
( 14.3%)	1/  1 
( 100.0%)	3/ 15 
( 20.0%)		
CT	0/ 15 
(  0.0%)	17/ 25 
( 68.0%)	17/ 40 
( 42.5%)		
TT	0/  5 
(  0.0%)	4/  5 
( 80.0%)	4/ 10 
( 40.0%)		
	
04c) Virologic Response at Week 2
>= 25 IU/mL								
CC	0/ 14 
(  0.0%)	0/  1 
(  0.0%)	0/ 15 
(  0.0%)		
CT	0/ 15 
(  0.0%)	8/ 25 
( 32.0%)	8/ 40 
( 20.0%)		
TT	0/  5 
(  0.0%)	1/  5 
( 20.0%)	1/ 10 
( 10.0%)		
	
05a) Virologic Response at Week 4
< 25 undetectable								
CC	14/ 14 
( 100.0%)	0/  1 
(  0.0%)	14/ 15 
( 93.3%)		
CT	15/ 15 
( 100.0%)	20/ 26 
( 76.9%)	35/ 41 
( 85.4%)		
TT	5/  5 
( 100.0%)	4/  5 
( 80.0%)	9/ 10 
( 90.0%)		
	
05b) Virologic Response at Week 4
< 25 detectable								
CC	0/ 14 
(  0.0%)	1/  1 
( 100.0%)	1/ 15 
(  6.7%)		
CT	0/ 15 
(  0.0%)	4/ 26 
( 15.4%)	4/ 41 
(  9.8%)		
TT	0/  5 
(  0.0%)	1/  5 
( 20.0%)	1/ 10 
( 10.0%)		
	
05c) Virologic Response at Week 4
>= 25 IU/mL								
CC	0/ 14 
(  0.0%)	0/  1 
(  0.0%)	0/ 15 
(  0.0%)		
CT	0/ 15 
(  0.0%)	2/ 26 
(  7.7%)	2/ 41 
(  4.9%)		
TT	0/  5 
(  0.0%)	0/  5 
(  0.0%)	0/ 10 
(  0.0%)		
	
06) <25 IU/mL undetectable at EOTb								
CC	14/ 14 
( 100.0%)	1/  1 
( 100.0%)	15/ 15 
( 100.0%)		
CT	15/ 15 
( 100.0%)	24/ 27 
( 88.9%)	39/ 42 
( 92.9%)		
TT	5/  5 
( 100.0%)	5/  5 
( 100.0%)	10/ 10 
( 100.0%)		
	
07) Viral Relapse								
CC	1/ 14 
(  7.1%)	0/  1 
(  0.0%)	1/ 15 
(  6.7%)		
CT	0/ 15 
(  0.0%)	2/ 24 
(  8.3%)	2/ 39 
(  5.1%)		
TT	0/  5 
(  0.0%)	1/  5 
( 20.0%)	1/ 10 
( 10.0%)		
	
08) Viral Breakthrougha								
CC	0/ 14 
(  0.0%)	0/  1 
(  0.0%)	0/ 15 
(  0.0%)		
CT	0/ 15 
(  0.0%)	1/ 26 
(  3.8%)	1/ 41 
(  2.4%)		
TT	0/  5 
(  0.0%)	0/  5 
(  0.0%)	0/ 10 
(  0.0%)		
	

a A subject can both meet a stopping rule and have a viral breakthrough 
b Undetectable at EOT or unconfirmed detectable at EOT (these are all subjects that are not on-treatment failures).
Subject with CRF ID TMC435HPC3014-0043 achieved HCV RNA <25 iu/ml detected at last study related visit (week 36) 
after previously having experienced a viral relapse. This subject will be further described in the CSR.	
[TEFSUB04C.rtf] [\STAT\Analyses\Programs\FinalAnalysis\Final1\2.TLF\2.Efficacy\EFF_FA.sas] 23OCT2015, 18:04	
