# Supplementary material for: Efficacy of a 12-Week Simeprevir Plus Peginterferon/Ribavirin (PR) Regimen in Treatment-Naïve Patients with Hepatitis C Virus (HCV) Genotype 4 (GT4) Infection and Mild-To-Moderate Fibrosis Displaying Early On-Treatment Virologic Response
Source: PLoS One. 2017 Jan 5;12(1):e0168713. doi: 10.1371/journal.pone.0168713 (PMC5215882; doi:10.1371/journal.pone.0168713)
Supplement: S1 Dataset — (ZIP) [file pone.0168713.s002.zip › TEFSVR02aA.rtf]

TEFSVR02aA:	Primary Endpoint: Sustained Virologic Response 12 Weeks After the Planned End of Treatment (SVR12); Intent-to-treat (Study TMC435HPC3014)
Treatment Group = Simeprevir 12Wks 150 mg PR12/24	
	Genotype 4		
	12 Weeks 
Treatment	>12 Weeks 
Treatment	All Subjects		
Analysis set: intent-to-treat					
	34	33	67		
	
SVR12								
Yes						
n/N (%)	33/ 34 
( 97.1%)	27/ 33 
( 81.8%)	60/ 67 
( 89.6%)		
95% CI	(91.38; 100.00)	(68.66; 94.98)	(82.23; 96.88)		
	
	
[TEFSVR02aA.rtf] [\STAT\Analyses\Programs\FinalAnalysis\Final1\2.TLF\2.Efficacy\EFF_FA.sas] 23OCT2015, 18:04	
