# Supplementary material for: Efficacy of a 12-Week Simeprevir Plus Peginterferon/Ribavirin (PR) Regimen in Treatment-Naïve Patients with Hepatitis C Virus (HCV) Genotype 4 (GT4) Infection and Mild-To-Moderate Fibrosis Displaying Early On-Treatment Virologic Response
Source: PLoS One. 2017 Jan 5;12(1):e0168713. doi: 10.1371/journal.pone.0168713 (PMC5215882; doi:10.1371/journal.pone.0168713)
Supplement: S1 Dataset — (ZIP) [file pone.0168713.s002.zip › TEFSVR03.rtf]

TEFSVR03:	Sustained Virologic Response, 4, 12 and 24 Weeks After the Planned End of Treatment - Details; Intent-to-treat (Study TMC435HPC3014)
Treatment Group = Simeprevir 12Wks 150 mg PR12/24	
	Genotype 4		
	12 Weeks 
Treatment	>12 Weeks 
Treatment	All Subjects	
Analysis set: intent-to-treat	34	33	67				
	
SVR4							
Total with SVR4	34/ 34 
( 100.0%)	29/ 33 
( 87.9%)	63/ 67 
( 94.0%)				
Total without SVR4 for any reason	0/ 34 
(  0.0%)	4/ 33 
( 12.1%)	4/ 67 
(  6.0%)				
Detectable at EOT	0/ 34 
(  0.0%)	3/ 33 
(  9.1%)	3/ 67 
(  4.5%)				
Discontinued at least one study therapy (any other case)	0/ 34 
(  0.0%)	3/ 33 
(  9.1%)	3/ 67 
(  4.5%)				
Adverse event	0/ 34 
(  0.0%)	1/ 33 
(  3.0%)	1/ 67 
(  1.5%)				
Subject reached a virologic endpoint	0/ 34 
(  0.0%)	2/ 33 
(  6.1%)	2/ 67 
(  3.0%)				
Undetectable at EOTa	0/ 34 
(  0.0%)	1/ 33 
(  3.0%)	1/ 67 
(  1.5%)				
Completed all study therapy	0/ 34 
(  0.0%)	0/ 33 
(  0.0%)	0/ 67 
(  0.0%)				
Discontinued at least one study therapy (any other case)	0/ 34 
(  0.0%)	1/ 33 
(  3.0%)	1/ 67 
(  1.5%)				
Adverse event	0/ 34 
(  0.0%)	1/ 33 
(  3.0%)	1/ 67 
(  1.5%)				
Other	0/ 34 
(  0.0%)	0/ 33 
(  0.0%)	0/ 67 
(  0.0%)				
Subject reached a virologic endpoint	0/ 34 
(  0.0%)	0/ 33 
(  0.0%)	0/ 67 
(  0.0%)				
HCV RNA >= 25 at Timepoint of SVR	0/ 34 
(  0.0%)	1/ 33 
(  3.0%)	1/ 67 
(  1.5%)				
Completed all study therapy	0/ 34 
(  0.0%)	0/ 33 
(  0.0%)	0/ 67 
(  0.0%)				
Discontinued at least one study therapy (any other case)	0/ 34 
(  0.0%)	1/ 33 
(  3.0%)	1/ 67 
(  1.5%)				
Adverse event	0/ 34 
(  0.0%)	1/ 33 
(  3.0%)	1/ 67 
(  1.5%)				
Other	0/ 34 
(  0.0%)	0/ 33 
(  0.0%)	0/ 67 
(  0.0%)				
Subject reached a virologic endpoint	0/ 34 
(  0.0%)	0/ 33 
(  0.0%)	0/ 67 
(  0.0%)				
Missing at Timepoint of SVR	0/ 34 
(  0.0%)	0/ 33 
(  0.0%)	0/ 67 
(  0.0%)				
Completed all study therapy	0/ 34 
(  0.0%)	0/ 33 
(  0.0%)	0/ 67 
(  0.0%)				
	
SVR12							
Total with SVR12	33/ 34 
( 97.1%)	27/ 33 
( 81.8%)	60/ 67 
( 89.6%)				
Total without SVR12 for any reason	1/ 34 
(  2.9%)	6/ 33 
( 18.2%)	7/ 67 
( 10.4%)				
Detectable at EOT	0/ 34 
(  0.0%)	3/ 33 
(  9.1%)	3/ 67 
(  4.5%)				
Discontinued at least one study therapy (any other case)	0/ 34 
(  0.0%)	3/ 33 
(  9.1%)	3/ 67 
(  4.5%)				
Adverse event	0/ 34 
(  0.0%)	1/ 33 
(  3.0%)	1/ 67 
(  1.5%)				
Subject reached a virologic endpoint	0/ 34 
(  0.0%)	2/ 33 
(  6.1%)	2/ 67 
(  3.0%)				
Undetectable at EOTa	1/ 34 
(  2.9%)	3/ 33 
(  9.1%)	4/ 67 
(  6.0%)				
Completed all study therapy	1/ 34 
(  2.9%)	2/ 33 
(  6.1%)	3/ 67 
(  4.5%)				
Discontinued at least one study therapy (any other case)	0/ 34 
(  0.0%)	1/ 33 
(  3.0%)	1/ 67 
(  1.5%)				
Adverse event	0/ 34 
(  0.0%)	1/ 33 
(  3.0%)	1/ 67 
(  1.5%)				
Other	0/ 34 
(  0.0%)	0/ 33 
(  0.0%)	0/ 67 
(  0.0%)				
Subject reached a virologic endpoint	0/ 34 
(  0.0%)	0/ 33 
(  0.0%)	0/ 67 
(  0.0%)				
HCV RNA >= 25 at Timepoint of SVR	1/ 34 
(  2.9%)	3/ 33 
(  9.1%)	4/ 67 
(  6.0%)				
Completed all study therapy	1/ 34 
(  2.9%)	2/ 33 
(  6.1%)	3/ 67 
(  4.5%)				
Discontinued at least one study therapy (any other case)	0/ 34 
(  0.0%)	1/ 33 
(  3.0%)	1/ 67 
(  1.5%)				
Adverse event	0/ 34 
(  0.0%)	1/ 33 
(  3.0%)	1/ 67 
(  1.5%)				
Other	0/ 34 
(  0.0%)	0/ 33 
(  0.0%)	0/ 67 
(  0.0%)				
Subject reached a virologic endpoint	0/ 34 
(  0.0%)	0/ 33 
(  0.0%)	0/ 67 
(  0.0%)				
Missing at Timepoint of SVR	0/ 34 
(  0.0%)	0/ 33 
(  0.0%)	0/ 67 
(  0.0%)				
Completed all study therapy	0/ 34 
(  0.0%)	0/ 33 
(  0.0%)	0/ 67 
(  0.0%)				
	
SVR24							
Total with SVR24	33/ 34 
( 97.1%)	27/ 33 
( 81.8%)	60/ 67 
( 89.6%)				
Total without SVR24 for any reason	1/ 34 
(  2.9%)	6/ 33 
( 18.2%)	7/ 67 
( 10.4%)				
Detectable at EOT	0/ 34 
(  0.0%)	3/ 33 
(  9.1%)	3/ 67 
(  4.5%)				
Discontinued at least one study therapy (any other case)	0/ 34 
(  0.0%)	3/ 33 
(  9.1%)	3/ 67 
(  4.5%)				
Adverse event	0/ 34 
(  0.0%)	1/ 33 
(  3.0%)	1/ 67 
(  1.5%)				
Subject reached a virologic endpoint	0/ 34 
(  0.0%)	2/ 33 
(  6.1%)	2/ 67 
(  3.0%)				
Undetectable at EOTa	1/ 34 
(  2.9%)	3/ 33 
(  9.1%)	4/ 67 
(  6.0%)				
Completed all study therapy	1/ 34 
(  2.9%)	2/ 33 
(  6.1%)	3/ 67 
(  4.5%)				
Discontinued at least one study therapy (any other case)	0/ 34 
(  0.0%)	1/ 33 
(  3.0%)	1/ 67 
(  1.5%)				
Adverse event	0/ 34 
(  0.0%)	1/ 33 
(  3.0%)	1/ 67 
(  1.5%)				
Other	0/ 34 
(  0.0%)	0/ 33 
(  0.0%)	0/ 67 
(  0.0%)				
Subject reached a virologic endpoint	0/ 34 
(  0.0%)	0/ 33 
(  0.0%)	0/ 67 
(  0.0%)				
HCV RNA >= 25 at Timepoint of SVR	1/ 34 
(  2.9%)	3/ 33 
(  9.1%)	4/ 67 
(  6.0%)				
Completed all study therapy	1/ 34 
(  2.9%)	2/ 33 
(  6.1%)	3/ 67 
(  4.5%)				
Discontinued at least one study therapy (any other case)	0/ 34 
(  0.0%)	1/ 33 
(  3.0%)	1/ 67 
(  1.5%)				
Adverse event	0/ 34 
(  0.0%)	1/ 33 
(  3.0%)	1/ 67 
(  1.5%)				
Other	0/ 34 
(  0.0%)	0/ 33 
(  0.0%)	0/ 67 
(  0.0%)				
Subject reached a virologic endpoint	0/ 34 
(  0.0%)	0/ 33 
(  0.0%)	0/ 67 
(  0.0%)				
Missing at Timepoint of SVR	0/ 34 
(  0.0%)	0/ 33 
(  0.0%)	0/ 67 
(  0.0%)				
Completed all study therapy	0/ 34 
(  0.0%)	0/ 33 
(  0.0%)	0/ 67 
(  0.0%)				
	

a	Combination of the 2 categories: HCV RNA >= 25 IU/mL at Timepoint of SVR and Missing at Timepoint of SVR	
[TEFSVR03.rtf] [\STAT\Analyses\Programs\FinalAnalysis\Final1\2.TLF\2.Efficacy\EFF_FA.sas] 23OCT2015, 18:04	
