# Supplementary material for: Efficacy of a 12-Week Simeprevir Plus Peginterferon/Ribavirin (PR) Regimen in Treatment-Naïve Patients with Hepatitis C Virus (HCV) Genotype 4 (GT4) Infection and Mild-To-Moderate Fibrosis Displaying Early On-Treatment Virologic Response
Source: PLoS One. 2017 Jan 5;12(1):e0168713. doi: 10.1371/journal.pone.0168713 (PMC5215882; doi:10.1371/journal.pone.0168713)
Supplement: S1 Dataset — (ZIP) [file pone.0168713.s002.zip › TEFVF01.rtf]

TEFVF01:	On-Treatment and Post-Treatment Failure; Intent-to-treat (Study TMC435HPC3014)
Treatment Group = Simeprevir 12Wks 150 mg PR12/24	
	Genotype 4		
	12 Weeks 
Treatment	>12 Weeks 
Treatment	All Subjects	
Analysis set: intent-to-treat	34	33	67				
	
Failurea	1/ 34 
(  2.9%)	6/ 33 
( 18.2%)	7/ 67 
( 10.4%)				
On-treatment failureb	0/ 34 
(  0.0%)	3/ 33 
(  9.1%)	3/ 67 
(  4.5%)				
Discontinued PegIFN and RBV	0/ 34 
(  0.0%)	3/ 33 
(  9.1%)	3/ 67 
(  4.5%)				
Met a stopping rule at Week 4	0/ 34 
(  0.0%)	2/ 33 
(  6.1%)	2/ 67 
(  3.0%)				
Other	0/ 34 
(  0.0%)	1/ 33 
(  3.0%)	1/ 67 
(  1.5%)				
Viral breakthrough	0/ 34 
(  0.0%)	0/ 33 
(  0.0%)	0/ 67 
(  0.0%)				
Post-treatment failurec	1/ 34 
(  2.9%)	3/ 33 
(  9.1%)	4/ 67 
(  6.0%)				
Missing at timepoint of SVR12d	0/ 34 
(  0.0%)	0/ 33 
(  0.0%)	0/ 67 
(  0.0%)				
Viral relapse	1/ 34 
(  2.9%)	3/ 33 
(  9.1%)	4/ 67 
(  6.0%)				
Completed PegIFN and/or RBV	1/ 34 
(  2.9%)	2/ 33 
(  6.1%)	3/ 67 
(  4.5%)				
Discontinued PegIFN and RBV	0/ 34 
(  0.0%)	1/ 33 
(  3.0%)	1/ 67 
(  1.5%)				
	

Note: A subject can occur in only one category.
	Stopping rules: HCV RNA >= 25 IU/ml at the week 4 visit, HCV RNA >= 25 IU/mL or detectable at the week 12 visit or
	subject has a viral breakthrough. Subjects who met a stopping rule had to discontinue all treatment.
a	Subject did not achieve SVR12 or achieved SVR12 and had a relapse thereafter.
b	Confirmed detectable HCV RNA levels at actual EOT.
c	Failure but with undetectable (or unconfirmed detectable) HCV RNA levels at EOT
d	Subjects with on-treatment response, without viral relapse, but who fail solely because of missing data at the time point
	of SVR12 (and thereafter)
Subject with CRF ID TMC435HPC3014-0043 achieved HCV RNA <25 iu/ml detected at last study related visit (week 36) 
after previously having experienced a viral relapse. This subject will be further described in the CSR.	
[TEFVF01.rtf] [\STAT\Analyses\Programs\FinalAnalysis\Final1\2.TLF\2.Efficacy\EFF_FA.sas] 23OCT2015, 18:04	
