# Supplementary material for: Efficacy of a 12-Week Simeprevir Plus Peginterferon/Ribavirin (PR) Regimen in Treatment-Naïve Patients with Hepatitis C Virus (HCV) Genotype 4 (GT4) Infection and Mild-To-Moderate Fibrosis Displaying Early On-Treatment Virologic Response
Source: PLoS One. 2017 Jan 5;12(1):e0168713. doi: 10.1371/journal.pone.0168713 (PMC5215882; doi:10.1371/journal.pone.0168713)
Supplement: S1 Dataset — (ZIP) [file pone.0168713.s002.zip › TSFAE01TDG4.rtf]

TSFAE01TDG4:	Adverse Event Summary Table; Intent-to-treat (Study TMC435HPC3014) HCVGTGR1='Genotype 4'	
	Simeprevir
12 Wks
150 mg
PR 12/24 	
	 Genotype 4 	
	 12 Wks 	 >12 Wks 	
Analysis set: intent-to-treat	34	33	
			
Any AE	31 (91.2%)	29 (87.9%)	
p-value		0.659	
Any SAE		2 (6.1%)	
p-value		0.157	
At least possibly related to any Study Therapy	25 (73.5%)	27 (81.8%)	
p-value		0.689	
At least possibly related to SMV	10 (29.4%)	15 (45.5%)	
p-value		0.394	
At least possibly related to Ribavirin	17 (50.0%)	19 (57.6%)	
p-value		0.907	
At least possibly related to PegIFN	20 (58.8%)	26 (78.8%)	
p-value		0.511	
Any AE with fatal outcome			
Worst grade 3 or 4 AE	6 (17.6%)	14 (42.4%)	
p-value		0.027	
AE leading to permanent stop(a)		4 (12.1%)	
SMV(b)		3 (9.1%)	
SMV only			
SMV + PegIFN		1 (3.0%)	
SMV + RBV			
SMV, PegIFN and RBV		2 (6.1%)	
PegIFN or RBV		2 (6.1%)	
PegIFN only			
RBV only		1 (3.0%)	
PegIFN and RBV		1 (3.0%)	
	
[TSFAE01TDG4.RTF] [TMC435\HPC3014\DBR_FINAL_ANALYSIS\RE_FINAL_ANALYSIS\PDEV\TEMPFILE.SAS] 12OCT2016, 09:47	
