# Supplementary material for: Efficacy of a 12-Week Simeprevir Plus Peginterferon/Ribavirin (PR) Regimen in Treatment-Naïve Patients with Hepatitis C Virus (HCV) Genotype 4 (GT4) Infection and Mild-To-Moderate Fibrosis Displaying Early On-Treatment Virologic Response
Source: PLoS One. 2017 Jan 5;12(1):e0168713. doi: 10.1371/journal.pone.0168713 (PMC5215882; doi:10.1371/journal.pone.0168713)
Supplement: S1 Dataset — (ZIP) [file pone.0168713.s002.zip › tsfae01tdg4all.rtf]

TSFAE01TDG4ALL:	Adverse Event Summary Table; Intent-to-treat (Study TMC435HPC3014) HCVGTGR1='Genotype 4'	
	Simeprevir
12 Wks
150 mg
PR 12/24 	
	SMV + PR 	Ent Trt 	PR Only 	Follow-Up 	Overall 	
Analysis set: intent-to-treat	67	67	30	66	67	
						
Any AE	59 (88.1%)	60 (89.6%)	20 (66.7%)	12 (18.2%)	61 (91.0%)	
Any SAE	1 (1.5%)	2 (3.0%)	1 (3.3%)	2 (3.0%)	4 (6.0%)	
At least possibly related to any Study Therapy	52 (77.6%)	52 (77.6%)	13 (43.3%)		52 (77.6%)	
At least possibly related to SMV	25 (37.3%)	25 (37.3%)			25 (37.3%)	
At least possibly related to Ribavirin	35 (52.2%)	36 (53.7%)	7 (23.3%)		36 (53.7%)	
At least possibly related to PegIFN	45 (67.2%)	46 (68.7%)	11 (36.7%)		46 (68.7%)	
Worst grade 1 AE	25 (37.3%)	24 (35.8%)	13 (43.3%)	5 (7.6%)	24 (35.8%)	
Worst grade 2 AE	17 (25.4%)	16 (23.9%)	3 (10.0%)	3 (4.5%)	14 (20.9%)	
Worst grade 3 AE	15 (22.4%)	17 (25.4%)	3 (10.0%)	4 (6.1%)	20 (29.9%)	
Worst grade 4 AE	2 (3.0%)	3 (4.5%)	1 (3.3%)		3 (4.5%)	
Worst grade 1 or 2 AE	42 (62.7%)	40 (59.7%)	16 (53.3%)	8 (12.1%)	38 (56.7%)	
Worst grade 3 or 4 AE	17 (25.4%)	20 (29.9%)	4 (13.3%)	4 (6.1%)	23 (34.3%)	
At least possibly related to SMV	3 (4.5%)	3 (4.5%)			3 (4.5%)	
AE leading to permanent stop(a)	3 (4.5%)	4 (6.0%)	1 (3.3%)		4 (6.0%)	
SMV(b)	3 (4.5%)	3 (4.5%)			3 (4.5%)	
SMV + PegIFN	1 (1.5%)	1 (1.5%)			1 (1.5%)	
SMV, PegIFN and RBV	2 (3.0%)	2 (3.0%)			2 (3.0%)	
PegIFN or RBV	1 (1.5%)	2 (3.0%)	1 (3.3%)		2 (3.0%)	
RBV only	1 (1.5%)	1 (1.5%)			1 (1.5%)	
PegIFN and RBV		1 (1.5%)	1 (3.3%)		1 (1.5%)	
	
[TSFAE01TDG4ALL.RTF] [TMC435\HPC3014\DBR_FINAL_ANALYSIS\RE_FINAL_ANALYSIS\PROD\TSFAE01TD.SAS] 02NOV2015, 11:23	
