# Supplementary material for: Efficacy of a 12-Week Simeprevir Plus Peginterferon/Ribavirin (PR) Regimen in Treatment-Naïve Patients with Hepatitis C Virus (HCV) Genotype 4 (GT4) Infection and Mild-To-Moderate Fibrosis Displaying Early On-Treatment Virologic Response
Source: PLoS One. 2017 Jan 5;12(1):e0168713. doi: 10.1371/journal.pone.0168713 (PMC5215882; doi:10.1371/journal.pone.0168713)
Supplement: S1 Dataset — (ZIP) [file pone.0168713.s002.zip › tsfae01tdg4gt12.rtf]

TSFAE01TDG4GT12:	Adverse Event Summary Table; Intent-to-treat (Study TMC435HPC3014) HCVGTGR1='Genotype 4' and (planeot='24 Wks' or planeot='48 Wks')	
	Simeprevir
12 Wks
150 mg
PR 12/24 	
	SMV + PR 	Ent Trt 	PR Only 	Follow-Up 	Overall 	
Analysis set: intent-to-treat	33	33	27	32	33	
						
Any AE	29 (87.9%)	29 (87.9%)	18 (66.7%)	7 (21.9%)	30 (90.9%)	
Any SAE	1 (3.0%)	2 (6.1%)	1 (3.7%)	1 (3.1%)	3 (9.1%)	
At least possibly related to any Study Therapy	27 (81.8%)	27 (81.8%)	12 (44.4%)		27 (81.8%)	
At least possibly related to SMV	15 (45.5%)	15 (45.5%)			15 (45.5%)	
At least possibly related to Ribavirin	18 (54.5%)	19 (57.6%)	7 (25.9%)		19 (57.6%)	
At least possibly related to PegIFN	25 (75.8%)	26 (78.8%)	10 (37.0%)		26 (78.8%)	
Worst grade 1 AE	10 (30.3%)	8 (24.2%)	11 (40.7%)	4 (12.5%)	9 (27.3%)	
Worst grade 2 AE	8 (24.2%)	7 (21.2%)	3 (11.1%)	2 (6.3%)	7 (21.2%)	
Worst grade 3 AE	9 (27.3%)	11 (33.3%)	3 (11.1%)	1 (3.1%)	11 (33.3%)	
Worst grade 4 AE	2 (6.1%)	3 (9.1%)	1 (3.7%)		3 (9.1%)	
Worst grade 1 or 2 AE	18 (54.5%)	15 (45.5%)	14 (51.9%)	6 (18.8%)	16 (48.5%)	
Worst grade 3 or 4 AE	11 (33.3%)	14 (42.4%)	4 (14.8%)	1 (3.1%)	14 (42.4%)	
At least possibly related to SMV	3 (9.1%)	3 (9.1%)			3 (9.1%)	
AE leading to permanent stop(a)	3 (9.1%)	4 (12.1%)	1 (3.7%)		4 (12.1%)	
SMV(b)	3 (9.1%)	3 (9.1%)			3 (9.1%)	
SMV + PegIFN	1 (3.0%)	1 (3.0%)			1 (3.0%)	
SMV, PegIFN and RBV	2 (6.1%)	2 (6.1%)			2 (6.1%)	
PegIFN or RBV	1 (3.0%)	2 (6.1%)	1 (3.7%)		2 (6.1%)	
RBV only	1 (3.0%)	1 (3.0%)			1 (3.0%)	
PegIFN and RBV		1 (3.0%)	1 (3.7%)		1 (3.0%)	
	
[TSFAE01TDG4GT12.RTF] [TMC435\HPC3014\DBR_FINAL_ANALYSIS\RE_FINAL_ANALYSIS\PROD\TSFAE01TD.SAS] 02NOV2015, 11:23	
