# Supplementary material for: Efficacy of a 12-Week Simeprevir Plus Peginterferon/Ribavirin (PR) Regimen in Treatment-Naïve Patients with Hepatitis C Virus (HCV) Genotype 4 (GT4) Infection and Mild-To-Moderate Fibrosis Displaying Early On-Treatment Virologic Response
Source: PLoS One. 2017 Jan 5;12(1):e0168713. doi: 10.1371/journal.pone.0168713 (PMC5215882; doi:10.1371/journal.pone.0168713)
Supplement: S1 Dataset — (ZIP) [file pone.0168713.s002.zip › tsfae01tdg412.rtf]

TSFAE01TDG412:	Adverse Event Summary Table; Intent-to-treat (Study TMC435HPC3014) HCVGTGR1='Genotype 4' and planeot='12 Wks'	
	Simeprevir
12 Wks
150 mg
PR 12/24 	
	SMV + PR 	Ent Trt 	PR Only 	Follow-Up 	Overall 	
Analysis set: intent-to-treat	34	34	3	34	34	
						
Any AE	30 (88.2%)	31 (91.2%)	2 (66.7%)	5 (14.7%)	31 (91.2%)	
Any SAE				1 (2.9%)	1 (2.9%)	
At least possibly related to any Study Therapy	25 (73.5%)	25 (73.5%)	1 (33.3%)		25 (73.5%)	
At least possibly related to SMV	10 (29.4%)	10 (29.4%)			10 (29.4%)	
At least possibly related to Ribavirin	17 (50.0%)	17 (50.0%)			17 (50.0%)	
At least possibly related to PegIFN	20 (58.8%)	20 (58.8%)	1 (33.3%)		20 (58.8%)	
Worst grade 1 AE	15 (44.1%)	16 (47.1%)	2 (66.7%)	1 (2.9%)	15 (44.1%)	
Worst grade 2 AE	9 (26.5%)	9 (26.5%)		1 (2.9%)	7 (20.6%)	
Worst grade 3 AE	6 (17.6%)	6 (17.6%)		3 (8.8%)	9 (26.5%)	
Worst grade 1 or 2 AE	24 (70.6%)	25 (73.5%)	2 (66.7%)	2 (5.9%)	22 (64.7%)	
Worst grade 3 or 4 AE	6 (17.6%)	6 (17.6%)		3 (8.8%)	9 (26.5%)	
	
[TSFAE01TDG412.RTF] [TMC435\HPC3014\DBR_FINAL_ANALYSIS\RE_FINAL_ANALYSIS\PROD\TSFAE01TD.SAS] 02NOV2015, 11:23	
