# Supplementary material for: Efficacy of a 12-Week Simeprevir Plus Peginterferon/Ribavirin (PR) Regimen in Treatment-Naïve Patients with Hepatitis C Virus (HCV) Genotype 4 (GT4) Infection and Mild-To-Moderate Fibrosis Displaying Early On-Treatment Virologic Response
Source: PLoS One. 2017 Jan 5;12(1):e0168713. doi: 10.1371/journal.pone.0168713 (PMC5215882; doi:10.1371/journal.pone.0168713)
Supplement: S1 Dataset — (ZIP) [file pone.0168713.s002.zip › TSFAE02G4ET.rtf]

TSFAE02G4ET:	Number (pcnt) of Genotype 4 Subjects with Selected Adverse Events During Entire Treatment Phase, Intent-to-treat, Study TMC435HPC3014  	
	Simeprevir
12 Wks
150 mg
PR 12/24 	
	 Genotype 4 	
	 12 Wks 	 >12 Wks 	
Analysis set: Intent-to-treat	34	33	
Any AE	31 (91.2%)	29 (87.9%)	
p-value		0.659	
Pruritus	9 (29.0%)	7 (24.1%)	
p-value		0.748	
Asthenia	5 (16.1%)	9 (31.0%)	
p-value		0.308	
Fatigue	6 (19.4%)	8 (27.6%)	
p-value		0.553	
Decreased appetite	6 (19.4%)	8 (27.6%)	
p-value		0.553	
Headache	5 (16.1%)	8 (27.6%)	
p-value		0.388	
Influenza-like illness	3 (9.7%)	9 (31.0%)	
p-value		0.091	

P-value s alculated o est ccurence f Es etween reatment urations sing hi-square est	
[TSFAE02G4ET.RTF] [TMC435\HPC3014\DBR_FINAL_ANALYSIS\RE_FINAL_ANALYSIS\PDEV\TEMPFILE.SAS] 12OCT2016, 09:47	
