# Supplementary material for: Efficacy of a 12-Week Simeprevir Plus Peginterferon/Ribavirin (PR) Regimen in Treatment-Naïve Patients with Hepatitis C Virus (HCV) Genotype 4 (GT4) Infection and Mild-To-Moderate Fibrosis Displaying Early On-Treatment Virologic Response
Source: PLoS One. 2017 Jan 5;12(1):e0168713. doi: 10.1371/journal.pone.0168713 (PMC5215882; doi:10.1371/journal.pone.0168713)
Supplement: S1 Dataset — (ZIP) [file pone.0168713.s002.zip › tsfae02tdg4all.rtf]

TSFAE02TDG4ALL:	Number (pcnt) of Genotype 4 Subjects with Adverse Events, Intent-to-treat, Study TMC435HPC3014 All Subjects	
	Simeprevir
12 Wks
150 mg
PR 12/24 	
	SMV + PR 	Ent Trt 	PR Only 	Follow-Up 	Overall 	
Analysis set: Intent-to-treat	67	67	30	66	67	
Any AE	59 (88.1%)	60 (89.6%)	20 (66.7%)	12 (18.2%)	61 (91.0%)	
General disorders and administration site conditions	36 (53.7%)	37 (55.2%)	7 (23.3%)	2 (3.0%)	37 (55.2%)	
Asthenia	14 (20.9%)	14 (20.9%)	0	1 (1.5%)	14 (20.9%)	
Fatigue	14 (20.9%)	14 (20.9%)	0	0	14 (20.9%)	
Influenza like illness	11 (16.4%)	12 (17.9%)	2 (6.7%)	1 (1.5%)	12 (17.9%)	
Pyrexia	7 (10.4%)	8 (11.9%)	1 (3.3%)	0	8 (11.9%)	
Injection site erythema	1 (1.5%)	3 (4.5%)	2 (6.7%)	0	3 (4.5%)	
Chest pain	2 (3.0%)	2 (3.0%)	0	0	2 (3.0%)	
Irritability	1 (1.5%)	2 (3.0%)	1 (3.3%)	0	2 (3.0%)	
Application site alopecia	0	1 (1.5%)	1 (3.3%)	0	1 (1.5%)	
Discomfort	1 (1.5%)	1 (1.5%)	0	0	1 (1.5%)	
Injection site pruritus	0	1 (1.5%)	1 (3.3%)	0	1 (1.5%)	
Injection site rash	1 (1.5%)	1 (1.5%)	0	0	1 (1.5%)	
Mucosal dryness	1 (1.5%)	1 (1.5%)	0	0	1 (1.5%)	
Pain	0	1 (1.5%)	1 (3.3%)	0	1 (1.5%)	
Skin and subcutaneous tissue disorders	24 (35.8%)	31 (46.3%)	9 (30.0%)	0	31 (46.3%)	
Pruritus	15 (22.4%)	16 (23.9%)	3 (10.0%)	0	16 (23.9%)	
Rash	8 (11.9%)	11 (16.4%)	3 (10.0%)	0	11 (16.4%)	
Erythema	5 (7.5%)	6 (9.0%)	1 (3.3%)	0	6 (9.0%)	
Dry skin	3 (4.5%)	4 (6.0%)	1 (3.3%)	0	4 (6.0%)	
Eczema	0	2 (3.0%)	2 (6.7%)	0	2 (3.0%)	
Alopecia	1 (1.5%)	1 (1.5%)	0	0	1 (1.5%)	
Erythema nodosum	0	1 (1.5%)	1 (3.3%)	0	1 (1.5%)	
Hyperhidrosis	0	1 (1.5%)	1 (3.3%)	0	1 (1.5%)	
Onychoclasis	0	1 (1.5%)	1 (3.3%)	0	1 (1.5%)	
Psoriasis	0	1 (1.5%)	0	0	1 (1.5%)	
Gastrointestinal disorders	27 (40.3%)	27 (40.3%)	3 (10.0%)	1 (1.5%)	27 (40.3%)	
Diarrhoea	10 (14.9%)	10 (14.9%)	1 (3.3%)	0	10 (14.9%)	
Vomiting	6 (9.0%)	7 (10.4%)	1 (3.3%)	1 (1.5%)	7 (10.4%)	
Constipation	5 (7.5%)	5 (7.5%)	0	0	5 (7.5%)	
Abdominal pain upper	3 (4.5%)	4 (6.0%)	1 (3.3%)	0	4 (6.0%)	
Abdominal pain	3 (4.5%)	3 (4.5%)	0	0	3 (4.5%)	
Dry mouth	3 (4.5%)	3 (4.5%)	0	0	3 (4.5%)	
Dyspepsia	3 (4.5%)	3 (4.5%)	0	0	3 (4.5%)	
Nausea	3 (4.5%)	3 (4.5%)	0	0	3 (4.5%)	
Abdominal distension	1 (1.5%)	2 (3.0%)	1 (3.3%)	0	2 (3.0%)	
Haemorrhoids	2 (3.0%)	2 (3.0%)	0	0	2 (3.0%)	
Mouth ulceration	2 (3.0%)	2 (3.0%)	0	0	2 (3.0%)	
Abdominal discomfort	1 (1.5%)	1 (1.5%)	0	0	1 (1.5%)	
Abdominal pain lower	1 (1.5%)	1 (1.5%)	0	0	1 (1.5%)	
Abdominal tenderness	1 (1.5%)	1 (1.5%)	0	0	1 (1.5%)	
Anal inflammation	1 (1.5%)	1 (1.5%)	0	0	1 (1.5%)	
Anal pruritus	1 (1.5%)	1 (1.5%)	0	0	1 (1.5%)	
Colitis	1 (1.5%)	1 (1.5%)	0	0	1 (1.5%)	
Blood and lymphatic system disorders	18 (26.9%)	21 (31.3%)	3 (10.0%)	0	21 (31.3%)	
Neutropenia	13 (19.4%)	16 (23.9%)	3 (10.0%)	0	16 (23.9%)	
Anaemia	6 (9.0%)	7 (10.4%)	0	0	7 (10.4%)	
Thrombocytopenia	3 (4.5%)	3 (4.5%)	0	0	3 (4.5%)	
Leukopenia	2 (3.0%)	2 (3.0%)	0	0	2 (3.0%)	
Psychiatric disorders	17 (25.4%)	19 (28.4%)	2 (6.7%)	1 (1.5%)	20 (29.9%)	
Depression	6 (9.0%)	7 (10.4%)	1 (3.3%)	1 (1.5%)	8 (11.9%)	
Insomnia	6 (9.0%)	6 (9.0%)	0	0	6 (9.0%)	
Depressed mood	3 (4.5%)	3 (4.5%)	0	0	3 (4.5%)	
Sleep disorder	2 (3.0%)	3 (4.5%)	1 (3.3%)	0	3 (4.5%)	
Anxiety	2 (3.0%)	2 (3.0%)	0	0	2 (3.0%)	
Anxiety disorder	1 (1.5%)	1 (1.5%)	0	0	1 (1.5%)	
Libido decreased	1 (1.5%)	1 (1.5%)	0	0	1 (1.5%)	
Nervousness	1 (1.5%)	1 (1.5%)	0	0	1 (1.5%)	
Nervous system disorders	16 (23.9%)	18 (26.9%)	2 (6.7%)	1 (1.5%)	18 (26.9%)	
Headache	11 (16.4%)	13 (19.4%)	2 (6.7%)	0	13 (19.4%)	
Dizziness	4 (6.0%)	4 (6.0%)	0	0	4 (6.0%)	
Memory impairment	2 (3.0%)	2 (3.0%)	0	0	2 (3.0%)	
Paraesthesia	2 (3.0%)	2 (3.0%)	0	1 (1.5%)	3 (4.5%)	
Carpal tunnel syndrome	1 (1.5%)	1 (1.5%)	0	0	1 (1.5%)	
Disturbance in attention	1 (1.5%)	1 (1.5%)	0	0	1 (1.5%)	
Metabolism and nutrition disorders	14 (20.9%)	15 (22.4%)	1 (3.3%)	0	15 (22.4%)	
Decreased appetite	13 (19.4%)	14 (20.9%)	1 (3.3%)	0	14 (20.9%)	
Hyperinsulinaemia	1 (1.5%)	1 (1.5%)	0	0	1 (1.5%)	
Hypokalaemia	1 (1.5%)	1 (1.5%)	0	0	1 (1.5%)	
Investigations	11 (16.4%)	12 (17.9%)	5 (16.7%)	3 (4.5%)	15 (22.4%)	
Neutrophil count decreased	6 (9.0%)	7 (10.4%)	2 (6.7%)	0	7 (10.4%)	
Alanine aminotransferase increased	1 (1.5%)	3 (4.5%)	2 (6.7%)	0	3 (4.5%)	
Blood bilirubin increased	3 (4.5%)	3 (4.5%)	1 (3.3%)	0	3 (4.5%)	
Aspartate aminotransferase increased	0	2 (3.0%)	2 (6.7%)	0	2 (3.0%)	
Blood glucose increased	1 (1.5%)	2 (3.0%)	1 (3.3%)	1 (1.5%)	3 (4.5%)	
Haemoglobin decreased	2 (3.0%)	2 (3.0%)	0	0	2 (3.0%)	
Amylase increased	1 (1.5%)	1 (1.5%)	0	1 (1.5%)	2 (3.0%)	
Blood lactate dehydrogenase increased	0	1 (1.5%)	1 (3.3%)	0	1 (1.5%)	
Blood pressure increased	0	1 (1.5%)	0	0	1 (1.5%)	
Lipase increased	1 (1.5%)	1 (1.5%)	0	1 (1.5%)	2 (3.0%)	
Platelet count decreased	1 (1.5%)	1 (1.5%)	0	0	1 (1.5%)	
Weight decreased	1 (1.5%)	1 (1.5%)	0	0	1 (1.5%)	
Blood thyroid stimulating hormone increased	0	0	0	1 (1.5%)	1 (1.5%)	
Musculoskeletal and connective tissue disorders	10 (14.9%)	11 (16.4%)	2 (6.7%)	3 (4.5%)	13 (19.4%)	
Back pain	5 (7.5%)	6 (9.0%)	1 (3.3%)	1 (1.5%)	7 (10.4%)	
Arthralgia	1 (1.5%)	3 (4.5%)	1 (3.3%)	0	3 (4.5%)	
Myalgia	2 (3.0%)	2 (3.0%)	0	0	2 (3.0%)	
Muscle spasms	1 (1.5%)	1 (1.5%)	0	0	1 (1.5%)	
Musculoskeletal stiffness	1 (1.5%)	1 (1.5%)	0	0	1 (1.5%)	
Arthritis	0	0	0	1 (1.5%)	1 (1.5%)	
Tendonitis	0	0	0	1 (1.5%)	1 (1.5%)	
Respiratory, thoracic and mediastinal disorders	9 (13.4%)	10 (14.9%)	1 (3.3%)	2 (3.0%)	12 (17.9%)	
Dyspnoea	5 (7.5%)	6 (9.0%)	1 (3.3%)	0	6 (9.0%)	
Oropharyngeal pain	3 (4.5%)	3 (4.5%)	0	0	3 (4.5%)	
Dyspnoea exertional	1 (1.5%)	1 (1.5%)	0	0	1 (1.5%)	
Nasal congestion	1 (1.5%)	1 (1.5%)	0	0	1 (1.5%)	
Asthma	0	0	0	1 (1.5%)	1 (1.5%)	
Pulmonary embolism	0	0	0	1 (1.5%)	1 (1.5%)	
Infections and infestations	6 (9.0%)	8 (11.9%)	0	1 (1.5%)	9 (13.4%)	
Acute sinusitis	1 (1.5%)	1 (1.5%)	0	0	1 (1.5%)	
Bronchitis	0	1 (1.5%)	0	0	1 (1.5%)	
Fungal skin infection	1 (1.5%)	1 (1.5%)	0	0	1 (1.5%)	
Furuncle	1 (1.5%)	1 (1.5%)	0	0	1 (1.5%)	
Gastroenteritis	1 (1.5%)	1 (1.5%)	0	0	1 (1.5%)	
Gingival infection	1 (1.5%)	1 (1.5%)	0	0	1 (1.5%)	
Oral candidiasis	0	1 (1.5%)	0	0	1 (1.5%)	
Tooth abscess	1 (1.5%)	1 (1.5%)	0	0	1 (1.5%)	
Enterobiasis	0	0	0	1 (1.5%)	1 (1.5%)	
Ear and labyrinth disorders	7 (10.4%)	7 (10.4%)	2 (6.7%)	0	7 (10.4%)	
Tinnitus	3 (4.5%)	4 (6.0%)	2 (6.7%)	0	4 (6.0%)	
Vertigo	4 (6.0%)	4 (6.0%)	0	0	4 (6.0%)	
Cardiac disorders	3 (4.5%)	3 (4.5%)	0	1 (1.5%)	3 (4.5%)	
Palpitations	3 (4.5%)	3 (4.5%)	0	1 (1.5%)	3 (4.5%)	
Eye disorders	1 (1.5%)	3 (4.5%)	2 (6.7%)	0	3 (4.5%)	
Eye disorder	1 (1.5%)	1 (1.5%)	0	0	1 (1.5%)	
Eye pain	1 (1.5%)	1 (1.5%)	0	0	1 (1.5%)	
Vision blurred	0	1 (1.5%)	1 (3.3%)	0	1 (1.5%)	
Visual acuity reduced	0	1 (1.5%)	1 (3.3%)	0	1 (1.5%)	
Vascular disorders	1 (1.5%)	3 (4.5%)	3 (10.0%)	2 (3.0%)	5 (7.5%)	
Cryoglobulinaemia	1 (1.5%)	1 (1.5%)	0	0	1 (1.5%)	
Hypertension	0	1 (1.5%)	1 (3.3%)	1 (1.5%)	2 (3.0%)	
Pallor	0	1 (1.5%)	1 (3.3%)	0	1 (1.5%)	
Phlebitis	0	1 (1.5%)	1 (3.3%)	0	1 (1.5%)	
Deep vein thrombosis	0	0	0	1 (1.5%)	1 (1.5%)	
Hepatobiliary disorders	2 (3.0%)	2 (3.0%)	0	0	2 (3.0%)	
Hepatic pain	1 (1.5%)	1 (1.5%)	0	0	1 (1.5%)	
Hyperbilirubinaemia	1 (1.5%)	1 (1.5%)	0	0	1 (1.5%)	
Injury, poisoning and procedural complications	1 (1.5%)	2 (3.0%)	1 (3.3%)	1 (1.5%)	3 (4.5%)	
Ligament sprain	1 (1.5%)	1 (1.5%)	0	0	1 (1.5%)	
Scratch	0	1 (1.5%)	1 (3.3%)	0	1 (1.5%)	
Pelvic fracture	0	0	0	1 (1.5%)	1 (1.5%)	
Road traffic accident	0	0	0	1 (1.5%)	1 (1.5%)	
Immune system disorders	1 (1.5%)	1 (1.5%)	0	0	1 (1.5%)	
Seasonal allergy	1 (1.5%)	1 (1.5%)	0	0	1 (1.5%)	
Reproductive system and breast disorders	1 (1.5%)	1 (1.5%)	0	0	1 (1.5%)	
Dysmenorrhoea	1 (1.5%)	1 (1.5%)	0	0	1 (1.5%)	
	
[TSFAE02TDG4ALL.RTF] [TMC435\HPC3014\DBR_FINAL_ANALYSIS\RE_FINAL_ANALYSIS\PROD\TSFAE02TDG4ALL.SAS] 02NOV2015, 11:22	
