# Supplementary material for: Efficacy of a 12-Week Simeprevir Plus Peginterferon/Ribavirin (PR) Regimen in Treatment-Naïve Patients with Hepatitis C Virus (HCV) Genotype 4 (GT4) Infection and Mild-To-Moderate Fibrosis Displaying Early On-Treatment Virologic Response
Source: PLoS One. 2017 Jan 5;12(1):e0168713. doi: 10.1371/journal.pone.0168713 (PMC5215882; doi:10.1371/journal.pone.0168713)
Supplement: S1 Dataset — (ZIP) [file pone.0168713.s002.zip › tsfae02tdg4gt12.rtf]

TSFAE02TDG4GT12:	Number (pcnt) of Genotype 4 Subjects with Adverse Events, Intent-to-treat, Study TMC435HPC3014 Trt Dur >12 Wks	
	Simeprevir
12 Wks
150 mg
PR 12/24 	
	SMV + PR 	Ent Trt 	PR Only 	Follow-Up 	Overall 	
Analysis set: Intent-to-treat	33	33	27	32	33	
Any AE	29 (87.9%)	29 (87.9%)	18 (66.7%)	7 (21.9%)	30 (90.9%)	
General disorders and administration site conditions	20 (60.6%)	21 (63.6%)	6 (22.2%)	2 (6.3%)	21 (63.6%)	
Asthenia	9 (27.3%)	9 (27.3%)	0	1 (3.1%)	9 (27.3%)	
Influenza like illness	8 (24.2%)	9 (27.3%)	2 (7.4%)	1 (3.1%)	9 (27.3%)	
Fatigue	8 (24.2%)	8 (24.2%)	0	0	8 (24.2%)	
Pyrexia	5 (15.2%)	5 (15.2%)	0	0	5 (15.2%)	
Injection site erythema	1 (3.0%)	3 (9.1%)	2 (7.4%)	0	3 (9.1%)	
Application site alopecia	0	1 (3.0%)	1 (3.7%)	0	1 (3.0%)	
Injection site pruritus	0	1 (3.0%)	1 (3.7%)	0	1 (3.0%)	
Injection site rash	1 (3.0%)	1 (3.0%)	0	0	1 (3.0%)	
Irritability	0	1 (3.0%)	1 (3.7%)	0	1 (3.0%)	
Mucosal dryness	1 (3.0%)	1 (3.0%)	0	0	1 (3.0%)	
Pain	0	1 (3.0%)	1 (3.7%)	0	1 (3.0%)	
Skin and subcutaneous tissue disorders	11 (33.3%)	16 (48.5%)	8 (29.6%)	0	16 (48.5%)	
Pruritus	6 (18.2%)	7 (21.2%)	3 (11.1%)	0	7 (21.2%)	
Rash	4 (12.1%)	6 (18.2%)	3 (11.1%)	0	6 (18.2%)	
Erythema	2 (6.1%)	3 (9.1%)	1 (3.7%)	0	3 (9.1%)	
Dry skin	1 (3.0%)	2 (6.1%)	1 (3.7%)	0	2 (6.1%)	
Eczema	0	1 (3.0%)	1 (3.7%)	0	1 (3.0%)	
Erythema nodosum	0	1 (3.0%)	1 (3.7%)	0	1 (3.0%)	
Hyperhidrosis	0	1 (3.0%)	1 (3.7%)	0	1 (3.0%)	
Onychoclasis	0	1 (3.0%)	1 (3.7%)	0	1 (3.0%)	
Gastrointestinal disorders	14 (42.4%)	14 (42.4%)	3 (11.1%)	1 (3.1%)	14 (42.4%)	
Diarrhoea	6 (18.2%)	6 (18.2%)	1 (3.7%)	0	6 (18.2%)	
Vomiting	4 (12.1%)	5 (15.2%)	1 (3.7%)	1 (3.1%)	5 (15.2%)	
Constipation	3 (9.1%)	3 (9.1%)	0	0	3 (9.1%)	
Abdominal distension	1 (3.0%)	2 (6.1%)	1 (3.7%)	0	2 (6.1%)	
Abdominal pain	2 (6.1%)	2 (6.1%)	0	0	2 (6.1%)	
Abdominal pain upper	1 (3.0%)	2 (6.1%)	1 (3.7%)	0	2 (6.1%)	
Dyspepsia	2 (6.1%)	2 (6.1%)	0	0	2 (6.1%)	
Nausea	2 (6.1%)	2 (6.1%)	0	0	2 (6.1%)	
Abdominal discomfort	1 (3.0%)	1 (3.0%)	0	0	1 (3.0%)	
Abdominal pain lower	1 (3.0%)	1 (3.0%)	0	0	1 (3.0%)	
Abdominal tenderness	1 (3.0%)	1 (3.0%)	0	0	1 (3.0%)	
Anal inflammation	1 (3.0%)	1 (3.0%)	0	0	1 (3.0%)	
Colitis	1 (3.0%)	1 (3.0%)	0	0	1 (3.0%)	
Dry mouth	1 (3.0%)	1 (3.0%)	0	0	1 (3.0%)	
Haemorrhoids	1 (3.0%)	1 (3.0%)	0	0	1 (3.0%)	
Nervous system disorders	10 (30.3%)	12 (36.4%)	2 (7.4%)	1 (3.1%)	12 (36.4%)	
Headache	6 (18.2%)	8 (24.2%)	2 (7.4%)	0	8 (24.2%)	
Dizziness	3 (9.1%)	3 (9.1%)	0	0	3 (9.1%)	
Carpal tunnel syndrome	1 (3.0%)	1 (3.0%)	0	0	1 (3.0%)	
Memory impairment	1 (3.0%)	1 (3.0%)	0	0	1 (3.0%)	
Paraesthesia	1 (3.0%)	1 (3.0%)	0	1 (3.1%)	2 (6.1%)	
Psychiatric disorders	10 (30.3%)	12 (36.4%)	2 (7.4%)	1 (3.1%)	13 (39.4%)	
Depression	4 (12.1%)	5 (15.2%)	1 (3.7%)	1 (3.1%)	6 (18.2%)	
Insomnia	3 (9.1%)	3 (9.1%)	0	0	3 (9.1%)	
Sleep disorder	2 (6.1%)	3 (9.1%)	1 (3.7%)	0	3 (9.1%)	
Depressed mood	2 (6.1%)	2 (6.1%)	0	0	2 (6.1%)	
Anxiety disorder	1 (3.0%)	1 (3.0%)	0	0	1 (3.0%)	
Libido decreased	1 (3.0%)	1 (3.0%)	0	0	1 (3.0%)	
Blood and lymphatic system disorders	8 (24.2%)	10 (30.3%)	3 (11.1%)	0	10 (30.3%)	
Neutropenia	4 (12.1%)	7 (21.2%)	3 (11.1%)	0	7 (21.2%)	
Anaemia	4 (12.1%)	4 (12.1%)	0	0	4 (12.1%)	
Thrombocytopenia	2 (6.1%)	2 (6.1%)	0	0	2 (6.1%)	
Leukopenia	1 (3.0%)	1 (3.0%)	0	0	1 (3.0%)	
Investigations	9 (27.3%)	10 (30.3%)	5 (18.5%)	0	10 (30.3%)	
Neutrophil count decreased	4 (12.1%)	5 (15.2%)	2 (7.4%)	0	5 (15.2%)	
Alanine aminotransferase increased	1 (3.0%)	3 (9.1%)	2 (7.4%)	0	3 (9.1%)	
Blood bilirubin increased	3 (9.1%)	3 (9.1%)	1 (3.7%)	0	3 (9.1%)	
Aspartate aminotransferase increased	0	2 (6.1%)	2 (7.4%)	0	2 (6.1%)	
Blood glucose increased	1 (3.0%)	2 (6.1%)	1 (3.7%)	0	2 (6.1%)	
Amylase increased	1 (3.0%)	1 (3.0%)	0	0	1 (3.0%)	
Blood lactate dehydrogenase increased	0	1 (3.0%)	1 (3.7%)	0	1 (3.0%)	
Blood pressure increased	0	1 (3.0%)	0	0	1 (3.0%)	
Haemoglobin decreased	1 (3.0%)	1 (3.0%)	0	0	1 (3.0%)	
Lipase increased	1 (3.0%)	1 (3.0%)	0	0	1 (3.0%)	
Platelet count decreased	1 (3.0%)	1 (3.0%)	0	0	1 (3.0%)	
Weight decreased	1 (3.0%)	1 (3.0%)	0	0	1 (3.0%)	
Metabolism and nutrition disorders	8 (24.2%)	9 (27.3%)	1 (3.7%)	0	9 (27.3%)	
Decreased appetite	7 (21.2%)	8 (24.2%)	1 (3.7%)	0	8 (24.2%)	
Hyperinsulinaemia	1 (3.0%)	1 (3.0%)	0	0	1 (3.0%)	
Hypokalaemia	1 (3.0%)	1 (3.0%)	0	0	1 (3.0%)	
Respiratory, thoracic and mediastinal disorders	7 (21.2%)	8 (24.2%)	1 (3.7%)	1 (3.1%)	9 (27.3%)	
Dyspnoea	5 (15.2%)	6 (18.2%)	1 (3.7%)	0	6 (18.2%)	
Dyspnoea exertional	1 (3.0%)	1 (3.0%)	0	0	1 (3.0%)	
Nasal congestion	1 (3.0%)	1 (3.0%)	0	0	1 (3.0%)	
Oropharyngeal pain	1 (3.0%)	1 (3.0%)	0	0	1 (3.0%)	
Asthma	0	0	0	1 (3.1%)	1 (3.0%)	
Musculoskeletal and connective tissue disorders	6 (18.2%)	7 (21.2%)	2 (7.4%)	3 (9.4%)	9 (27.3%)	
Back pain	2 (6.1%)	3 (9.1%)	1 (3.7%)	1 (3.1%)	4 (12.1%)	
Arthralgia	1 (3.0%)	2 (6.1%)	1 (3.7%)	0	2 (6.1%)	
Muscle spasms	1 (3.0%)	1 (3.0%)	0	0	1 (3.0%)	
Musculoskeletal stiffness	1 (3.0%)	1 (3.0%)	0	0	1 (3.0%)	
Myalgia	1 (3.0%)	1 (3.0%)	0	0	1 (3.0%)	
Arthritis	0	0	0	1 (3.1%)	1 (3.0%)	
Tendonitis	0	0	0	1 (3.1%)	1 (3.0%)	
Ear and labyrinth disorders	4 (12.1%)	4 (12.1%)	1 (3.7%)	0	4 (12.1%)	
Vertigo	3 (9.1%)	3 (9.1%)	0	0	3 (9.1%)	
Tinnitus	1 (3.0%)	2 (6.1%)	1 (3.7%)	0	2 (6.1%)	
Infections and infestations	3 (9.1%)	4 (12.1%)	0	1 (3.1%)	5 (15.2%)	
Acute sinusitis	1 (3.0%)	1 (3.0%)	0	0	1 (3.0%)	
Bronchitis	0	1 (3.0%)	0	0	1 (3.0%)	
Gastroenteritis	1 (3.0%)	1 (3.0%)	0	0	1 (3.0%)	
Tooth abscess	1 (3.0%)	1 (3.0%)	0	0	1 (3.0%)	
Enterobiasis	0	0	0	1 (3.1%)	1 (3.0%)	
Vascular disorders	1 (3.0%)	3 (9.1%)	3 (11.1%)	0	3 (9.1%)	
Cryoglobulinaemia	1 (3.0%)	1 (3.0%)	0	0	1 (3.0%)	
Hypertension	0	1 (3.0%)	1 (3.7%)	0	1 (3.0%)	
Pallor	0	1 (3.0%)	1 (3.7%)	0	1 (3.0%)	
Phlebitis	0	1 (3.0%)	1 (3.7%)	0	1 (3.0%)	
Eye disorders	0	2 (6.1%)	2 (7.4%)	0	2 (6.1%)	
Vision blurred	0	1 (3.0%)	1 (3.7%)	0	1 (3.0%)	
Visual acuity reduced	0	1 (3.0%)	1 (3.7%)	0	1 (3.0%)	
Cardiac disorders	1 (3.0%)	1 (3.0%)	0	1 (3.1%)	1 (3.0%)	
Palpitations	1 (3.0%)	1 (3.0%)	0	1 (3.1%)	1 (3.0%)	
Injury, poisoning and procedural complications	0	1 (3.0%)	1 (3.7%)	1 (3.1%)	2 (6.1%)	
Scratch	0	1 (3.0%)	1 (3.7%)	0	1 (3.0%)	
Pelvic fracture	0	0	0	1 (3.1%)	1 (3.0%)	
Road traffic accident	0	0	0	1 (3.1%)	1 (3.0%)	
	
[TSFAE02TDG4GT12.RTF] [TMC435\HPC3014\DBR_FINAL_ANALYSIS\RE_FINAL_ANALYSIS\PROD\TSFAE02TDG4.SAS] 02NOV2015, 11:22	
