# Supplementary material for: Efficacy of a 12-Week Simeprevir Plus Peginterferon/Ribavirin (PR) Regimen in Treatment-Naïve Patients with Hepatitis C Virus (HCV) Genotype 4 (GT4) Infection and Mild-To-Moderate Fibrosis Displaying Early On-Treatment Virologic Response
Source: PLoS One. 2017 Jan 5;12(1):e0168713. doi: 10.1371/journal.pone.0168713 (PMC5215882; doi:10.1371/journal.pone.0168713)
Supplement: S1 Dataset — (ZIP) [file pone.0168713.s002.zip › tsfae05tdg4all.rtf]

TSFAE05TDG4ALL:	TSFAE05TDG4ALL: Number (pcnt) of Genotype 4 Subjects with Adverse Events by Worst WHO Toxicity Grade, Intent-to-treat, Study TMC435HPC3014 All Subjects	
	Simeprevir
12 Wks
150 mg
PR 12/24 	
	SMV + PR 	Ent Trt 	PR Only 	Follow-Up 	Overall 	
Analysis set: Intent-to-treat	67	67	30	66	67	
Any Grade 1 AE	25 (37.3%)	24 (35.8%)	13 (43.3%)	5 (7.6%)	24 (35.8%)	
General disorders and administration site conditions	27 (40.3%)	28 (41.8%)	6 (20.0%)	1 (1.5%)	27 (40.3%)	
Fatigue	11 (16.4%)	11 (16.4%)	0	0	11 (16.4%)	
Asthenia	10 (14.9%)	10 (14.9%)	0	1 (1.5%)	10 (14.9%)	
Influenza like illness	9 (13.4%)	9 (13.4%)	1 (3.3%)	0	8 (11.9%)	
Pyrexia	6 (9.0%)	7 (10.4%)	1 (3.3%)	0	7 (10.4%)	
Injection site erythema	1 (1.5%)	3 (4.5%)	2 (6.7%)	0	3 (4.5%)	
Irritability	1 (1.5%)	2 (3.0%)	1 (3.3%)	0	2 (3.0%)	
Application site alopecia	0	1 (1.5%)	1 (3.3%)	0	1 (1.5%)	
Chest pain	1 (1.5%)	1 (1.5%)	0	0	1 (1.5%)	
Discomfort	1 (1.5%)	1 (1.5%)	0	0	1 (1.5%)	
Injection site pruritus	0	1 (1.5%)	1 (3.3%)	0	1 (1.5%)	
Injection site rash	1 (1.5%)	1 (1.5%)	0	0	1 (1.5%)	
Mucosal dryness	1 (1.5%)	1 (1.5%)	0	0	1 (1.5%)	
Pain	0	1 (1.5%)	1 (3.3%)	0	1 (1.5%)	
Gastrointestinal disorders	22 (32.8%)	22 (32.8%)	3 (10.0%)	0	22 (32.8%)	
Diarrhoea	10 (14.9%)	10 (14.9%)	1 (3.3%)	0	10 (14.9%)	
Vomiting	6 (9.0%)	7 (10.4%)	1 (3.3%)	0	6 (9.0%)	
Abdominal pain upper	3 (4.5%)	4 (6.0%)	1 (3.3%)	0	4 (6.0%)	
Constipation	3 (4.5%)	3 (4.5%)	0	0	3 (4.5%)	
Dry mouth	3 (4.5%)	3 (4.5%)	0	0	3 (4.5%)	
Dyspepsia	3 (4.5%)	3 (4.5%)	0	0	3 (4.5%)	
Nausea	3 (4.5%)	3 (4.5%)	0	0	3 (4.5%)	
Abdominal distension	1 (1.5%)	2 (3.0%)	1 (3.3%)	0	2 (3.0%)	
Abdominal pain	2 (3.0%)	2 (3.0%)	0	0	2 (3.0%)	
Haemorrhoids	2 (3.0%)	2 (3.0%)	0	0	2 (3.0%)	
Abdominal discomfort	1 (1.5%)	1 (1.5%)	0	0	1 (1.5%)	
Abdominal tenderness	1 (1.5%)	1 (1.5%)	0	0	1 (1.5%)	
Anal inflammation	1 (1.5%)	1 (1.5%)	0	0	1 (1.5%)	
Anal pruritus	1 (1.5%)	1 (1.5%)	0	0	1 (1.5%)	
Mouth ulceration	1 (1.5%)	1 (1.5%)	0	0	1 (1.5%)	
Skin and subcutaneous tissue disorders	17 (25.4%)	22 (32.8%)	7 (23.3%)	0	22 (32.8%)	
Pruritus	11 (16.4%)	12 (17.9%)	3 (10.0%)	0	12 (17.9%)	
Rash	6 (9.0%)	9 (13.4%)	3 (10.0%)	0	9 (13.4%)	
Erythema	4 (6.0%)	5 (7.5%)	1 (3.3%)	0	5 (7.5%)	
Dry skin	2 (3.0%)	3 (4.5%)	1 (3.3%)	0	3 (4.5%)	
Alopecia	1 (1.5%)	1 (1.5%)	0	0	1 (1.5%)	
Eczema	0	1 (1.5%)	1 (3.3%)	0	1 (1.5%)	
Erythema nodosum	0	1 (1.5%)	1 (3.3%)	0	1 (1.5%)	
Hyperhidrosis	0	1 (1.5%)	1 (3.3%)	0	1 (1.5%)	
Psoriasis	0	1 (1.5%)	0	0	1 (1.5%)	
Nervous system disorders	13 (19.4%)	15 (22.4%)	2 (6.7%)	1 (1.5%)	15 (22.4%)	
Headache	9 (13.4%)	11 (16.4%)	2 (6.7%)	0	11 (16.4%)	
Dizziness	3 (4.5%)	3 (4.5%)	0	0	3 (4.5%)	
Paraesthesia	2 (3.0%)	2 (3.0%)	0	1 (1.5%)	3 (4.5%)	
Memory impairment	2 (3.0%)	2 (3.0%)	0	0	2 (3.0%)	
Carpal tunnel syndrome	1 (1.5%)	1 (1.5%)	0	0	1 (1.5%)	
Disturbance in attention	1 (1.5%)	1 (1.5%)	0	0	1 (1.5%)	
Metabolism and nutrition disorders	14 (20.9%)	14 (20.9%)	0	0	14 (20.9%)	
Decreased appetite	13 (19.4%)	13 (19.4%)	0	0	13 (19.4%)	
Hyperinsulinaemia	1 (1.5%)	1 (1.5%)	0	0	1 (1.5%)	
Hypokalaemia	1 (1.5%)	1 (1.5%)	0	0	1 (1.5%)	
Psychiatric disorders	12 (17.9%)	13 (19.4%)	1 (3.3%)	1 (1.5%)	14 (20.9%)	
Depression	4 (6.0%)	5 (7.5%)	1 (3.3%)	1 (1.5%)	6 (9.0%)	
Insomnia	5 (7.5%)	5 (7.5%)	0	0	5 (7.5%)	
Anxiety	2 (3.0%)	2 (3.0%)	0	0	2 (3.0%)	
Anxiety disorder	1 (1.5%)	1 (1.5%)	0	0	1 (1.5%)	
Depressed mood	1 (1.5%)	1 (1.5%)	0	0	1 (1.5%)	
Libido decreased	1 (1.5%)	1 (1.5%)	0	0	1 (1.5%)	
Nervousness	1 (1.5%)	1 (1.5%)	0	0	1 (1.5%)	
Sleep disorder	1 (1.5%)	1 (1.5%)	0	0	1 (1.5%)	
Musculoskeletal and connective tissue disorders	10 (14.9%)	11 (16.4%)	2 (6.7%)	1 (1.5%)	11 (16.4%)	
Back pain	5 (7.5%)	6 (9.0%)	1 (3.3%)	0	6 (9.0%)	
Arthralgia	1 (1.5%)	3 (4.5%)	1 (3.3%)	0	3 (4.5%)	
Myalgia	2 (3.0%)	2 (3.0%)	0	0	2 (3.0%)	
Arthritis	0	0	0	1 (1.5%)	1 (1.5%)	
Muscle spasms	1 (1.5%)	1 (1.5%)	0	0	1 (1.5%)	
Musculoskeletal stiffness	1 (1.5%)	1 (1.5%)	0	0	1 (1.5%)	
Respiratory, thoracic and mediastinal disorders	7 (10.4%)	8 (11.9%)	1 (3.3%)	0	8 (11.9%)	
Dyspnoea	4 (6.0%)	5 (7.5%)	1 (3.3%)	0	5 (7.5%)	
Oropharyngeal pain	2 (3.0%)	2 (3.0%)	0	0	2 (3.0%)	
Dyspnoea exertional	1 (1.5%)	1 (1.5%)	0	0	1 (1.5%)	
Nasal congestion	1 (1.5%)	1 (1.5%)	0	0	1 (1.5%)	
Blood and lymphatic system disorders	5 (7.5%)	7 (10.4%)	1 (3.3%)	0	7 (10.4%)	
Neutropenia	4 (6.0%)	5 (7.5%)	1 (3.3%)	0	5 (7.5%)	
Anaemia	3 (4.5%)	4 (6.0%)	0	0	4 (6.0%)	
Leukopenia	2 (3.0%)	2 (3.0%)	0	0	2 (3.0%)	
Thrombocytopenia	2 (3.0%)	2 (3.0%)	0	0	2 (3.0%)	
Ear and labyrinth disorders	7 (10.4%)	7 (10.4%)	2 (6.7%)	0	7 (10.4%)	
Tinnitus	3 (4.5%)	4 (6.0%)	2 (6.7%)	0	4 (6.0%)	
Vertigo	4 (6.0%)	4 (6.0%)	0	0	4 (6.0%)	
Infections and infestations	2 (3.0%)	4 (6.0%)	0	1 (1.5%)	5 (7.5%)	
Bronchitis	0	1 (1.5%)	0	0	1 (1.5%)	
Enterobiasis	0	0	0	1 (1.5%)	1 (1.5%)	
Furuncle	1 (1.5%)	1 (1.5%)	0	0	1 (1.5%)	
Oral candidiasis	0	1 (1.5%)	0	0	1 (1.5%)	
Tooth abscess	1 (1.5%)	1 (1.5%)	0	0	1 (1.5%)	
Cardiac disorders	3 (4.5%)	3 (4.5%)	0	1 (1.5%)	3 (4.5%)	
Palpitations	3 (4.5%)	3 (4.5%)	0	1 (1.5%)	3 (4.5%)	
Eye disorders	1 (1.5%)	3 (4.5%)	2 (6.7%)	0	3 (4.5%)	
Eye disorder	1 (1.5%)	1 (1.5%)	0	0	1 (1.5%)	
Eye pain	1 (1.5%)	1 (1.5%)	0	0	1 (1.5%)	
Vision blurred	0	1 (1.5%)	1 (3.3%)	0	1 (1.5%)	
Visual acuity reduced	0	1 (1.5%)	1 (3.3%)	0	1 (1.5%)	
Investigations	2 (3.0%)	2 (3.0%)	2 (6.7%)	1 (1.5%)	3 (4.5%)	
Blood glucose increased	1 (1.5%)	2 (3.0%)	1 (3.3%)	0	2 (3.0%)	
Alanine aminotransferase increased	0	1 (1.5%)	1 (3.3%)	0	1 (1.5%)	
Blood lactate dehydrogenase increased	0	1 (1.5%)	1 (3.3%)	0	1 (1.5%)	
Blood pressure increased	0	1 (1.5%)	0	0	1 (1.5%)	
Blood thyroid stimulating hormone increased	0	0	0	1 (1.5%)	1 (1.5%)	
Haemoglobin decreased	1 (1.5%)	1 (1.5%)	0	0	1 (1.5%)	
Lipase increased	1 (1.5%)	1 (1.5%)	0	0	1 (1.5%)	
Neutrophil count decreased	1 (1.5%)	1 (1.5%)	0	0	1 (1.5%)	
Blood bilirubin increased	0	0	1 (3.3%)	0	0	
Injury, poisoning and procedural complications	1 (1.5%)	2 (3.0%)	1 (3.3%)	0	2 (3.0%)	
Ligament sprain	1 (1.5%)	1 (1.5%)	0	0	1 (1.5%)	
Scratch	0	1 (1.5%)	1 (3.3%)	0	1 (1.5%)	
Hepatobiliary disorders	1 (1.5%)	1 (1.5%)	0	0	1 (1.5%)	
Hepatic pain	1 (1.5%)	1 (1.5%)	0	0	1 (1.5%)	
Immune system disorders	1 (1.5%)	1 (1.5%)	0	0	1 (1.5%)	
Seasonal allergy	1 (1.5%)	1 (1.5%)	0	0	1 (1.5%)	
Vascular disorders	1 (1.5%)	1 (1.5%)	1 (3.3%)	0	1 (1.5%)	
Cryoglobulinaemia	1 (1.5%)	1 (1.5%)	0	0	1 (1.5%)	
Hypertension	0	1 (1.5%)	1 (3.3%)	0	1 (1.5%)	
Any Grade 2 AE	17 (25.4%)	16 (23.9%)	3 (10.0%)	3 (4.5%)	14 (20.9%)	
Skin and subcutaneous tissue disorders	7 (10.4%)	9 (13.4%)	2 (6.7%)	0	9 (13.4%)	
Pruritus	4 (6.0%)	4 (6.0%)	0	0	4 (6.0%)	
Rash	2 (3.0%)	2 (3.0%)	0	0	2 (3.0%)	
Dry skin	1 (1.5%)	1 (1.5%)	0	0	1 (1.5%)	
Eczema	0	1 (1.5%)	1 (3.3%)	0	1 (1.5%)	
Erythema	1 (1.5%)	1 (1.5%)	0	0	1 (1.5%)	
Onychoclasis	0	1 (1.5%)	1 (3.3%)	0	1 (1.5%)	
General disorders and administration site conditions	7 (10.4%)	7 (10.4%)	1 (3.3%)	1 (1.5%)	8 (11.9%)	
Influenza like illness	2 (3.0%)	3 (4.5%)	1 (3.3%)	1 (1.5%)	4 (6.0%)	
Fatigue	3 (4.5%)	3 (4.5%)	0	0	3 (4.5%)	
Asthenia	2 (3.0%)	2 (3.0%)	0	0	2 (3.0%)	
Chest pain	1 (1.5%)	1 (1.5%)	0	0	1 (1.5%)	
Pyrexia	1 (1.5%)	1 (1.5%)	0	0	1 (1.5%)	
Blood and lymphatic system disorders	6 (9.0%)	6 (9.0%)	1 (3.3%)	0	6 (9.0%)	
Anaemia	3 (4.5%)	3 (4.5%)	0	0	3 (4.5%)	
Neutropenia	2 (3.0%)	3 (4.5%)	1 (3.3%)	0	3 (4.5%)	
Thrombocytopenia	1 (1.5%)	1 (1.5%)	0	0	1 (1.5%)	
Gastrointestinal disorders	5 (7.5%)	5 (7.5%)	0	1 (1.5%)	5 (7.5%)	
Constipation	2 (3.0%)	2 (3.0%)	0	0	2 (3.0%)	
Abdominal pain	1 (1.5%)	1 (1.5%)	0	0	1 (1.5%)	
Abdominal pain lower	1 (1.5%)	1 (1.5%)	0	0	1 (1.5%)	
Colitis	1 (1.5%)	1 (1.5%)	0	0	1 (1.5%)	
Mouth ulceration	1 (1.5%)	1 (1.5%)	0	0	1 (1.5%)	
Vomiting	0	0	0	1 (1.5%)	1 (1.5%)	
Psychiatric disorders	4 (6.0%)	5 (7.5%)	1 (3.3%)	0	5 (7.5%)	
Depressed mood	2 (3.0%)	2 (3.0%)	0	0	2 (3.0%)	
Sleep disorder	1 (1.5%)	2 (3.0%)	1 (3.3%)	0	2 (3.0%)	
Depression	1 (1.5%)	1 (1.5%)	0	0	1 (1.5%)	
Insomnia	1 (1.5%)	1 (1.5%)	0	0	1 (1.5%)	
Infections and infestations	4 (6.0%)	4 (6.0%)	0	0	4 (6.0%)	
Acute sinusitis	1 (1.5%)	1 (1.5%)	0	0	1 (1.5%)	
Fungal skin infection	1 (1.5%)	1 (1.5%)	0	0	1 (1.5%)	
Gastroenteritis	1 (1.5%)	1 (1.5%)	0	0	1 (1.5%)	
Gingival infection	1 (1.5%)	1 (1.5%)	0	0	1 (1.5%)	
Respiratory, thoracic and mediastinal disorders	2 (3.0%)	2 (3.0%)	0	1 (1.5%)	3 (4.5%)	
Asthma	0	0	0	1 (1.5%)	1 (1.5%)	
Dyspnoea	1 (1.5%)	1 (1.5%)	0	0	1 (1.5%)	
Oropharyngeal pain	1 (1.5%)	1 (1.5%)	0	0	1 (1.5%)	
Vascular disorders	0	2 (3.0%)	2 (6.7%)	1 (1.5%)	3 (4.5%)	
Hypertension	0	0	0	1 (1.5%)	1 (1.5%)	
Pallor	0	1 (1.5%)	1 (3.3%)	0	1 (1.5%)	
Phlebitis	0	1 (1.5%)	1 (3.3%)	0	1 (1.5%)	
Musculoskeletal and connective tissue disorders	0	0	0	2 (3.0%)	2 (3.0%)	
Back pain	0	0	0	1 (1.5%)	1 (1.5%)	
Tendonitis	0	0	0	1 (1.5%)	1 (1.5%)	
Nervous system disorders	2 (3.0%)	2 (3.0%)	0	0	2 (3.0%)	
Dizziness	1 (1.5%)	1 (1.5%)	0	0	1 (1.5%)	
Headache	1 (1.5%)	1 (1.5%)	0	0	1 (1.5%)	
Hepatobiliary disorders	1 (1.5%)	1 (1.5%)	0	0	1 (1.5%)	
Hyperbilirubinaemia	1 (1.5%)	1 (1.5%)	0	0	1 (1.5%)	
Investigations	2 (3.0%)	1 (1.5%)	0	0	1 (1.5%)	
Aspartate aminotransferase increased	0	2 (3.0%)	2 (6.7%)	0	2 (3.0%)	
Blood bilirubin increased	2 (3.0%)	2 (3.0%)	0	0	2 (3.0%)	
Haemoglobin decreased	1 (1.5%)	1 (1.5%)	0	0	1 (1.5%)	
Platelet count decreased	1 (1.5%)	1 (1.5%)	0	0	1 (1.5%)	
Weight decreased	1 (1.5%)	1 (1.5%)	0	0	1 (1.5%)	
Metabolism and nutrition disorders	0	1 (1.5%)	1 (3.3%)	0	1 (1.5%)	
Decreased appetite	0	1 (1.5%)	1 (3.3%)	0	1 (1.5%)	
Reproductive system and breast disorders	1 (1.5%)	1 (1.5%)	0	0	1 (1.5%)	
Dysmenorrhoea	1 (1.5%)	1 (1.5%)	0	0	1 (1.5%)	
Any Grade 3 AE	15 (22.4%)	17 (25.4%)	3 (10.0%)	4 (6.1%)	20 (29.9%)	
Blood and lymphatic system disorders	7 (10.4%)	8 (11.9%)	1 (3.3%)	0	8 (11.9%)	
Neutropenia	7 (10.4%)	8 (11.9%)	1 (3.3%)	0	8 (11.9%)	
Investigations	5 (7.5%)	6 (9.0%)	2 (6.7%)	2 (3.0%)	8 (11.9%)	
Neutrophil count decreased	3 (4.5%)	3 (4.5%)	1 (3.3%)	0	3 (4.5%)	
Alanine aminotransferase increased	1 (1.5%)	2 (3.0%)	1 (3.3%)	0	2 (3.0%)	
Amylase increased	1 (1.5%)	1 (1.5%)	0	1 (1.5%)	2 (3.0%)	
Blood bilirubin increased	1 (1.5%)	1 (1.5%)	0	0	1 (1.5%)	
Blood glucose increased	0	0	0	1 (1.5%)	1 (1.5%)	
Lipase increased	0	0	0	1 (1.5%)	1 (1.5%)	
General disorders and administration site conditions	2 (3.0%)	2 (3.0%)	0	0	2 (3.0%)	
Asthenia	2 (3.0%)	2 (3.0%)	0	0	2 (3.0%)	
Injury, poisoning and procedural complications	0	0	0	1 (1.5%)	1 (1.5%)	
Pelvic fracture	0	0	0	1 (1.5%)	1 (1.5%)	
Road traffic accident	0	0	0	1 (1.5%)	1 (1.5%)	
Nervous system disorders	1 (1.5%)	1 (1.5%)	0	0	1 (1.5%)	
Headache	1 (1.5%)	1 (1.5%)	0	0	1 (1.5%)	
Psychiatric disorders	1 (1.5%)	1 (1.5%)	0	0	1 (1.5%)	
Depression	1 (1.5%)	1 (1.5%)	0	0	1 (1.5%)	
Respiratory, thoracic and mediastinal disorders	0	0	0	1 (1.5%)	1 (1.5%)	
Pulmonary embolism	0	0	0	1 (1.5%)	1 (1.5%)	
Vascular disorders	0	0	0	1 (1.5%)	1 (1.5%)	
Deep vein thrombosis	0	0	0	1 (1.5%)	1 (1.5%)	
Any Grade 4 AE	2 (3.0%)	3 (4.5%)	1 (3.3%)	0	3 (4.5%)	
Investigations	2 (3.0%)	3 (4.5%)	1 (3.3%)	0	3 (4.5%)	
Neutrophil count decreased	2 (3.0%)	3 (4.5%)	1 (3.3%)	0	3 (4.5%)	
Any Grade 3-4 AE	17 (25.4%)	20 (29.9%)	4 (13.3%)	4 (6.1%)	23 (34.3%)	
Investigations	7 (10.4%)	9 (13.4%)	3 (10.0%)	2 (3.0%)	11 (16.4%)	
Neutrophil count decreased	5 (7.5%)	6 (9.0%)	2 (6.7%)	0	6 (9.0%)	
Alanine aminotransferase increased	1 (1.5%)	2 (3.0%)	1 (3.3%)	0	2 (3.0%)	
Amylase increased	1 (1.5%)	1 (1.5%)	0	1 (1.5%)	2 (3.0%)	
Blood bilirubin increased	1 (1.5%)	1 (1.5%)	0	0	1 (1.5%)	
Blood glucose increased	0	0	0	1 (1.5%)	1 (1.5%)	
Lipase increased	0	0	0	1 (1.5%)	1 (1.5%)	
Blood and lymphatic system disorders	7 (10.4%)	8 (11.9%)	1 (3.3%)	0	8 (11.9%)	
Neutropenia	7 (10.4%)	8 (11.9%)	1 (3.3%)	0	8 (11.9%)	
General disorders and administration site conditions	2 (3.0%)	2 (3.0%)	0	0	2 (3.0%)	
Asthenia	2 (3.0%)	2 (3.0%)	0	0	2 (3.0%)	
Injury, poisoning and procedural complications	0	0	0	1 (1.5%)	1 (1.5%)	
Pelvic fracture	0	0	0	1 (1.5%)	1 (1.5%)	
Road traffic accident	0	0	0	1 (1.5%)	1 (1.5%)	
Nervous system disorders	1 (1.5%)	1 (1.5%)	0	0	1 (1.5%)	
Headache	1 (1.5%)	1 (1.5%)	0	0	1 (1.5%)	
Psychiatric disorders	1 (1.5%)	1 (1.5%)	0	0	1 (1.5%)	
Depression	1 (1.5%)	1 (1.5%)	0	0	1 (1.5%)	
Respiratory, thoracic and mediastinal disorders	0	0	0	1 (1.5%)	1 (1.5%)	
Pulmonary embolism	0	0	0	1 (1.5%)	1 (1.5%)	
Vascular disorders	0	0	0	1 (1.5%)	1 (1.5%)	
Deep vein thrombosis	0	0	0	1 (1.5%)	1 (1.5%)	
	
[TSFAE05TDG4ALL.RTF] [TMC435\HPC3014\DBR_FINAL_ANALYSIS\RE_FINAL_ANALYSIS\PROD\TSFAE05TDG4ALL.SAS] 02NOV2015, 11:21	
