# Supplementary material for: Efficacy of a 12-Week Simeprevir Plus Peginterferon/Ribavirin (PR) Regimen in Treatment-Naïve Patients with Hepatitis C Virus (HCV) Genotype 4 (GT4) Infection and Mild-To-Moderate Fibrosis Displaying Early On-Treatment Virologic Response
Source: PLoS One. 2017 Jan 5;12(1):e0168713. doi: 10.1371/journal.pone.0168713 (PMC5215882; doi:10.1371/journal.pone.0168713)
Supplement: S1 Dataset — (ZIP) [file pone.0168713.s002.zip › tsfae05tdg4gt12.rtf]

TSFAE05TDG4GT12:	TSFAE05TDG4GT12: Number (pcnt) of Genotype 4 Subjects with Adverse Events by Worst WHO Toxicity Grade, Intent-to-treat, Study TMC435HPC3014 Trt Dur >12 Wks	
	Simeprevir
12 Wks
150 mg
PR 12/24 	
	SMV + PR 	Ent Trt 	PR Only 	Follow-Up 	Overall 	
Analysis set: Intent-to-treat	33	33	27	32	33	
Any Grade 1 AE	10 (30.3%)	8 (24.2%)	11 (40.7%)	4 (12.5%)	9 (27.3%)	
General disorders and administration site conditions	13 (39.4%)	14 (42.4%)	5 (18.5%)	1 (3.1%)	13 (39.4%)	
Fatigue	6 (18.2%)	6 (18.2%)	0	0	6 (18.2%)	
Influenza like illness	7 (21.2%)	7 (21.2%)	1 (3.7%)	0	6 (18.2%)	
Asthenia	5 (15.2%)	5 (15.2%)	0	1 (3.1%)	5 (15.2%)	
Pyrexia	4 (12.1%)	4 (12.1%)	0	0	4 (12.1%)	
Injection site erythema	1 (3.0%)	3 (9.1%)	2 (7.4%)	0	3 (9.1%)	
Application site alopecia	0	1 (3.0%)	1 (3.7%)	0	1 (3.0%)	
Injection site pruritus	0	1 (3.0%)	1 (3.7%)	0	1 (3.0%)	
Injection site rash	1 (3.0%)	1 (3.0%)	0	0	1 (3.0%)	
Irritability	0	1 (3.0%)	1 (3.7%)	0	1 (3.0%)	
Mucosal dryness	1 (3.0%)	1 (3.0%)	0	0	1 (3.0%)	
Pain	0	1 (3.0%)	1 (3.7%)	0	1 (3.0%)	
Gastrointestinal disorders	11 (33.3%)	11 (33.3%)	3 (11.1%)	0	11 (33.3%)	
Diarrhoea	6 (18.2%)	6 (18.2%)	1 (3.7%)	0	6 (18.2%)	
Vomiting	4 (12.1%)	5 (15.2%)	1 (3.7%)	0	4 (12.1%)	
Abdominal distension	1 (3.0%)	2 (6.1%)	1 (3.7%)	0	2 (6.1%)	
Abdominal pain upper	1 (3.0%)	2 (6.1%)	1 (3.7%)	0	2 (6.1%)	
Constipation	2 (6.1%)	2 (6.1%)	0	0	2 (6.1%)	
Dyspepsia	2 (6.1%)	2 (6.1%)	0	0	2 (6.1%)	
Nausea	2 (6.1%)	2 (6.1%)	0	0	2 (6.1%)	
Abdominal discomfort	1 (3.0%)	1 (3.0%)	0	0	1 (3.0%)	
Abdominal pain	1 (3.0%)	1 (3.0%)	0	0	1 (3.0%)	
Abdominal tenderness	1 (3.0%)	1 (3.0%)	0	0	1 (3.0%)	
Anal inflammation	1 (3.0%)	1 (3.0%)	0	0	1 (3.0%)	
Dry mouth	1 (3.0%)	1 (3.0%)	0	0	1 (3.0%)	
Haemorrhoids	1 (3.0%)	1 (3.0%)	0	0	1 (3.0%)	
Nervous system disorders	8 (24.2%)	10 (30.3%)	2 (7.4%)	1 (3.1%)	10 (30.3%)	
Headache	5 (15.2%)	7 (21.2%)	2 (7.4%)	0	7 (21.2%)	
Dizziness	2 (6.1%)	2 (6.1%)	0	0	2 (6.1%)	
Paraesthesia	1 (3.0%)	1 (3.0%)	0	1 (3.1%)	2 (6.1%)	
Carpal tunnel syndrome	1 (3.0%)	1 (3.0%)	0	0	1 (3.0%)	
Memory impairment	1 (3.0%)	1 (3.0%)	0	0	1 (3.0%)	
Skin and subcutaneous tissue disorders	7 (21.2%)	10 (30.3%)	6 (22.2%)	0	10 (30.3%)	
Pruritus	4 (12.1%)	5 (15.2%)	3 (11.1%)	0	5 (15.2%)	
Rash	3 (9.1%)	5 (15.2%)	3 (11.1%)	0	5 (15.2%)	
Erythema	2 (6.1%)	3 (9.1%)	1 (3.7%)	0	3 (9.1%)	
Dry skin	0	1 (3.0%)	1 (3.7%)	0	1 (3.0%)	
Erythema nodosum	0	1 (3.0%)	1 (3.7%)	0	1 (3.0%)	
Hyperhidrosis	0	1 (3.0%)	1 (3.7%)	0	1 (3.0%)	
Metabolism and nutrition disorders	8 (24.2%)	8 (24.2%)	0	0	8 (24.2%)	
Decreased appetite	7 (21.2%)	7 (21.2%)	0	0	7 (21.2%)	
Hyperinsulinaemia	1 (3.0%)	1 (3.0%)	0	0	1 (3.0%)	
Hypokalaemia	1 (3.0%)	1 (3.0%)	0	0	1 (3.0%)	
Psychiatric disorders	6 (18.2%)	7 (21.2%)	1 (3.7%)	1 (3.1%)	8 (24.2%)	
Depression	2 (6.1%)	3 (9.1%)	1 (3.7%)	1 (3.1%)	4 (12.1%)	
Insomnia	3 (9.1%)	3 (9.1%)	0	0	3 (9.1%)	
Anxiety disorder	1 (3.0%)	1 (3.0%)	0	0	1 (3.0%)	
Libido decreased	1 (3.0%)	1 (3.0%)	0	0	1 (3.0%)	
Sleep disorder	1 (3.0%)	1 (3.0%)	0	0	1 (3.0%)	
Musculoskeletal and connective tissue disorders	6 (18.2%)	7 (21.2%)	2 (7.4%)	1 (3.1%)	7 (21.2%)	
Back pain	2 (6.1%)	3 (9.1%)	1 (3.7%)	0	3 (9.1%)	
Arthralgia	1 (3.0%)	2 (6.1%)	1 (3.7%)	0	2 (6.1%)	
Arthritis	0	0	0	1 (3.1%)	1 (3.0%)	
Muscle spasms	1 (3.0%)	1 (3.0%)	0	0	1 (3.0%)	
Musculoskeletal stiffness	1 (3.0%)	1 (3.0%)	0	0	1 (3.0%)	
Myalgia	1 (3.0%)	1 (3.0%)	0	0	1 (3.0%)	
Respiratory, thoracic and mediastinal disorders	5 (15.2%)	6 (18.2%)	1 (3.7%)	0	6 (18.2%)	
Dyspnoea	4 (12.1%)	5 (15.2%)	1 (3.7%)	0	5 (15.2%)	
Dyspnoea exertional	1 (3.0%)	1 (3.0%)	0	0	1 (3.0%)	
Nasal congestion	1 (3.0%)	1 (3.0%)	0	0	1 (3.0%)	
Blood and lymphatic system disorders	3 (9.1%)	4 (12.1%)	1 (3.7%)	0	4 (12.1%)	
Neutropenia	2 (6.1%)	3 (9.1%)	1 (3.7%)	0	3 (9.1%)	
Anaemia	2 (6.1%)	2 (6.1%)	0	0	2 (6.1%)	
Leukopenia	1 (3.0%)	1 (3.0%)	0	0	1 (3.0%)	
Thrombocytopenia	1 (3.0%)	1 (3.0%)	0	0	1 (3.0%)	
Ear and labyrinth disorders	4 (12.1%)	4 (12.1%)	1 (3.7%)	0	4 (12.1%)	
Vertigo	3 (9.1%)	3 (9.1%)	0	0	3 (9.1%)	
Tinnitus	1 (3.0%)	2 (6.1%)	1 (3.7%)	0	2 (6.1%)	
Infections and infestations	1 (3.0%)	2 (6.1%)	0	1 (3.1%)	3 (9.1%)	
Bronchitis	0	1 (3.0%)	0	0	1 (3.0%)	
Enterobiasis	0	0	0	1 (3.1%)	1 (3.0%)	
Tooth abscess	1 (3.0%)	1 (3.0%)	0	0	1 (3.0%)	
Eye disorders	0	2 (6.1%)	2 (7.4%)	0	2 (6.1%)	
Vision blurred	0	1 (3.0%)	1 (3.7%)	0	1 (3.0%)	
Visual acuity reduced	0	1 (3.0%)	1 (3.7%)	0	1 (3.0%)	
Cardiac disorders	1 (3.0%)	1 (3.0%)	0	1 (3.1%)	1 (3.0%)	
Palpitations	1 (3.0%)	1 (3.0%)	0	1 (3.1%)	1 (3.0%)	
Injury, poisoning and procedural complications	0	1 (3.0%)	1 (3.7%)	0	1 (3.0%)	
Scratch	0	1 (3.0%)	1 (3.7%)	0	1 (3.0%)	
Investigations	1 (3.0%)	1 (3.0%)	2 (7.4%)	0	1 (3.0%)	
Blood glucose increased	1 (3.0%)	2 (6.1%)	1 (3.7%)	0	2 (6.1%)	
Alanine aminotransferase increased	0	1 (3.0%)	1 (3.7%)	0	1 (3.0%)	
Blood lactate dehydrogenase increased	0	1 (3.0%)	1 (3.7%)	0	1 (3.0%)	
Blood pressure increased	0	1 (3.0%)	0	0	1 (3.0%)	
Lipase increased	1 (3.0%)	1 (3.0%)	0	0	1 (3.0%)	
Blood bilirubin increased	0	0	1 (3.7%)	0	0	
Vascular disorders	1 (3.0%)	1 (3.0%)	1 (3.7%)	0	1 (3.0%)	
Cryoglobulinaemia	1 (3.0%)	1 (3.0%)	0	0	1 (3.0%)	
Hypertension	0	1 (3.0%)	1 (3.7%)	0	1 (3.0%)	
Any Grade 2 AE	8 (24.2%)	7 (21.2%)	3 (11.1%)	2 (6.3%)	7 (21.2%)	
General disorders and administration site conditions	5 (15.2%)	5 (15.2%)	1 (3.7%)	1 (3.1%)	6 (18.2%)	
Influenza like illness	1 (3.0%)	2 (6.1%)	1 (3.7%)	1 (3.1%)	3 (9.1%)	
Asthenia	2 (6.1%)	2 (6.1%)	0	0	2 (6.1%)	
Fatigue	2 (6.1%)	2 (6.1%)	0	0	2 (6.1%)	
Pyrexia	1 (3.0%)	1 (3.0%)	0	0	1 (3.0%)	
Skin and subcutaneous tissue disorders	4 (12.1%)	6 (18.2%)	2 (7.4%)	0	6 (18.2%)	
Pruritus	2 (6.1%)	2 (6.1%)	0	0	2 (6.1%)	
Dry skin	1 (3.0%)	1 (3.0%)	0	0	1 (3.0%)	
Eczema	0	1 (3.0%)	1 (3.7%)	0	1 (3.0%)	
Onychoclasis	0	1 (3.0%)	1 (3.7%)	0	1 (3.0%)	
Rash	1 (3.0%)	1 (3.0%)	0	0	1 (3.0%)	
Psychiatric disorders	3 (9.1%)	4 (12.1%)	1 (3.7%)	0	4 (12.1%)	
Depressed mood	2 (6.1%)	2 (6.1%)	0	0	2 (6.1%)	
Sleep disorder	1 (3.0%)	2 (6.1%)	1 (3.7%)	0	2 (6.1%)	
Depression	1 (3.0%)	1 (3.0%)	0	0	1 (3.0%)	
Blood and lymphatic system disorders	3 (9.1%)	3 (9.1%)	1 (3.7%)	0	3 (9.1%)	
Anaemia	2 (6.1%)	2 (6.1%)	0	0	2 (6.1%)	
Neutropenia	0	1 (3.0%)	1 (3.7%)	0	1 (3.0%)	
Thrombocytopenia	1 (3.0%)	1 (3.0%)	0	0	1 (3.0%)	
Gastrointestinal disorders	3 (9.1%)	3 (9.1%)	0	1 (3.1%)	3 (9.1%)	
Abdominal pain	1 (3.0%)	1 (3.0%)	0	0	1 (3.0%)	
Abdominal pain lower	1 (3.0%)	1 (3.0%)	0	0	1 (3.0%)	
Colitis	1 (3.0%)	1 (3.0%)	0	0	1 (3.0%)	
Constipation	1 (3.0%)	1 (3.0%)	0	0	1 (3.0%)	
Vomiting	0	0	0	1 (3.1%)	1 (3.0%)	
Respiratory, thoracic and mediastinal disorders	2 (6.1%)	2 (6.1%)	0	1 (3.1%)	3 (9.1%)	
Asthma	0	0	0	1 (3.1%)	1 (3.0%)	
Dyspnoea	1 (3.0%)	1 (3.0%)	0	0	1 (3.0%)	
Oropharyngeal pain	1 (3.0%)	1 (3.0%)	0	0	1 (3.0%)	
Infections and infestations	2 (6.1%)	2 (6.1%)	0	0	2 (6.1%)	
Acute sinusitis	1 (3.0%)	1 (3.0%)	0	0	1 (3.0%)	
Gastroenteritis	1 (3.0%)	1 (3.0%)	0	0	1 (3.0%)	
Musculoskeletal and connective tissue disorders	0	0	0	2 (6.3%)	2 (6.1%)	
Back pain	0	0	0	1 (3.1%)	1 (3.0%)	
Tendonitis	0	0	0	1 (3.1%)	1 (3.0%)	
Vascular disorders	0	2 (6.1%)	2 (7.4%)	0	2 (6.1%)	
Pallor	0	1 (3.0%)	1 (3.7%)	0	1 (3.0%)	
Phlebitis	0	1 (3.0%)	1 (3.7%)	0	1 (3.0%)	
Investigations	2 (6.1%)	1 (3.0%)	0	0	1 (3.0%)	
Aspartate aminotransferase increased	0	2 (6.1%)	2 (7.4%)	0	2 (6.1%)	
Blood bilirubin increased	2 (6.1%)	2 (6.1%)	0	0	2 (6.1%)	
Haemoglobin decreased	1 (3.0%)	1 (3.0%)	0	0	1 (3.0%)	
Platelet count decreased	1 (3.0%)	1 (3.0%)	0	0	1 (3.0%)	
Weight decreased	1 (3.0%)	1 (3.0%)	0	0	1 (3.0%)	
Metabolism and nutrition disorders	0	1 (3.0%)	1 (3.7%)	0	1 (3.0%)	
Decreased appetite	0	1 (3.0%)	1 (3.7%)	0	1 (3.0%)	
Nervous system disorders	1 (3.0%)	1 (3.0%)	0	0	1 (3.0%)	
Dizziness	1 (3.0%)	1 (3.0%)	0	0	1 (3.0%)	
Any Grade 3 AE	9 (27.3%)	11 (33.3%)	3 (11.1%)	1 (3.1%)	11 (33.3%)	
Investigations	4 (12.1%)	5 (15.2%)	2 (7.4%)	0	5 (15.2%)	
Alanine aminotransferase increased	1 (3.0%)	2 (6.1%)	1 (3.7%)	0	2 (6.1%)	
Neutrophil count decreased	2 (6.1%)	2 (6.1%)	1 (3.7%)	0	2 (6.1%)	
Amylase increased	1 (3.0%)	1 (3.0%)	0	0	1 (3.0%)	
Blood bilirubin increased	1 (3.0%)	1 (3.0%)	0	0	1 (3.0%)	
Blood and lymphatic system disorders	2 (6.1%)	3 (9.1%)	1 (3.7%)	0	3 (9.1%)	
Neutropenia	2 (6.1%)	3 (9.1%)	1 (3.7%)	0	3 (9.1%)	
General disorders and administration site conditions	2 (6.1%)	2 (6.1%)	0	0	2 (6.1%)	
Asthenia	2 (6.1%)	2 (6.1%)	0	0	2 (6.1%)	
Injury, poisoning and procedural complications	0	0	0	1 (3.1%)	1 (3.0%)	
Pelvic fracture	0	0	0	1 (3.1%)	1 (3.0%)	
Road traffic accident	0	0	0	1 (3.1%)	1 (3.0%)	
Nervous system disorders	1 (3.0%)	1 (3.0%)	0	0	1 (3.0%)	
Headache	1 (3.0%)	1 (3.0%)	0	0	1 (3.0%)	
Psychiatric disorders	1 (3.0%)	1 (3.0%)	0	0	1 (3.0%)	
Depression	1 (3.0%)	1 (3.0%)	0	0	1 (3.0%)	
Any Grade 4 AE	2 (6.1%)	3 (9.1%)	1 (3.7%)	0	3 (9.1%)	
Investigations	2 (6.1%)	3 (9.1%)	1 (3.7%)	0	3 (9.1%)	
Neutrophil count decreased	2 (6.1%)	3 (9.1%)	1 (3.7%)	0	3 (9.1%)	
Any Grade 3-4 AE	11 (33.3%)	14 (42.4%)	4 (14.8%)	1 (3.1%)	14 (42.4%)	
Investigations	6 (18.2%)	8 (24.2%)	3 (11.1%)	0	8 (24.2%)	
Neutrophil count decreased	4 (12.1%)	5 (15.2%)	2 (7.4%)	0	5 (15.2%)	
Alanine aminotransferase increased	1 (3.0%)	2 (6.1%)	1 (3.7%)	0	2 (6.1%)	
Amylase increased	1 (3.0%)	1 (3.0%)	0	0	1 (3.0%)	
Blood bilirubin increased	1 (3.0%)	1 (3.0%)	0	0	1 (3.0%)	
Blood and lymphatic system disorders	2 (6.1%)	3 (9.1%)	1 (3.7%)	0	3 (9.1%)	
Neutropenia	2 (6.1%)	3 (9.1%)	1 (3.7%)	0	3 (9.1%)	
General disorders and administration site conditions	2 (6.1%)	2 (6.1%)	0	0	2 (6.1%)	
Asthenia	2 (6.1%)	2 (6.1%)	0	0	2 (6.1%)	
Injury, poisoning and procedural complications	0	0	0	1 (3.1%)	1 (3.0%)	
Pelvic fracture	0	0	0	1 (3.1%)	1 (3.0%)	
Road traffic accident	0	0	0	1 (3.1%)	1 (3.0%)	
Nervous system disorders	1 (3.0%)	1 (3.0%)	0	0	1 (3.0%)	
Headache	1 (3.0%)	1 (3.0%)	0	0	1 (3.0%)	
Psychiatric disorders	1 (3.0%)	1 (3.0%)	0	0	1 (3.0%)	
Depression	1 (3.0%)	1 (3.0%)	0	0	1 (3.0%)	
	
[TSFAE05TDG4GT12.RTF] [TMC435\HPC3014\DBR_FINAL_ANALYSIS\RE_FINAL_ANALYSIS\PROD\TSFAE05TDG4GT12.SAS] 02NOV2015, 11:21	
