# Supplementary material for: Efficacy of a 12-Week Simeprevir Plus Peginterferon/Ribavirin (PR) Regimen in Treatment-Naïve Patients with Hepatitis C Virus (HCV) Genotype 4 (GT4) Infection and Mild-To-Moderate Fibrosis Displaying Early On-Treatment Virologic Response
Source: PLoS One. 2017 Jan 5;12(1):e0168713. doi: 10.1371/journal.pone.0168713 (PMC5215882; doi:10.1371/journal.pone.0168713)
Supplement: S1 Dataset — (ZIP) [file pone.0168713.s002.zip › tsfae05tdg412.rtf]

TSFAE05TDG412:	TSFAE05TDG412: Number (pcnt) of Genotype 4 Subjects with Adverse Events by Worst WHO Toxicity Grade, Intent-to-treat, Study TMC435HPC3014 Trt Dur 12 Wks	
	Simeprevir
12 Wks
150 mg
PR 12/24 	
	SMV + PR 	Ent Trt 	PR Only 	Follow-Up 	Overall 	
No data to report	-	-	-	-	-	
Analysis set: Intent-to-treat	34	34	3	34	34	
Any Grade 1 AE	15 (44.1%)	16 (47.1%)	2 (66.7%)	1 (2.9%)	15 (44.1%)	
General disorders and administration site conditions	14 (41.2%)	14 (41.2%)	1 (33.3%)	0	14 (41.2%)	
Asthenia	5 (14.7%)	5 (14.7%)	0	0	5 (14.7%)	
Fatigue	5 (14.7%)	5 (14.7%)	0	0	5 (14.7%)	
Pyrexia	2 (5.9%)	3 (8.8%)	1 (33.3%)	0	3 (8.8%)	
Influenza like illness	2 (5.9%)	2 (5.9%)	0	0	2 (5.9%)	
Chest pain	1 (2.9%)	1 (2.9%)	0	0	1 (2.9%)	
Discomfort	1 (2.9%)	1 (2.9%)	0	0	1 (2.9%)	
Irritability	1 (2.9%)	1 (2.9%)	0	0	1 (2.9%)	
Skin and subcutaneous tissue disorders	10 (29.4%)	12 (35.3%)	1 (33.3%)	0	12 (35.3%)	
Pruritus	7 (20.6%)	7 (20.6%)	0	0	7 (20.6%)	
Rash	3 (8.8%)	4 (11.8%)	0	0	4 (11.8%)	
Dry skin	2 (5.9%)	2 (5.9%)	0	0	2 (5.9%)	
Erythema	2 (5.9%)	2 (5.9%)	0	0	2 (5.9%)	
Alopecia	1 (2.9%)	1 (2.9%)	0	0	1 (2.9%)	
Eczema	0	1 (2.9%)	1 (33.3%)	0	1 (2.9%)	
Psoriasis	0	1 (2.9%)	0	0	1 (2.9%)	
Gastrointestinal disorders	11 (32.4%)	11 (32.4%)	0	0	11 (32.4%)	
Diarrhoea	4 (11.8%)	4 (11.8%)	0	0	4 (11.8%)	
Abdominal pain upper	2 (5.9%)	2 (5.9%)	0	0	2 (5.9%)	
Dry mouth	2 (5.9%)	2 (5.9%)	0	0	2 (5.9%)	
Vomiting	2 (5.9%)	2 (5.9%)	0	0	2 (5.9%)	
Abdominal pain	1 (2.9%)	1 (2.9%)	0	0	1 (2.9%)	
Anal pruritus	1 (2.9%)	1 (2.9%)	0	0	1 (2.9%)	
Constipation	1 (2.9%)	1 (2.9%)	0	0	1 (2.9%)	
Dyspepsia	1 (2.9%)	1 (2.9%)	0	0	1 (2.9%)	
Haemorrhoids	1 (2.9%)	1 (2.9%)	0	0	1 (2.9%)	
Mouth ulceration	1 (2.9%)	1 (2.9%)	0	0	1 (2.9%)	
Nausea	1 (2.9%)	1 (2.9%)	0	0	1 (2.9%)	
Metabolism and nutrition disorders	6 (17.6%)	6 (17.6%)	0	0	6 (17.6%)	
Decreased appetite	6 (17.6%)	6 (17.6%)	0	0	6 (17.6%)	
Psychiatric disorders	6 (17.6%)	6 (17.6%)	0	0	6 (17.6%)	
Anxiety	2 (5.9%)	2 (5.9%)	0	0	2 (5.9%)	
Depression	2 (5.9%)	2 (5.9%)	0	0	2 (5.9%)	
Insomnia	2 (5.9%)	2 (5.9%)	0	0	2 (5.9%)	
Depressed mood	1 (2.9%)	1 (2.9%)	0	0	1 (2.9%)	
Nervousness	1 (2.9%)	1 (2.9%)	0	0	1 (2.9%)	
Nervous system disorders	5 (14.7%)	5 (14.7%)	0	0	5 (14.7%)	
Headache	4 (11.8%)	4 (11.8%)	0	0	4 (11.8%)	
Disturbance in attention	1 (2.9%)	1 (2.9%)	0	0	1 (2.9%)	
Dizziness	1 (2.9%)	1 (2.9%)	0	0	1 (2.9%)	
Memory impairment	1 (2.9%)	1 (2.9%)	0	0	1 (2.9%)	
Paraesthesia	1 (2.9%)	1 (2.9%)	0	0	1 (2.9%)	
Musculoskeletal and connective tissue disorders	4 (11.8%)	4 (11.8%)	0	0	4 (11.8%)	
Back pain	3 (8.8%)	3 (8.8%)	0	0	3 (8.8%)	
Arthralgia	0	1 (2.9%)	0	0	1 (2.9%)	
Myalgia	1 (2.9%)	1 (2.9%)	0	0	1 (2.9%)	
Blood and lymphatic system disorders	2 (5.9%)	3 (8.8%)	0	0	3 (8.8%)	
Anaemia	1 (2.9%)	2 (5.9%)	0	0	2 (5.9%)	
Neutropenia	2 (5.9%)	2 (5.9%)	0	0	2 (5.9%)	
Leukopenia	1 (2.9%)	1 (2.9%)	0	0	1 (2.9%)	
Thrombocytopenia	1 (2.9%)	1 (2.9%)	0	0	1 (2.9%)	
Ear and labyrinth disorders	3 (8.8%)	3 (8.8%)	1 (33.3%)	0	3 (8.8%)	
Tinnitus	2 (5.9%)	2 (5.9%)	1 (33.3%)	0	2 (5.9%)	
Vertigo	1 (2.9%)	1 (2.9%)	0	0	1 (2.9%)	
Cardiac disorders	2 (5.9%)	2 (5.9%)	0	0	2 (5.9%)	
Palpitations	2 (5.9%)	2 (5.9%)	0	0	2 (5.9%)	
Infections and infestations	1 (2.9%)	2 (5.9%)	0	0	2 (5.9%)	
Furuncle	1 (2.9%)	1 (2.9%)	0	0	1 (2.9%)	
Oral candidiasis	0	1 (2.9%)	0	0	1 (2.9%)	
Investigations	1 (2.9%)	1 (2.9%)	0	1 (2.9%)	2 (5.9%)	
Blood thyroid stimulating hormone increased	0	0	0	1 (2.9%)	1 (2.9%)	
Haemoglobin decreased	1 (2.9%)	1 (2.9%)	0	0	1 (2.9%)	
Neutrophil count decreased	1 (2.9%)	1 (2.9%)	0	0	1 (2.9%)	
Respiratory, thoracic and mediastinal disorders	2 (5.9%)	2 (5.9%)	0	0	2 (5.9%)	
Oropharyngeal pain	2 (5.9%)	2 (5.9%)	0	0	2 (5.9%)	
Eye disorders	1 (2.9%)	1 (2.9%)	0	0	1 (2.9%)	
Eye disorder	1 (2.9%)	1 (2.9%)	0	0	1 (2.9%)	
Eye pain	1 (2.9%)	1 (2.9%)	0	0	1 (2.9%)	
Hepatobiliary disorders	1 (2.9%)	1 (2.9%)	0	0	1 (2.9%)	
Hepatic pain	1 (2.9%)	1 (2.9%)	0	0	1 (2.9%)	
Immune system disorders	1 (2.9%)	1 (2.9%)	0	0	1 (2.9%)	
Seasonal allergy	1 (2.9%)	1 (2.9%)	0	0	1 (2.9%)	
Injury, poisoning and procedural complications	1 (2.9%)	1 (2.9%)	0	0	1 (2.9%)	
Ligament sprain	1 (2.9%)	1 (2.9%)	0	0	1 (2.9%)	
Any Grade 2 AE	9 (26.5%)	9 (26.5%)	0	1 (2.9%)	7 (20.6%)	
Blood and lymphatic system disorders	3 (8.8%)	3 (8.8%)	0	0	3 (8.8%)	
Neutropenia	2 (5.9%)	2 (5.9%)	0	0	2 (5.9%)	
Anaemia	1 (2.9%)	1 (2.9%)	0	0	1 (2.9%)	
Skin and subcutaneous tissue disorders	3 (8.8%)	3 (8.8%)	0	0	3 (8.8%)	
Pruritus	2 (5.9%)	2 (5.9%)	0	0	2 (5.9%)	
Erythema	1 (2.9%)	1 (2.9%)	0	0	1 (2.9%)	
Rash	1 (2.9%)	1 (2.9%)	0	0	1 (2.9%)	
Gastrointestinal disorders	2 (5.9%)	2 (5.9%)	0	0	2 (5.9%)	
Constipation	1 (2.9%)	1 (2.9%)	0	0	1 (2.9%)	
Mouth ulceration	1 (2.9%)	1 (2.9%)	0	0	1 (2.9%)	
General disorders and administration site conditions	2 (5.9%)	2 (5.9%)	0	0	2 (5.9%)	
Chest pain	1 (2.9%)	1 (2.9%)	0	0	1 (2.9%)	
Fatigue	1 (2.9%)	1 (2.9%)	0	0	1 (2.9%)	
Influenza like illness	1 (2.9%)	1 (2.9%)	0	0	1 (2.9%)	
Infections and infestations	2 (5.9%)	2 (5.9%)	0	0	2 (5.9%)	
Fungal skin infection	1 (2.9%)	1 (2.9%)	0	0	1 (2.9%)	
Gingival infection	1 (2.9%)	1 (2.9%)	0	0	1 (2.9%)	
Hepatobiliary disorders	1 (2.9%)	1 (2.9%)	0	0	1 (2.9%)	
Hyperbilirubinaemia	1 (2.9%)	1 (2.9%)	0	0	1 (2.9%)	
Nervous system disorders	1 (2.9%)	1 (2.9%)	0	0	1 (2.9%)	
Headache	1 (2.9%)	1 (2.9%)	0	0	1 (2.9%)	
Psychiatric disorders	1 (2.9%)	1 (2.9%)	0	0	1 (2.9%)	
Insomnia	1 (2.9%)	1 (2.9%)	0	0	1 (2.9%)	
Reproductive system and breast disorders	1 (2.9%)	1 (2.9%)	0	0	1 (2.9%)	
Dysmenorrhoea	1 (2.9%)	1 (2.9%)	0	0	1 (2.9%)	
Vascular disorders	0	0	0	1 (2.9%)	1 (2.9%)	
Hypertension	0	0	0	1 (2.9%)	1 (2.9%)	
Any Grade 3 AE	6 (17.6%)	6 (17.6%)	0	3 (8.8%)	9 (26.5%)	
Blood and lymphatic system disorders	5 (14.7%)	5 (14.7%)	0	0	5 (14.7%)	
Neutropenia	5 (14.7%)	5 (14.7%)	0	0	5 (14.7%)	
Investigations	1 (2.9%)	1 (2.9%)	0	2 (5.9%)	3 (8.8%)	
Amylase increased	0	0	0	1 (2.9%)	1 (2.9%)	
Blood glucose increased	0	0	0	1 (2.9%)	1 (2.9%)	
Lipase increased	0	0	0	1 (2.9%)	1 (2.9%)	
Neutrophil count decreased	1 (2.9%)	1 (2.9%)	0	0	1 (2.9%)	
Respiratory, thoracic and mediastinal disorders	0	0	0	1 (2.9%)	1 (2.9%)	
Pulmonary embolism	0	0	0	1 (2.9%)	1 (2.9%)	
Vascular disorders	0	0	0	1 (2.9%)	1 (2.9%)	
Deep vein thrombosis	0	0	0	1 (2.9%)	1 (2.9%)	
Any Grade 4 AE	0	0	0	0	0	
No data to report	-	-	-	-	-	
Any Grade 3-4 AE	6 (17.6%)	6 (17.6%)	0	3 (8.8%)	9 (26.5%)	
Blood and lymphatic system disorders	5 (14.7%)	5 (14.7%)	0	0	5 (14.7%)	
Neutropenia	5 (14.7%)	5 (14.7%)	0	0	5 (14.7%)	
Investigations	1 (2.9%)	1 (2.9%)	0	2 (5.9%)	3 (8.8%)	
Amylase increased	0	0	0	1 (2.9%)	1 (2.9%)	
Blood glucose increased	0	0	0	1 (2.9%)	1 (2.9%)	
Lipase increased	0	0	0	1 (2.9%)	1 (2.9%)	
Neutrophil count decreased	1 (2.9%)	1 (2.9%)	0	0	1 (2.9%)	
Respiratory, thoracic and mediastinal disorders	0	0	0	1 (2.9%)	1 (2.9%)	
Pulmonary embolism	0	0	0	1 (2.9%)	1 (2.9%)	
Vascular disorders	0	0	0	1 (2.9%)	1 (2.9%)	
Deep vein thrombosis	0	0	0	1 (2.9%)	1 (2.9%)	
	
[TSFAE05TDG412.RTF] [TMC435\HPC3014\DBR_FINAL_ANALYSIS\RE_FINAL_ANALYSIS\PROD\TSFAE05TDG412.SAS] 02NOV2015, 11:20	
