# Supplementary material for: Efficacy of a 12-Week Simeprevir Plus Peginterferon/Ribavirin (PR) Regimen in Treatment-Naïve Patients with Hepatitis C Virus (HCV) Genotype 4 (GT4) Infection and Mild-To-Moderate Fibrosis Displaying Early On-Treatment Virologic Response
Source: PLoS One. 2017 Jan 5;12(1):e0168713. doi: 10.1371/journal.pone.0168713 (PMC5215882; doi:10.1371/journal.pone.0168713)
Supplement: S1 Dataset — (ZIP) [file pone.0168713.s002.zip › TSFAE11TDG4.rtf]

TSFAE11TDG4:	Number and percentage of GT4 Subjects with Adverse Events at Least Possibly Related to Ribavirin by Worst WHO Toxicity Grade - Intent-to-treat (Study TMC435HPC3014)	
	Simeprevir
12 Wks
150 mg
PR 12/24 	
	SMV + PR 	Ent Trt 	PR Only 	Follow-Up 	Overall 	
Analysis set: Intent-to-treat	67	67	30	66	67	
Any Grade 1 AE	19 (28.4%)	18 (26.9%)	3 (10.0%)	0	18 (26.9%)	
General disorders and administration site conditions	9 (13.4%)	10 (14.9%)	1 (3.3%)	0	10 (14.9%)	
Fatigue	4 (6.0%)	4 (6.0%)	0	0	4 (6.0%)	
Asthenia	3 (4.5%)	3 (4.5%)	0	0	3 (4.5%)	
Injection site erythema	0	1 (1.5%)	1 (3.3%)	0	1 (1.5%)	
Irritability	1 (1.5%)	1 (1.5%)	0	0	1 (1.5%)	
Mucosal dryness	1 (1.5%)	1 (1.5%)	0	0	1 (1.5%)	
Pyrexia	1 (1.5%)	1 (1.5%)	0	0	1 (1.5%)	
Skin and subcutaneous tissue disorders	8 (11.9%)	9 (13.4%)	2 (6.7%)	0	9 (13.4%)	
Pruritus	4 (6.0%)	5 (7.5%)	2 (6.7%)	0	5 (7.5%)	
Rash	3 (4.5%)	4 (6.0%)	1 (3.3%)	0	4 (6.0%)	
Erythema	3 (4.5%)	3 (4.5%)	0	0	3 (4.5%)	
Alopecia	1 (1.5%)	1 (1.5%)	0	0	1 (1.5%)	
Dry skin	1 (1.5%)	1 (1.5%)	0	0	1 (1.5%)	
Gastrointestinal disorders	8 (11.9%)	8 (11.9%)	1 (3.3%)	0	8 (11.9%)	
Diarrhoea	5 (7.5%)	5 (7.5%)	1 (3.3%)	0	5 (7.5%)	
Vomiting	3 (4.5%)	3 (4.5%)	0	0	3 (4.5%)	
Dyspepsia	2 (3.0%)	2 (3.0%)	0	0	2 (3.0%)	
Abdominal discomfort	1 (1.5%)	1 (1.5%)	0	0	1 (1.5%)	
Abdominal distension	1 (1.5%)	1 (1.5%)	0	0	1 (1.5%)	
Constipation	1 (1.5%)	1 (1.5%)	0	0	1 (1.5%)	
Dry mouth	1 (1.5%)	1 (1.5%)	0	0	1 (1.5%)	
Haemorrhoids	1 (1.5%)	1 (1.5%)	0	0	1 (1.5%)	
Nausea	1 (1.5%)	1 (1.5%)	0	0	1 (1.5%)	
Blood and lymphatic system disorders	5 (7.5%)	6 (9.0%)	0	0	6 (9.0%)	
Anaemia	3 (4.5%)	4 (6.0%)	0	0	4 (6.0%)	
Neutropenia	2 (3.0%)	2 (3.0%)	0	0	2 (3.0%)	
Leukopenia	1 (1.5%)	1 (1.5%)	0	0	1 (1.5%)	
Thrombocytopenia	1 (1.5%)	1 (1.5%)	0	0	1 (1.5%)	
Metabolism and nutrition disorders	6 (9.0%)	6 (9.0%)	0	0	6 (9.0%)	
Decreased appetite	6 (9.0%)	6 (9.0%)	0	0	6 (9.0%)	
Respiratory, thoracic and mediastinal disorders	4 (6.0%)	5 (7.5%)	1 (3.3%)	0	5 (7.5%)	
Dyspnoea	3 (4.5%)	4 (6.0%)	1 (3.3%)	0	4 (6.0%)	
Dyspnoea exertional	1 (1.5%)	1 (1.5%)	0	0	1 (1.5%)	
Nervous system disorders	4 (6.0%)	4 (6.0%)	0	0	4 (6.0%)	
Headache	3 (4.5%)	3 (4.5%)	0	0	3 (4.5%)	
Memory impairment	2 (3.0%)	2 (3.0%)	0	0	2 (3.0%)	
Disturbance in attention	1 (1.5%)	1 (1.5%)	0	0	1 (1.5%)	
Dizziness	1 (1.5%)	1 (1.5%)	0	0	1 (1.5%)	
Ear and labyrinth disorders	3 (4.5%)	3 (4.5%)	0	0	3 (4.5%)	
Vertigo	2 (3.0%)	2 (3.0%)	0	0	2 (3.0%)	
Tinnitus	1 (1.5%)	1 (1.5%)	0	0	1 (1.5%)	
Eye disorders	1 (1.5%)	2 (3.0%)	1 (3.3%)	0	2 (3.0%)	
Eye disorder	1 (1.5%)	1 (1.5%)	0	0	1 (1.5%)	
Eye pain	1 (1.5%)	1 (1.5%)	0	0	1 (1.5%)	
Vision blurred	0	1 (1.5%)	1 (3.3%)	0	1 (1.5%)	
Musculoskeletal and connective tissue disorders	2 (3.0%)	2 (3.0%)	0	0	2 (3.0%)	
Myalgia	2 (3.0%)	2 (3.0%)	0	0	2 (3.0%)	
Cardiac disorders	1 (1.5%)	1 (1.5%)	0	0	1 (1.5%)	
Palpitations	1 (1.5%)	1 (1.5%)	0	0	1 (1.5%)	
Injury, poisoning and procedural complications	0	1 (1.5%)	1 (3.3%)	0	1 (1.5%)	
Scratch	0	1 (1.5%)	1 (3.3%)	0	1 (1.5%)	
Investigations	1 (1.5%)	1 (1.5%)	0	0	1 (1.5%)	
Blood lactate dehydrogenase increased	0	1 (1.5%)	1 (3.3%)	0	1 (1.5%)	
Haemoglobin decreased	1 (1.5%)	1 (1.5%)	0	0	1 (1.5%)	
Psychiatric disorders	0	1 (1.5%)	1 (3.3%)	0	1 (1.5%)	
Depression	0	1 (1.5%)	1 (3.3%)	0	1 (1.5%)	
Vascular disorders	1 (1.5%)	1 (1.5%)	0	0	1 (1.5%)	
Cryoglobulinaemia	1 (1.5%)	1 (1.5%)	0	0	1 (1.5%)	
Any Grade 2 AE	13 (19.4%)	12 (17.9%)	1 (3.3%)	0	12 (17.9%)	
Skin and subcutaneous tissue disorders	5 (7.5%)	7 (10.4%)	2 (6.7%)	0	7 (10.4%)	
Pruritus	3 (4.5%)	3 (4.5%)	0	0	3 (4.5%)	
Dry skin	1 (1.5%)	1 (1.5%)	0	0	1 (1.5%)	
Eczema	0	1 (1.5%)	1 (3.3%)	0	1 (1.5%)	
Onychoclasis	0	1 (1.5%)	1 (3.3%)	0	1 (1.5%)	
Rash	1 (1.5%)	1 (1.5%)	0	0	1 (1.5%)	
General disorders and administration site conditions	4 (6.0%)	4 (6.0%)	0	0	4 (6.0%)	
Fatigue	3 (4.5%)	3 (4.5%)	0	0	3 (4.5%)	
Asthenia	1 (1.5%)	1 (1.5%)	0	0	1 (1.5%)	
Chest pain	1 (1.5%)	1 (1.5%)	0	0	1 (1.5%)	
Psychiatric disorders	2 (3.0%)	3 (4.5%)	1 (3.3%)	0	3 (4.5%)	
Depressed mood	2 (3.0%)	2 (3.0%)	0	0	2 (3.0%)	
Sleep disorder	0	1 (1.5%)	1 (3.3%)	0	1 (1.5%)	
Blood and lymphatic system disorders	3 (4.5%)	2 (3.0%)	0	0	2 (3.0%)	
Anaemia	3 (4.5%)	3 (4.5%)	0	0	3 (4.5%)	
Investigations	2 (3.0%)	2 (3.0%)	0	0	2 (3.0%)	
Haemoglobin decreased	1 (1.5%)	1 (1.5%)	0	0	1 (1.5%)	
Weight decreased	1 (1.5%)	1 (1.5%)	0	0	1 (1.5%)	
Nervous system disorders	2 (3.0%)	2 (3.0%)	0	0	2 (3.0%)	
Dizziness	1 (1.5%)	1 (1.5%)	0	0	1 (1.5%)	
Headache	1 (1.5%)	1 (1.5%)	0	0	1 (1.5%)	
Respiratory, thoracic and mediastinal disorders	2 (3.0%)	2 (3.0%)	0	0	2 (3.0%)	
Dyspnoea	1 (1.5%)	1 (1.5%)	0	0	1 (1.5%)	
Oropharyngeal pain	1 (1.5%)	1 (1.5%)	0	0	1 (1.5%)	
Gastrointestinal disorders	1 (1.5%)	1 (1.5%)	0	0	1 (1.5%)	
Mouth ulceration	1 (1.5%)	1 (1.5%)	0	0	1 (1.5%)	
Hepatobiliary disorders	1 (1.5%)	1 (1.5%)	0	0	1 (1.5%)	
Hyperbilirubinaemia	1 (1.5%)	1 (1.5%)	0	0	1 (1.5%)	
Infections and infestations	1 (1.5%)	1 (1.5%)	0	0	1 (1.5%)	
Gingival infection	1 (1.5%)	1 (1.5%)	0	0	1 (1.5%)	
Metabolism and nutrition disorders	0	1 (1.5%)	1 (3.3%)	0	1 (1.5%)	
Decreased appetite	0	1 (1.5%)	1 (3.3%)	0	1 (1.5%)	
Vascular disorders	0	1 (1.5%)	1 (3.3%)	0	1 (1.5%)	
Pallor	0	1 (1.5%)	1 (3.3%)	0	1 (1.5%)	
Any Grade 3 AE	3 (4.5%)	6 (9.0%)	3 (10.0%)	0	6 (9.0%)	
Blood and lymphatic system disorders	1 (1.5%)	2 (3.0%)	1 (3.3%)	0	2 (3.0%)	
Neutropenia	1 (1.5%)	2 (3.0%)	1 (3.3%)	0	2 (3.0%)	
General disorders and administration site conditions	2 (3.0%)	2 (3.0%)	0	0	2 (3.0%)	
Asthenia	2 (3.0%)	2 (3.0%)	0	0	2 (3.0%)	
Investigations	0	2 (3.0%)	2 (6.7%)	0	2 (3.0%)	
Neutrophil count decreased	0	2 (3.0%)	2 (6.7%)	0	2 (3.0%)	
Any Grade 4 AE	0	0	0	0	0	
No data to report	-	-	-	-	-	
Any Grade 3-4 AE	3 (4.5%)	6 (9.0%)	3 (10.0%)	0	6 (9.0%)	
Blood and lymphatic system disorders	1 (1.5%)	2 (3.0%)	1 (3.3%)	0	2 (3.0%)	
Neutropenia	1 (1.5%)	2 (3.0%)	1 (3.3%)	0	2 (3.0%)	
General disorders and administration site conditions	2 (3.0%)	2 (3.0%)	0	0	2 (3.0%)	
Asthenia	2 (3.0%)	2 (3.0%)	0	0	2 (3.0%)	
Investigations	0	2 (3.0%)	2 (6.7%)	0	2 (3.0%)	
Neutrophil count decreased	0	2 (3.0%)	2 (6.7%)	0	2 (3.0%)	
	
[TSFAE11TDG4.RTF] [TMC435\HPC3014\DBR_FINAL_ANALYSIS\RE_FINAL_ANALYSIS\PDEV\TEMPFILE.SAS] 21OCT2016, 16:35	
