# Supplementary material for: Efficacy of a 12-Week Simeprevir Plus Peginterferon/Ribavirin (PR) Regimen in Treatment-Naïve Patients with Hepatitis C Virus (HCV) Genotype 4 (GT4) Infection and Mild-To-Moderate Fibrosis Displaying Early On-Treatment Virologic Response
Source: PLoS One. 2017 Jan 5;12(1):e0168713. doi: 10.1371/journal.pone.0168713 (PMC5215882; doi:10.1371/journal.pone.0168713)
Supplement: S1 Fig — (DOCX) [file pone.0168713.s003.docx]

**S1 Fig |** Study design.

**
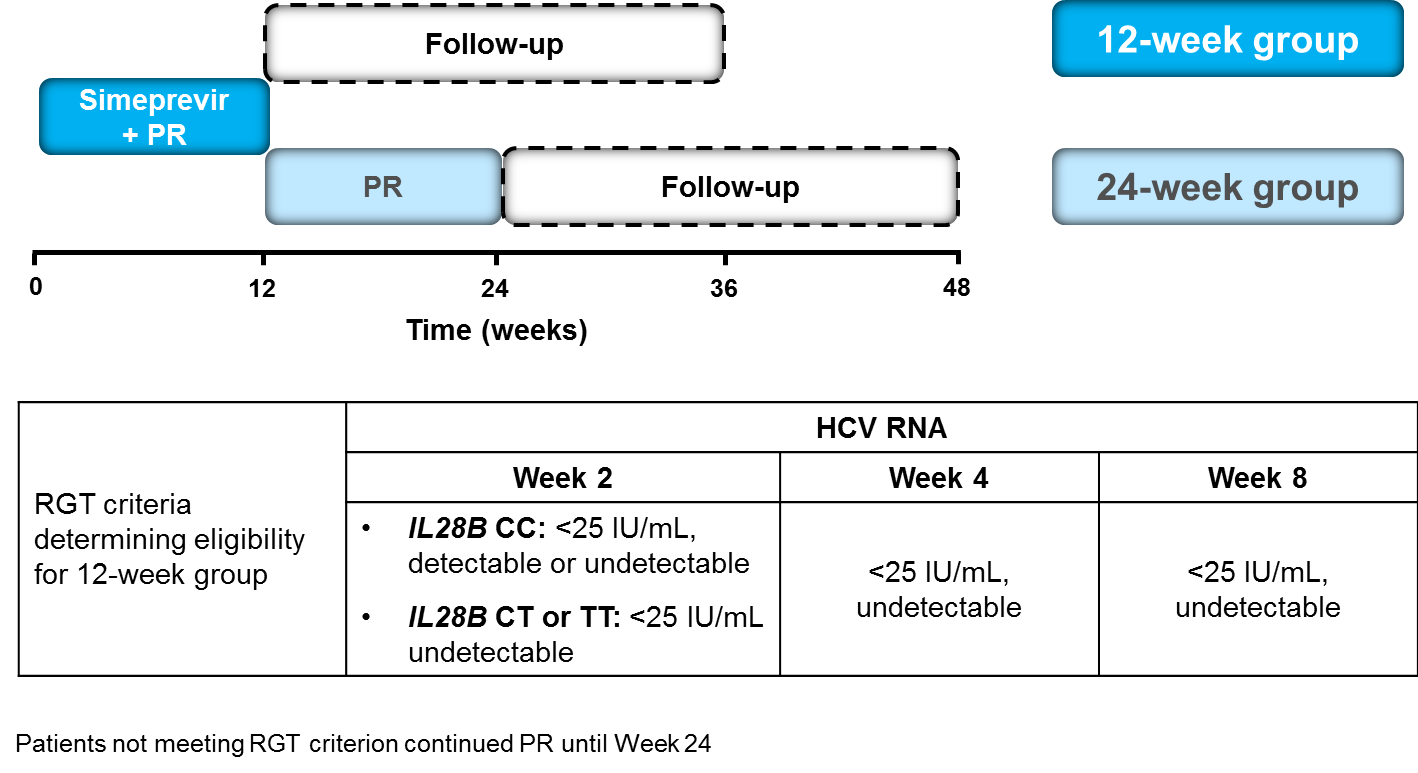
**

Patients not meeting the RGT criteria continued PR until Week 24

PR, peginterferon/ribavirin; RGT, response guided therapy
