# Supplementary material for: Efficacy of a 12-Week Simeprevir Plus Peginterferon/Ribavirin (PR) Regimen in Treatment-Naïve Patients with Hepatitis C Virus (HCV) Genotype 4 (GT4) Infection and Mild-To-Moderate Fibrosis Displaying Early On-Treatment Virologic Response
Source: PLoS One. 2017 Jan 5;12(1):e0168713. doi: 10.1371/journal.pone.0168713 (PMC5215882; doi:10.1371/journal.pone.0168713)
Supplement: S2 Fig — Mean (±SE) On-treatment and EOT Laboratory Measures in Patients Receiving 12 and >12 Weeks’ Treatment: [A] Haemoglobin (g/L); [B] Neutrophils and Precursors (x109/L); [C] Platelets (x109/L); [D] Total Bilirubin (μmol/L). (DOCX) [file pone.0168713.s004.docx]

**S2 Fig |** Mean (±SE) on-treatment and EOT laboratory measures in patients receiving 12 and >12 weeks’ treatment: **A** haemoglobin (g/L); **B** neutrophils and precursors (x10^9^/L); **C** platelets (x10^9^/L); **D** total bilirubin (µmol/L).

**A**


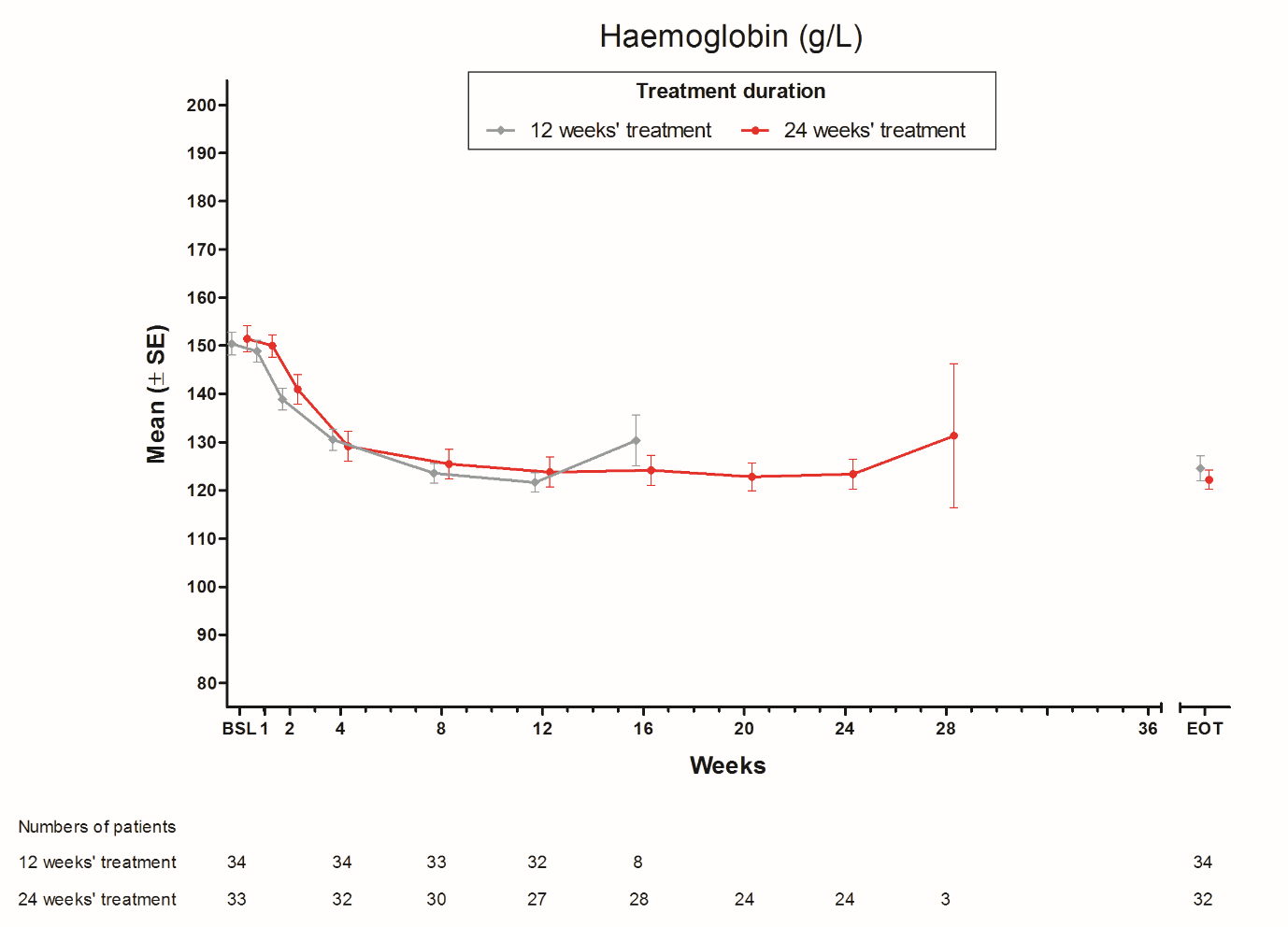


**B**

**
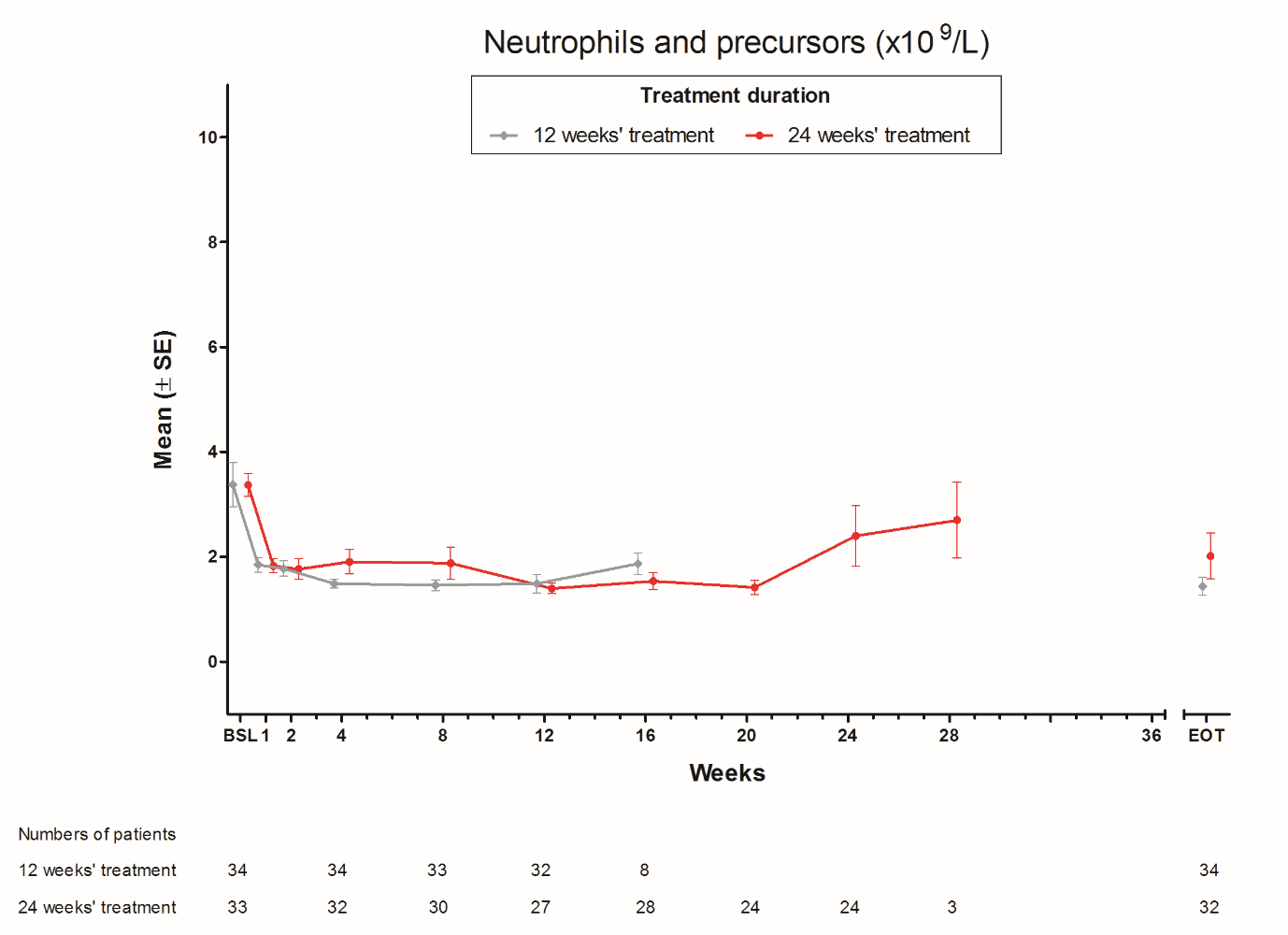
**

**C**

**
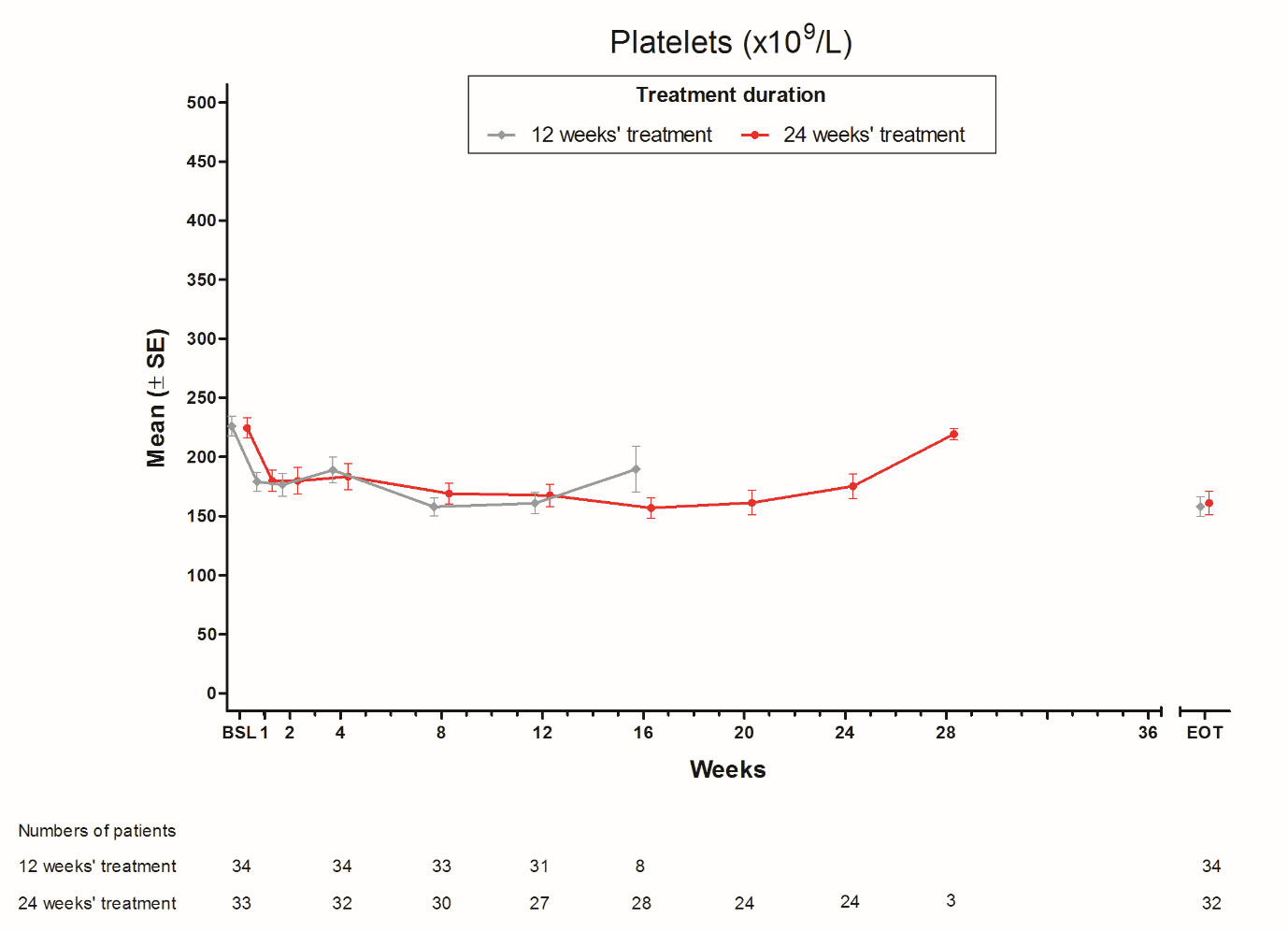
**

**D**

**
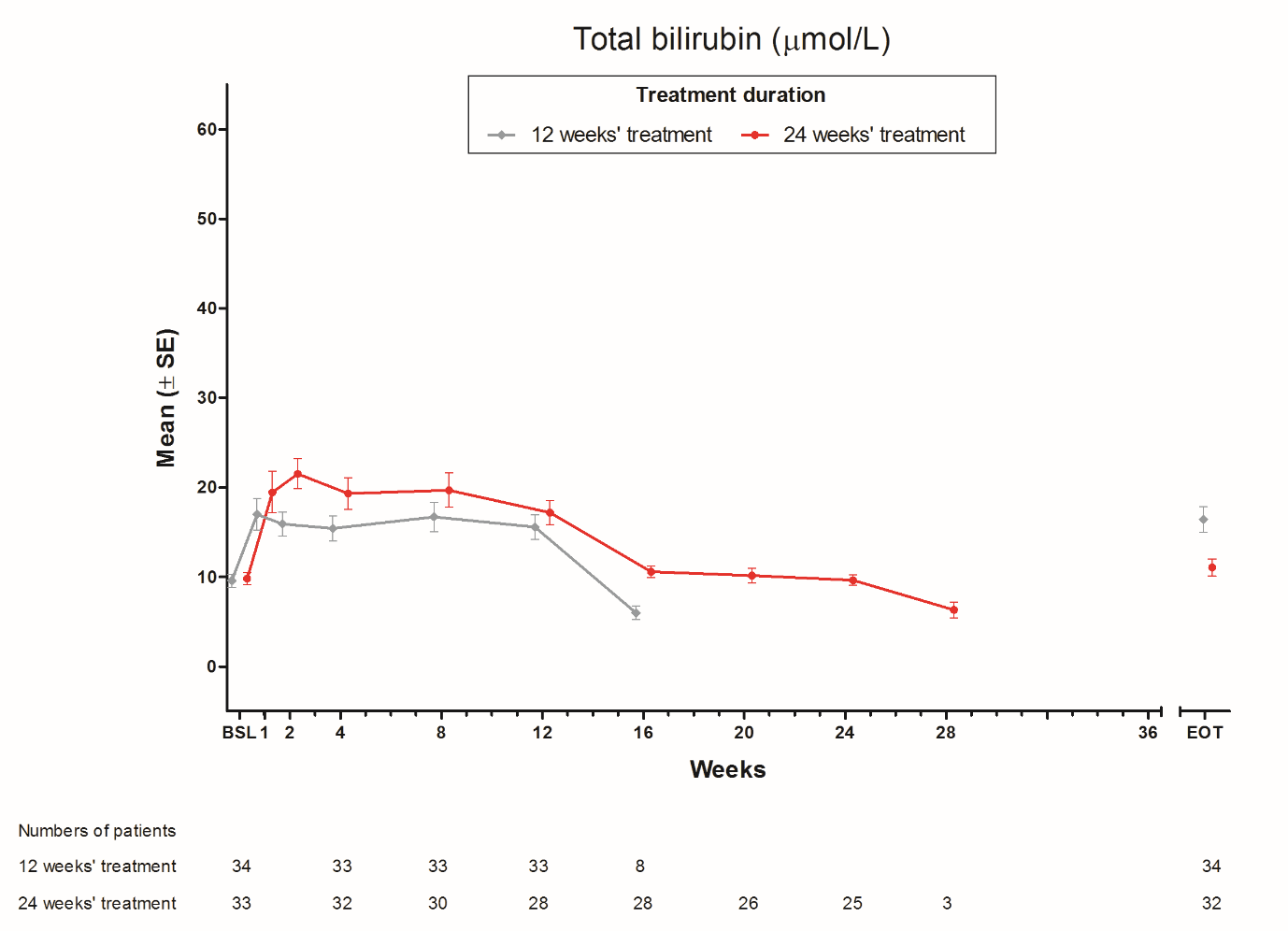
**

BSL, baseline; EOT, end of treatment; SE, standard error
