# Supplementary material for: Efficacy of a 12-Week Simeprevir Plus Peginterferon/Ribavirin (PR) Regimen in Treatment-Naïve Patients with Hepatitis C Virus (HCV) Genotype 4 (GT4) Infection and Mild-To-Moderate Fibrosis Displaying Early On-Treatment Virologic Response
Source: PLoS One. 2017 Jan 5;12(1):e0168713. doi: 10.1371/journal.pone.0168713 (PMC5215882; doi:10.1371/journal.pone.0168713)
Supplement: S1 Table — HCV GT4 Subtype of All Patients According to a) Duration of Treatment; b) Country of Study Site. (DOCX) [file pone.0168713.s006.docx]

**S1 Table |** Demographics and disease characteristics of patients in the 24-week group who experienced viral relapse (*n* = 3)

| **Patient** | **Sex** | **Age** | **Race** | **Region** | **Weight (kg)** | **BMI (kg/m^2^)** | **METAVIR score** | **Baseline HCV RNA (IU/mL)** | **HCV GT1 subtype** | ***IL28B* genotype** |
| --- | --- | --- | --- | --- | --- | --- | --- | --- | --- | --- |
| 1 * | Male | 59 | Caucasian | Europe | 55.0 | 19.0 | F0–F1 | 1,490,000 | 4a | TT |
| 2 | Female | 49 | Caucasian | Europe | 85.8 | 31.9 | F0–F1 | 3,990,000 | 4d | CT |
| 3 | Male | 45 | Caucasian | Europe | 80.0 | 24.4 | F0–F1 | 4,730,000 | 4/other | CT |

BMI, body mass index; HCV, hepatitis C virus.

*Patient 1 discontinued all treatment at week 8 due to an adverse event (depression); Patients 2 and 3 completed all treatment.
