# Supplementary material for: Efficacy of a 12-Week Simeprevir Plus Peginterferon/Ribavirin (PR) Regimen in Treatment-Naïve Patients with Hepatitis C Virus (HCV) Genotype 4 (GT4) Infection and Mild-To-Moderate Fibrosis Displaying Early On-Treatment Virologic Response
Source: PLoS One. 2017 Jan 5;12(1):e0168713. doi: 10.1371/journal.pone.0168713 (PMC5215882; doi:10.1371/journal.pone.0168713)
Supplement: S2 Table — (DOCX) [file pone.0168713.s007.docx]

**S2 Table |** HCV GT4 subtype of all patients according to a) duration of treatment; b) country of study site

**a)**

| **HCV GT4 subtype** | **12-week treatment** | **24-week treatment** | **All patients** |
| --- | --- | --- | --- |
| 4 | 1 (3) | 1 (3) | 2 (3) |
| 4a | 14 (41) | 13 (39) | 27 (40) |
| 4a/4c/4d | 1 (3) | 1 (3) | 2 (3) |
| 4c | 1 (3) | 1 (3) | 2 (3) |
| 4d | 13 (38) | 12 (36) | 25 (37) |
| 4 – other subtypes | 4 (12) | 5 (15) | 9 (13) |
| Total | 34 | 33 | 67 |

**b)**

| **HCV GT4 subtype** | **Austria** | **Belgium** | **Spain** | **France** | **Italy** | **Saudi Arabia** | **All patients** |
| --- | --- | --- | --- | --- | --- | --- | --- |
| 4 | 1 (13) | 0 | 0 | 0 | 0 | 1 (13) | 2 (3) |
| 4a | 6 (75) | 2 (25) | 2 (15) | 7 (54) | 1 (13) | 9 (53) | 27 (40) |
| 4a/4c/4d | 0 | 0 | 1 (8) | 0 | 1 (13) | 0 | 2 (3) |
| 4c | 0 | 1 (13) | 0 | 1 (8) | 0 | 0 | 2 (3) |
| 4d | 1 (13) | 0 | 10 (77) | 3 (23) | 6 (75) | 5 (29) | 25 (37) |
| 4 – other subtypes | 0 | 5 (63) | 0 | 2 (15) | 0 | 2 (12) | 9 (13) |
| Total | 8 | 8 | 13 | 13 | 8 | 17 | 67 |

GT4, genotype 4; HCV, hepatitis C virus.

*Note:* HCV GT4 subtype is based on the NS5B assay, and if not available on LIPA HCV II or Trugene results.
