# Supplementary material for: Efficacy of a 12-Week Simeprevir Plus Peginterferon/Ribavirin (PR) Regimen in Treatment-Naïve Patients with Hepatitis C Virus (HCV) Genotype 4 (GT4) Infection and Mild-To-Moderate Fibrosis Displaying Early On-Treatment Virologic Response
Source: PLoS One. 2017 Jan 5;12(1):e0168713. doi: 10.1371/journal.pone.0168713 (PMC5215882; doi:10.1371/journal.pone.0168713)
Supplement: S3 Table — (DOCX) [file pone.0168713.s008.docx]

**S3 Table |** Grade 3/4 AEs considered at least possibly related to a) peg-IFN and b) RBV

**a)**

|  | **Patients  (*N* = 67)** |
| --- | --- |
| **Any Grade 3 or 4 AE** | **18 (27)** |
| **Any Grade 3 AE** | **15 (22)** |
| Neutropenia | 8 (12) |
| Neutrophil count decreased | 3 (4) |
| Asthenia | 2 (3) |
| Alanine aminotransferase increased | 1 (1) |
| Headache | 1 (1) |
| Depression | 1 (1) |
| **Any Grade 4 AE** | **3 (4)** |
| Neutrophil count decreased | 3 (4) |

**b)**

|  | **Patients  (*N* = 67)** |
| --- | --- |
| **Any Grade 3 or 4 AE** | **6 (9)** |
| **Any Grade 3 AE** | **6 (9)** |
| Neutropenia | 2 (3) |
| Neutrophil count decreased | 2 (3) |
| Asthenia | 2 (3) |
| **Any Grade 4 AE** | **0 (0)** |
