# Supplementary material for: Efficacy of a 12-Week Simeprevir Plus Peginterferon/Ribavirin (PR) Regimen in Treatment-Naïve Patients with Hepatitis C Virus (HCV) Genotype 4 (GT4) Infection and Mild-To-Moderate Fibrosis Displaying Early On-Treatment Virologic Response
Source: PLoS One. 2017 Jan 5;12(1):e0168713. doi: 10.1371/journal.pone.0168713 (PMC5215882; doi:10.1371/journal.pone.0168713)
Supplement: S1 Text — (DOCX) [file pone.0168713.s009.docx]

**S1 Supporting information – List of Institutional review boards**

1. Ethikkommission der Medizinischen Universität Wien, Vienna, Austria

2. Commission d’Éthique Biomédicale Hospitalo-Facultaire, Brussels, Belgium

3. Comité de Protection des Personnes Ile-de-France IV, Paris, France

4. Segreteria Tecnico Scientifica Comitato di Bioetica, Palermo, Italy

5. Comitato Etico Policlinico Umberto I, Roma, Italy

6. Comitato Etico Indipendente Fondazione Policlinico Tor Vergata, Roma, Italy

7. Comitato Etico Azienda Ospedaliero-Universitaria di Parma, Parma, Italy

8. Comitato Etico di Area Vasta Nord-Ovest per la sperimentazione clinica, Pisa, Italy

9. Ethik-Kommission des Landes Berlin, Berlin, Germany

10. Ethik-Kommission der Ärztekammer Hamburg, Hamburg, Germany

11. Hospital Universitari Vall D’Hebrón - Unidad de Soporte al CEIC (SCEI) - Vall d’Hebron Institut de Recerca (VHIR), Barcelona, Spain

12. West of Scotland Research Ethics Service, Glasgow, UK

13. King Saud University, King Khalid University Hospital -College of Medicine IRB, Riyadh, Saudi Arabia

14. King Abdullah International Medical research center- Ministry of National Guard Health Affairs IRB, Riyadh, Saudi Arabia

15. King Faisal Specialist Hospital & Research Center IRB, Riyadh, Saudi Arabia

16. Centralna Komisja Bioetyczna - Komisja Bioetyczna Uniwersytetu Mikołaja Kopernika w Toruniu przy Collegium Medicum im, Bydgoszcz, Poland
